# Glucose and Lactate Concentrations in Plasma, Cerebrospinal Fluid, and Brain Parenchyma Following Aneurysmal Subarachnoid Hemorrhage: A Cross-compartmental Correlation Study

**DOI:** 10.1007/s12028-025-02442-7

**Published:** 2026-02-04

**Authors:** Niko Schmaling, Miriam M. Moser, Lena Weyer, Robin Ristl, Walter Plöchl, Andrea Reinprecht, Johannes Herta, Karl Rössler, Arthur Hosmann

**Affiliations:** 1 Department of Neurosurgery, Medical University of Vienna, Währinger-Gürtel 18-20, 1090 Vienna, Austria; 2 Institute for Medical Statistics, Center for Medical Statistics, Informatics and Intelligent Systems, Medical University of Vienna, Vienna, Austria; 3 Department of Anesthesia, General Intensive Care Medicine and Pain Management, Medical University of Vienna, Vienna, Austria

**Keywords:** Microdialysis, Subarachnoid hemorrhage, Glucose, Lactate, Cerebrospinal fluid

## Abstract

**Objectives:**

Patients with aneurysmal subarachnoid hemorrhage (aSAH) are at high risk of secondary ischemia, and timely insight into cerebral metabolism may improve clinical management. Cerebral microdialysis offers continuous metabolic monitoring but is invasive, focal, and confined to specialized centers. Peripheral blood and cerebrospinal fluid (CSF) could provide less invasive, more accessible surrogates for assessing cerebral metabolic status. This study aimed to evaluate glucose and lactate dynamics across blood, CSF, and brain parenchyma in patients with severe aSAH.

**Patients and Methods:**

A total of 39 patients with aSAH undergoing multimodal neuromonitoring were retrospectively analyzed. Glucose and lactate levels from plasma, CSF, and cerebral microdialysis were matched within 90-min intervals relative to each microdialysis measurement. Associations were analyzed using linear mixed-effects models.

**Results:**

Plasma (*p* < 0.001) and CSF (*p* < 0.001) glucose levels were significantly associated with cerebral glucose concentrations. Moderate correlations were observed between plasma–brain (*r* = 0.54) and plasma–CSF (*r* = 0.55), while CSF–brain correlation was weaker (*r* = 0.36). For lactate, significant associations were found between CSF and brain (*p* = 0.04; *r* = 0.24) and between plasma and CSF (*p* < 0.001; *r* = 0.33), but plasma–brain lactate showed no significant relationship.

The plasma–brain glucose association weakened slightly over time (*p* = 0.008) and strengthened during episodes of low brain tissue oxygenation (pbtO_2_ < 15 mm Hg; *p* = 0.01). Insulin had no effect on glucose relationships but significantly attenuated the plasma–brain lactate association (*p* < 0.001). The presence of metabolic crisis (lactate/pyruvate ratio > 40) strengthened the CSF–brain lactate association (*p* = 0.04). CSF cell count had no significant effect.

**Conclusions:**

In severe aSAH, glucose and lactate levels in blood and CSF reflect cerebral values in a compartment- and metabolite-specific manner, but with high variability influenced by clinical conditions. These findings suggest that blood and CSF provide only limited information about cerebral metabolism, highlighting the complementary value of cerebral microdialysis for individualized, brain-targeted monitoring.

**Supplementary Information:**

The online version contains supplementary material available at 10.1007/s12028-025-02442-7.

## Introduction

In patients with aneurysmal subarachnoid hemorrhage (aSAH), a major contributor to poor functional outcomes is secondary brain injury, which has increasingly been a focus of research in recent years, leading to a more comprehensive understanding of the underlying pathophysiological mechanisms [1–4]. In this context, disruptions in cerebral energy metabolism are increasingly recognized as a central feature of this secondary injury phase [5, 6].

The continuous availability of glucose, as a primary energy substrate of the brain, is essential for neuronal integrity and function [7]. A linear relationship between plasma and brain parenchymal glucose concentrations has been described in both animal studies [8] and in studies involving healthy human subjects [9, 10]. However, clinical observation suggests that this relationship may not be preserved in the injured human brain. Schlenk et al. [11, 12] demonstrated that both cerebral hyperglycemia and hypoglycemia can occur independently of systemic glucose levels in patients after aSAH, indicating possible alterations in glucose transport or utilization in the injured brain.

Lactate is well recognized as a crucial alternative energy substrate in the brain, particularly during metabolic stress or injury [5]. Its accumulation reflects a shift toward anaerobic metabolism and serves as an early marker of cerebral ischemia and energy failure [6].

Both glucose and lactate concentrations have been studied following aSAH by focusing on the relationship between two individual compartments [13–15].

However, a comprehensive, compartment-spanning analysis of glucose and lactate dynamics across blood, CSF, and brain parenchyma in patients with aSAH is still lacking. It is unclear to what extent less invasive measurements from blood or CSF can reliably reflect cerebral metabolic status, particularly in the presence of dynamic clinical factors such as systemic glucose control, brain tissue oxygenation, or metabolic crisis.

This exploratory study aimed to assess the strength and clinical relevance of associations between glucose and lactate concentrations across plasma, CSF, and brain parenchyma in patients with severe aSAH. We hypothesized that systemic and compartmental metabolite levels partially reflect cerebral concentrations in a metabolite- and compartment-specific manner. We hypothesized that metabolite levels in blood and CSF partially reflect cerebral concentrations in a metabolite- and compartment-specific manner, with measurable but variable relationships that mirror the complex metabolic coupling between systemic, cerebrospinal, and cerebral compartments. Improved insight into these intercompartmental dynamics may enhance metabolic monitoring strategies and guide targeted therapeutic interventions in the neurocritical care of aSAH patients.

## Methods

In this explorative retrospective study of a prospectively collected database, all consecutive patients with aSAH who received invasive arterial blood pressure monitoring, external ventricular drainage (EVD), and cerebral microdialysis (MD) as part of standard care in the neurosurgical intensive care unit at the Medical University of Vienna between 2015 and 2021 were included. The study protocol was approved by the Ethics Committee of the Medical University of Vienna (approval no. 2018/2020).

## Clinical Management

Patient care followed a standardized institutional protocol. Aneurysm repair was intended to be performed by microsurgical clipping or endovascular coiling within 72 h of aSAH onset. Invasive arterial blood pressure monitoring was used to guide hemodynamic management as standard of care. In patients presenting with poor clinical grades, EVD was placed upon admission to facilitate CSF drainage and monitor intracranial pressure (ICP).

The indication for multimodal neuromonitoring, including MD, was poor-grade aSAH or secondary neurological deterioration requiring prolonged sedation for optimal cerebral protection. Long-term sedation was initiated in patients with impaired consciousness (Glasgow Coma Scale score < 9) in combination with diffuse cerebral edema and obliteration of basal cisterns and sulci. Sedation initially consisted of continuous propofol and remifentanil infusions. To reduce the risk of propofol infusion syndrome, sedatives were transitioned after 3–5 days to midazolam (up to 20 mg/h) and sufentanil (up to 0.25 µg/kg/min). In cases where sedation was inadequate, adjunctive ketamine infusion (up to 200 mg/h) was employed. Routine wake-up tests were omitted to minimize secondary cerebral injury.

Mean arterial pressure was monitored at the level of the right atrium for clinical interventions and at the level of the tragus for cerebral perfusion pressure (CPP) calculations. The therapeutic targets included maintaining ICP below 20 mm Hg and brain tissue oxygen tension (pbtO_2_) above 20 mm Hg. CPP thresholds were tailored to individual patients using multimodal monitoring, but a minimum CPP of 60 mm Hg was consistently maintained. No hypertonic lactate infusions were used for ICP control.

Glycemic control was maintained using continuous insulin infusion via a perfusor, targeting a plasma glucose level between 4 and 10 mmol/L.

MD data were incorporated into bedside clinical decision-making as part of multimodal neuromonitoring (MMNM). Trends in glucose, lactate, pyruvate, glutamate, and glycerol were interpreted alongside ICP, CPP, pbtO_2_, arterial blood gases, and systemic glucose levels. Abnormal patterns suggesting metabolic distress or ischemia prompted individualized adjustments of CPP, ventilation, or sedation depth, and guided the indication for neuroimaging or endovascular spasmolysis when appropriate.

## Multimodality Neuromonitoring

All patients received comprehensive MMNM, encompassing assessments of ICP, CPP, pbtO_2_, and cerebral metabolism via MD. The primary indications for initiating such monitoring included poor-grade aSAH or secondary neurological decline necessitating prolonged sedation for neuroprotection.

For MMNM, a NEUROVENT-PTO 2L catheter (RAUMEDIC AG, Helmbrechts, Germany) was implanted alongside a 70 MD Bolt Microdialysis Catheter (M Dialysis AB, Stockholm, Sweden) using a two-lumen bolt system (BOLT KIT PTO 2L, RAUMEDIC AG, Helmbrechts, Germany). The probes were inserted into the frontal white matter and placed ipsilateral to the ruptured aneurysm or ipsilateral to the maximal extension of subarachnoid blood. A 70 Brain MD Catheter (M Dialysis AB) was used in cases of decompressive craniectomy. The perfusion of the MD catheter was kept at a constant rate of 0.3 µL/min using Perfusion Fluid CNS (M Dialysis AB), delivered via a microinfusion pump (107 Microdialysis Pump, M Dialysis AB). Microdialysate was collected in microvials (M Dialysis AB).

## Collection and Analysis of Glucose and Lactate Samples

Plasma samples were routinely collected every 4 h via an arterial line. Glucose and lactate concentrations were measured using a blood gas analyzer (ABL800 FLEX, Radiometer Medical ApS, Brønshøj, Denmark).

CSF samples were obtained at regular 2-day intervals through EVD placed in the frontal horn of the lateral ventricle as part of standard clinical care. Routinely, glucose, lactate, and cell count values from the CSF were analyzed.

Cerebral microdialysate samples were collected every 1–2 h in microvials and immediately analyzed for glucose and lactate concentrations using a bedside analyzer (ISCUSflex, M Dialysis AB, Sweden). In the applied MD setting (10 mm microdialysis membrane, perfusion rate 0.3 µL/min), the recovery for glucose and lactate is estimated at approximately 70% [16]. Reported values were not adjusted to true interstitial concentrations to ensure comparability with routine clinical measurements. However, the relative nature of these values must be considered when interpreting the data.

## Statistical Analysis

Continuous variables are reported as median and interquartile range (IQR); categorical variables as absolute frequencies and percentages. Due to variability in the monitoring duration post-aSAH, the number of datapoints differed across patients. To account for the longitudinal structure and heterogeneity of the data, linear mixed models with random intercepts and slopes were used.

Measurements across compartments were linked within a 90-min window, corresponding to the microdialysis sampling interval. This resulted in 2632 paired datapoints for plasma and cerebral glucose, 2614 for plasma and cerebral lactate, and 219 for CSF and cerebral glucose as well as CSF and cerebral lactate, respectively. The primary aim was to test whether MD values can be predicted by plasma and CSF glucose and lactate levels.

Mixed models were fitted with MD glucose or lactate as outcomes. For the primary analyses, univariable models with CSF and plasma glucose or lactate, respectively, were used as fixed covariates and a random intercept and a random slope for glucose and lactate respectively were estimated for every patient. Additionally, a multivariable model with both of the covariates, CSF and plasma glucose and lactate, respectively, and a random intercept for every patient fitted.

For the subset with 66 datapoints from 29 patients (after linking compartments), only random intercepts were included due to limited degrees of freedom. Fixed-effects estimates are reported with 95% profile confidence intervals and *p*-values. Bland–Altman correlations [17] were also computed. To assess the sensitivity of the models to outliers, outlying glucose (plasma glucose ≥ 11.5 mmol/L, CSF glucose ≥ 6 mmol/L) and lactate (plasma lactate ≥ 2 mmol/L, CSF lactate ≥ 6 mmol/L) data were removed and the new dataset was used to estimate the regression parameters (supplemental material: tables of the sensitivity analyses).

Secondary analyses examined the relationship between plasma and CSF glucose/lactate using univariable mixed models with CSF glucose/lactate as outcome, plasma glucose/lactate as fixed effect, and random intercepts and slopes. Further analyses assessed the influence of additional variables on parenchymal values and their relationship to other compartments. The additional variables are glycemic state (hypo-, normo-, hyperglycemia), time post-bleed, insulin therapy, pbtO_2_ (with hypoxia defined as an arithmetic mean of < 15 mm Hg for 90 min prior to the MD value), lactate–pyruvate ratio, and CSF cell count. To explore the influence of insulin, multivariable mixed models with the brain parenchyma glucose/lactate as outcome were modeled. For the secondary analyses, plasma and CSF glucose/lactate, insulin, and the interaction of glucose/lactate and insulin were used as fixed covariates. A random intercept and random slope for glucose/lactate was estimated for every patient. Secondary analyses, with the other variables (hypoxia, cerebral metabolic crisis, and cell count) were estimated in the same manner. An exception is the exploration of hypo-, normo-, and hyperglycemia. For this analysis, variable breaks are modeled in the slope at the change points of hypo-, normo-, and hyperglycemia. Systemic hypoglycemia was defined as a plasma glucose concentration of < 60 mg/dL or < 3.33 mmol/L, while hyperglycemia was defined as a plasma glucose concentration of > 180 mg/dL or > 10 mmol/L.

The time intervals between consecutive glucose and lactate measurements, as well as the number of datapoints per patient, varied.

Due to missing data, one patient was excluded from the analysis of the pbtO2 variable.

All tests were two-tailed, with *p* < 0.05 considered significant. No correction for multiple testing was applied due to the exploratory nature of the analyses. Statistical analyses were performed using R version 4.3.3.

## Results

### Study Population

Between 2015 and 2021, a total of 362 patients with aSAH were treated at our neurosurgical ICU, of whom 39 (11%) underwent multimodal neuromonitoring including MD and were therefore included in the present analysis. Table 1 gives an overview of the study population. The median patient age was 53 years (IQR 43–58 years). The median Hunt and Hess score at admission was 4 (IQR 3–5). MMNM was started at a median interval of 3 days (IQR 1–5 days) after the bleeding and lasted for 14 days (IQR 9–19 days). Delayed cerebral ischemia occurred in 13 of 39 patients (33%), with a median onset on day 12 (IQR 7–14 days) after the bleeding event.

**Table 1:** Patient characteristics

	Number (*n*) (%)
*Number of included patients*	39 (100%)
*Gender*	
Female	29 (74.4%)
Male	10 (25.6%)
*Age in years*	
Median [IQR]	53 [43–58]
*Ruptured aneurysm*	
AcomA	15 (38.5%)
BA	1 (2.6%)
ICA	1 (2.6%)
MCA	8 (20.5%)
P1	1 (2.6%)
PcomA	7 (17.9%)
Pericallosa	2 (5.1%)
PICA	3 (7.7%)
SCA	1 (2.6%)
*Days from ictus to aneurysm repair*	
0	18 (46.2%)
1	13 (33.3%)
≥ 2	7 (17.9%)
Untreated	1 (2.6%)
*Start of MMNM after bleeding event (days)*	
Median [IQR]	3 [1–5]
*Length of MMNM (days)*	
Median [IQR]	14 [9–18]
*Outcome after 3 month (mRS)* 0	2 (5.6%)
1	5 (13.9%)
2	5 (13.9%)
3	1 (2.8%)
4	7 (19.4%)
5	11 (30.6%)
6	5 (13.9%)
Loss of follow-up	3 (7.7%)

*AcomA* Anterior communicating artery, *BA* Basilar artery, *ICA* Internal carotid artery, *IQR* Interquartile range, *MCA* Middle cerebral artery, *MMNM* Multimodality neuromonitoring, *mRS* Modified rankin scale, *P*1 P1 segment of fetal-type posterior cerebral artery, *PcomA* Posterior communicating artery, *PICA* Posterior inferior cerebellar artery, *SCA* Superior cerebellar artery

The MD catheter was placed in the presumed watershed region between the anterior cerebral artery (ACA) and middle cerebral artery (MCA) territory in 34 patients, in the ACA territory in 3 patients, and in the MCA territory in 2 patients; 22 catheters were positioned in the left hemisphere and 17 in the right. Among the 13 patients who developed DCI-related infarction, the catheter was located ipsilateral to the affected vascular territory in 11 cases.

**Fig. 1:**
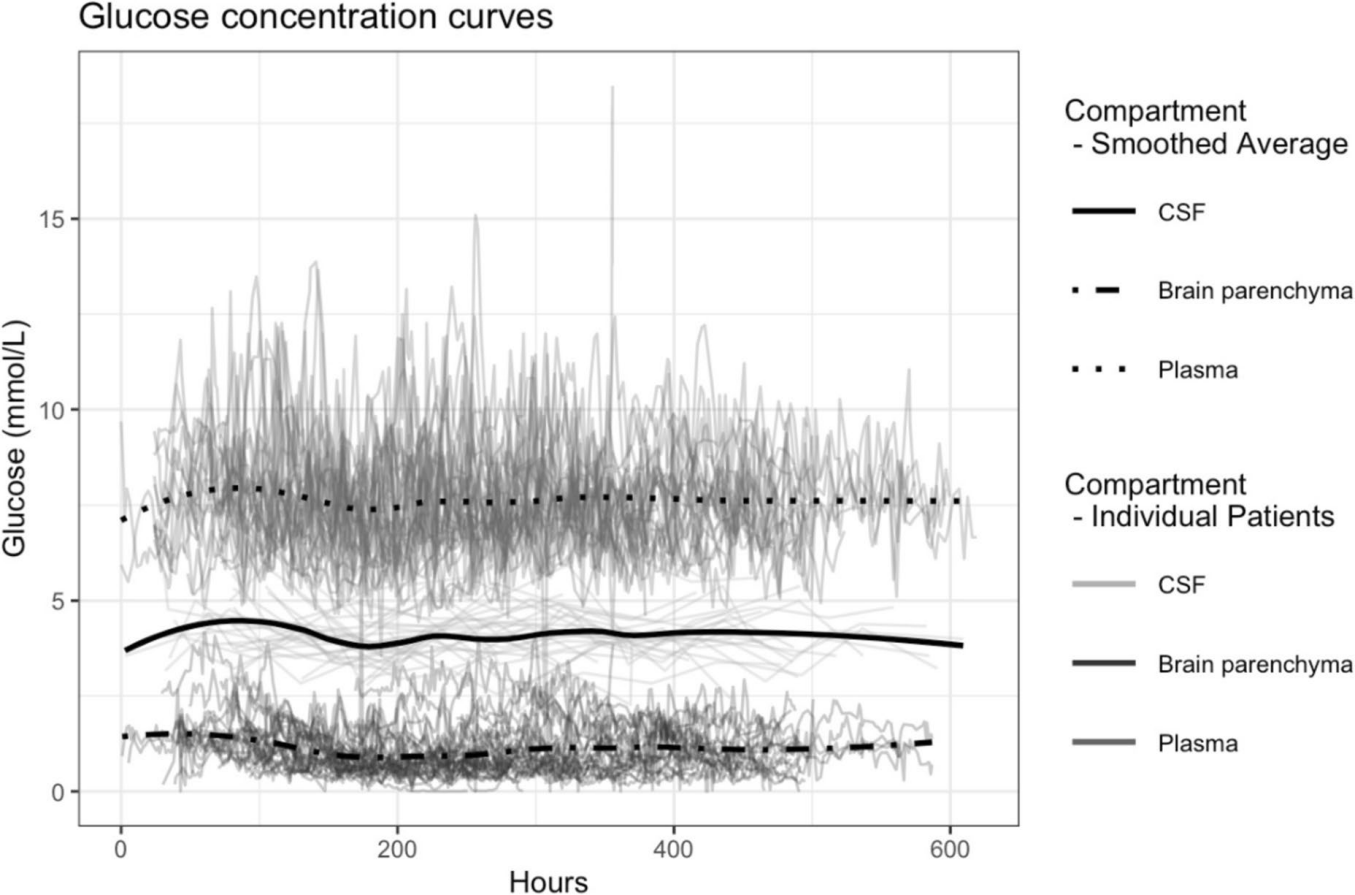
Glucose concentration curves across compartments. Time course of glucose concentrations (mmol/L) over h relative to the day of bleeding. Smoothed average trajectories are shown for plasma, cerebrospinal fluid (CSF), and brain parenchyma (microdialysis), along with individual patient curves. Supplementary material includes detailed trajectories for all patients and the corresponding fixed and random effects from the mixed-effects models

**Fig. 2:**
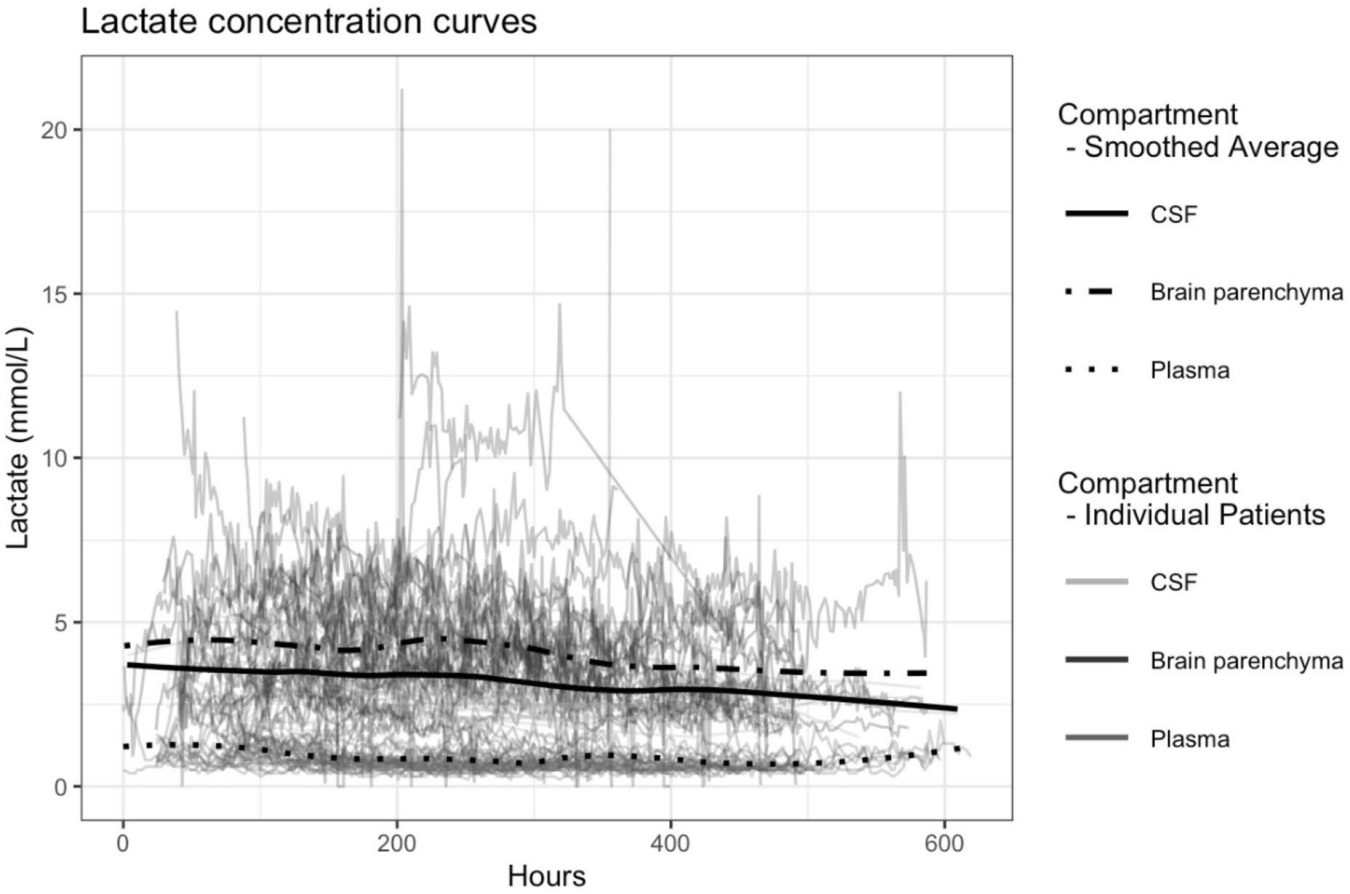
Lactate concentration curves across compartments. Time course of lactate concentrations (mmol/L) over h relative to the day of bleeding. Smoothed average trajectories are shown for plasma, cerebrospinal fluid (CSF), and brain parenchyma (microdialysis), together with individual patient curves. Supplementary material includes detailed trajectories for all patients and the corresponding fixed and random effects from the mixed-effects models

Glucose and lactate concentrations over time in all three compartments are shown in Figs. 1 and 2, respectively, starting on the first day of MMNM.

#### Glucose Concentrations

Higher glucose levels in both plasma and CSF were significantly associated with elevated cerebral glucose concentrations (Table 2). Specifically, a 1 mmol/L increase in either plasma or CSF glucose corresponded to an average rise of 0.2 mmol/L in cerebral glucose. A combined linear mixed model including all three compartments further confirmed the significance of both associations (Table 3).

**Table 2:** Association of glucose and lactate concentrations in univariable mixed models

Association	Metabolite	Regression slope (95% CI)	SD	*p*-Value	*R* (95% CI)
CSF as predictor for brain parenchyma	Glucose	**0.24 (0.13–0.35)**	**0.2**	** < 0.001**	**0.36 (0.23–0.48)**
	Lactate	**0.35 (0.01–0.68)**	**0.7**	**0.04**	**0.24 (0.09–0.37)**
Plasma as predictor for brain parenchyma	Glucose	**0.20 (0.17–0.23)**	**0.09**	** < 0.001**	**0.54 (0.52–0.56)**
	Lactate	− 0.05 (− 1.2 to 1.1)	3.5	0.93	**0.14 (0.11–0.18)**
Plasma as predictor for CSF	Glucose	**0.29 (0.19–0.39)**	**0.2**	** < 0.001**	**0.55 (0.43–0.66)**
	Lactate	**0.96 (0.55–1.4)**	**0.6**	** < 0.001**	**0.33 (0.18–0.47)**

Significant parameters are shown in bold. *95% CI* 95% confidence interval, *CSF* cerebrospinal fluid, *R* Bland–Altman correlation, *SD* standard deviation of the random slopes

**Table 3:** Associations of glucose and lactate concentrations in two multivariable mixed models regressing on brain parenchyma with both plasma and CSF concentrations as covariates

Metabolite	Association with brain parenchyma	Regression slope (95% CI)	*p*-Value
Glucose	Plasma	**0.12 (0.02–0.22)**	**0.01**
	CSF	**0.24 (0.03–0.46)**	**0.03**
Lactate	Plasma	0.33 (− 0.84 to 1.5)	0.58
	CSF	0.39 (0.008–0.76)	0.05

Cells refer to the estimates and *p*-values. Significant parameters are shown in bold. *95% CI* 95% confidence interval, *CSF* cerebrospinal fluid

A significant correlation was found between plasma and cerebral glucose levels (*r* = 0.54; Table 2), as well as between plasma and CSF glucose (*r* = 0.55; Table 2). In contrast, the correlation between CSF and cerebral glucose was weaker (*r* = 0.36; Table 2).

**Fig. 3:**
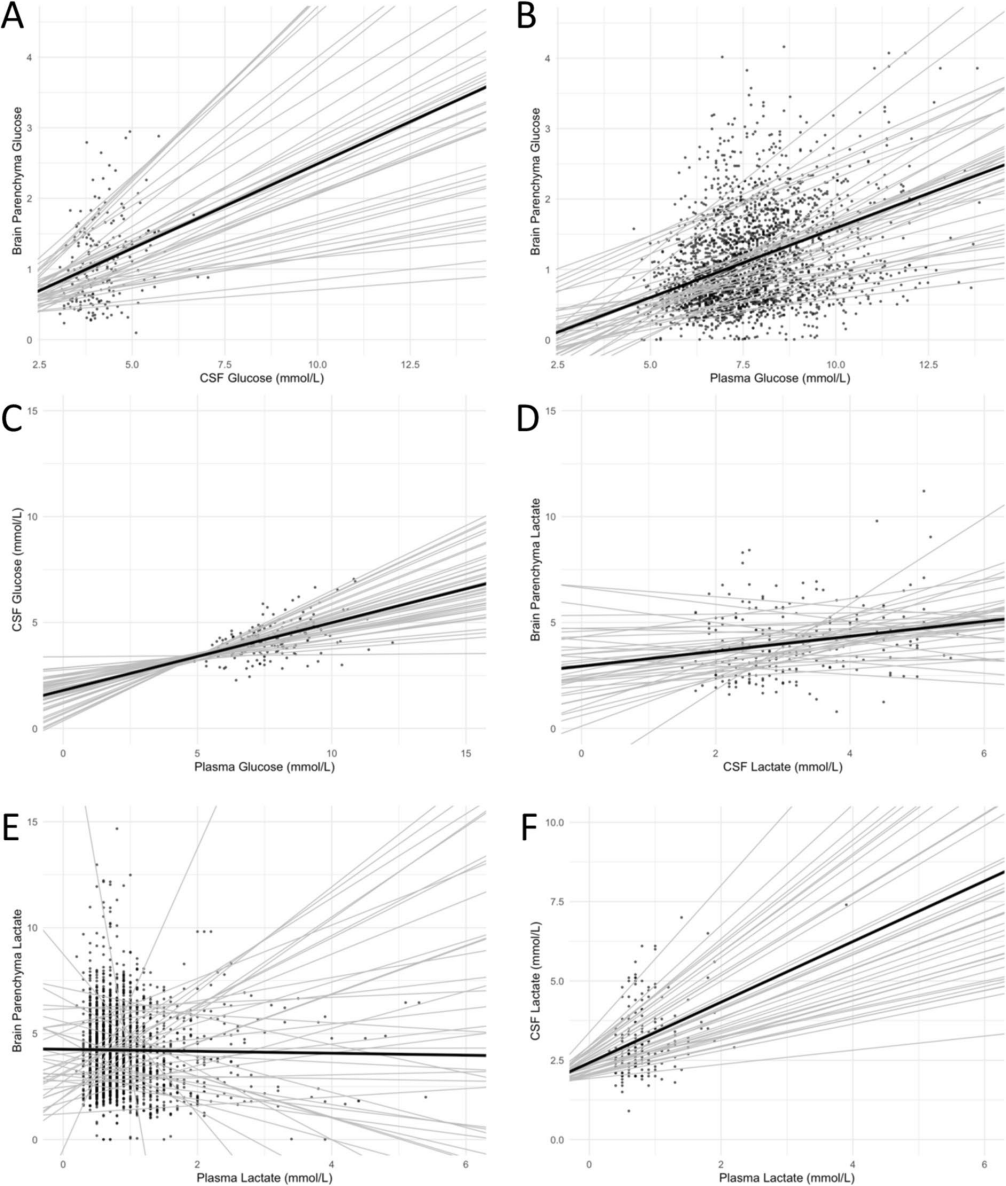
Patient-specific regression effects from the linear mixed-effects models for glucose and lactate across compartments. Panels A–C present the patient-specific effects for glucose for the compartmental comparisons cerebrospinal fluid (CSF) to brain parenchyma (**A**), plasma to brain parenchyma (**B**), and plasma to CSF (**C**). Panels D–F show the corresponding patient-specific effects for lactate for CSF to brain parenchyma (**D**), plasma to brain parenchyma (**E**), and plasma to CSF (**F**). Gray lines represent individual patient-specific effects including both fixed and random components, while the black line represents the population-level fixed effect, illustrating interindividual variability in baseline metabolite levels and in the strength and direction of the compartmental associations. Additional patient-level scatter plots with fitted regression lines are provided in the Supplementary Material

The interindividual variability in patient-specific effects for these glucose relationships is illustrated in Fig. 3A–C.

#### Lactate Concentrations

No significant association was found between plasma and cerebral lactate concentrations. In contrast, CSF lactate levels showed a significant association with cerebral lactate, as did plasma with CSF lactate concentrations (Table 2).

A 1 mmol/L increase in CSF lactate was significantly associated with an estimated 0.4 mmol/L rise in cerebral lactate.

A significant correlation was found between CSF and cerebral lactate levels (*r* = 0.24; Table 2), as well as between plasma and CSF lactate levels (*r* = 0.33; Table 2). In contrast, no significant correlation was observed between plasma and cerebral lactate concentrations.

The interindividual variability in patient-specific effects for lactate associations across compartments is shown in Fig. 3D–F.

#### Sensitivity Analysis

The results of the sensitivity analysis are provided in the supplementary material and generally support the previously presented findings. The only exception is the relationship between CSF and brain parenchyma lactate, which was no longer statistically significant despite showing a slightly stronger effect. This change is likely due to the smaller sample size and does not contradict the overall results.

#### Impact of Hypoglycemia, Normoglycemia, and Hyperglycemia

When analyzing the effects of hypoglycemia (plasma glucose < 3.33 mmol/L), normoglycemia, and hyperglycemia (plasma glucose > 10 mmol/L) on the associations between plasma and CSF concentrations with cerebral concentrations, neither normoglycemia nor hyperglycemia had a significant impact on these associations. The effect of hypoglycemia could not be assessed due to a low number of datapoints (tables reported in the supplementary material).

#### Impact of the Interval After aSAH

Following aSAH, the association between plasma and cerebral glucose concentrations decreased slightly over time (*p* = 0.008; Table 4). For lactate, the association between cerebral and plasma concentrations showed a small but significant positive shift over time (*p* < 0.001; Table 5).

**Table 4:** Multivariable analyses of main and interaction effects on cerebral glucose

Predictor	Estimate (95% CI)	*p*-Value
Plasma glucose	0.23 (0.19–0.26)	< 0.001
Days after aSAH	0.01 (− 0.003 to 0.03)	0.09
Plasma glucose × days after aSAH	** − 0.003 (− 0.005 to − 0.0008)**	**0.008**
Plasma glucose	0.18 (0.15–0.22)	< 0.001
Insulin yes	− 0.07 (− 0.32 to 0.18)	0.59
Plasma glucose × insulin yes	0.02 (− 0.01 to 0.05)	0.2
CSF glucose	0.21 (0.09–0.34)	0.002
Insulin yes	− 0.23 (− 0.91 to 0.46)	0.52
CSF glucose × insulin yes	0.05 (− 0.11 to 0.22)	0.51
Plasma glucose	0.17 (0.13–0.21)	< 0.001
pbtO_2_ < 15 mm Hg	− 0.42 (− 0.68 to − 0.14)	0.002
Plasma glucose × pbtO_2_ < 15 mm Hg	**0.04 (0.009–0.08)**	**0.01**
CSF glucose	0.23 (− 0.0005 to 0.46)	0.05
pbtO_2_ < 15 mm Hg	0.02 (− 1.06 to 1.07)	0.97
CSF glucose × pbtO_2_ < 15 mm Hg	0.02 (− 0.24 to 0.28)	0.88
Plasma glucose	0.21 (0.17–0.24)	< 0.001
LPR > 40	− 0.03 (− 0.21 to 0.15)	0.76
Plasma glucose × LPR > 40	− 0.02 (− 0.04 to 0.005)	0.13
CSF glucose	0.20 (0.08–0.33)	0.002
LPR > 40	− 0.47 (− 1.16 to 0.24)	0.19
CSF glucose × LPR > 40	0.10 (− 0.07 to 0.27)	0.26
CSF glucose	0.27 (0.15 to 0.40)	< 0.001
Cell count	0.0003 (− 0.0002 to 0.0008)	0.19
CSF glucose × cell count	− 0.0001 (− 0.0002 to 0.0000)	0.16

“Insulin yes” indicates peripheral insulin administration via a perfusor. “pbtO_2_ < 15 mm Hg” indicates the arithmetic mean for 90 min prior to the MD value. Significant interaction effects are shown in bold. *95% CI* 95% confidence interval, *CSF* cerebrospinal fluid, *LPR* lactate pyruvate ratio in cerebral microdialysate, *pbtO*_2_ brain tissue oxygen

**Table 5:** Multivariable analyses of main and interaction effects on cerebral lactate

Predictor	Estimate (95% CI)	*p*-Value
Plasma lactate	− 0.86 (− 2.0 to 0.23)	0.13
Days after aSAH	− 0.11 (− 0.13 to − 0.09)	< 0.001
Plasma lactate × days after aSAH	**0.06 (0.04–0.09)**	** < 0.001**
Plasma lactate	0.14 (− 0.95 to 1.22)	0.8
Insulin yes	0.92 (0.62–1.22)	< 0.001
Plasma lactate × insulin yes	** − 0.50 (− 0.77 to − 0.23)**	** < 0.001**
CSF lactate	0.25 (− 0.15 to 0.65)	0.22
Insulin yes	− 0.05 (− 1.73 to 1.69)	0.95
CSF lactate × insulin yes	0.16 (− 0.40 to 0.70)	0.55
Plasma Lactate	− 0.22 (− 1.27 to 0.82)	0.68
pbtO_2_ < 15 mmHg	− 0.12 (− 0.46 to 0.22)	0.48
Plasma lactate × pbtO_2_ < 15 mm Hg	0.21 (− 0.11 to 0.54)	0.19
CSF lactate	0.36 (− 0.20 to 0.90)	0.22
pbtO_2_ < 15 mmHg	− 0.48 (− 2.5 to 1.6)	0.62
CSF lactate × pbtO_2_ < 15 mm Hg	− 0.00 (− 0.61 to 0.59)	1.0
Plasma lactate	− 0.07 (− 1.23 to 1.08)	0.91
LPR > 40	0.02 (− 0.16 to 0.20)	0.82
Plasma lactate × LPR > 40	0.99 (− 0.08 to 0.28)	0.29
CSF lactate	0.26 (− 0.08 to 0.60)	0.13
LPR > 40	− 1.09 (− 2.41 to 0.26)	0.11
CSF lactate × LPR > 40	**0.43 (0.02–0.84)**	**0.04**
CSF lactate	0.41 (0.01–0.80)	0.05
Cell count	− 0.0001 (− 0.001 to 0.001)	0.81
CSF lactate × cell count	0 (− 0.0002 to 0.0003)	0.91

“Insulin yes” indicates peripheral insulin administration via a perfusor. “pbtO_2_ < 15 mm Hg” indicates the arithmetic mean for 90 min prior to the MD value. Significant interaction effects are shown in bold. *95% CI* 95% confidence interval, *CSF* cerebrospinal fluid, *LPR* lactate pyruvate ratio in cerebral microdialysate, *pbtO*_2_ brain tissue oxygen

#### Impact of Insulin Administration

When analyzing the effect of insulin administration on the associations between plasma, CSF, and cerebral concentrations, no significant effect on the glucose associations was found (*p* > 0.05; Table 4). In contrast, a significant negative effect was observed on the association between cerebral and plasma lactate concentrations (*p* < 0.001; Table 5).

#### Impact of Hypoxia

Analysis of the effect of pbtO_2_ on plasma and cerebral concentrations showed a significant impact on the association between cerebral and plasma glucose. For pbtO_2_ levels less than 15 mm Hg, a significant increase in the association between cerebral and plasma glucose was observed (*p* = 0.01; Table 4). There was a significant negative impact of hypoxia on cerebral glucose with an estimate of − 0.4 (*p* = 0.002; Table 4). In contrast, there was no significant effect of hypoxia on the associations of lactate concentrations (*p* > 0.05; Table 5).

#### Impact of Cerebral Metabolic Crisis

Analyzing the effect of cerebral metabolic crisis [defined as lactate–pyruvate ratio (LPR) > 40] on the associations between plasma, CSF, and cerebral concentrations, a significant impact was found on the relationship between cerebral and CSF lactate concentrations, with a positive effect of 0.4 (*p* = 0.04; Table 5).

#### Impact of CSF Cell Count

CSF cell count did not significantly influence the associations between plasma, CSF, and cerebral glucose and lactate concentrations (Tables 4 and 5).

## Discussion

In this study involving 39 patients with severe aSAH, we found that cerebral glucose concentrations were significantly associated with both plasma and CSF glucose levels, with a stronger correlation observed for plasma glucose. In contrast, cerebral lactate levels were primarily associated with CSF lactate, while no significant relationship was observed with plasma lactate.

Under physiological conditions, glucose transport across the blood–brain barrier (BBB) is primarily mediated by the glucose transporter 1 (GLUT1) [18] and follows reversible Michaelis–Menten kinetics [9, 10]. To a lesser extent, other GLUT isoforms and sodium-glucose cotransporters (SGLTs) contribute to the transport of glucose across the BBB [19]. Following aSAH, the integrity of the BBB is typically compromised [20, 21]. Similarly to the BBB, GLUT1 is the principal glucose transporter in the choroid plexus, enabling facilitated diffusion across the blood–CSF barrier [22]. It operates alongside SGLTs, which mediate active, sodium-coupled glucose uptake [22]. Between the brain parenchyma and CSF, glucose exchange is largely unimpeded due to the absence of tight junctions between the ependymal cells lining the ventricles [23]. Therefore, glucose exchange between these compartments can be assumed to be at least partly driven by passive diffusion along concentration gradients; however, comprehensive research on the mechanisms of these intercompartmental dynamics is lacking.

Our findings indicate that significant intercompartmental associations for glucose persist across all three compartments, although the standard deviations of the random slopes combined with the plots of individual patient concentrations curves show that considerable interindividual variation exists. Our findings are generally in alignment with the results of previous studies for the coupling of plasma–MD and plasma–CSF. The correlation of glucose between plasma and brain parenchyma was studied by Zetterling et al. [13] following aSAH, with a weak but significant correlation being reported. The results of other studies investigating this intercompartmental relationship in acutely brain injured patients are generally consistent with this finding [24–28]. A recent study by Fenger et al. [29] investigated the relationship between systemic and cerebral glucose in patients with traumatic brain injury (TBI) and aSAH using a methodology similar to ours. Their linear regression model demonstrated a 0.16 mmol/L increase in MD glucose for every 1 mmol/L increase in systemic glucose in patients with aSAH, indicating a relationship very similar to our findings. A previous study in patients with aSAH reported a significant but weaker correlation between blood and CSF glucose than in our present results, which may be attributable to differences in methodology and patient population [14]. Following TBI, a similar weak correlation was observed [30]. In a bicentric retrospective analysis of patients with acute brain injury, which is the first study to date that investigated the coupling of CSF and MD glucose concentrations, no significant correlation was found between these two compartments [15]. In contrast, our current analysis revealed a modest association between CSF and MD glucose in our patient population, which appears to contradict the earlier findings.

While prior research focusing on intercompartmental relationships following aSAH analyzed glucose concentrations between two compartments in isolation [13–15], our study extends these findings by simultaneously examining the interrelationship between plasma, CSF, and brain parenchyma. Elevated glucose concentrations in plasma and CSF correlate with higher cerebral glucose levels, indicating that systemic glucose management is important for modulating cerebral availability after aSAH. Although glucose levels can fluctuate considerably during the acute phase of aSAH, our data suggest that hyperglycemia does not markedly alter the fundamental relationship between systemic and cerebral glucose.

The modest strength of intercompartmental correlations observed for glucose likely reflects the complex interplay of systemic, vascular, and cellular factors influencing cerebral metabolism after aSAH. Cerebral glucose availability depends not only on arterial substrate levels, but also on cerebral blood flow, perfusion pressure, and the integrity of the blood–brain barrier, all of which fluctuate substantially during the acute phase and throughout neurointensive care. In addition, regional differences in mitochondrial function, aerobic–anaerobic balance, and metabolic demand contribute to spatial heterogeneity of cerebral substrate utilization. Clinical conditions such as delayed cerebral ischemia, sedation, and systemic glucose management further modulate these dynamics. While CSF reflects a more global metabolic environment, MD measures focal extracellular concentrations from a small tissue volume, which may not represent the overall cerebral metabolic state, particularly in the context of evolving delayed cerebral ischemia and heterogeneous perfusion [31].

A similar concept applies to lactate, where local production and regional metabolic stress are likely to outweigh systemic influences, further limiting intercompartmental correlations. In line with this, associations for lactate concentrations were less consistent. Significant associations were found between CSF and cerebral concentrations, as well as between plasma and CSF; however, these correlations were generally weaker. Previous studies on lactate dynamics in TBI and the coupling of plasma–CSF have yielded inconclusive results. While some investigations found no significant correlation between plasma and CSF lactate concentrations [32, 33], a more recent analysis by Lozano et al. [30] reported a moderate and significant correlation (*R*^2^ = 0.32, *p* < 0.001). Following aSAH, a weak but significant correlation has been reported [14]. Regarding the relationship between CSF and brain parenchyma lactate concentrations, the aforementioned study by Bellettieri et al. [15] found no significant correlation. Our differing results highlight the complexity of lactate dynamics and underscore the need for further research to clarify these compartmental relationships.

Lactate primarily reflects local tissue metabolism and is produced under ischemic conditions or under nonischemic conditions from activated glycolysis [34]. The absence of a significant association between plasma and cerebral lactate concentrations aligns with the localized nature of lactate production. CSF lactate concentrations are more directly reflective of cerebral metabolic processes than plasma lactate, which is often influenced by systemic factors such as peripheral tissue metabolism, hepatic function, and global perfusion status [35]. Consequently, CSF lactate may demonstrate a closer, though still modest, association with cerebral extracellular lactate levels. CSF lactate concentrations may potentially reflect changes in brain tissue lactate with a delay and on a more global scale compared with MD measurements. However, considering the findings of Bellettieri et al. [15] and the limited amount of datapoints in our analysis, these results should be interpreted with caution.

The astrocyte–neuron lactate shuttle (ANLS) provides an important mechanistic framework for understanding lactate dynamics within the injured brain. According to this concept, astrocytes metabolize glucose to lactate through glycolysis and release lactate into the extracellular space, where it serves as an oxidative substrate for neurons under both physiological and stress conditions [36, 37]. Following aSAH, activation of astrocytic glycolysis and mitochondrial dysfunction may enhance local lactate production and alter its utilization, resulting in elevated microdialysate lactate levels that are not necessarily mirrored in plasma or CSF. This focal production, combined with heterogeneous perfusion and variable BBB permeability, likely attenuates correlations between systemic, CSF, and cerebral lactate concentrations.

While the ANLS explains local lactate dynamics at the cellular level, the distribution of lactate between systemic, CSF, and cerebral compartments further depends on transport mechanisms across barrier structures. Lactate transport across the blood–CSF barrier and the BBB is facilitated primarily by monocarboxylate transporters (MCTs) expressed in the epithelial cells of the choroid plexus and the endothelial cells of cerebral microvessels, respectively [38]. Similar to glucose and other small molecules, passive diffusion, facilitated by the absence of tight junctions between ependymal cells, should aid lactate exchange between brain parenchyma and CSF [23]. Additionally, the glymphatic system facilitates the clearance and transport of metabolic waste products, including lactate, within the brain [39]. This system may play a significant role in modulating the intercompartmental dynamics between brain tissue and CSF, potentially impacting metabolite distribution and clearance.

Plasma lactate concentrations reflect systemic metabolic activity, whereas CSF, as an intermediate compartment between the systemic circulation and brain tissue, may better capture localized cerebral metabolic changes [23]. The relationship between CSF and cerebral lactate levels suggests that lactate accumulation in CSF could serve as an indicator of cerebral metabolic disturbance, potentially reflecting impaired oxygenation or energy metabolism after aSAH.

After establishing the relationships of glucose and lactate across compartments, we investigated factors that might modulate these associations. Specifically, we examined how different cerebral conditions affected parenchymal glucose and lactate concentrations and their coupling with plasma and CSF levels.

Interestingly, the presence of insulin was associated with a markedly reduced association between plasma and cerebral lactate in our population, suggesting that insulin may modulate cerebral lactate metabolism or alter lactate dynamics between the compartments. Conversely, glucose associations were not significantly impacted by insulin administration. While Taccone et al. [14] found no influence of insulin therapy on the relationship between CSF glucose and blood glucose, there have been some authors reporting data suggesting a possible impact on the relationship between cerebral and blood glucose levels [11, 13, 40].

Critically low brain tissue oxygenation significantly modulated the association between plasma and brain parenchymal glucose levels. The regression of brain parenchyma glucose on the interaction between plasma glucose and hypoxia revealed a positive effect, indicating a stronger association between plasma and cerebral glucose concentrations under hypoxic conditions. This might suggest that during hypoxia, the brain’s reliance on systemic glucose increases, likely due to compromised local glucose availability. In general glucose concentrations in brain tissue, changes in perfusion and pbtO_2_ have been closely followed [41], and it has been reported that under induced hyper- and hypoglycemia in normal subjects, glucose levels in brain tissue changed, with blood glucose lagging about 30 min behind, indicating a strengthening of the intercompartmental coupling under these conditions [41, 42].

In contrast, the relationship between plasma and cerebral lactate was not significantly affected by pbtO_2_. This is consistent with lactate being predominantly produced endogenously via anaerobic glycolysis, particularly under hypoxic conditions [5, 34]. It is plausible that the augmented local production of lactate outweighs the impact of plasma lactate levels, reducing the intercompartmental association. Our findings are generally consistent with the notion that extracellular lactate in the brain primarily reflects local metabolic activity rather than systemic transport, especially during hypoxia or metabolic stress [5, 34].

While hypoxia strengthened the association between plasma and cerebral glucose, the presence of a cerebral metabolic crisis—defined by LPR > 40 in microdialysate [6]—did not significantly modify this relationship. This indicates that the association between systemic and parenchymal glucose concentrations remains largely unaffected during episodes of metabolic crisis. Elevated LPR, however, can arise from diverse mechanisms, including impaired oxygen or substrate delivery due to hypoperfusion, or intrinsic mitochondrial dysfunction where oxygen and glucose delivery are preserved but oxidative metabolism is compromised [6, 43, 44]. This complexity complicates the interpretation of glucose dynamics during metabolic crises.

Conversely, our findings revealed a positive effect of metabolic crisis on the association between CSF and cerebral lactate, suggesting a stronger link between these compartments under metabolic stress. This may reflect increased lactate production both locally and more globally within the brain tissue, contributing to elevated CSF lactate levels. As lactate accumulates via anaerobic metabolism, the metabolic profile across compartments becomes more homogeneous, thereby strengthening their correlation. Thus, CSF lactate may serve as a global marker of cerebral metabolic distress while still reflecting local metabolic dysfunction.

CSF cell count, typically used as an indicator of infection or inflammation [45], does not appear to affect the relationships between glucose or lactate concentrations across compartments. This suggests that metabolic alterations in the brain and plasma occur independently of inflammatory processes reflected by CSF cell count. A study by ﻿Schlenk et al﻿. [46] reported that bacterial meningitis, characterized by elevated CSF cell count, was associated with reduced cerebral glucose, while CSF glucose levels remained unchanged, indicating that inflammation may impair the reliability of CSF glucose as a surrogate for cerebral metabolism.

This study is limited by its small sample size, which reduces statistical power and generalizability. Its retrospective design further constrained analysis to routinely collected clinical data, limiting usable datapoints, especially in models combining plasma, CSF, and brain parenchyma variables. MD was mainly performed in patients with poor-grade aSAH, introducing selection bias toward critically ill individuals and limiting the representativeness of the cohort relative to the broader aSAH population. Additionally, MD reflects only local tissue metabolism near the probe, limiting extrapolation to global brain function. Because many catheters were located ipsilateral to DCI-related injury, local metabolic alterations at the probe site may have influenced the measured MD parameters and thereby limit interpretation of the intercompartmental relationships.

## Conclusions

In severe aSAH, glucose and lactate dynamics across plasma, CSF, and brain parenchyma are interrelated but vary by metabolite and compartment. Cerebral glucose levels correlated with both plasma and CSF glucose, while cerebral lactate was more closely linked to CSF. These associations were influenced by clinical factors such as brain oxygenation, insulin, and metabolic crisis. The marked interindividual variability and dependence on clinical conditions indicate that blood and CSF provide only limited insight into cerebral metabolic changes, underscoring the complementary value of MD for individualized, brain-targeted monitoring.

## Supplementary Information

Below is the link to the electronic supplementary material.

Supplementary file1 (PDF 835 KB)

## Abbreviations

ACA: Anterior cerebral artery
AcomA: Anterior communicating artery
ANLS: Astrocyte-neuron lactate shuttle
aSAH: Aneurysmal subarachnoid hemorrhage
BA: Basilar artery
BBB: Blood–brain barrier
CI: Confidence interval
CSF: Cerebrospinal fluid
CPP: Cerebral perfusion pressure
EVD: External ventricular drainage
GLUT: Glucose transporter protein
ICA: Internal carotid artery
ICP: Intracranial pressure
IQR: Interquartile range
LPR: Lactate–pyruvate ratio
MCA: Middle cerebral artery
MCT: Monocarboxylate transporter
MD: Cerebral microdialysis
MMNM: Multimodality neuromonitoring
mRS: Modified Rankin scale
pbtO_2_: Brain tissue oxygen
P1: P1 segment of posterior cerebral artery
PcomA: Posterior communicating artery
PICA: Posterior inferior cerebellar artery
SCA: Superior cerebellar artery
Sd: Standard deviation
SGLT: Sodium-glucose cotransporter
TBI: Traumatic brain injury
