# Supplementary material for: Glucose and Lactate Concentrations in Plasma, Cerebrospinal Fluid, and Brain Parenchyma Following Aneurysmal Subarachnoid Hemorrhage: A Cross-compartmental Correlation Study
Source: Neurocrit Care. 2026 Feb 4;44(3):998–1010. doi: 10.1007/s12028-025-02442-7 (PMC13249765; doi:10.1007/s12028-025-02442-7)
Supplement: Supplementary file 1 — Supplementary file1 (PDF 835 KB) [file 12028_2025_2442_MOESM1_ESM.pdf]

# Glucose and Lactate Concentrations in Plasma, Cerebrospinal Fluid and Brain Parenchyma Following Aneurysmal Subarachnoid Hemorrhage: A Cross-Compartmental Correlation Study

## Supplemental material

Niko Schmaling[1], Miriam M Moser MD[1], Lena Weyer [2], Robin Ristl, PhD[2], Walter Plöchl, MD[3], Andrea Reinprecht, MD[1], Johannes Herta, MD PhD[1], Karl Rössler, MD[1], Arthur Hosmann, MD PhD[1]

[1]Department of Neurosurgery, Medical University of Vienna, Vienna, Austria

[2]Institute for Medical Statistics, Center for Medical Statistics, Informatics and Intelligent Systems, Medical University of Vienna, Austria

[3]Department of Anesthesia, General Intensive Care Medicine and Pain Management, Medical University of Vienna, Vienna, Austria

Corresponding Author: Arthur Hosmann, MD PhD

Department of Neurosurgery, Medical University of Vienna, Währinger-Gürtel 18-20, A-1090 Vienna, Austria, Phone: -43-1-40400-25650, Fax: -43-1-40400-45660, E-mail: arthur.hosmann@meduniwien.ac.at

Table of Contents

1. Secondary Hypothesis- Exploration of the Relationship Between the Concentrations……………………………………3

2. Results of Sensitivity Analysis……………………………3

3. Trajectories of the Glucose Concentration in Different Compartments for Individual Patients………………………5

4. Trajectories of the Lactate Concentration in Different Compartments for Individual Patients……………………45

5. Scatter Plots……………………………………………………85

5.1 CSF Glucose vs. Brain Parenchyma Glucose………85
5.2 CSF Lactate vs. Brain Parenchyma Lactate…………96
5.3 Plasma Glucose vs. Brain Parenchyma Glucose…107
5.4 Plasma Lactate vs. Brain Parenchyma Lactate….118
5.5 Plasma Glucose vs. CSF Glucose……………………129
5.6 Plasma Lactate vs. CSF Lactate………………………140

Sections 3, 4, and 5 present trajectories as well as scatterplots for individual patients. Within each (sub-)chapter, the first plot corresponds to the data of a single patient, the second plot to a different patient, and so forth.

# 1.Secondary hypothesis- exploration of the relationship between the concentrations

Only two hypoglycemic values occurred in the data and they could not be assigned to any brain parenchyma glucose value, so no hypoglycemia could be evaluated in the model. Therefore this group is missing.

Table 1B: Fixed effect, standard deviation of the random effects and residuals estimates derived by a mixed model regressing brain parenchyma glucose on plasma glucose with a slope break ((180/18.02) mmol/L) in plasma glucose.

| | **Estimates** | **CI 2.5%** | **CI 97.5%** | **p-value** |
|---|---|---|---|---|
| **1. Fixed Effects** | | | | |
| Intercept | -0.3841 | -0.6014 | -0.1689 | 0.0011 |
| Glucose Serum | 0.1972 | 0.1647 | 0.2298 | <0.001 |
| Serum_Hyper | 0.0003 | -0.0501 | 0.0505 | 0.9898 |
| **2. Random Effects and Residuals** | | | | |
| sd residuals | 0.3612 | 0.3515 | 0.3713 | |
| sd intercept | 0.5808 | 0.4221 | 0.7736 | |
| sd Glucose Serum | 0.0916 | 0.0695 | 0.1189 | |

Table 2B: Fixed effect, standard deviation of the random effects and residuals estimates derived by a mixed model regressing brain parenchyma lactate on plasma lactate with a slope break ((180/18.02) mmol/L) in plasma glucose.

| | **Estimates** | **CI 2.5%** | **CI 97.5%** | **p-value** |
|---|---|---|---|---|
| **1. Fixed Effects** | | | | |
| Intercept | 4.2677 | 3.1627 | 5.3798 | <0.001 |
| Laktat Serum | -0.0653 | -1.2204 | 1.0788 | 0.9109 |
| Serum_Hyper | 0.0750 | -0.0644 | 0.2142 | 0.2914 |
| **2. Random Effects and Residuals** | | | | |
| sd residuals | 1.0731 | 1.0440 | 1.1031 | |
| sd intercept | 3.4381 | 2.7061 | 4.3621 | |
| sd Laktat Serum | 3.5267 | 2.7430 | 4.5079 | |

## 2. Results of sensitivity analysis

To assess the sensitivity of the models to outliers that were determined with the help of box plots, outlying glucose (plasma glucose ≥ 11.5 mmol/l, CSF glucose ≥ 6 mmol/l ) and lactate (plasma lactate ≥ 2mmol/l, CSF lactate ≥ 6 mmol/l ) data were removed and the new data set was used to estimate the regression parameters.

Table 3B: Fixed effect, standard deviation and correlation estimates derived by a univariable mixed model
regressing brain parenchyma glucose on CSF glucose excluding outliers (outlier: CSF glucose ≥ 6).

| | Estimates | CI 2.5% | CI 97.5% | p-value |
|---|---|---|---|---|
| **1. Fixed Effects** | | | | |
| Intercept | 0.0966 | -0.3285 | 0.5428 | 0.6599 |
| Glucose CSF | 0.2390 | 0.1209 | 0.3522 | <0.001 |
| **2. Random Effects and Residuals** | | | | |
| sd residuals | 0.3716 | 0.3335 | 0.4169 | |
| sd intercept | 0.5696 | 0 | 1.1458 | |
| sd Glucose CSF | 0.1938 | 0.0293 | 0.3344 | |

Table 4B: Fixed effect, standard deviation and correlation estimates derived by a univariable mixed model
regressing brain parenchyma glucose on plasma glucose excluding outliers (outlier: plasma glucose ≥ 11.5).

| | Estimates | CI 2.5% | CI 97.5% | p-value |
|---|---|---|---|---|
| **1. Fixed Effects** | | | | |
| Intercept | -0.4173 | -0.6427 | -0.1950 | <0.001 |
| Glucose Plasma | 0.2018 | 0.1668 | 0.2370 | <0.001 |
| **2. Random Effects and Residuals** | | | | |
| sd residuals | 0.3568 | 0.3471 | 0.3669 | |
| sd intercept | 0.6145 | 0.4479 | 0.8169 | |
| sd Glucose Plasma | 0.1011 | 0.0767 | 0.1312 | |

Table 5B: Fixed effect, standard deviation and correlation estimates derived by a univariable mixed model regressing brain parenchyma lactate on CSF lactate excluding outliers (outlier: CSF lactate ≥ 6).

| | Estimates | CI 2.5% | CI 97.5% | p-value |
|---|---|---|---|---|
| **1. Fixed Effects** | | | | |
| Intercept | 2.7488 | 1.4226 | 4.0550 | <0.001 |
| Lactate CSF | 0.3982 | -0.0001 | 0.7877 | 0.0549 |
| **2. Random Effects and Residuals** | | | | |
| sd residuals | 1.1120 | 0.9937 | 1.2579 | |
| sd intercept | 3.1114 | 1.4544 | 4.6669 | |
| sd Lactate CSF | 0.8894 | 0.4265 | 1.3213 | |

Table 6B: Fixed effect, standard deviation and correlation estimates derived by a univariable mixed model regressing brain parenchyma glucose on plasma lactate excluding outliers (outlier: plasma lactate ≥ 2).

| | Estimates | CI 2.5% | CI 97.5% | p-value |
|---|---|---|---|---|
| **1. Fixed Effects** | | | | |
| Intercept | 4.1875 | 3.0602 | 5.3214 | <0.001 |
| Lactate Plasma | 0.0236 | -1.1502 | 1.1859 | 0.9682 |
| **2. Random Effects and Residuals** | | | | |
| sd residuals | 1.0700 | 1.0406 | 1.1009 | |
| sd intercept | 3.5012 | 2.7571 | 4.4411 | |
| sd Lactate Plasma | 3.5799 | 2.7890 | 4.5724 | |

The sensitivity analyses support the main results. Only the connection between CSF and brain parenchyma lactate is slightly not significant anymore although the effect is slightly higher. This is due to the smaller sample size and still supports the results.

## 3. Trajectories of the glucose concentration in different compartments for individual patients

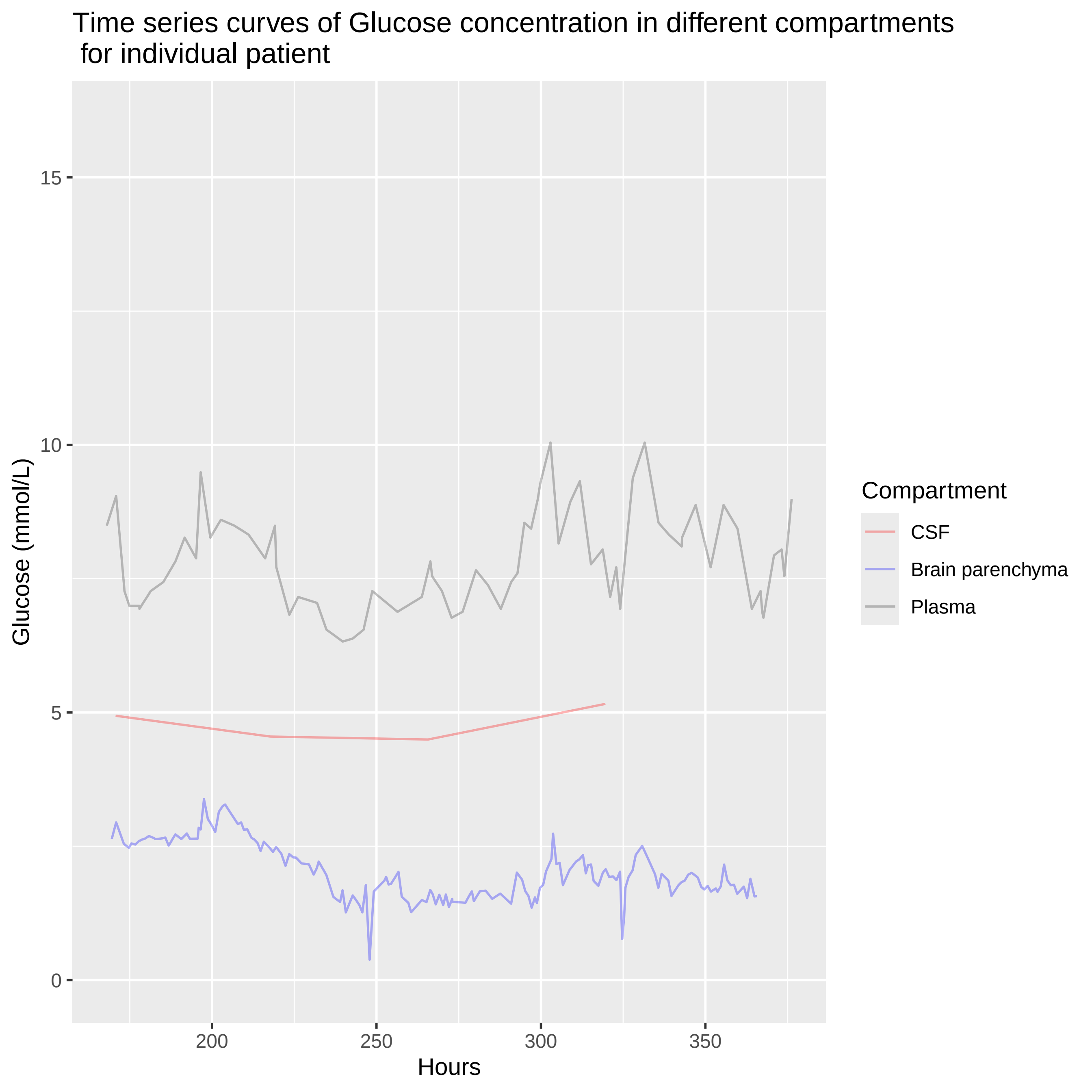
Time series curves of Glucose concentration in different compartments
for individual patient
Glucose (mmol/L)
15
10
5
0
200
250
300
350
Hours
Compartment
CSF
Brain parenchyma
Plasma

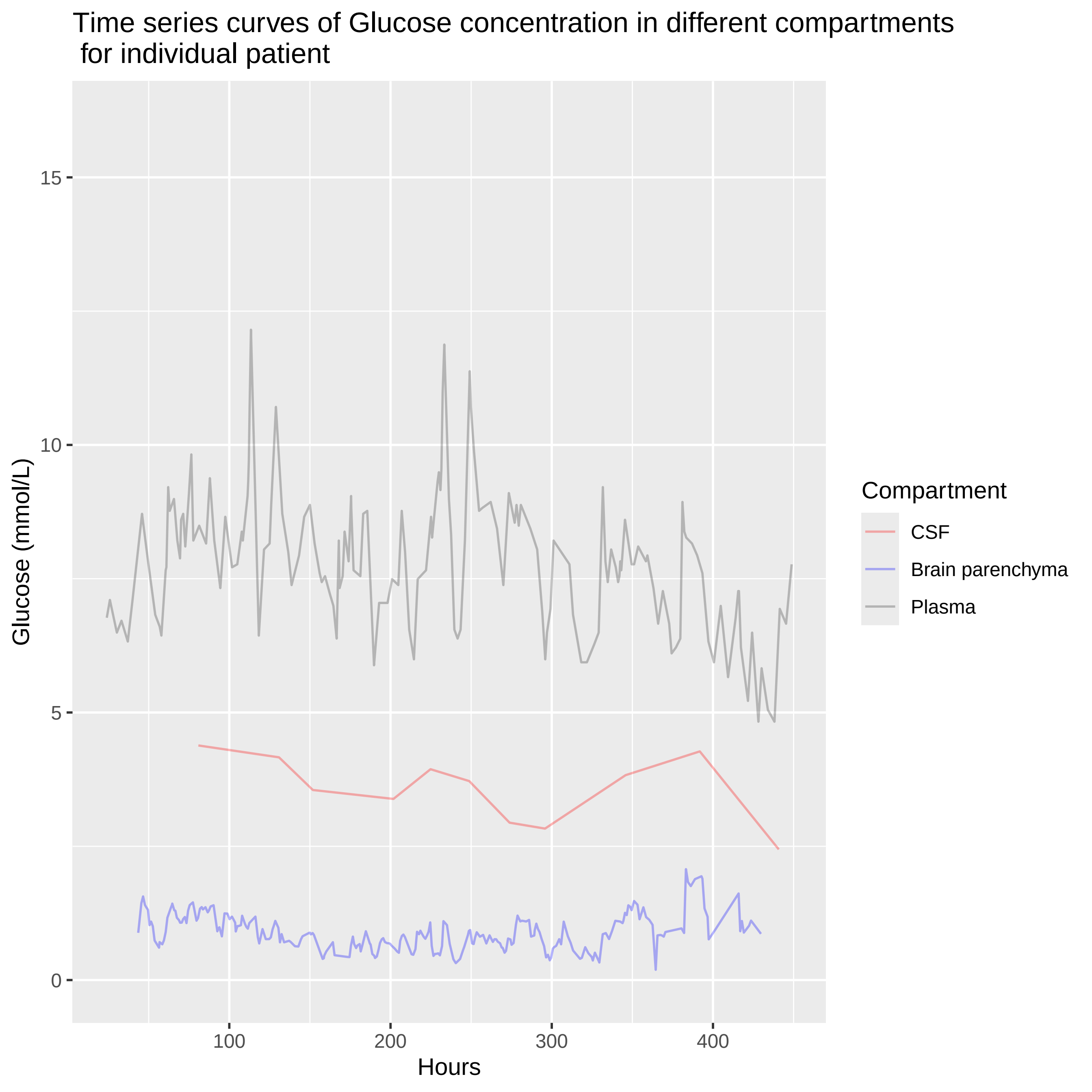

Time series curves of Glucose concentration in different compartments
for individual patient
Glucose (mmol/L)
Hours
0
5
10
15
100
200
300
400
Compartment
CSF
Brain parenchyma
Plasma

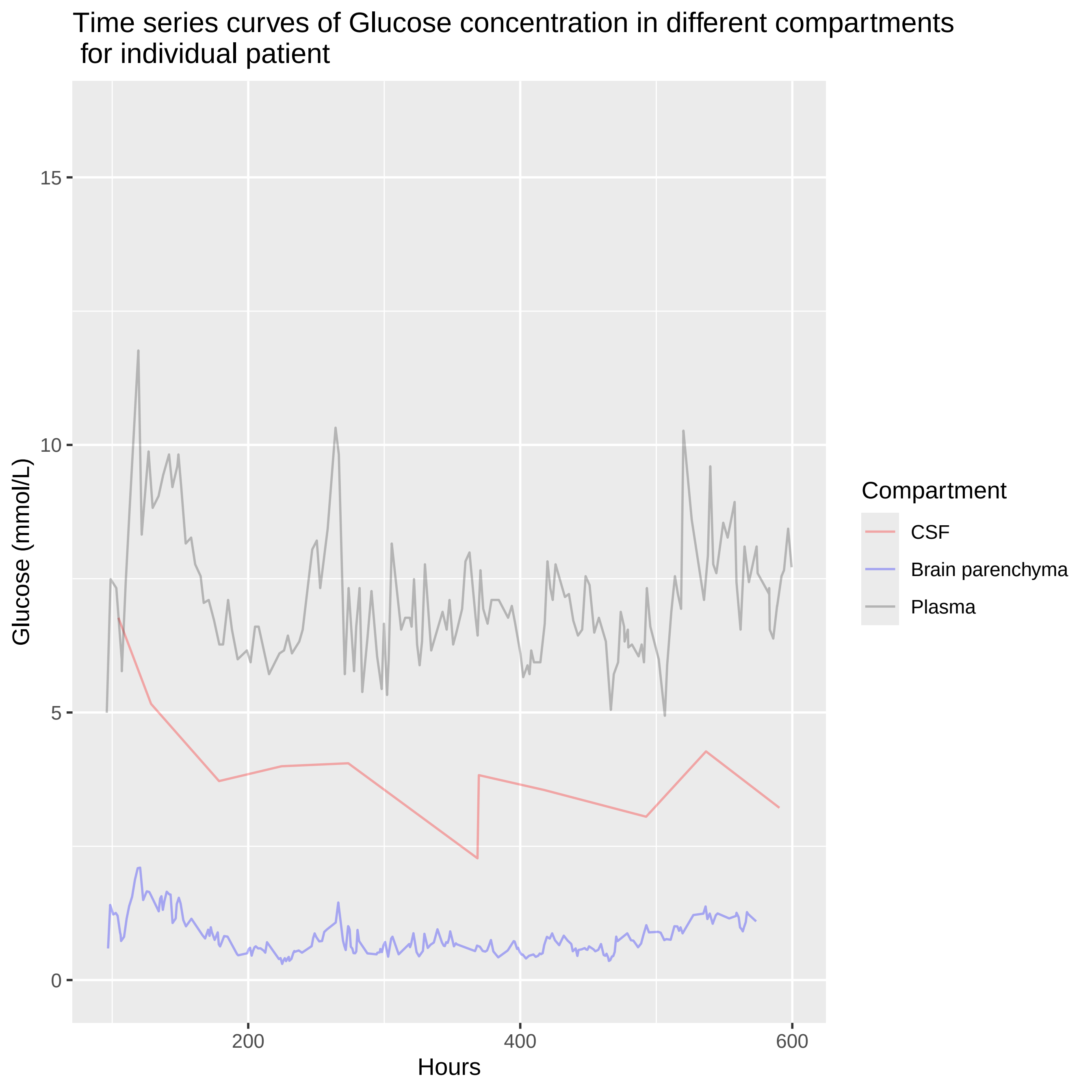

Time series curves of Glucose concentration in different compartments
for individual patient
Glucose (mmol/L)
15
10
5
0
200
400
600
Hours
Compartment
CSF
Brain parenchyma
Plasma

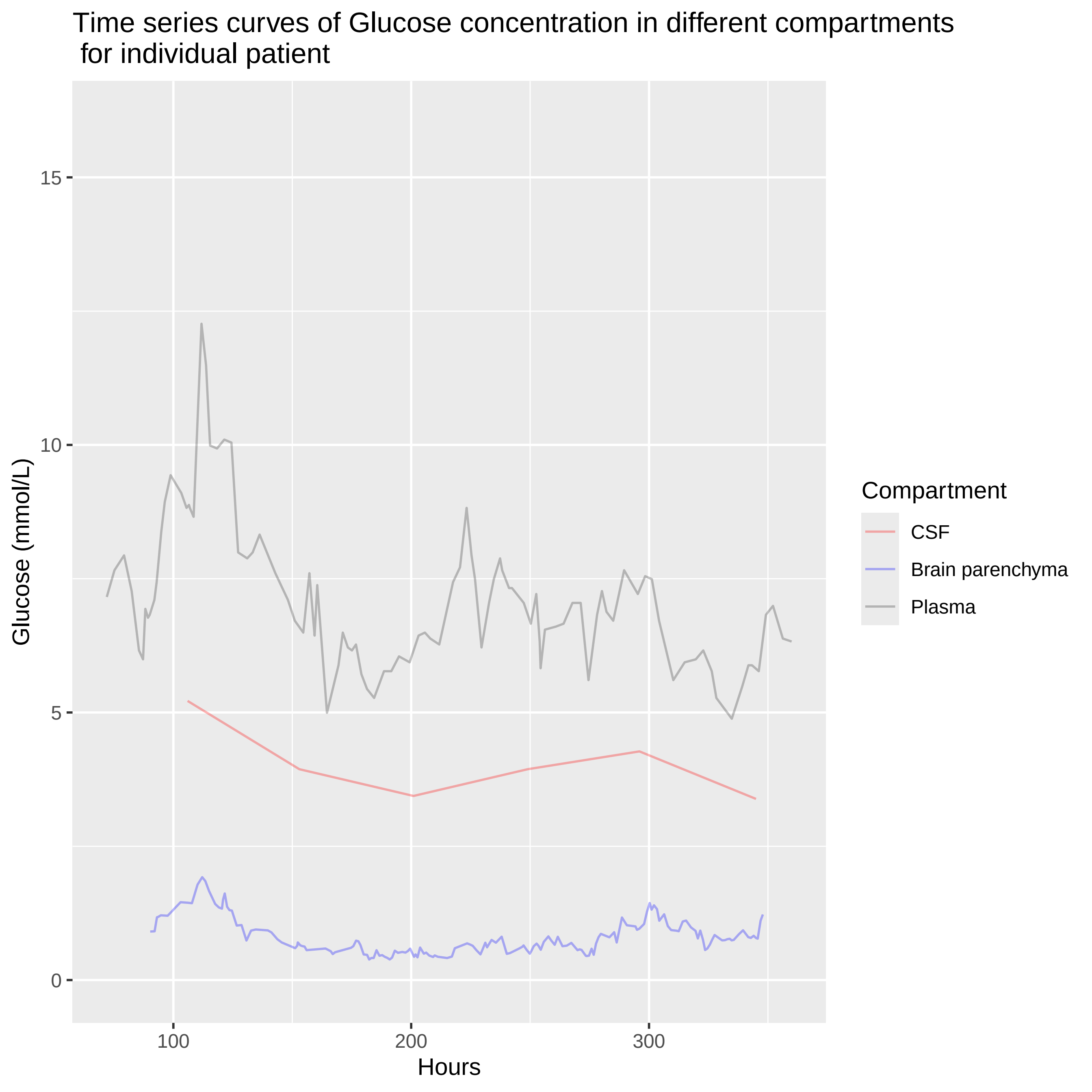

Time series curves of Glucose concentration in different compartments
for individual patient
15
10
5
0
Glucose (mmol/L)
100
200
300
Hours
Compartment
CSF
Brain parenchyma
Plasma

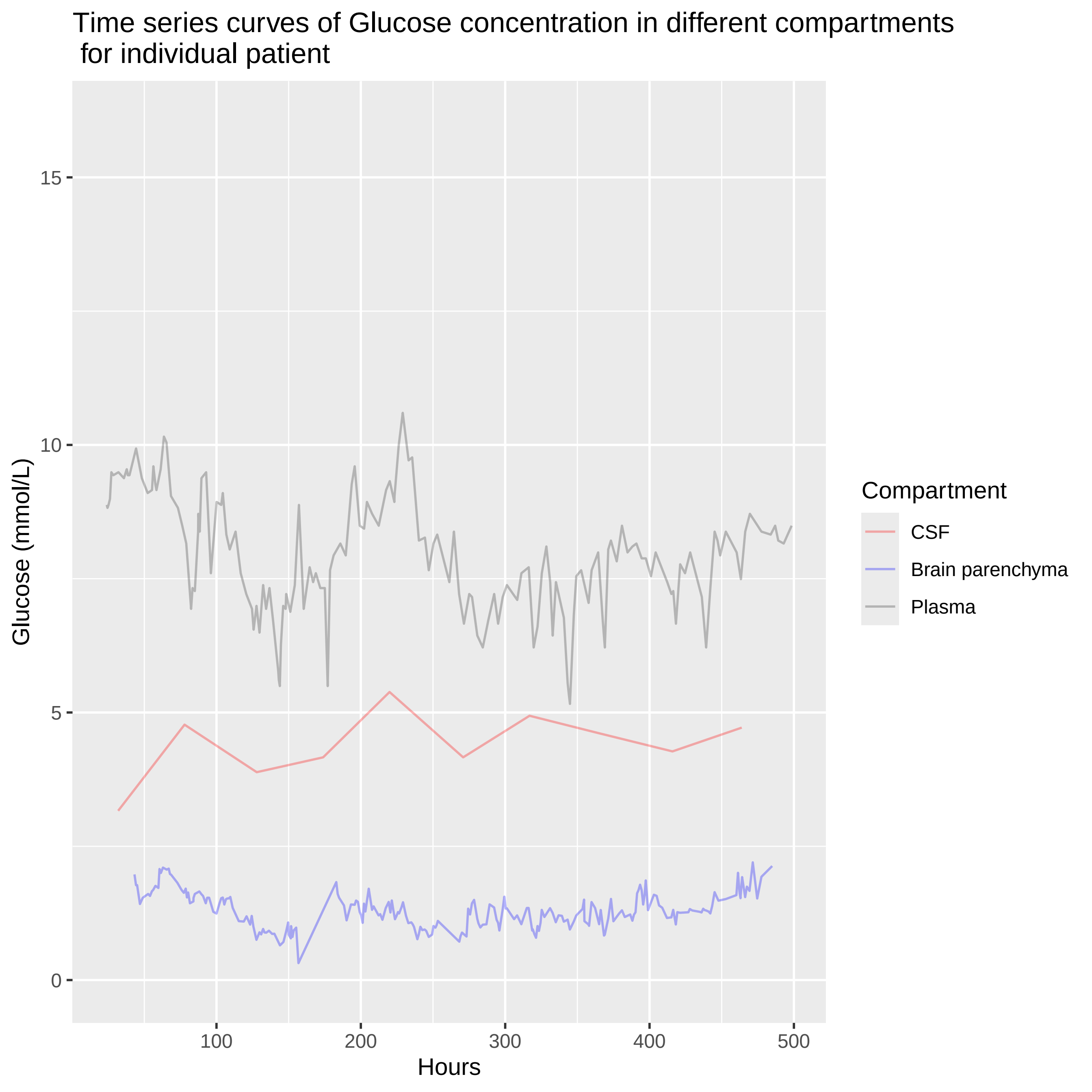

Time series curves of Glucose concentration in different compartments
for individual patient
Glucose (mmol/L)
15
10
5
0
100
200
300
400
500
Hours
Compartment
CSF
Brain parenchyma
Plasma

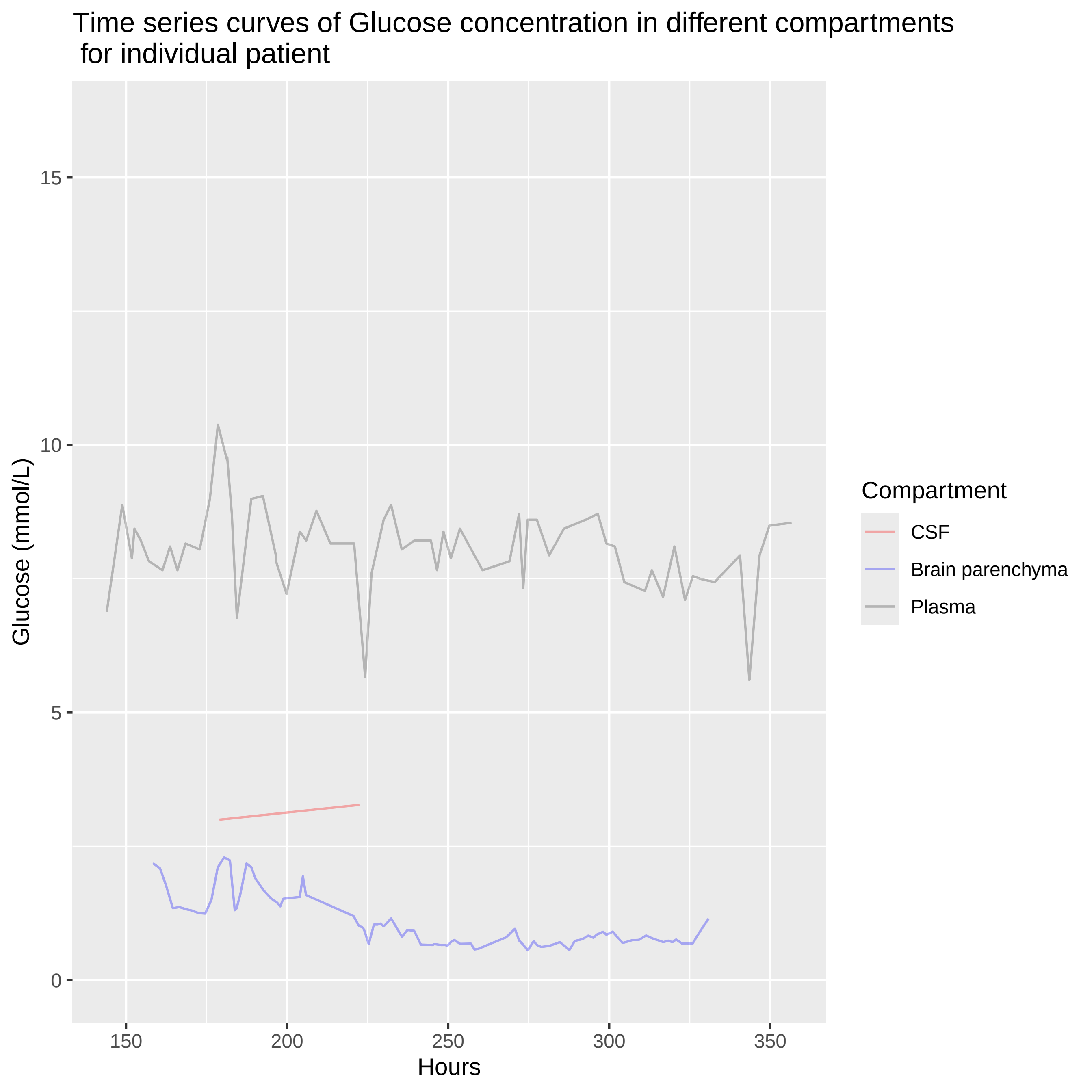

Time series curves of Glucose concentration in different compartments
for individual patient
Glucose (mmol/L)
0
5
10
15
150
200
250
300
350
Hours
Compartment
CSF
Brain parenchyma
Plasma

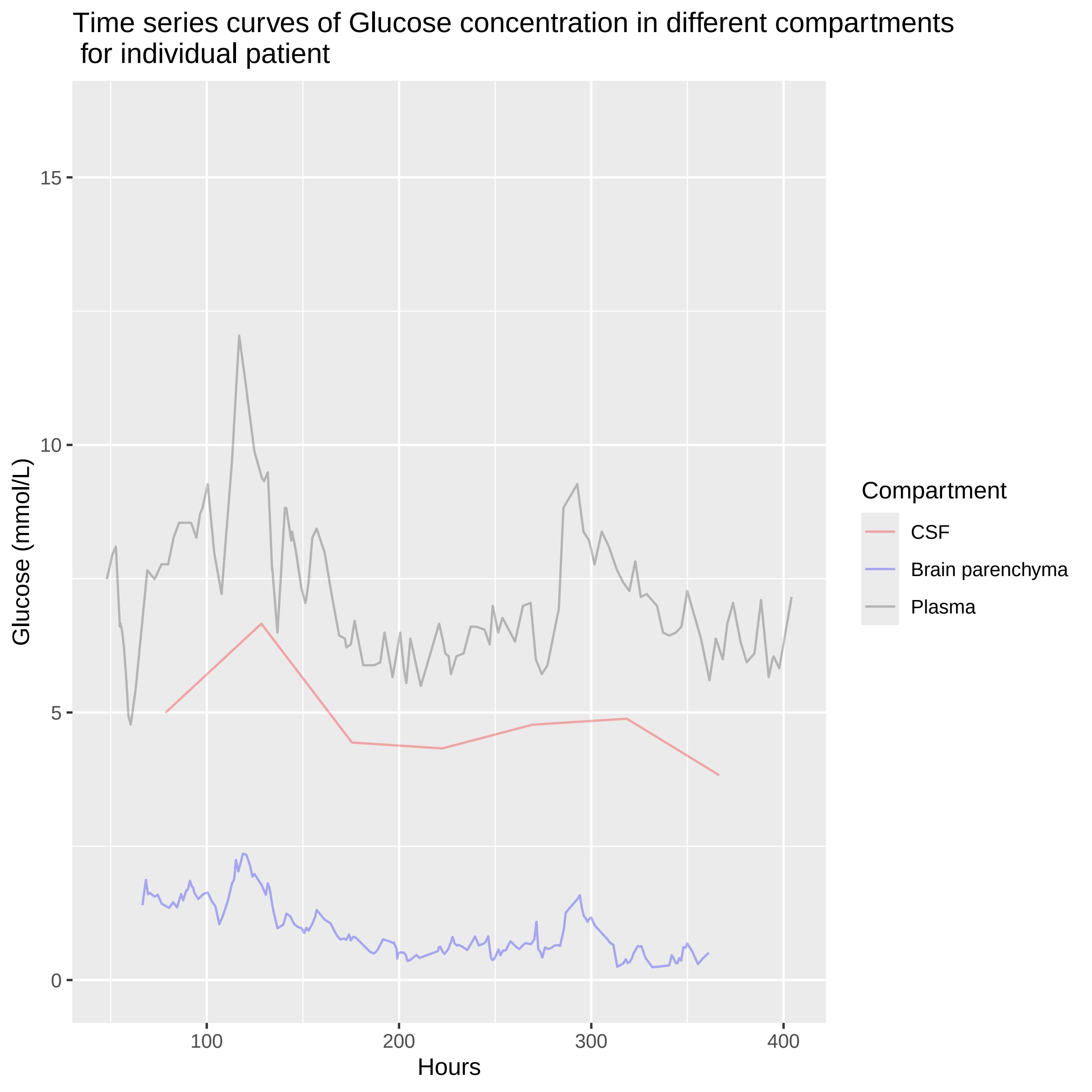

Time series curves of Glucose concentration in different compartments
for individual patient
Glucose (mmol/L)
15
10
5
0
100
200
300
400
Hours
Compartment
CSF
Brain parenchyma
Plasma

Time series curves of Glucose concentration in different compartments
for individual patient

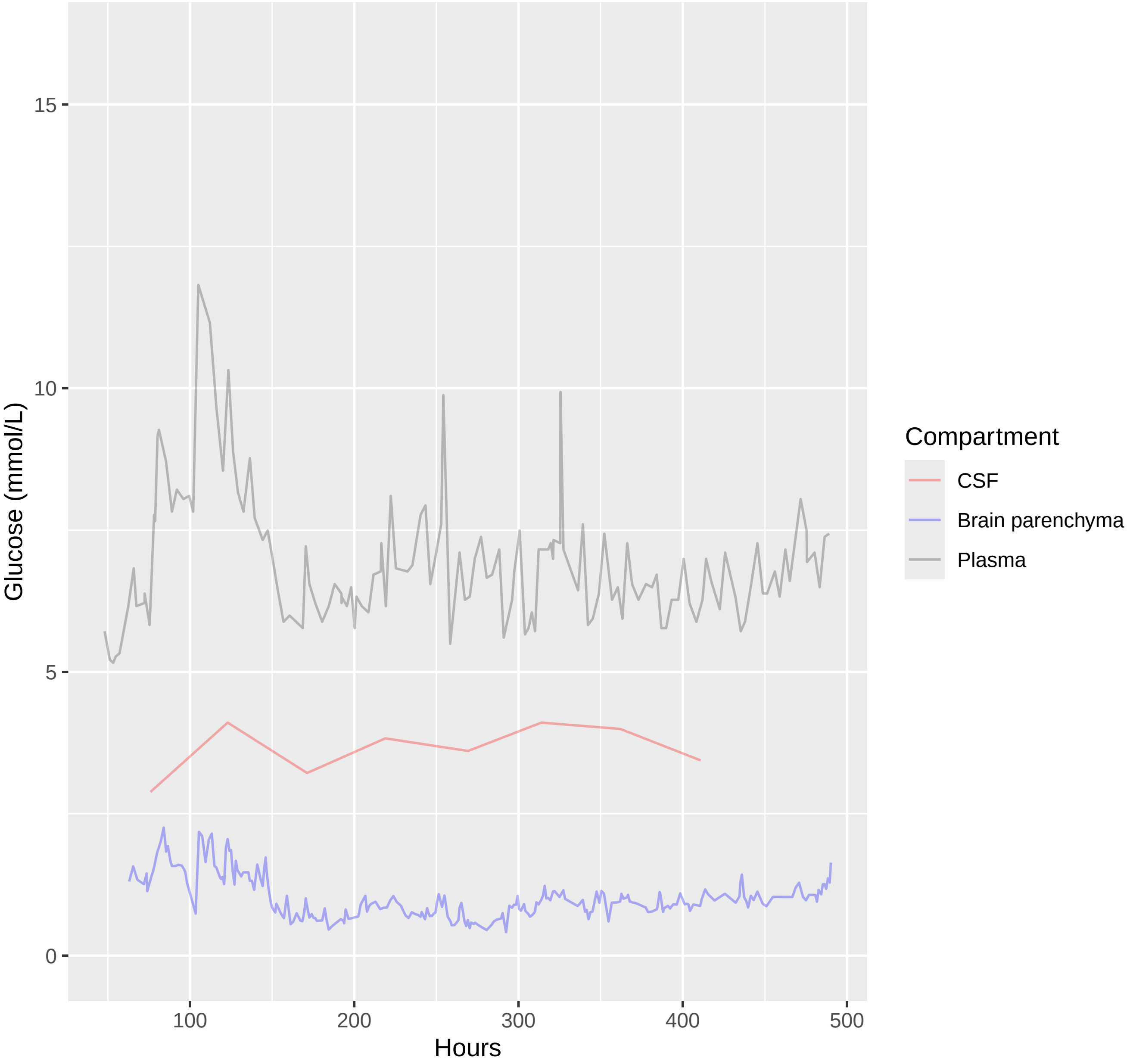

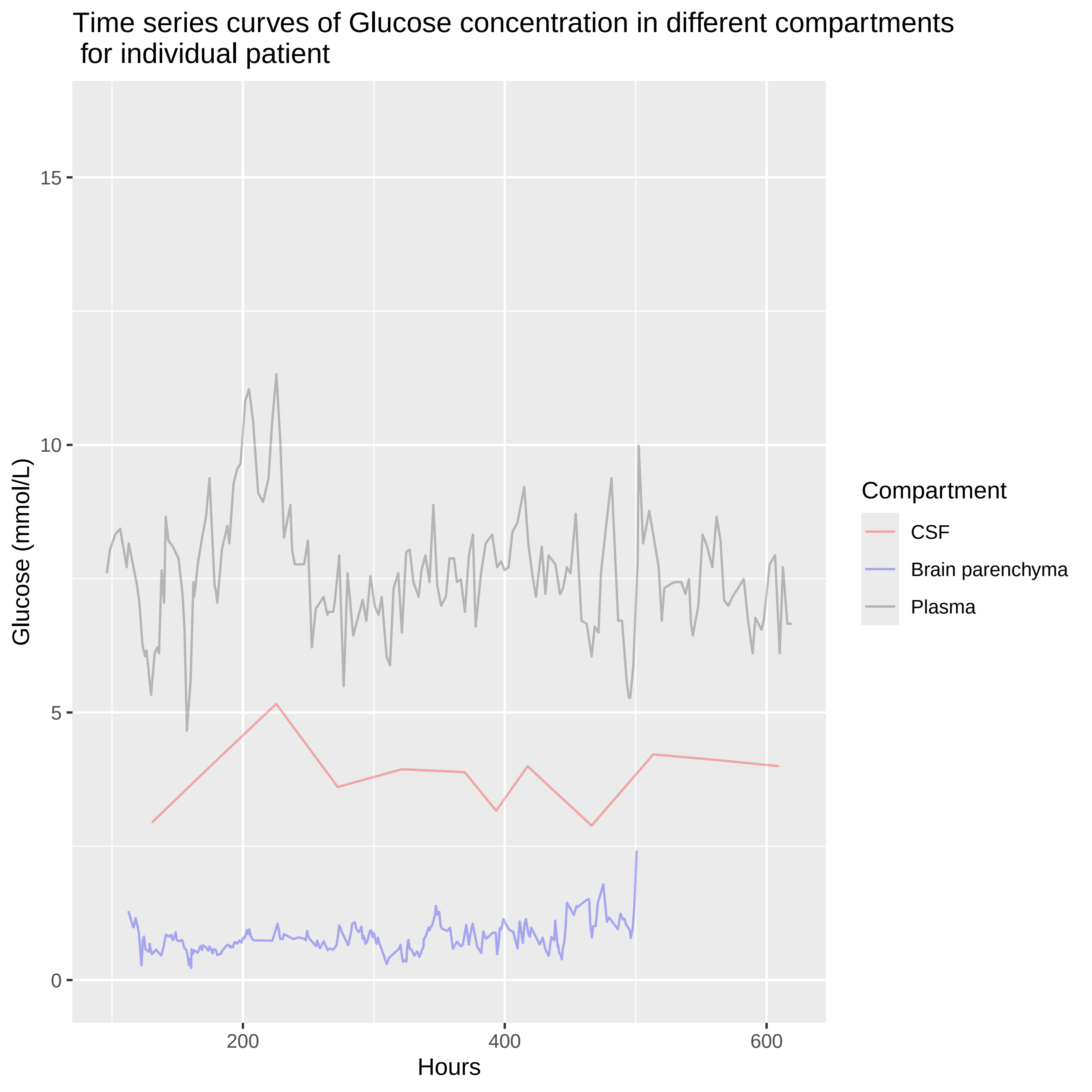
Time series curves of Glucose concentration in different compartments
for individual patient
15
10
5
0
Glucose (mmol/L)
200
400
600
Hours
Compartment
CSF
Brain parenchyma
Plasma

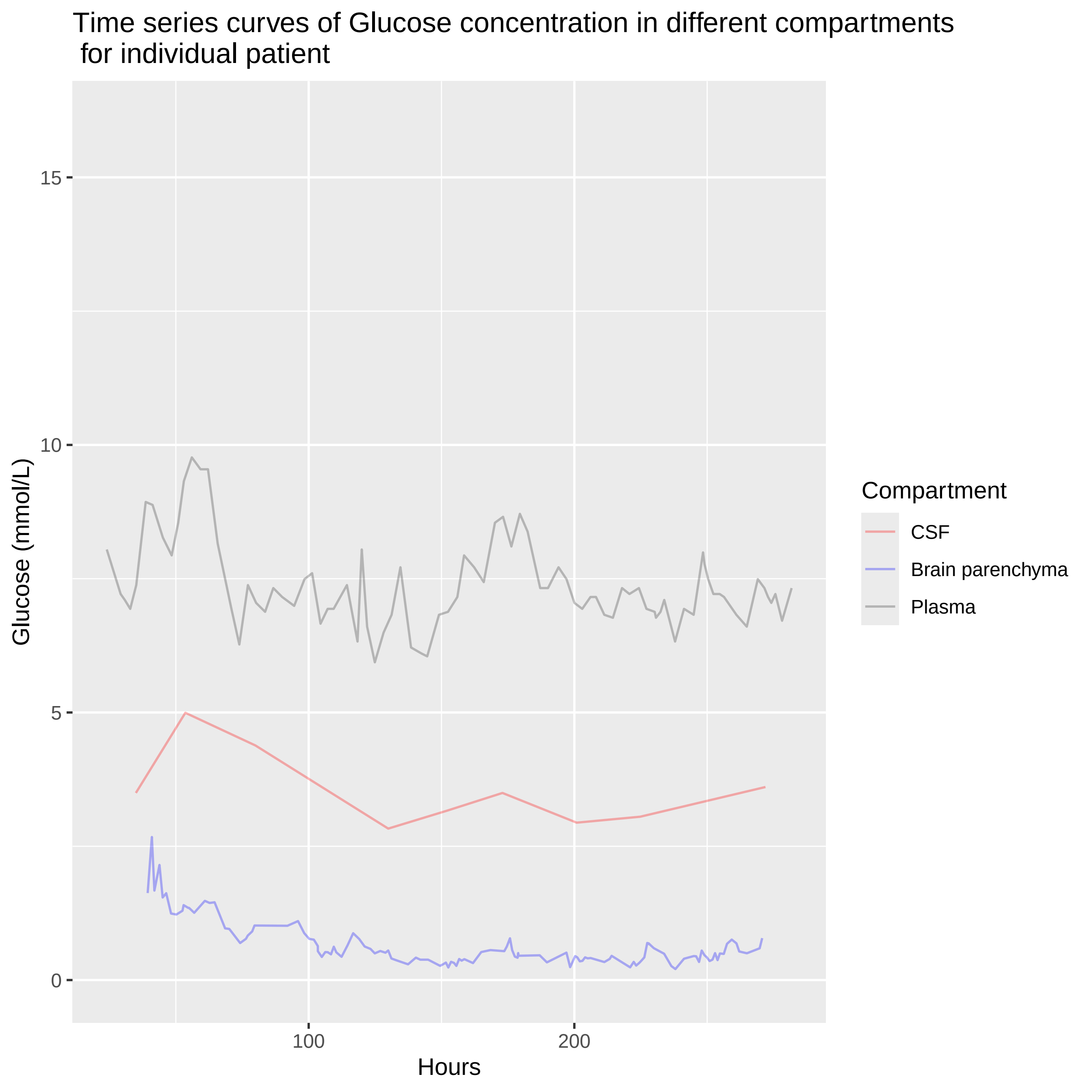
Time series curves of Glucose concentration in different compartments
for individual patient
15
10
5
0
Glucose (mmol/L)
100
200
Hours
Compartment
CSF
Brain parenchyma
Plasma

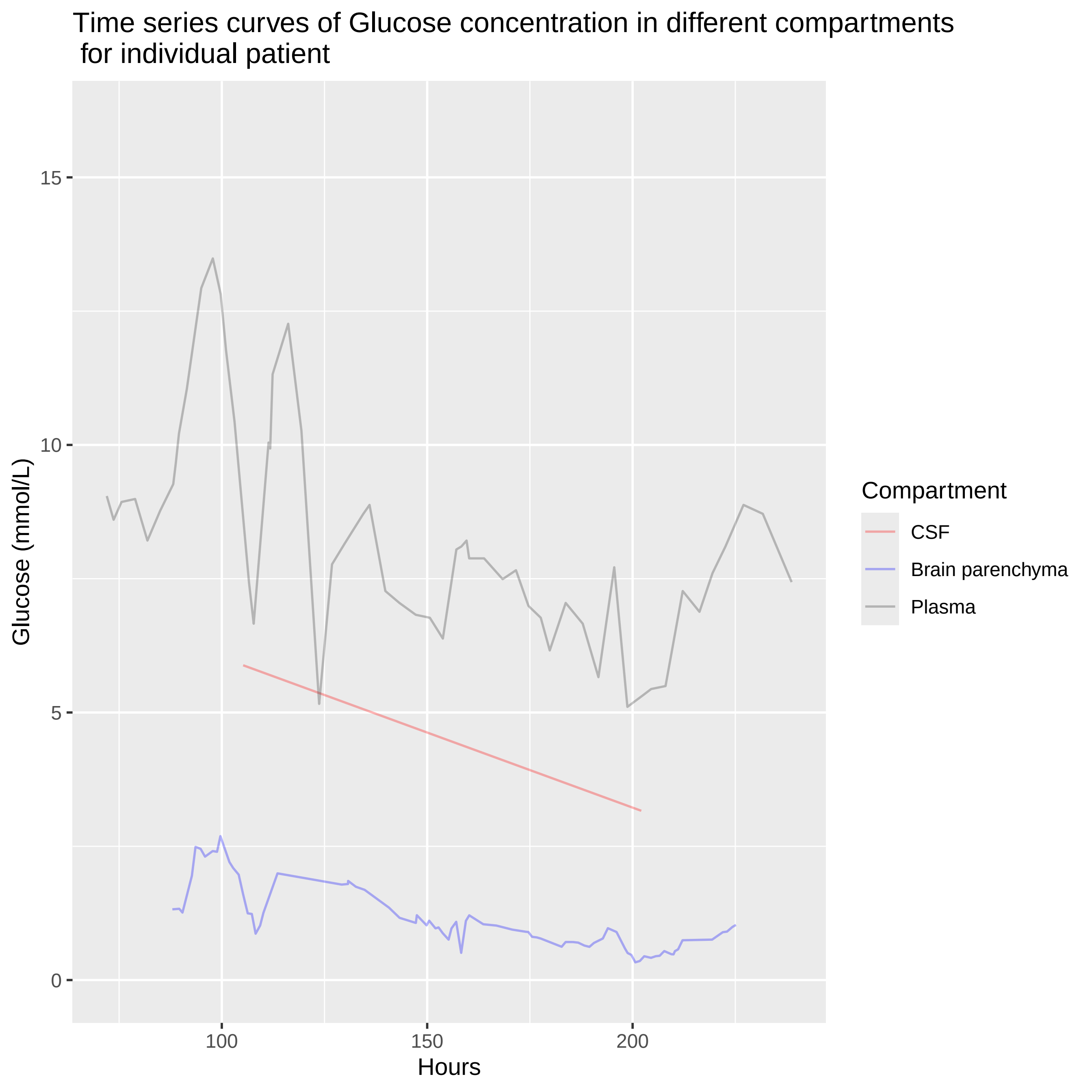

Time series curves of Glucose concentration in different compartments
for individual patient
Glucose (mmol/L)
15
10
5
0
100
150
200
Hours
Compartment
CSF
Brain parenchyma
Plasma

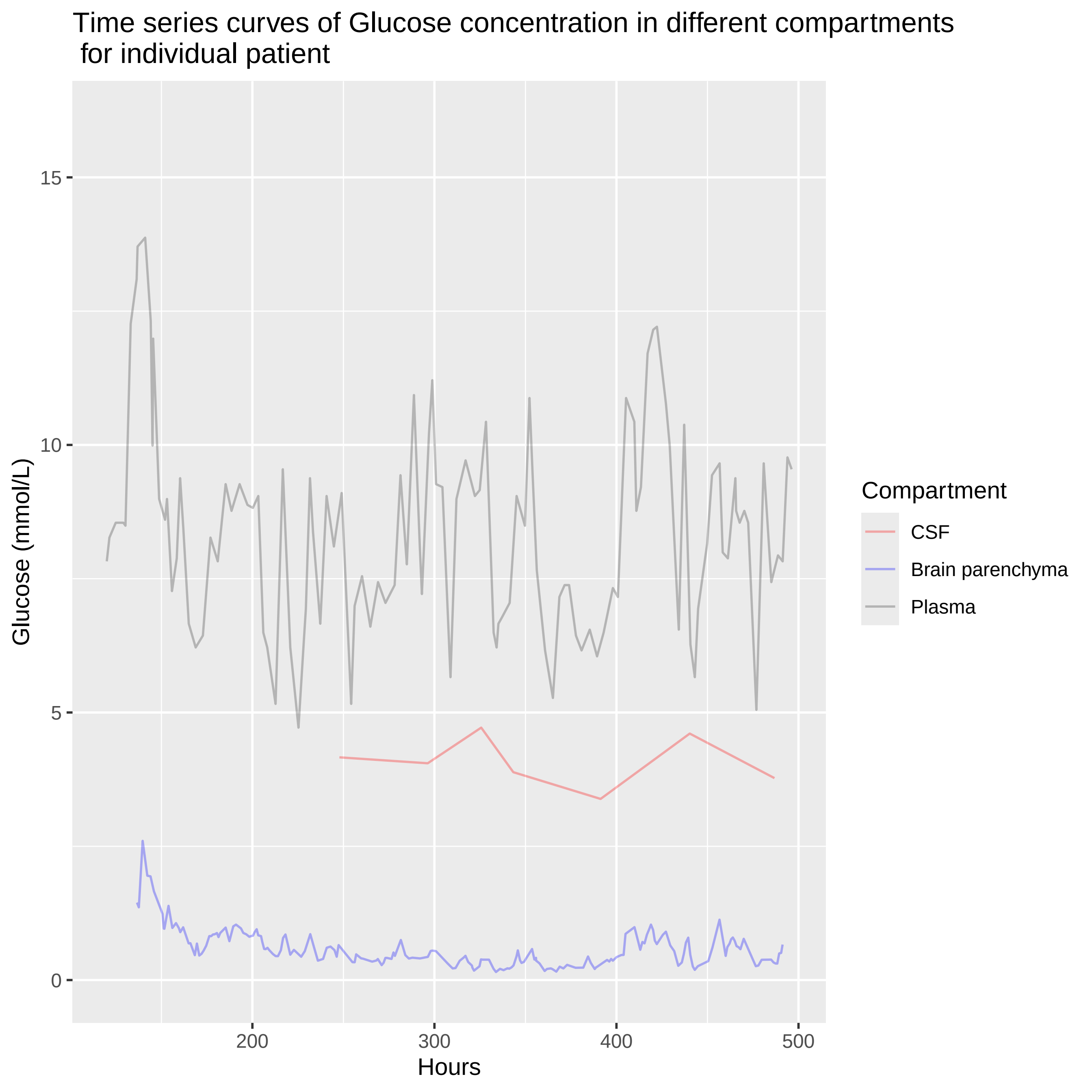

Time series curves of Glucose concentration in different compartments for individual patient
Glucose (mmol/L)
15
10
5
0
200
300
400
500
Hours
Compartment
CSF
Brain parenchyma
Plasma

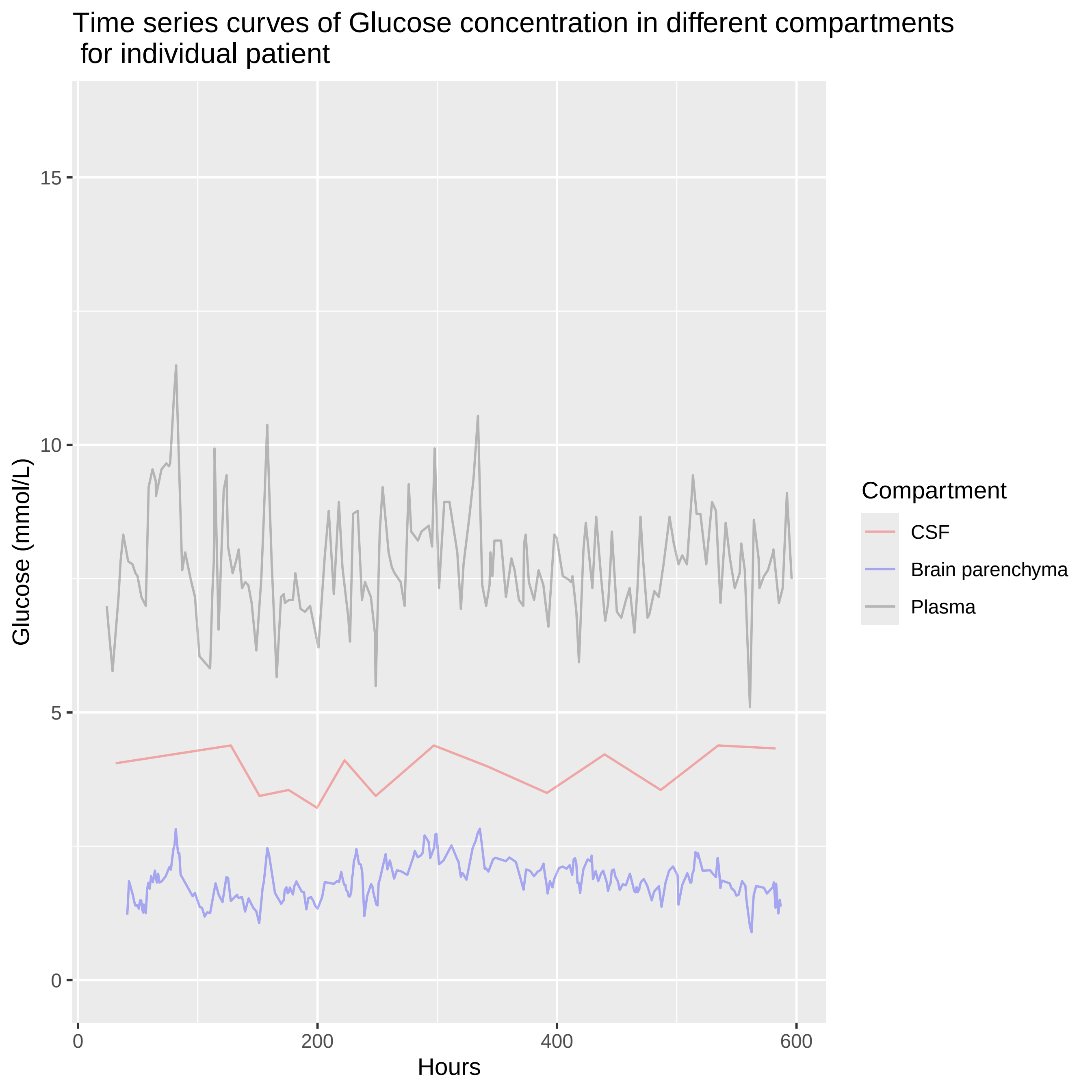
Time series curves of Glucose concentration in different compartments
for individual patient
Glucose (mmol/L)
15
10
5
0
0
200
400
600
Hours
Compartment
CSF
Brain parenchyma
Plasma

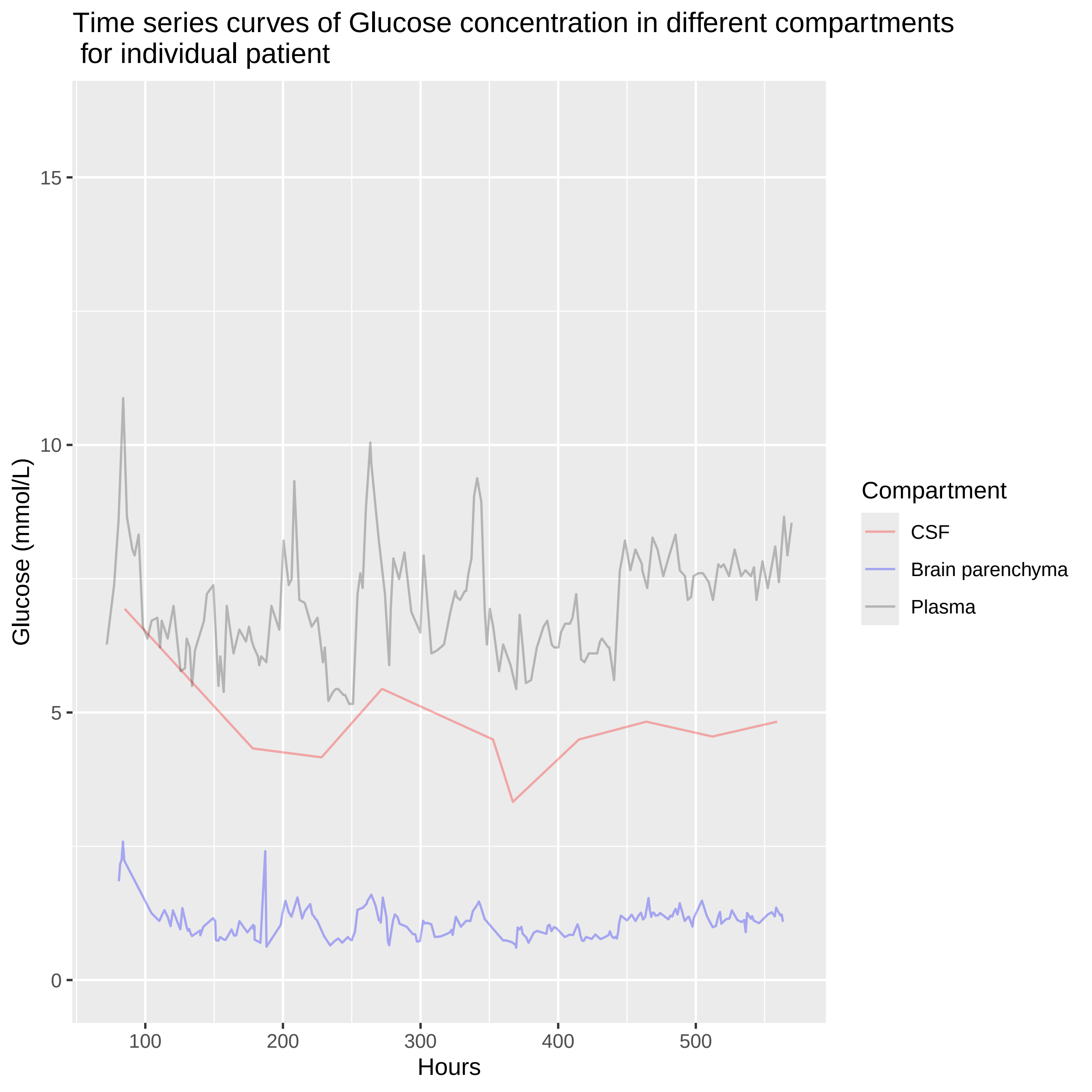

Time series curves of Glucose concentration in different compartments
for individual patient
Glucose (mmol/L)
15
10
5
0
100
200
300
400
500
Hours
Compartment
CSF
Brain parenchyma
Plasma

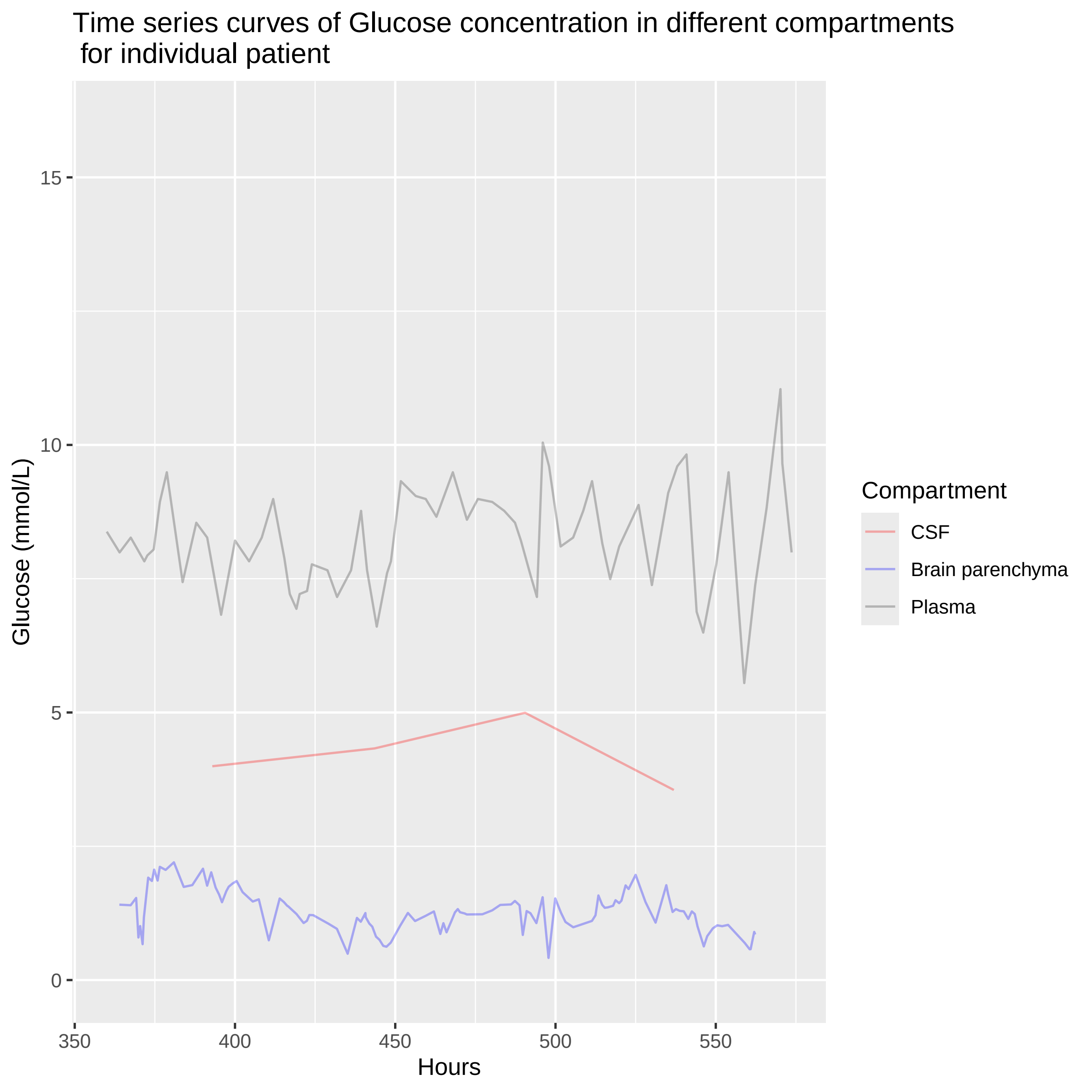

Time series curves of Glucose concentration in different compartments
for individual patient
Glucose (mmol/L)
Hours
0
5
10
15
350
400
450
500
550
Compartment
CSF
Brain parenchyma
Plasma

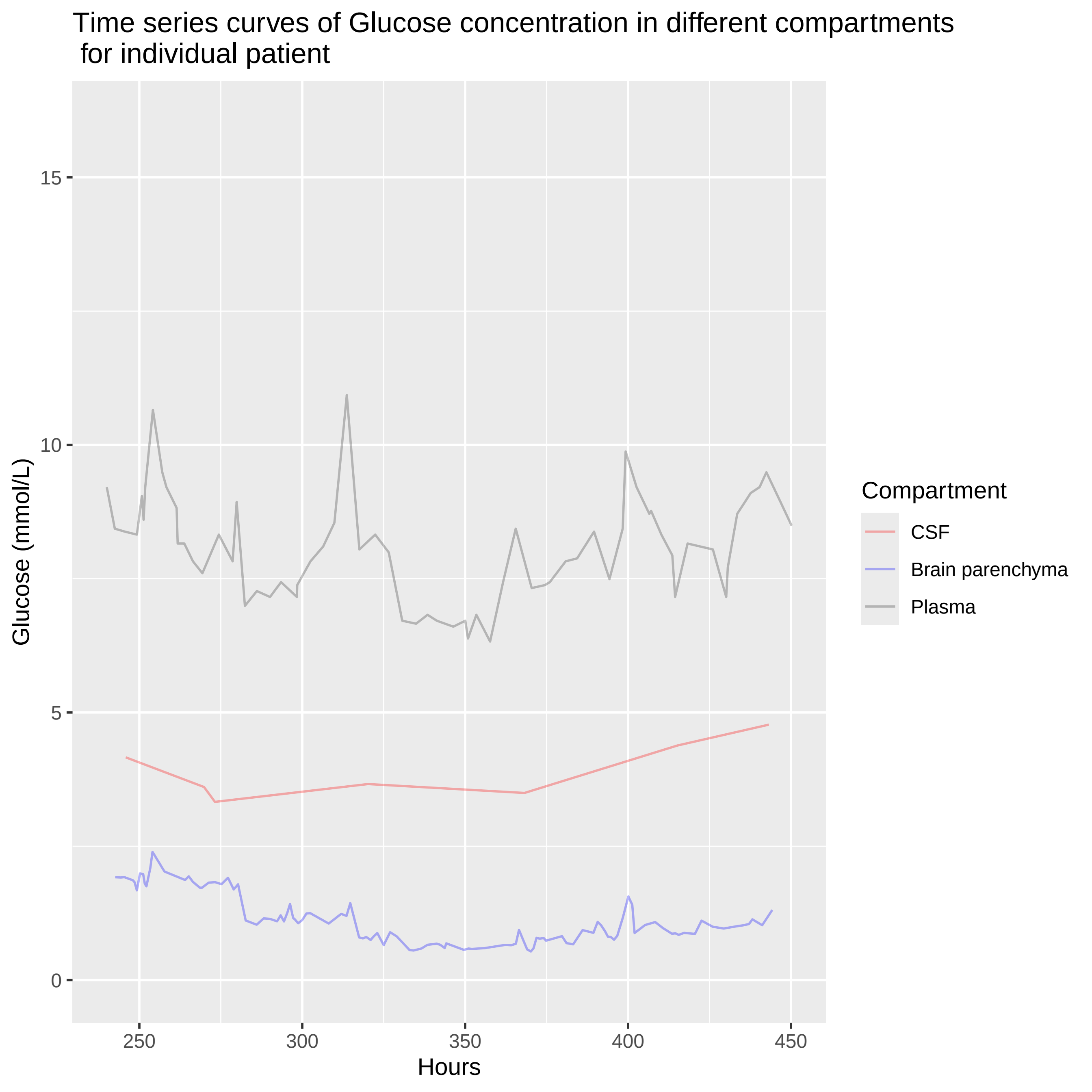

Time series curves of Glucose concentration in different compartments
for individual patient
Glucose (mmol/L)
Hours
0
5
10
15
250
300
350
400
450
Compartment
CSF
Brain parenchyma
Plasma

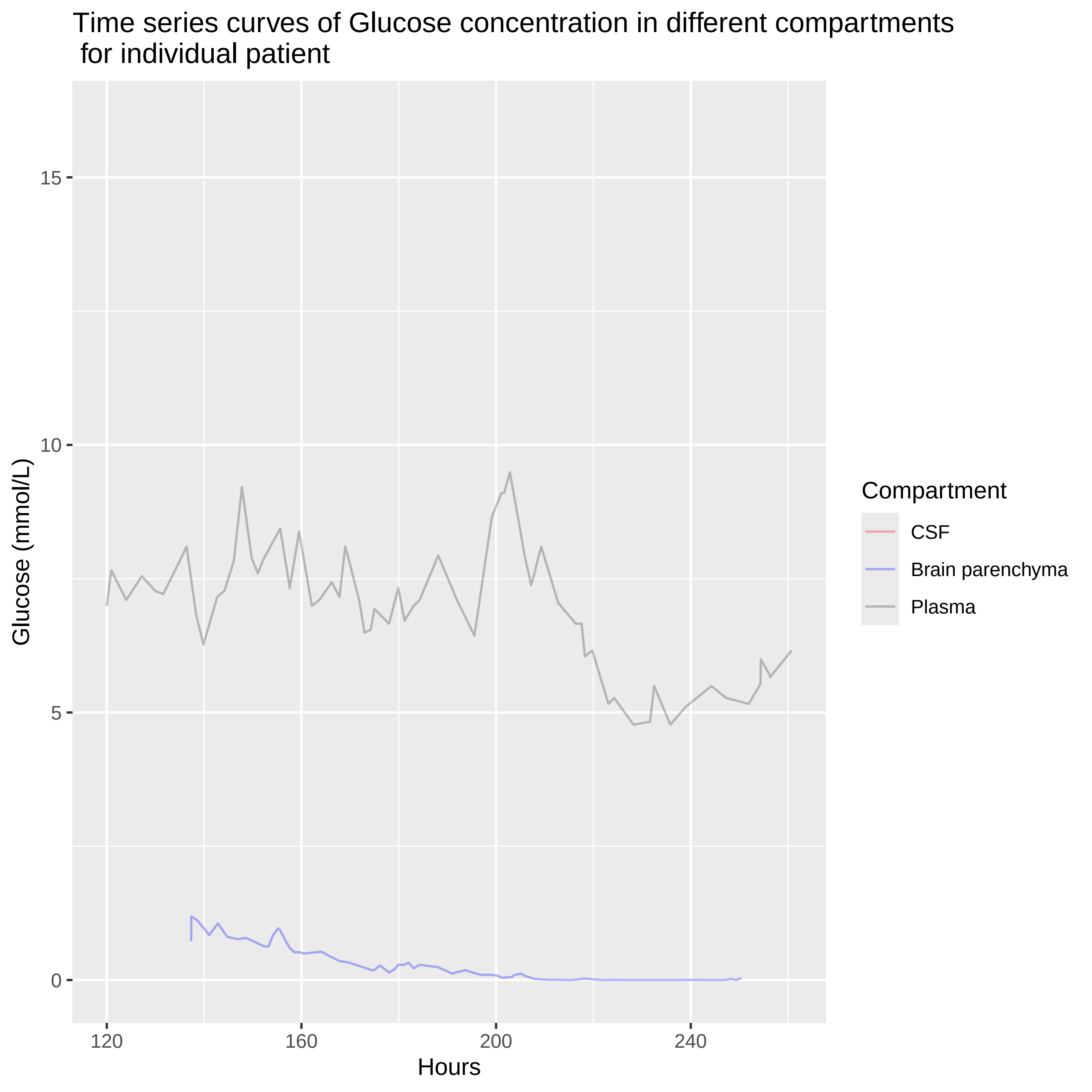

Time series curves of Glucose concentration in different compartments
for individual patient
Glucose (mmol/L)
15
10
5
0
120
160
200
240
Hours
Compartment
CSF
Brain parenchyma
Plasma

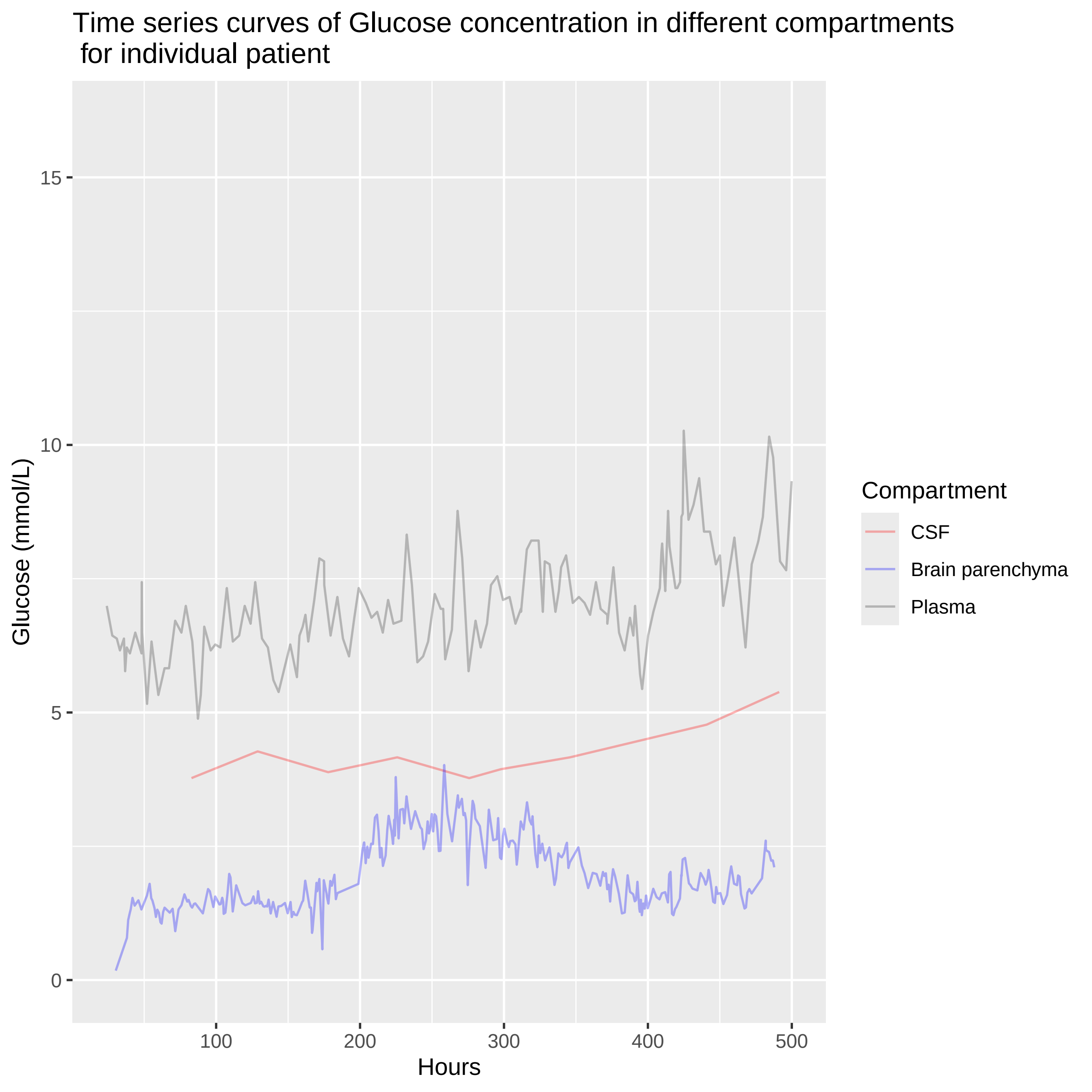

Time series curves of Glucose concentration in different compartments
for individual patient
Glucose (mmol/L)
15
10
5
0
100
200
300
400
500
Hours
Compartment
CSF
Brain parenchyma
Plasma

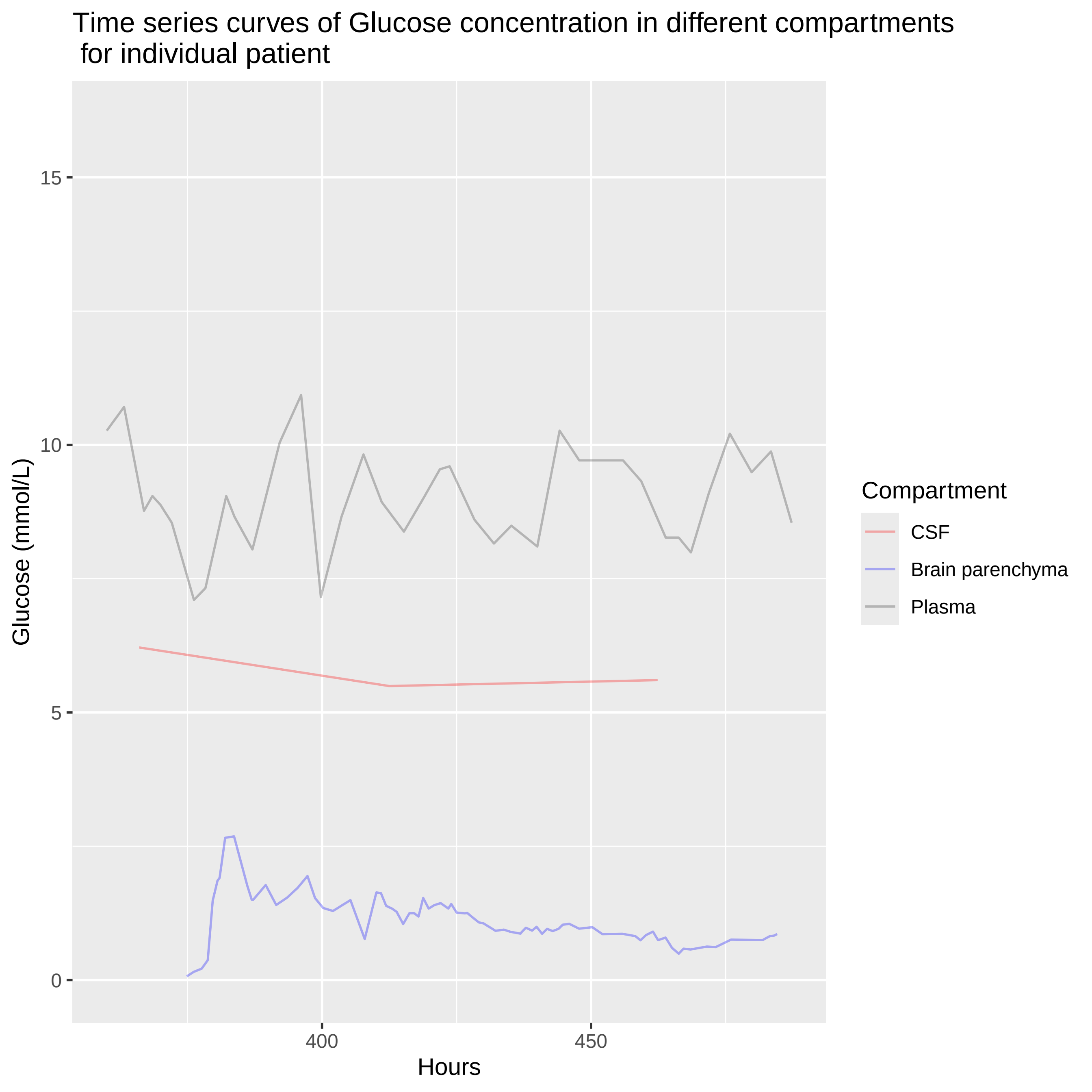
Time series curves of Glucose concentration in different compartments
for individual patient
15
10
5
0
400
450
Hours
Glucose (mmol/L)
Compartment
CSF
Brain parenchyma
Plasma

Time series curves of Glucose concentration in different compartments
for individual patient

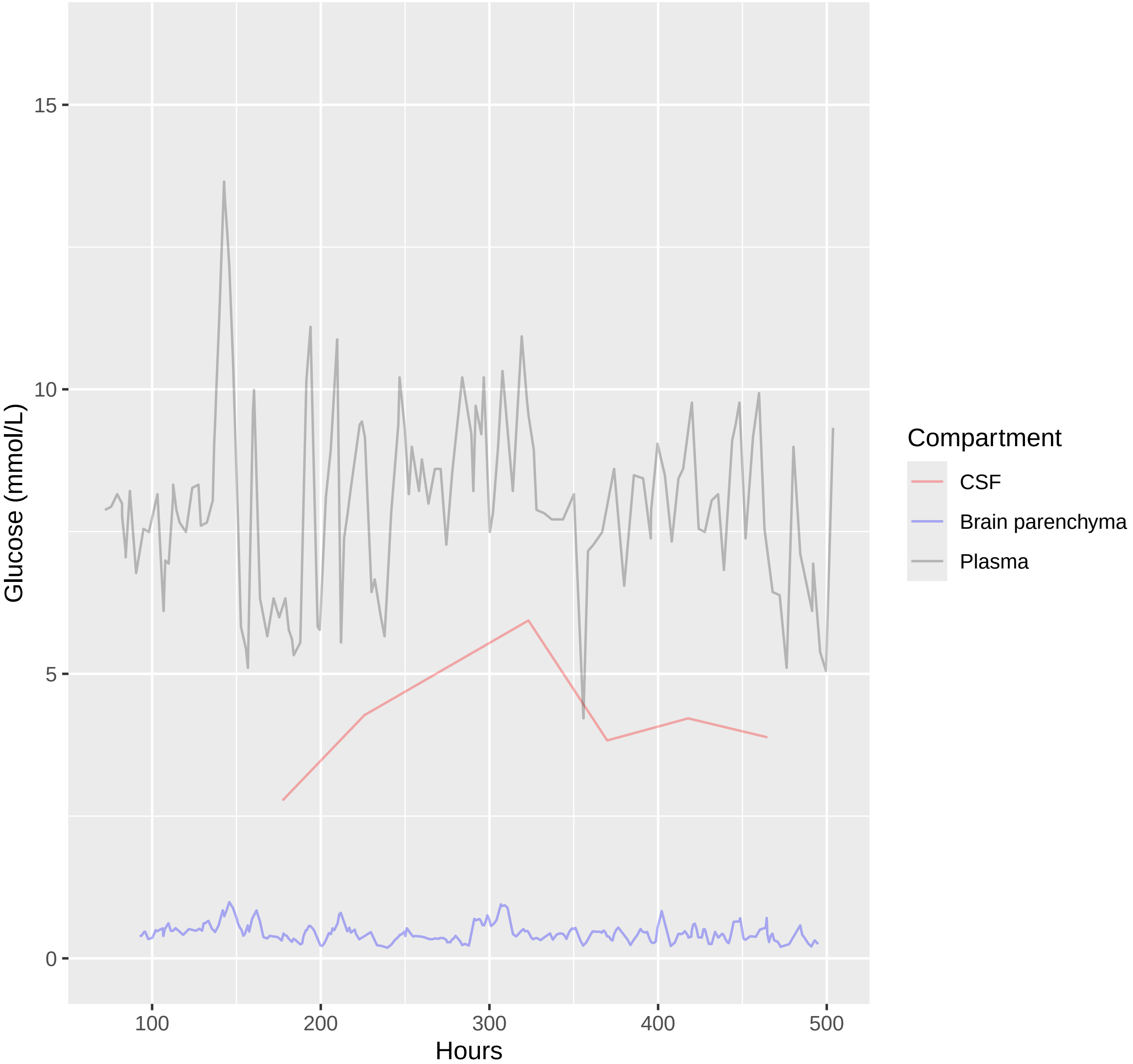

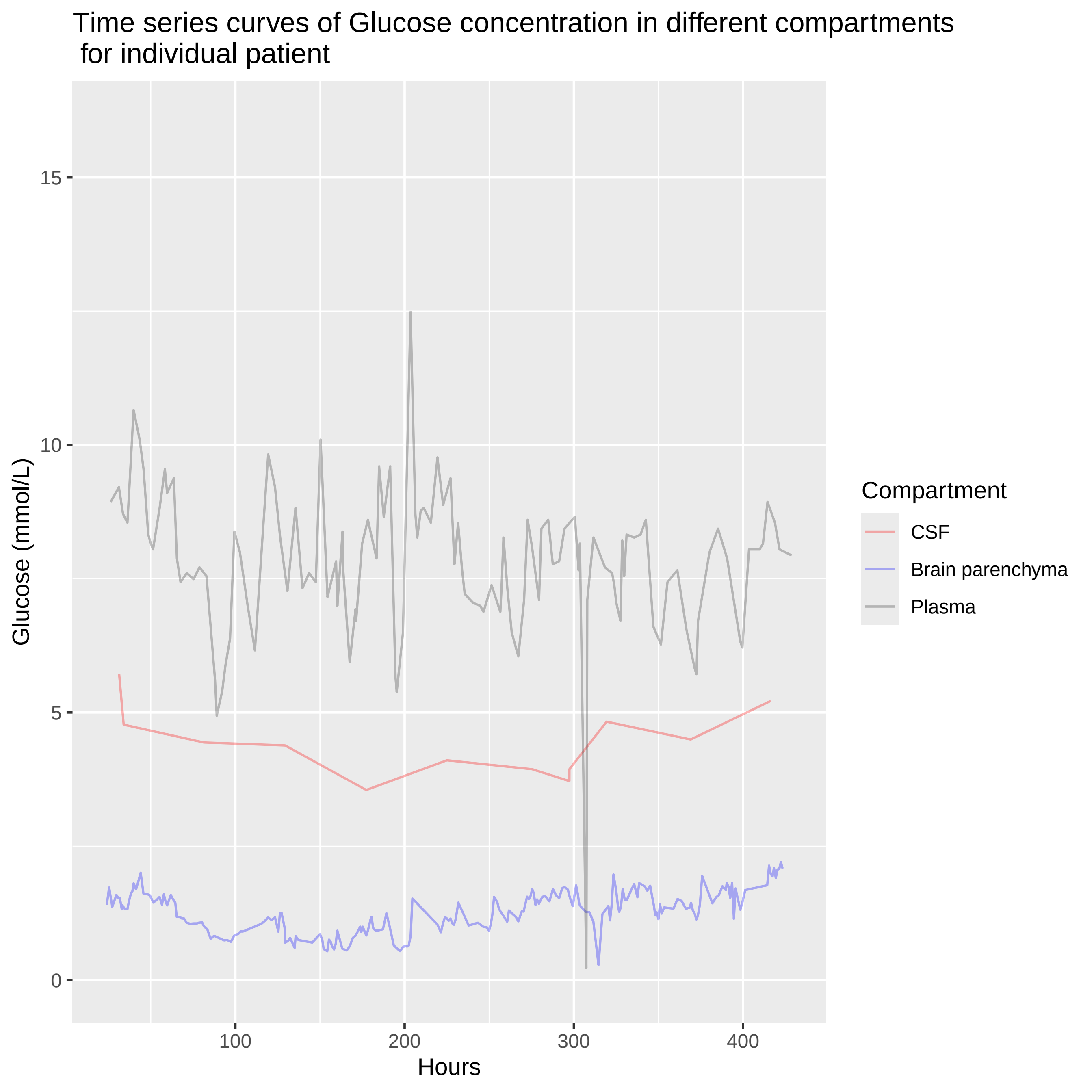

Time series curves of Glucose concentration in different compartments
for individual patient
Glucose (mmol/L)
15
10
5
0
100
200
300
400
Hours
Compartment
CSF
Brain parenchyma
Plasma

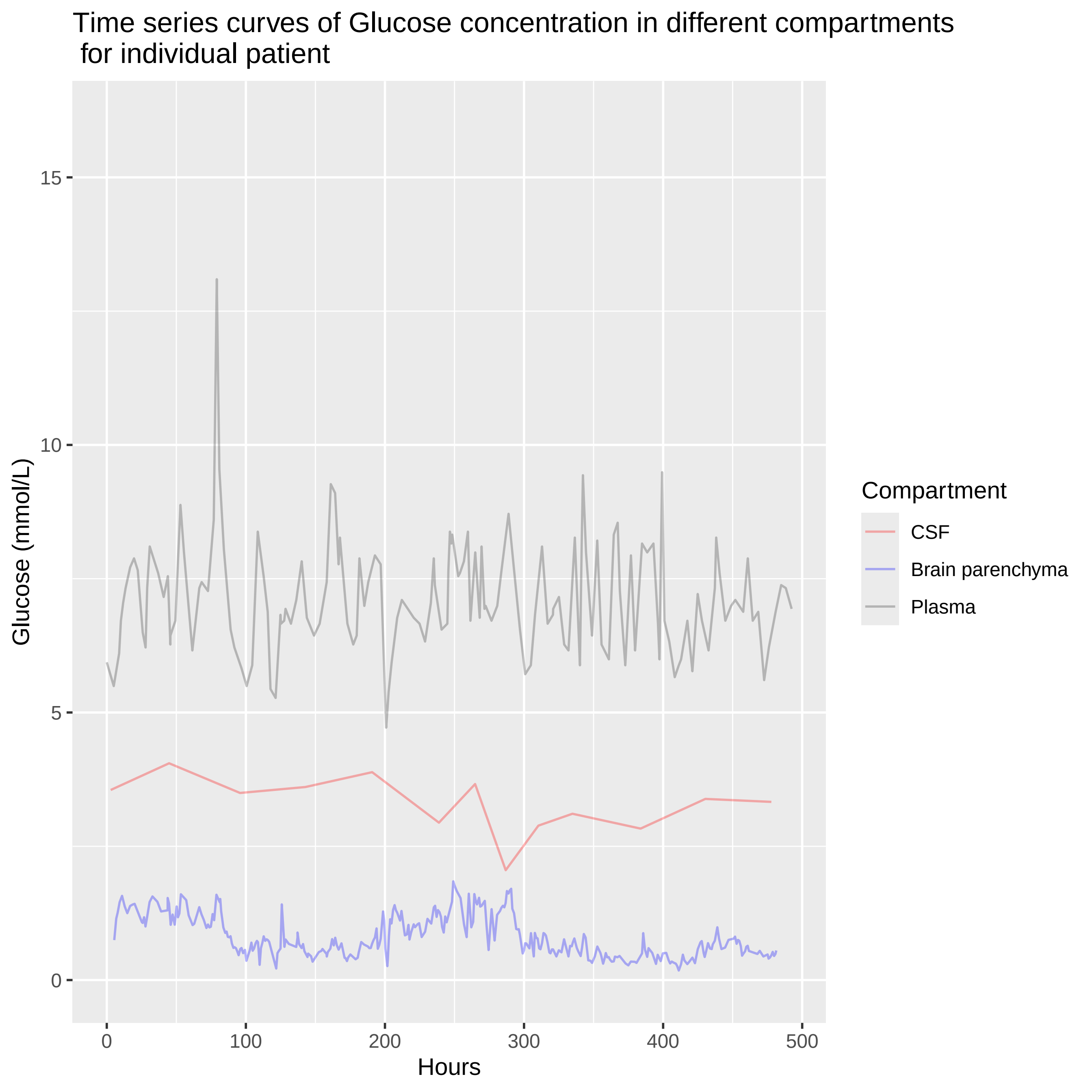
Time series curves of Glucose concentration in different compartments
for individual patient
Glucose (mmol/L)
0
5
10
15
Hours
0
100
200
300
400
500
Compartment
CSF
Brain parenchyma
Plasma

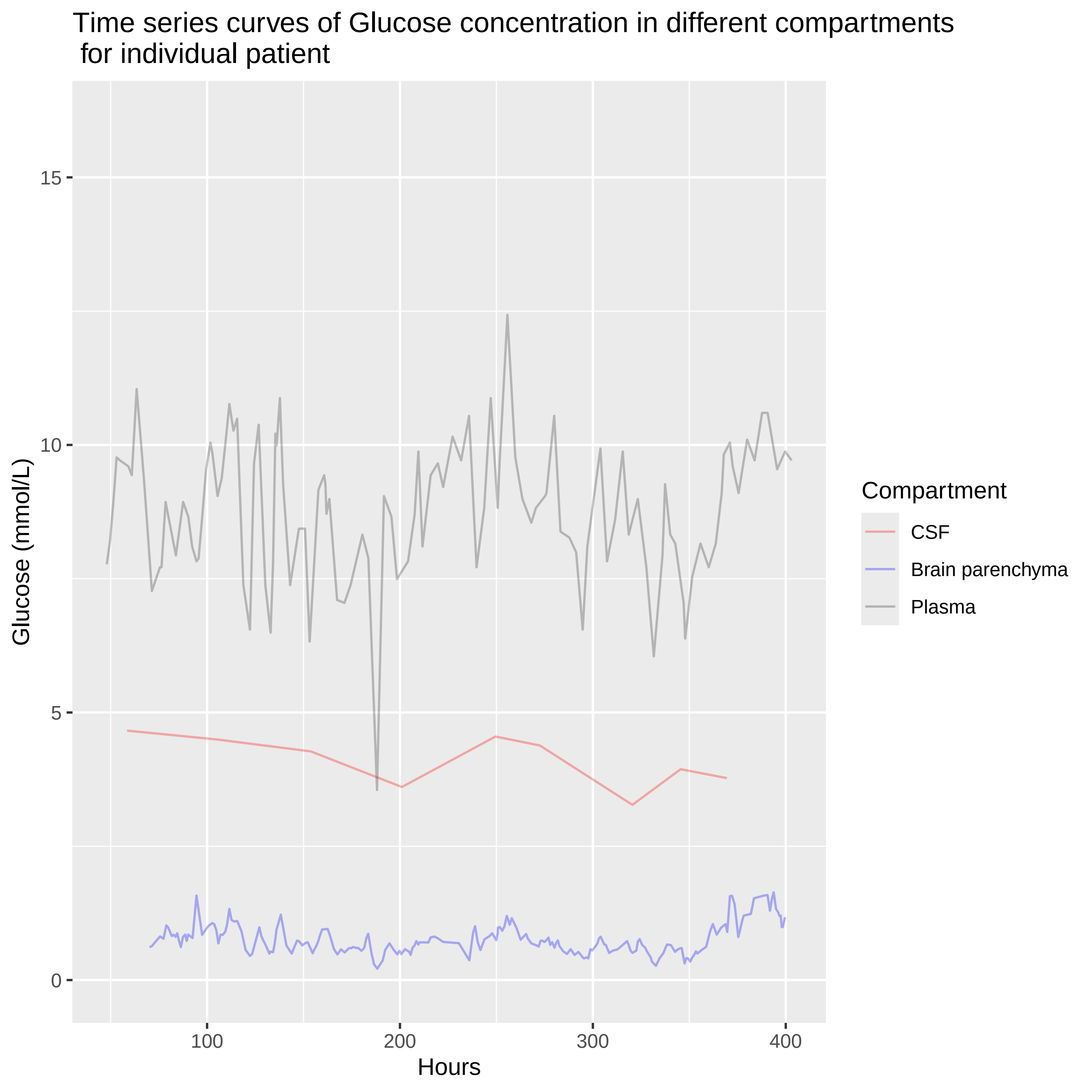

Time series curves of Glucose concentration in different compartments
for individual patient
Glucose (mmol/L)
15
10
5
0
100
200
300
400
Hours
Compartment
CSF
Brain parenchyma
Plasma

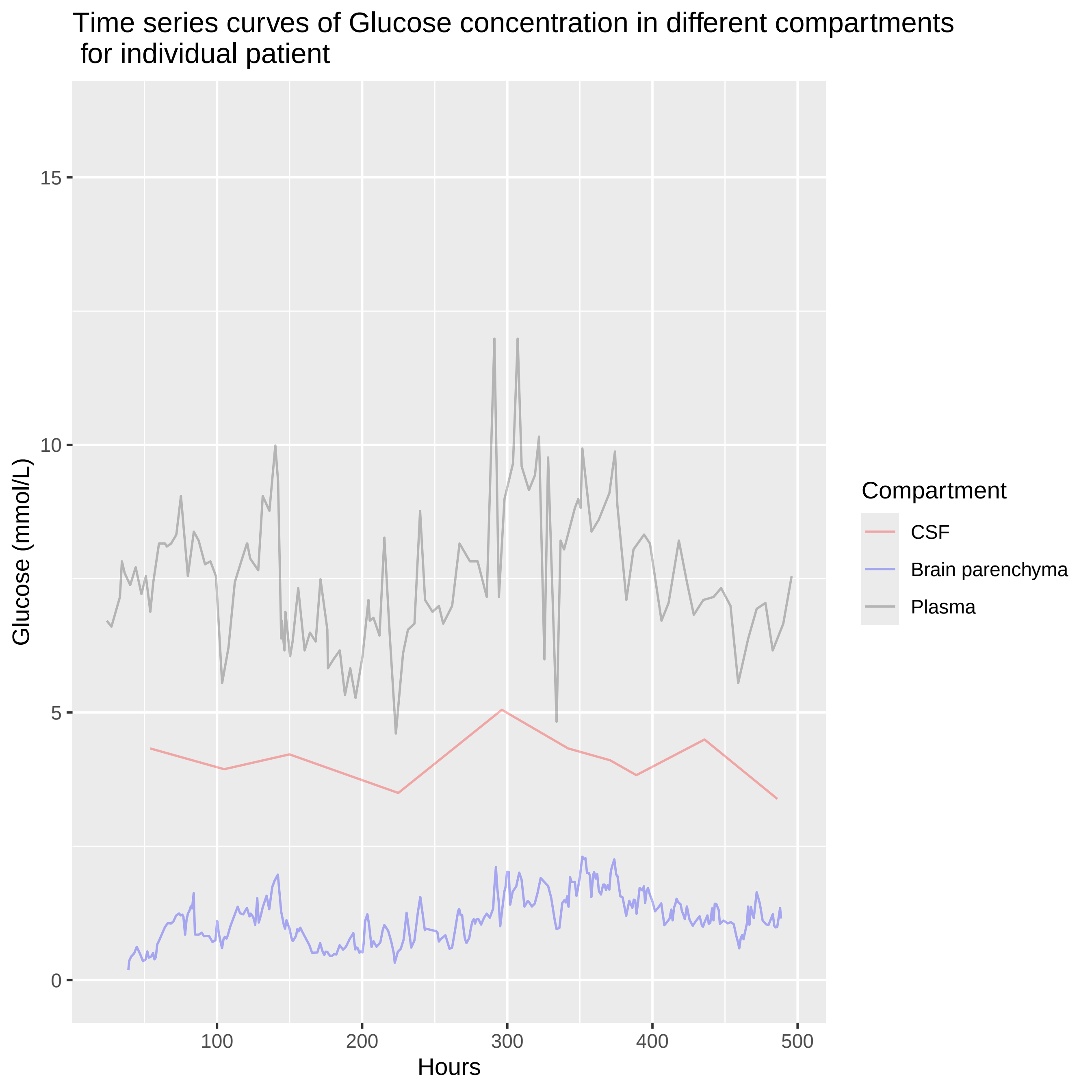

Time series curves of Glucose concentration in different compartments
for individual patient
Glucose (mmol/L)
15
10
5
0
100
200
300
400
500
Hours
Compartment
CSF
Brain parenchyma
Plasma

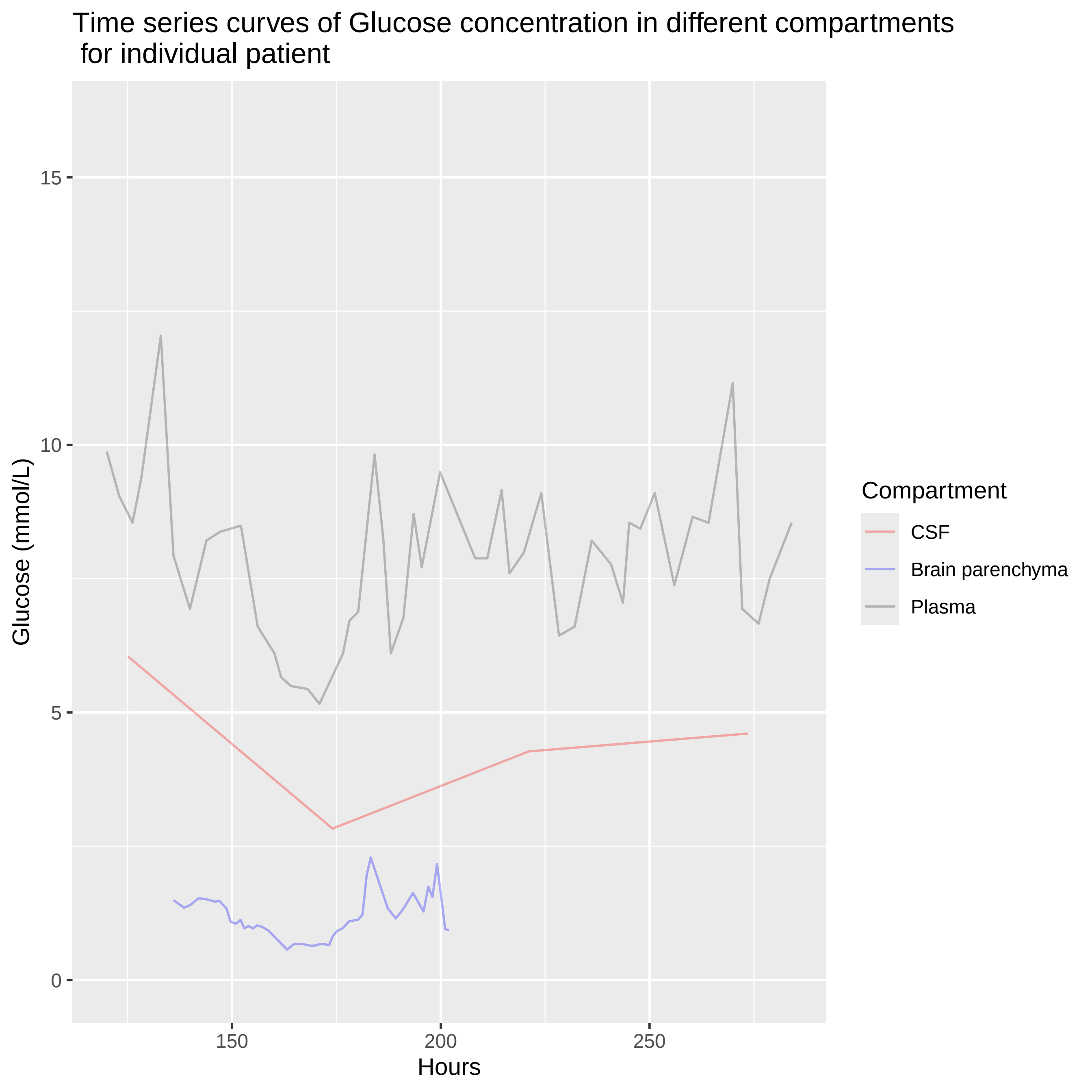

Time series curves of Glucose concentration in different compartments
for individual patient
Glucose (mmol/L)
15
10
5
0
150
200
250
Hours
Compartment
CSF
Brain parenchyma
Plasma

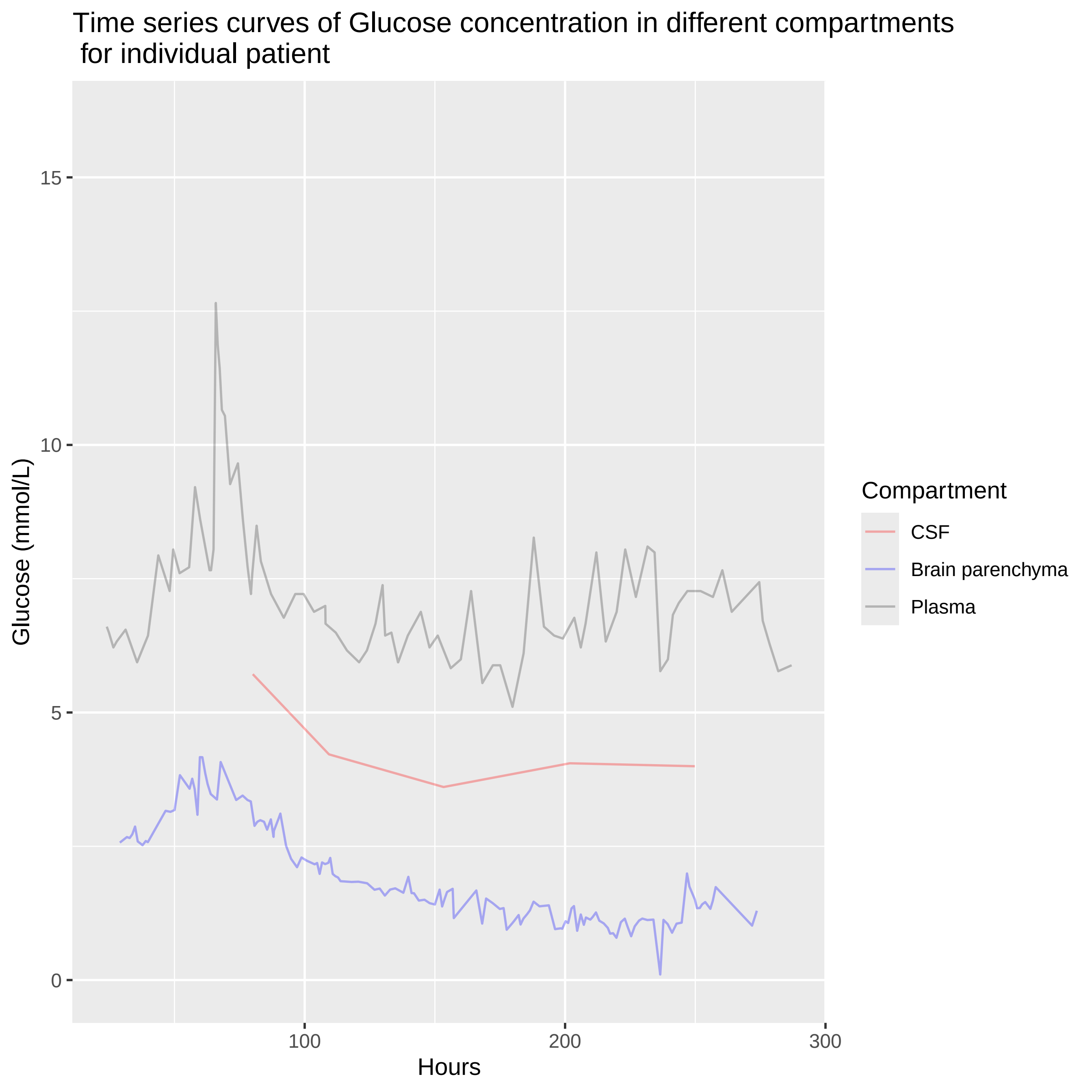

Time series curves of Glucose concentration in different compartments for individual patient
Glucose (mmol/L)
Hours
0
5
10
15
100
200
300
Compartment
CSF
Brain parenchyma
Plasma

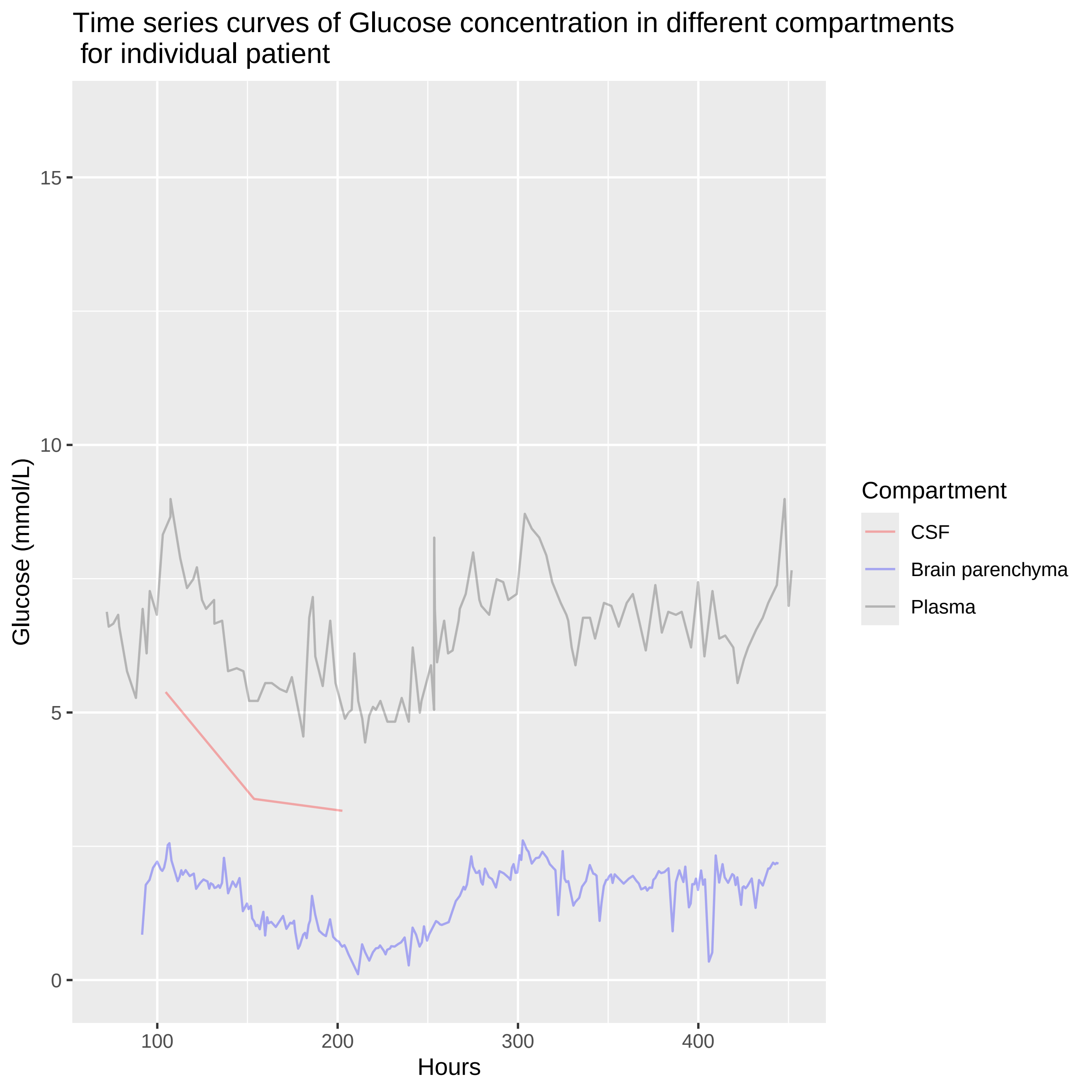
Time series curves of Glucose concentration in different compartments
for individual patient
Glucose (mmol/L)
15
10
5
0
100
200
300
400
Hours
Compartment
CSF
Brain parenchyma
Plasma

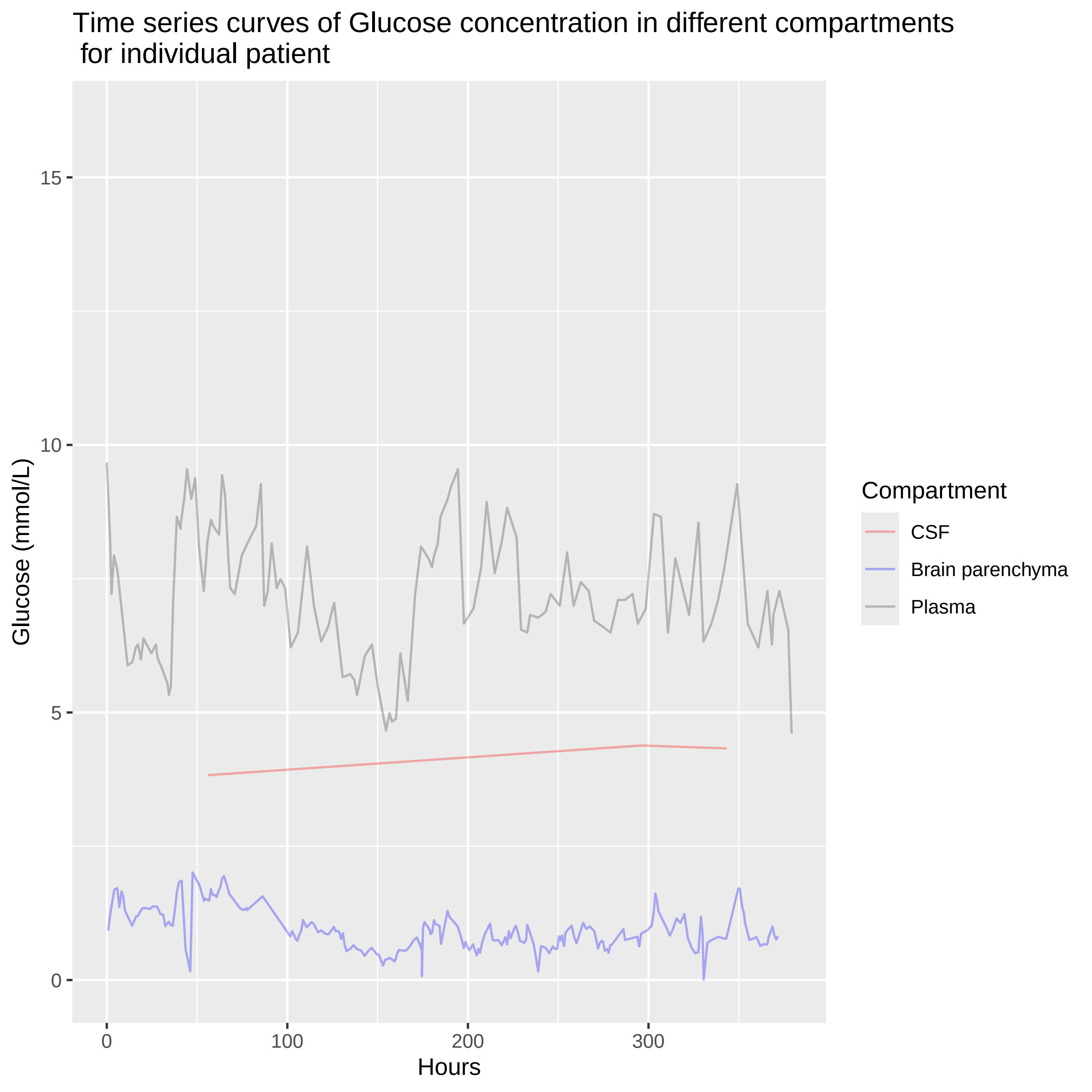
Time series curves of Glucose concentration in different compartments
for individual patient
Glucose (mmol/L)
15
10
5
0
0
100
200
300
Hours
Compartment
CSF
Brain parenchyma
Plasma

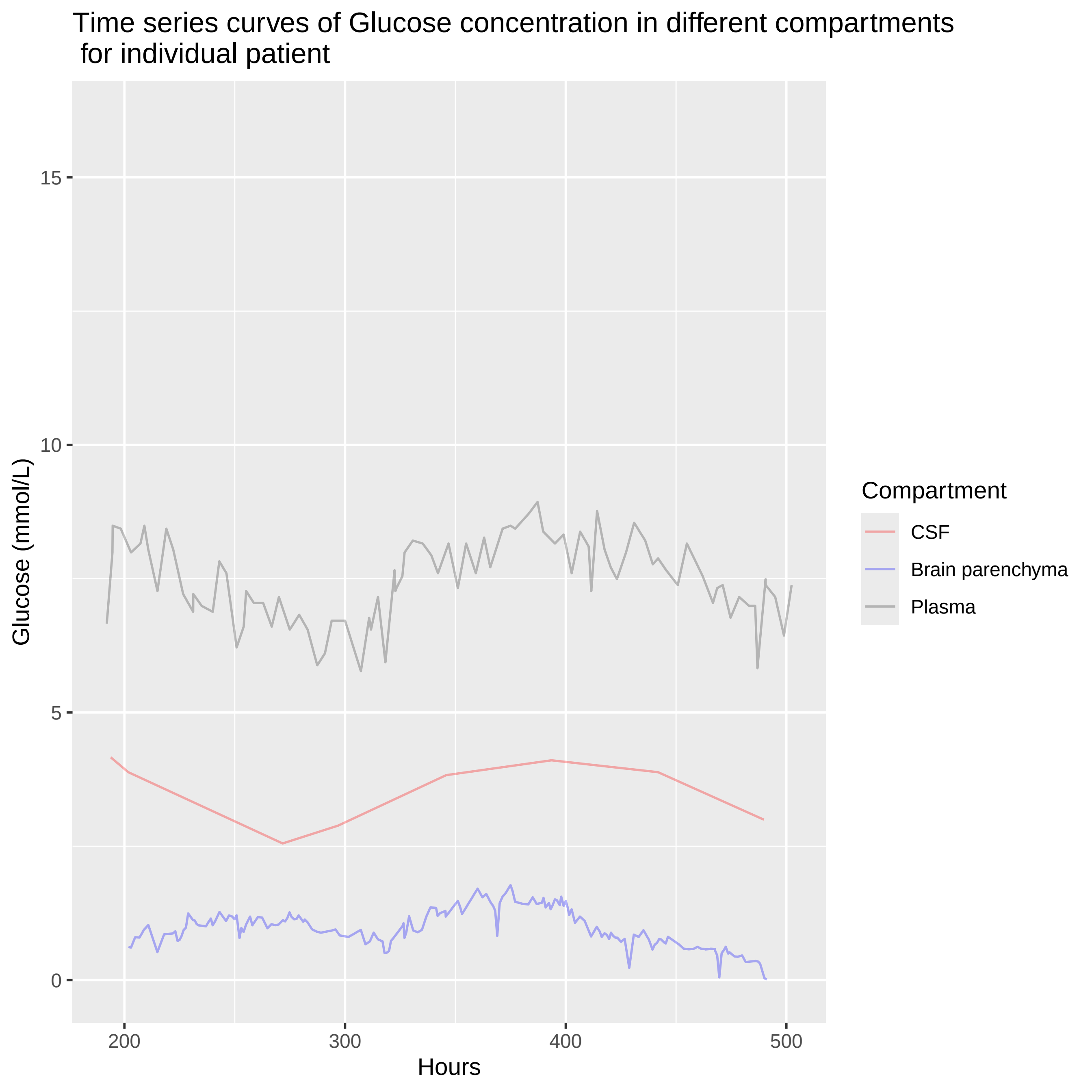

Time series curves of Glucose concentration in different compartments
for individual patient
Glucose (mmol/L)
15
10
5
0
200
300
400
500
Hours
Compartment
CSF
Brain parenchyma
Plasma

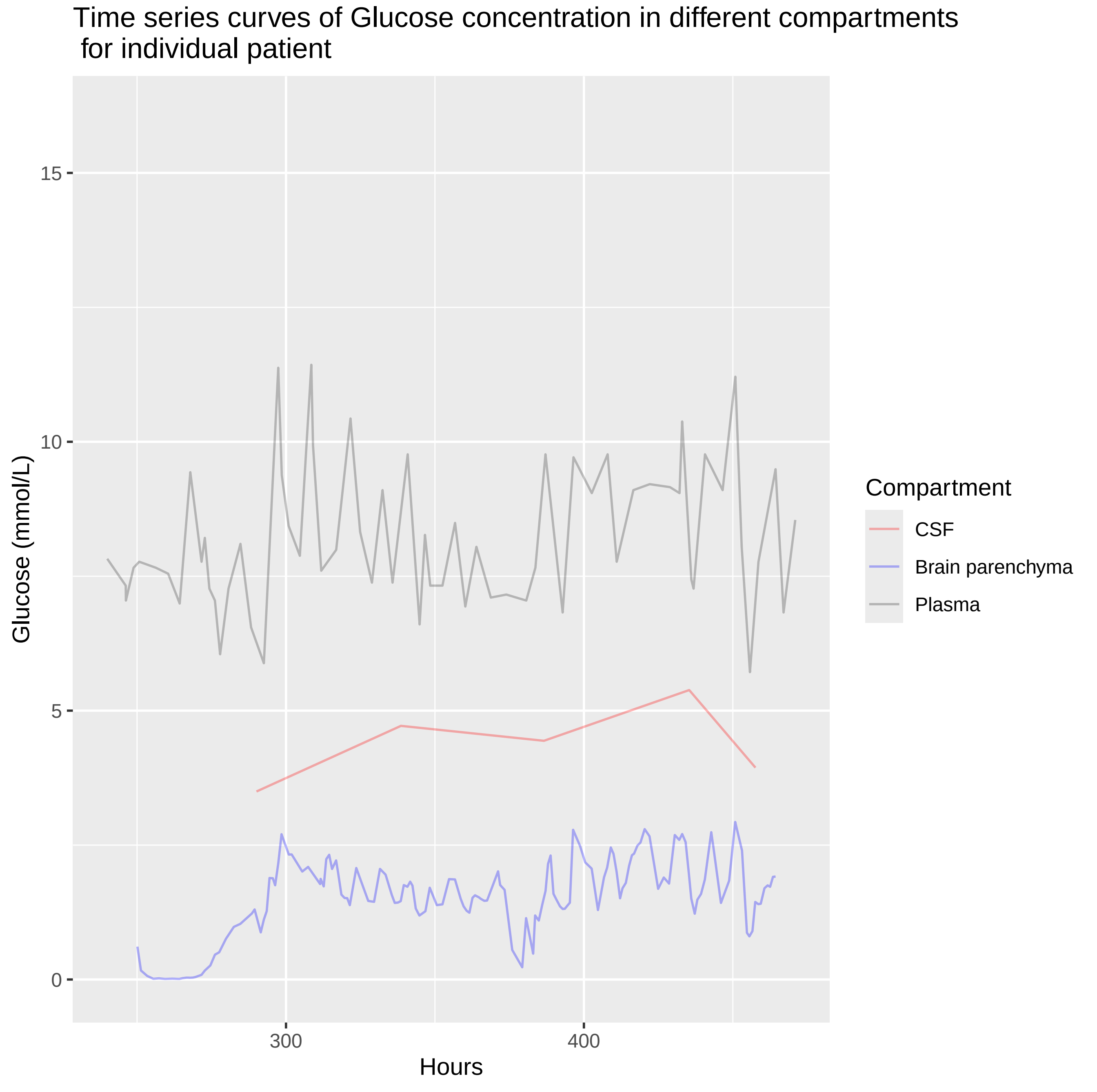

Time series curves of Glucose concentration in different compartments
for individual patient
Glucose (mmol/L)
15
10
5
0
300
400
Hours
Compartment
CSF
Brain parenchyma
Plasma

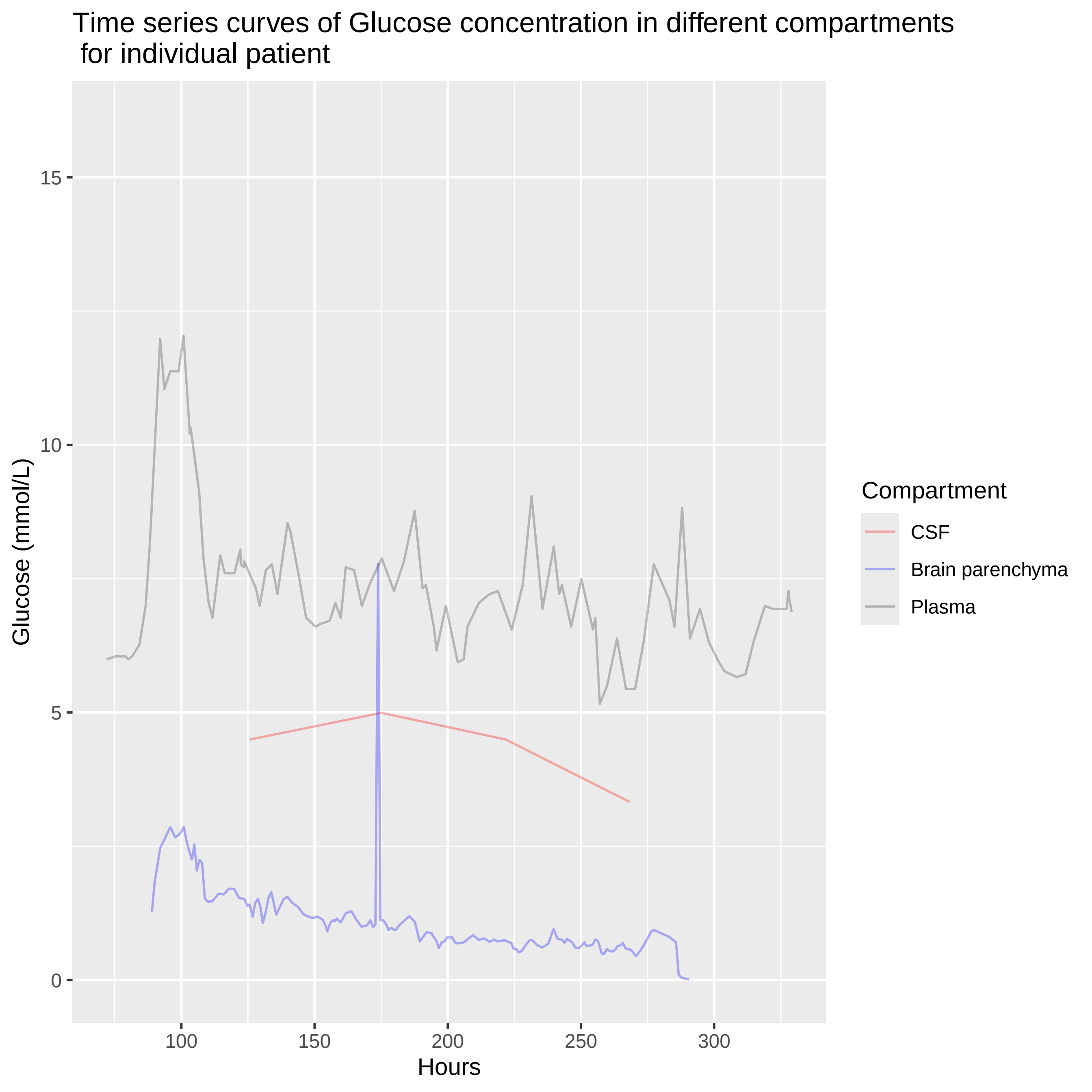
Time series curves of Glucose concentration in different compartments
for individual patient
Glucose (mmol/L)
15
10
5
0
100
150
200
250
300
Hours
Compartment
CSF
Brain parenchyma
Plasma

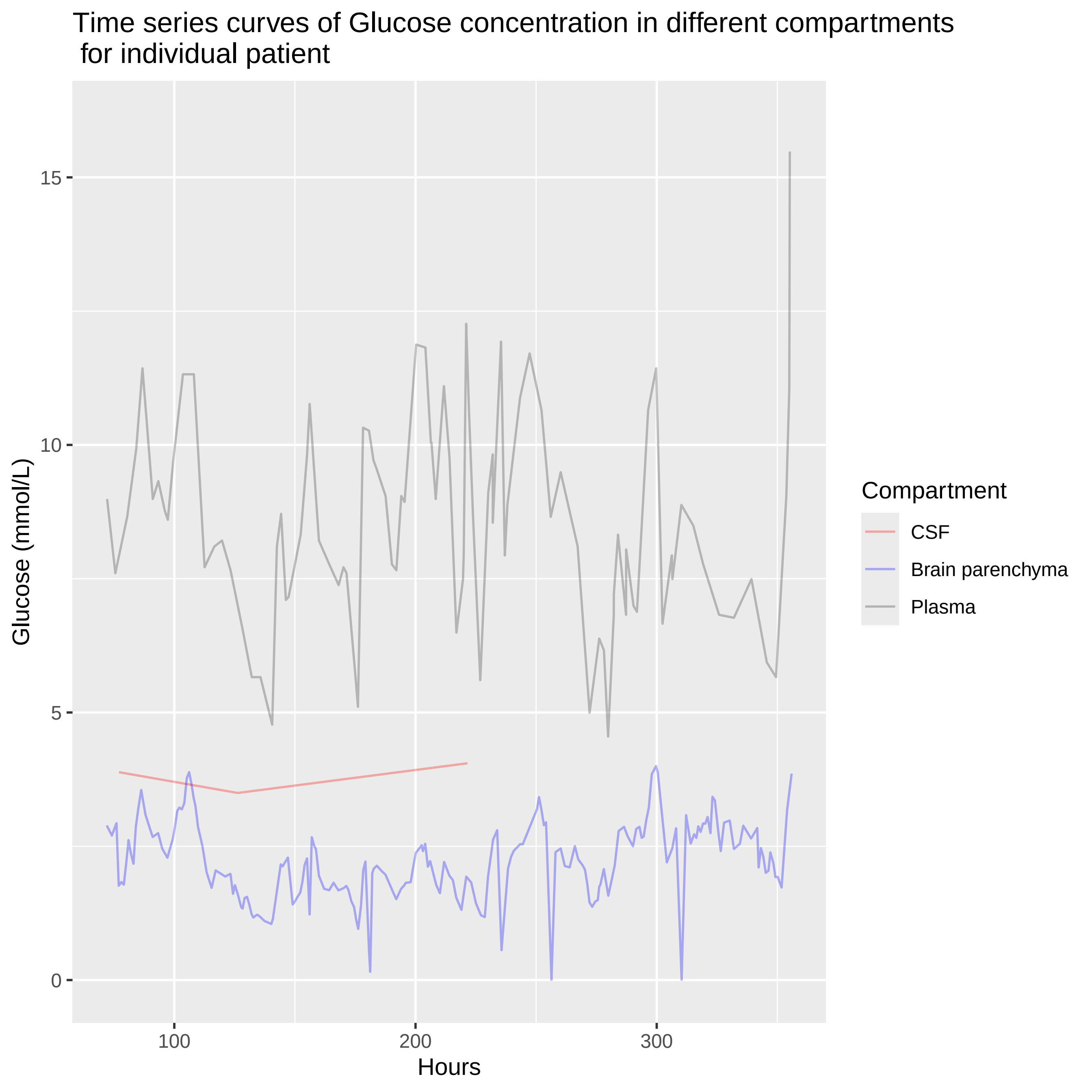

Time series curves of Glucose concentration in different compartments
for individual patient
Glucose (mmol/L)
15
10
5
0
100
200
300
Hours
Compartment
CSF
Brain parenchyma
Plasma

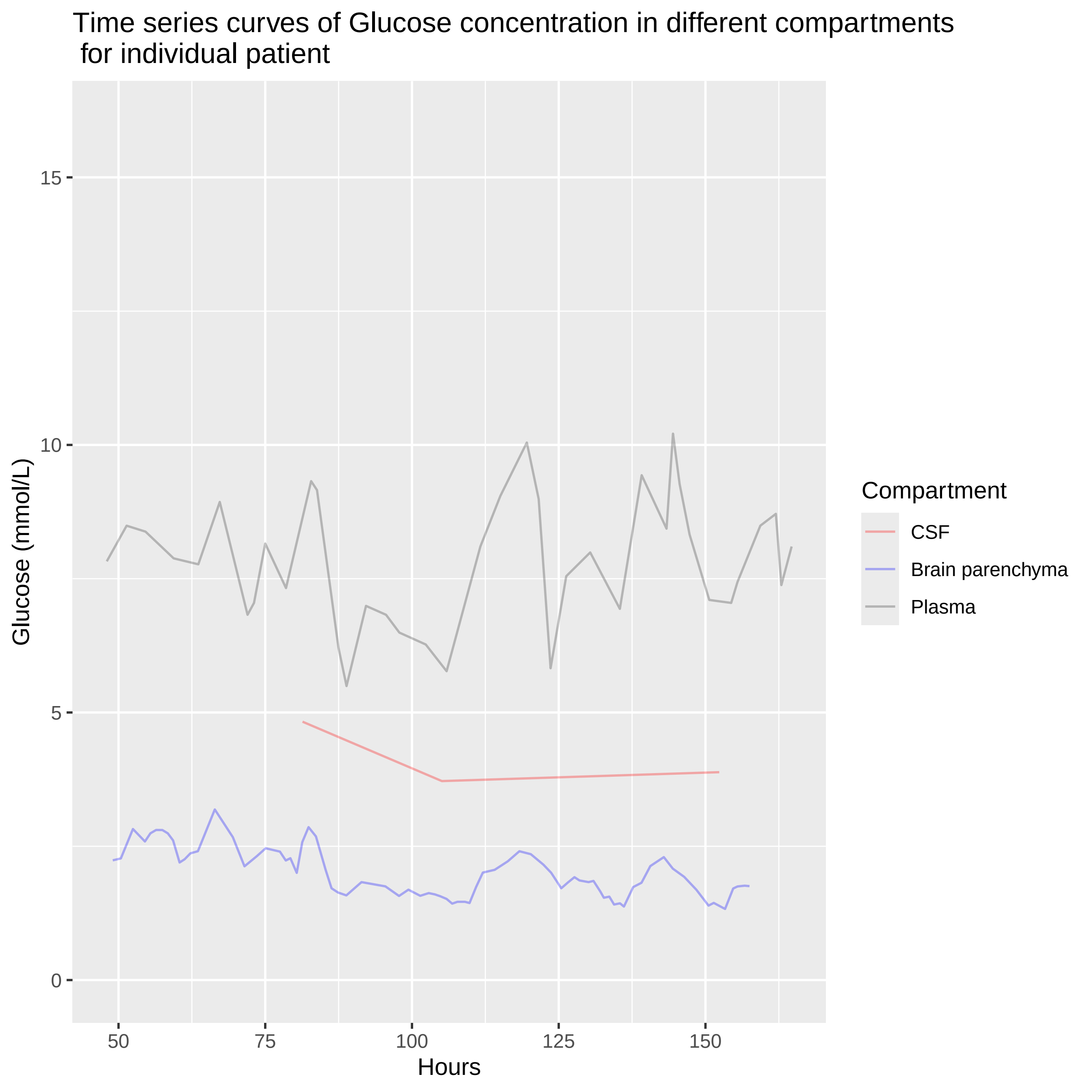

Time series curves of Glucose concentration in different compartments
for individual patient
15
10
5
0
Glucose (mmol/L)
50
75
100
125
150
Hours
Compartment
CSF
Brain parenchyma
Plasma

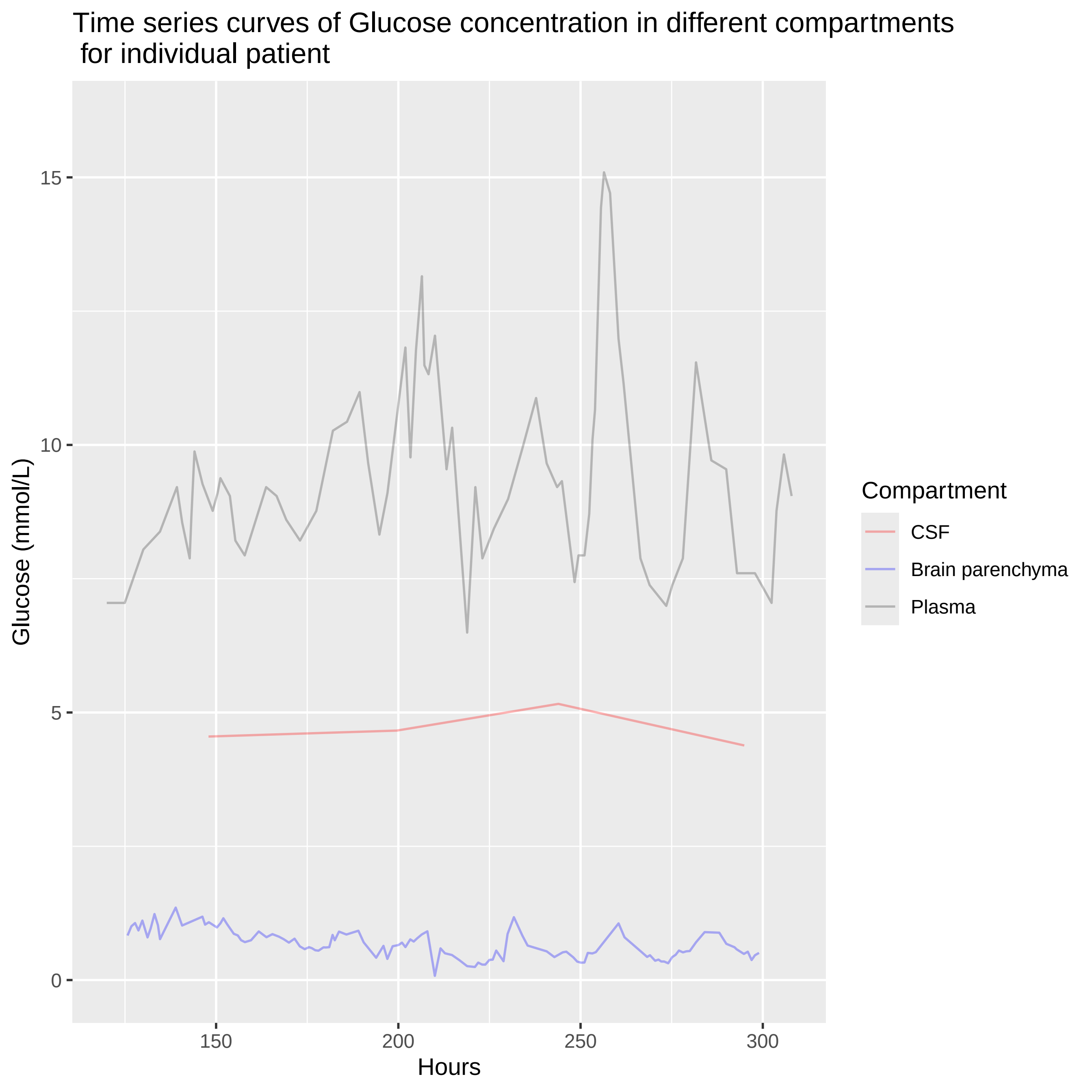
Time series curves of Glucose concentration in different compartments
for individual patient
Glucose (mmol/L)
15
10
5
0
150
200
250
300
Hours
Compartment
CSF
Brain parenchyma
Plasma

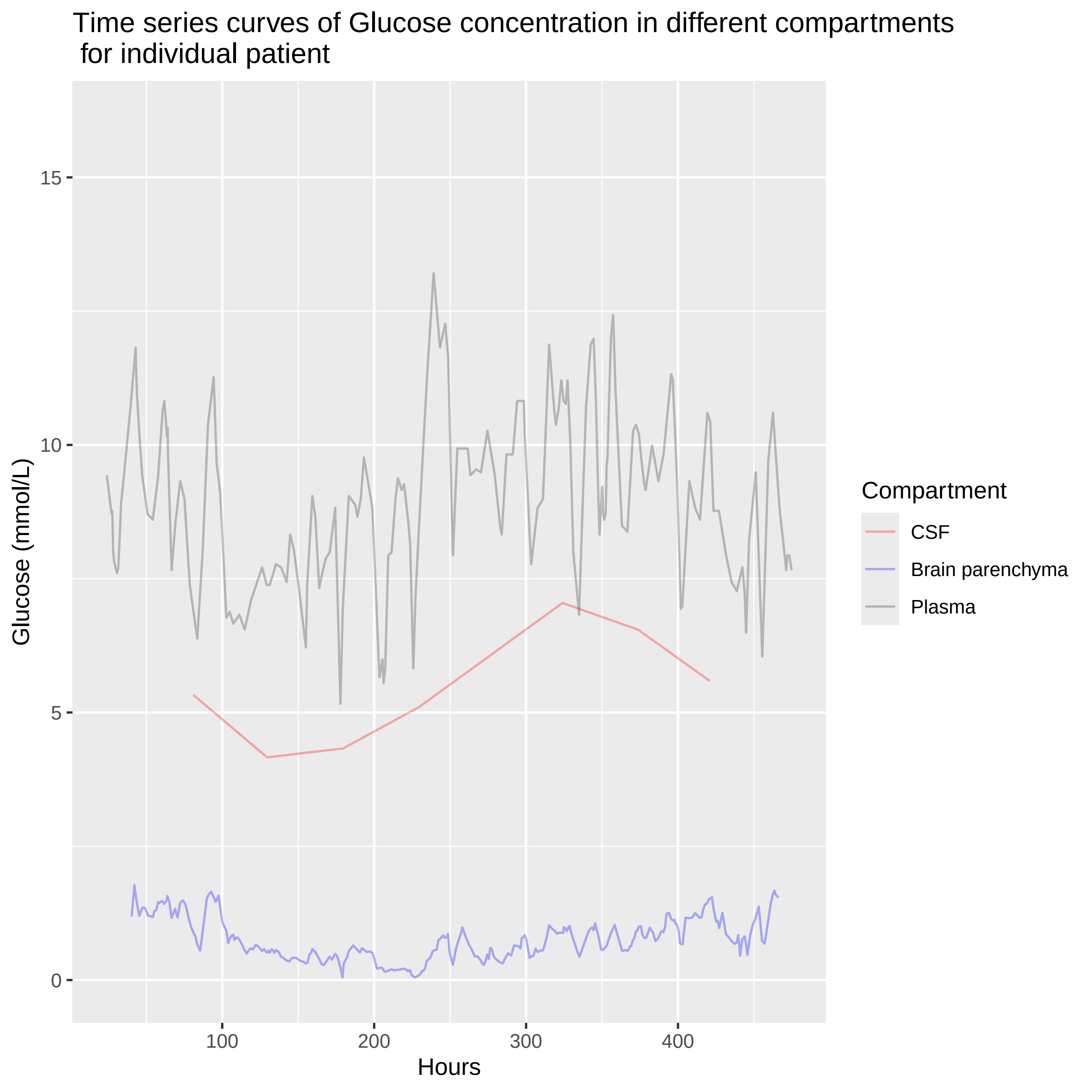
Time series curves of Glucose concentration in different compartments
for individual patient
Glucose (mmol/L)
15
10
5
0
100
200
300
400
Hours
Compartment
CSF
Brain parenchyma
Plasma

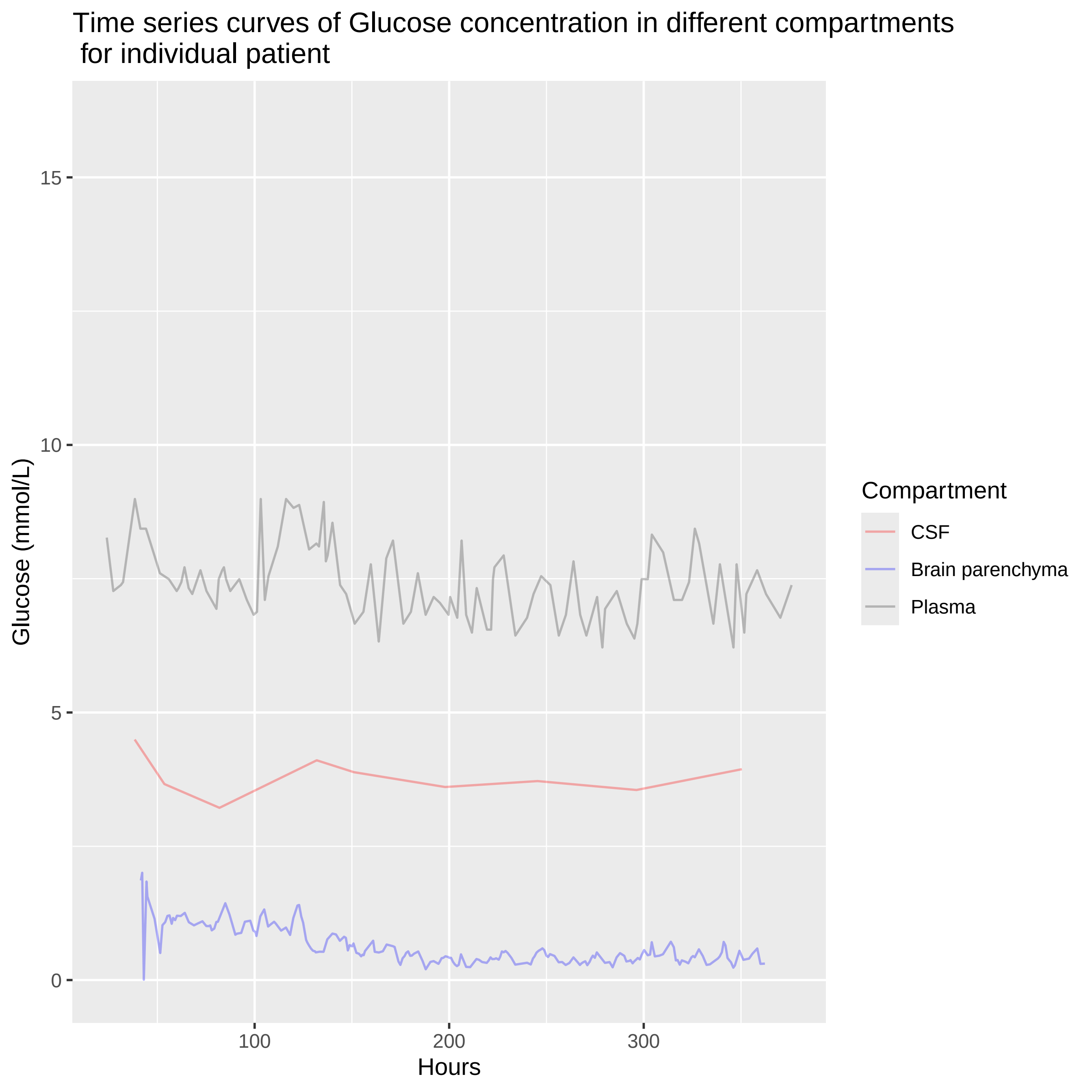
Time series curves of Glucose concentration in different compartments
for individual patient
Glucose (mmol/L)
15
10
5
0
100
200
300
Hours
Compartment
CSF
Brain parenchyma
Plasma

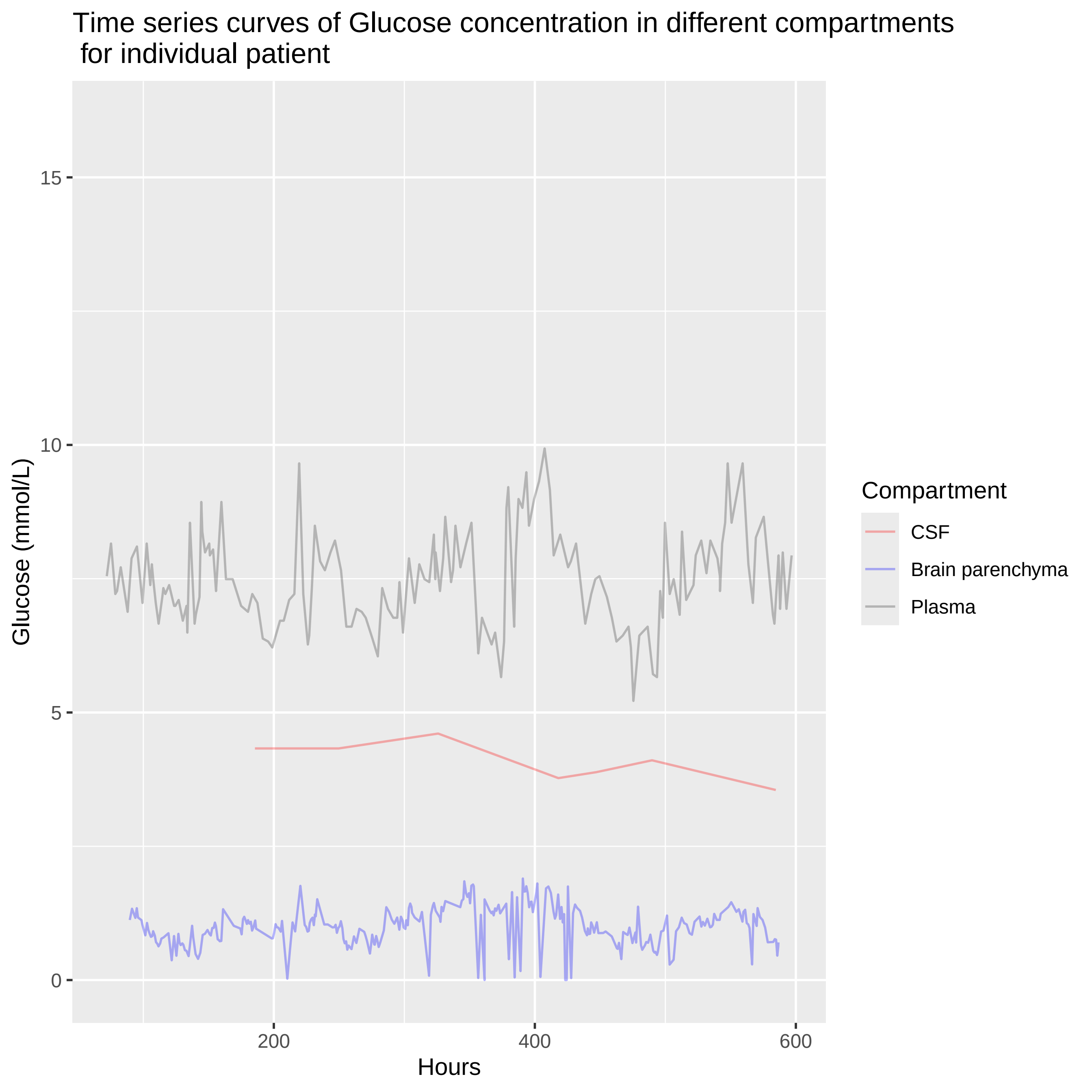
Time series curves of Glucose concentration in different compartments
for individual patient
Glucose (mmol/L)
15
10
5
0
200
400
600
Hours
Compartment
CSF
Brain parenchyma
Plasma

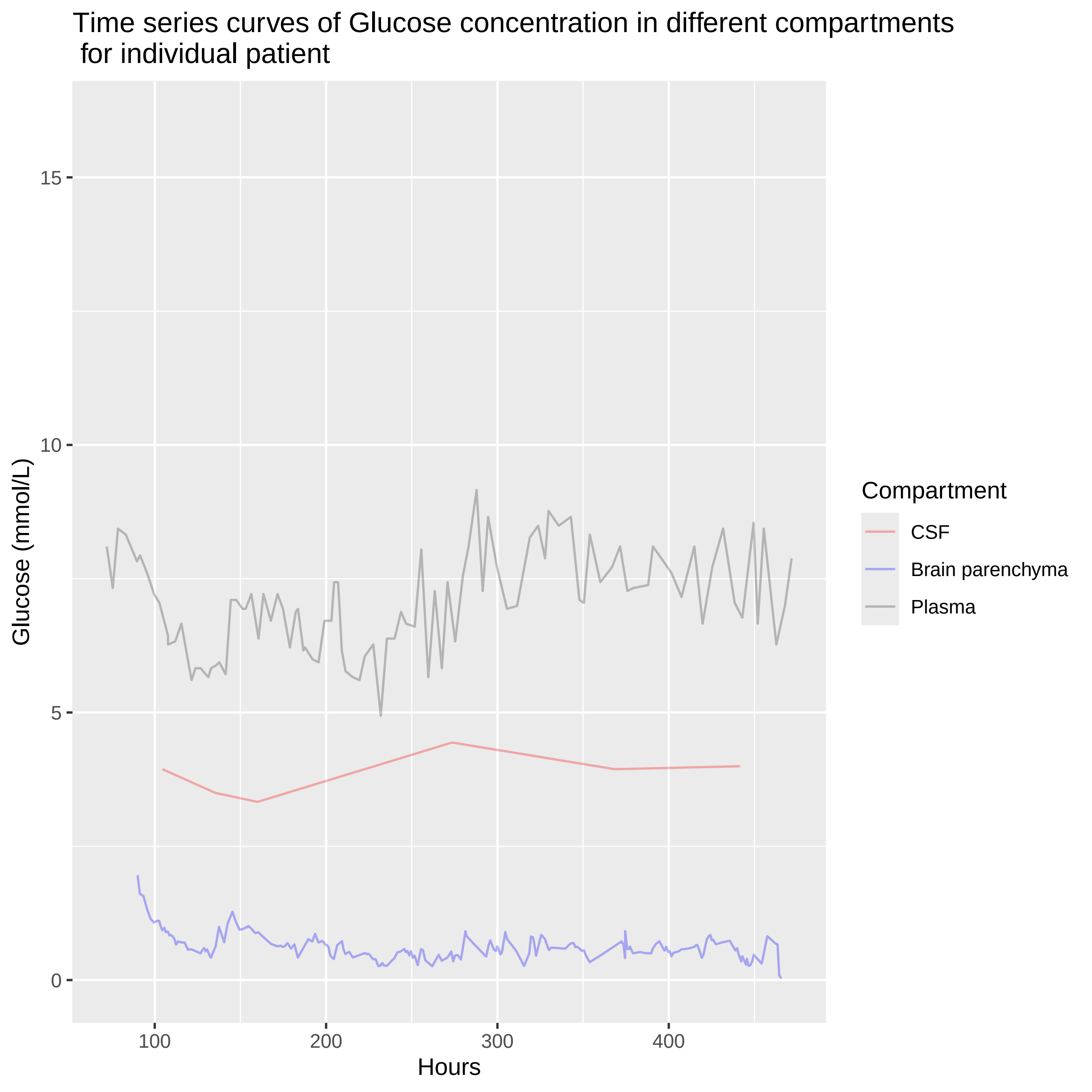
Time series curves of Glucose concentration in different compartments
for individual patient
Glucose (mmol/L)
Hours
15
10
5
0
100
200
300
400
Compartment
CSF
Brain parenchyma
Plasma

Time series curves of Glucose concentration in different compartments
for individual patient

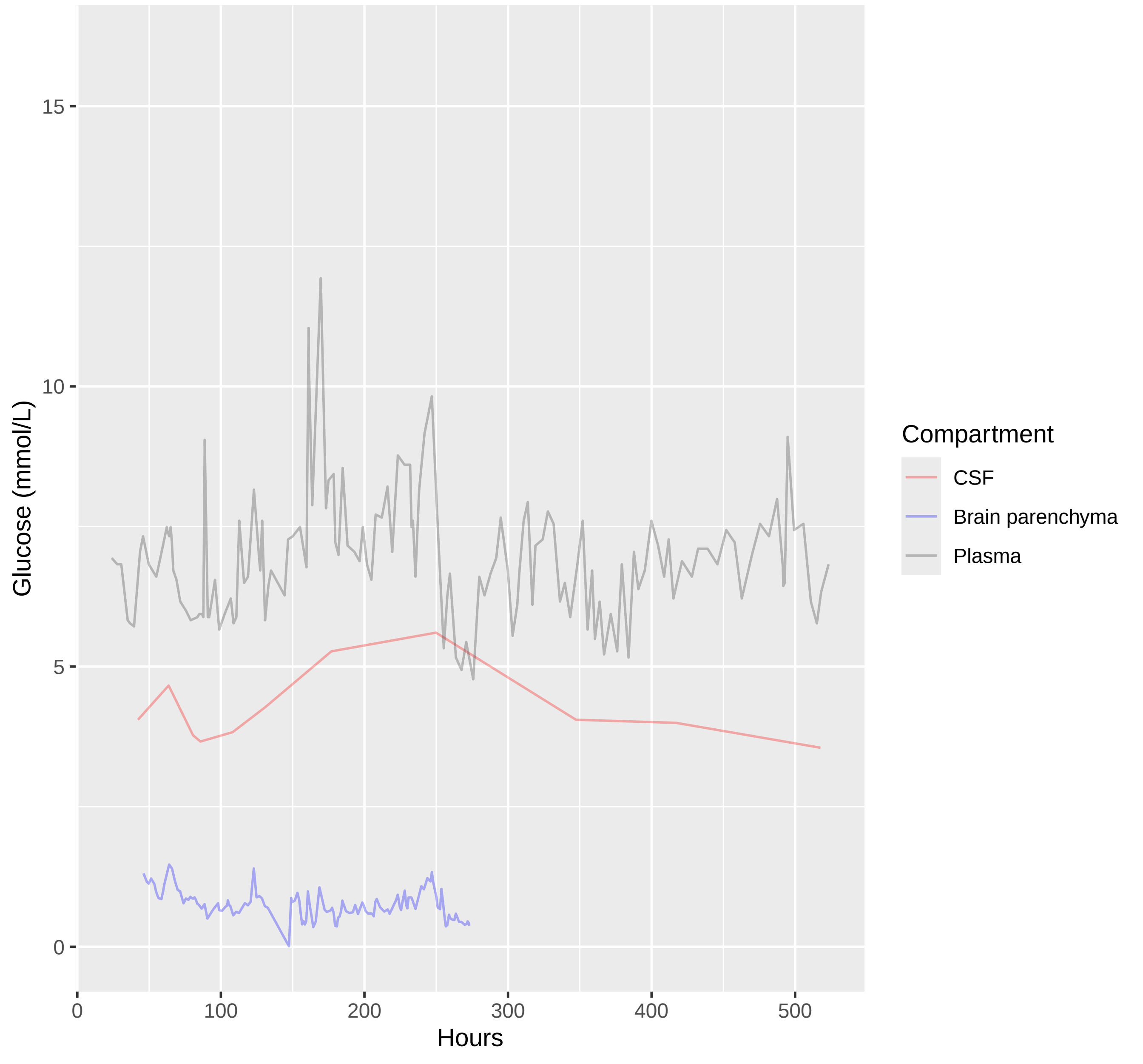

## 4. Trajectories of the Lactate Concentration in Different Compartments for Individual Patients

Values above 15mmol/L where cut off at 15 mmol/L in the following plots.

# Time series curves of lactate concentration in different compartments for individual patient

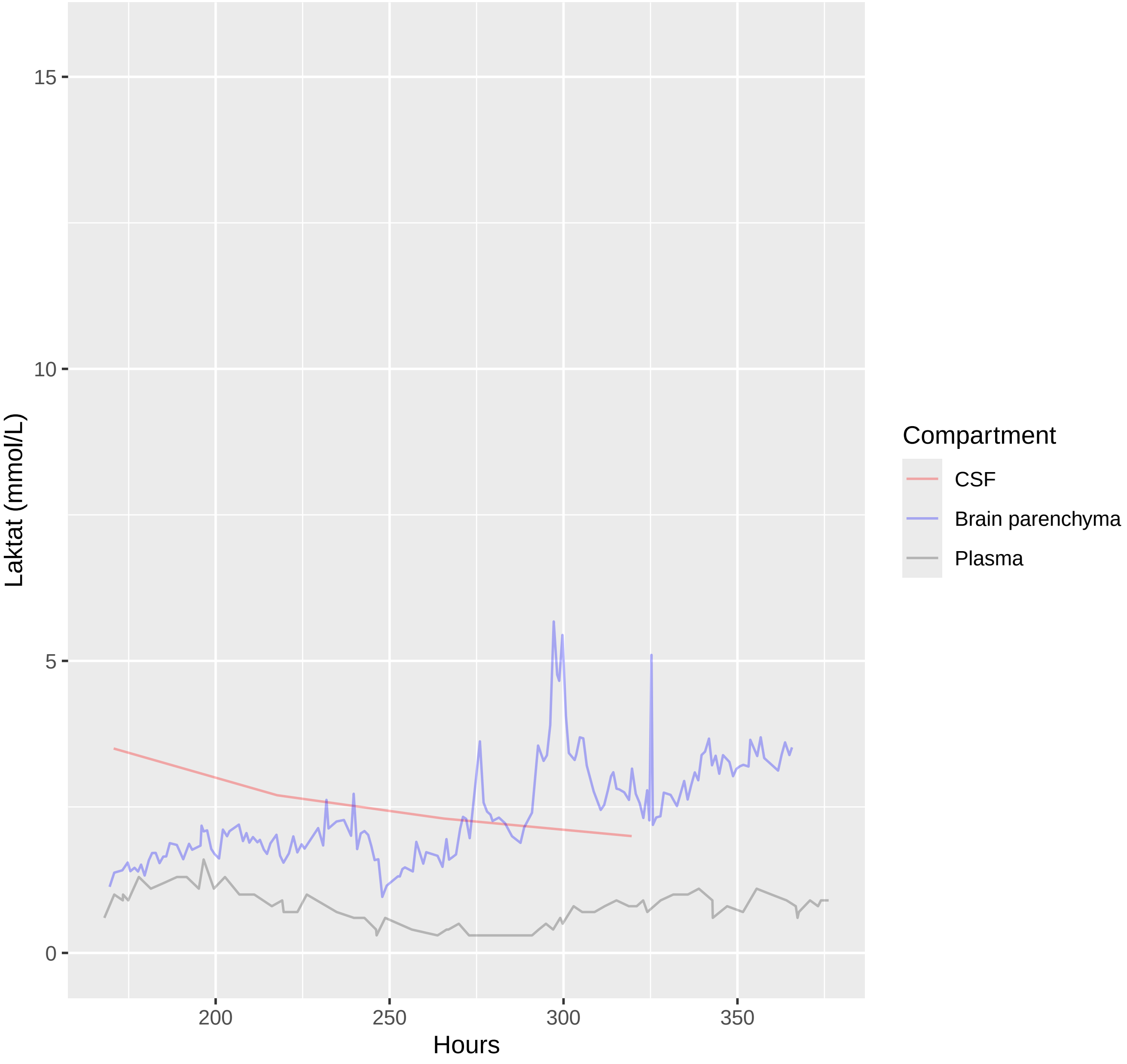

Time series curves of lactate concentration in different compartments
for individual patient

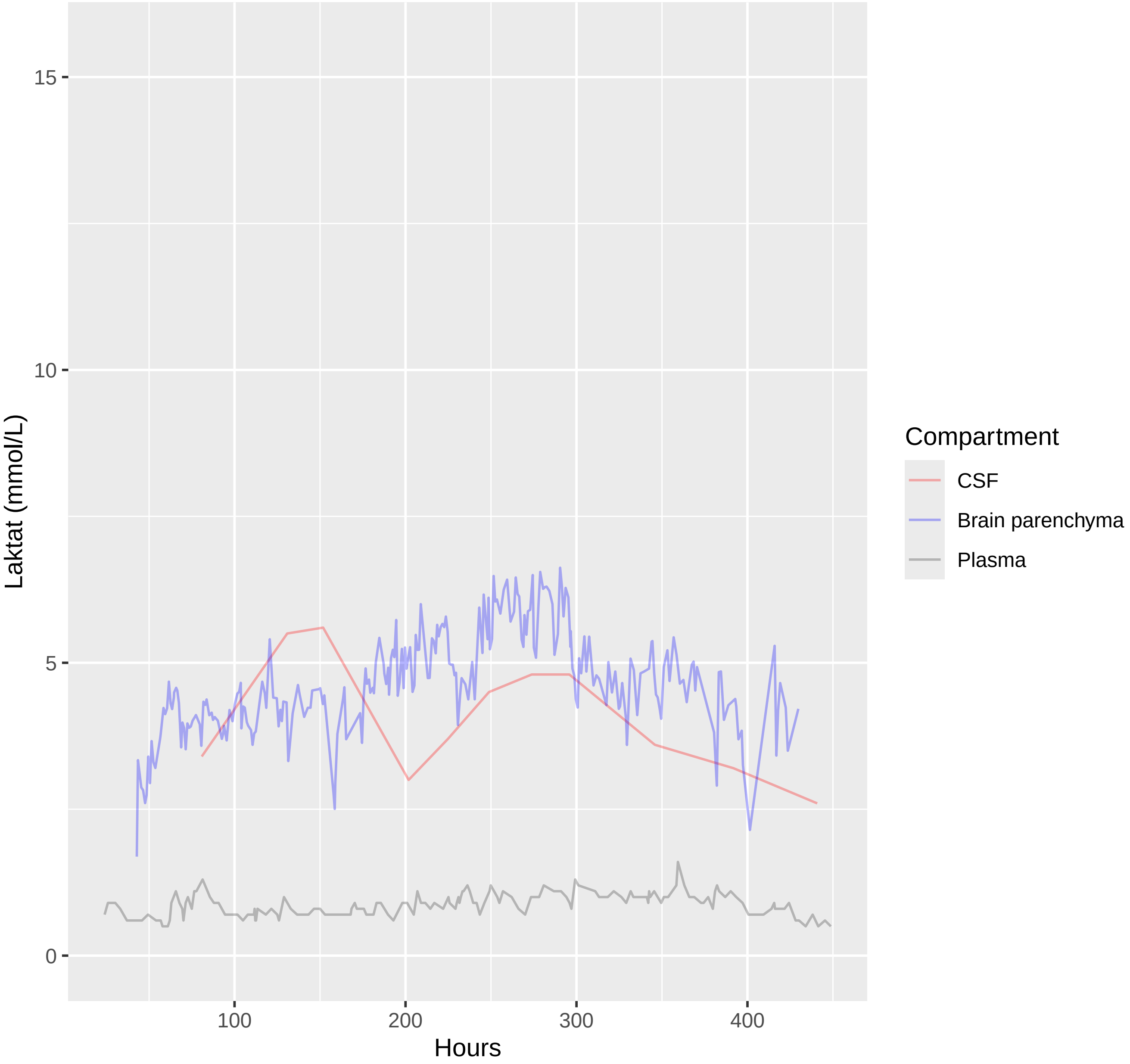

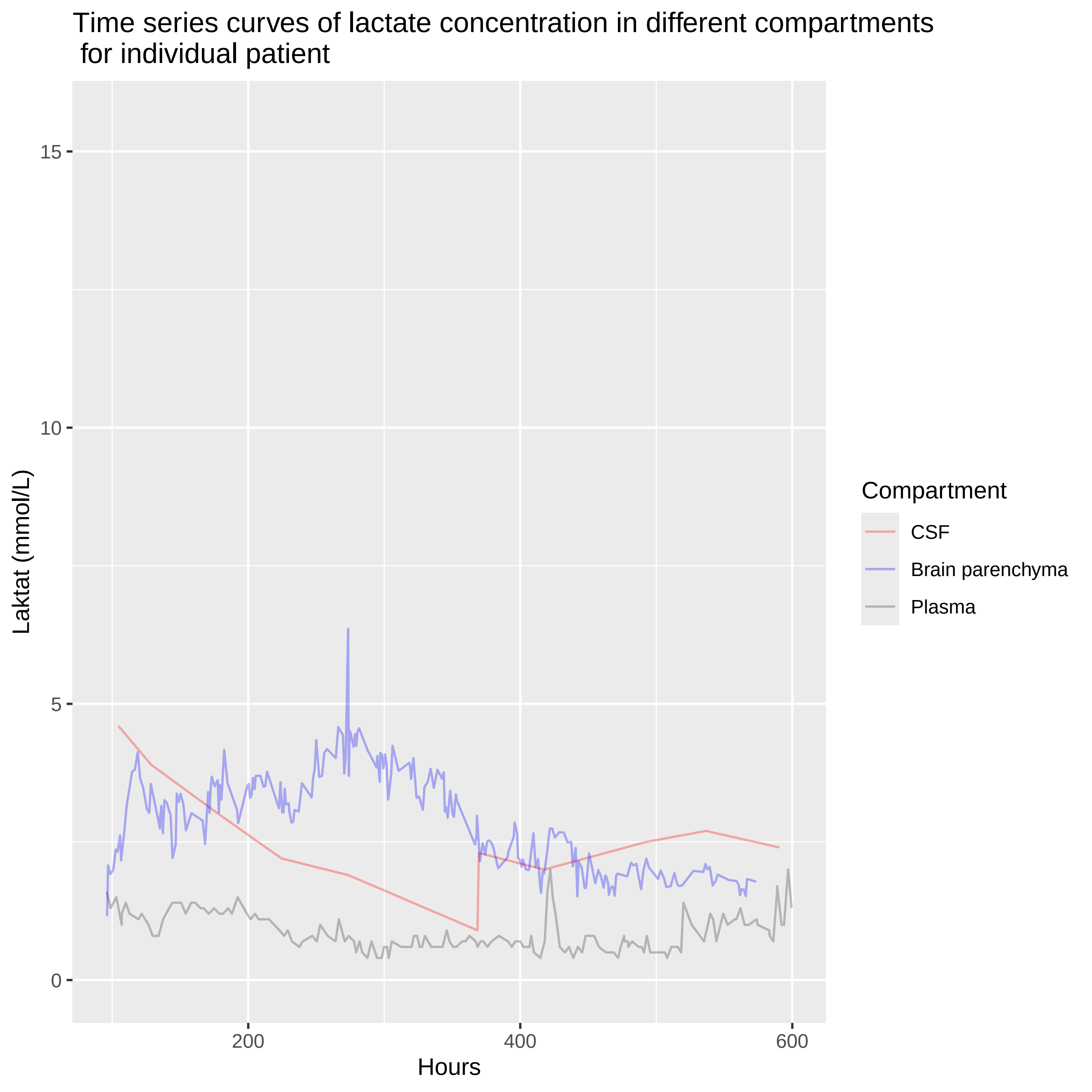
Time series curves of lactate concentration in different compartments
for individual patient
Laktat (mmol/L)
15
10
5
0
200
400
600
Hours
Compartment
CSF
Brain parenchyma
Plasma

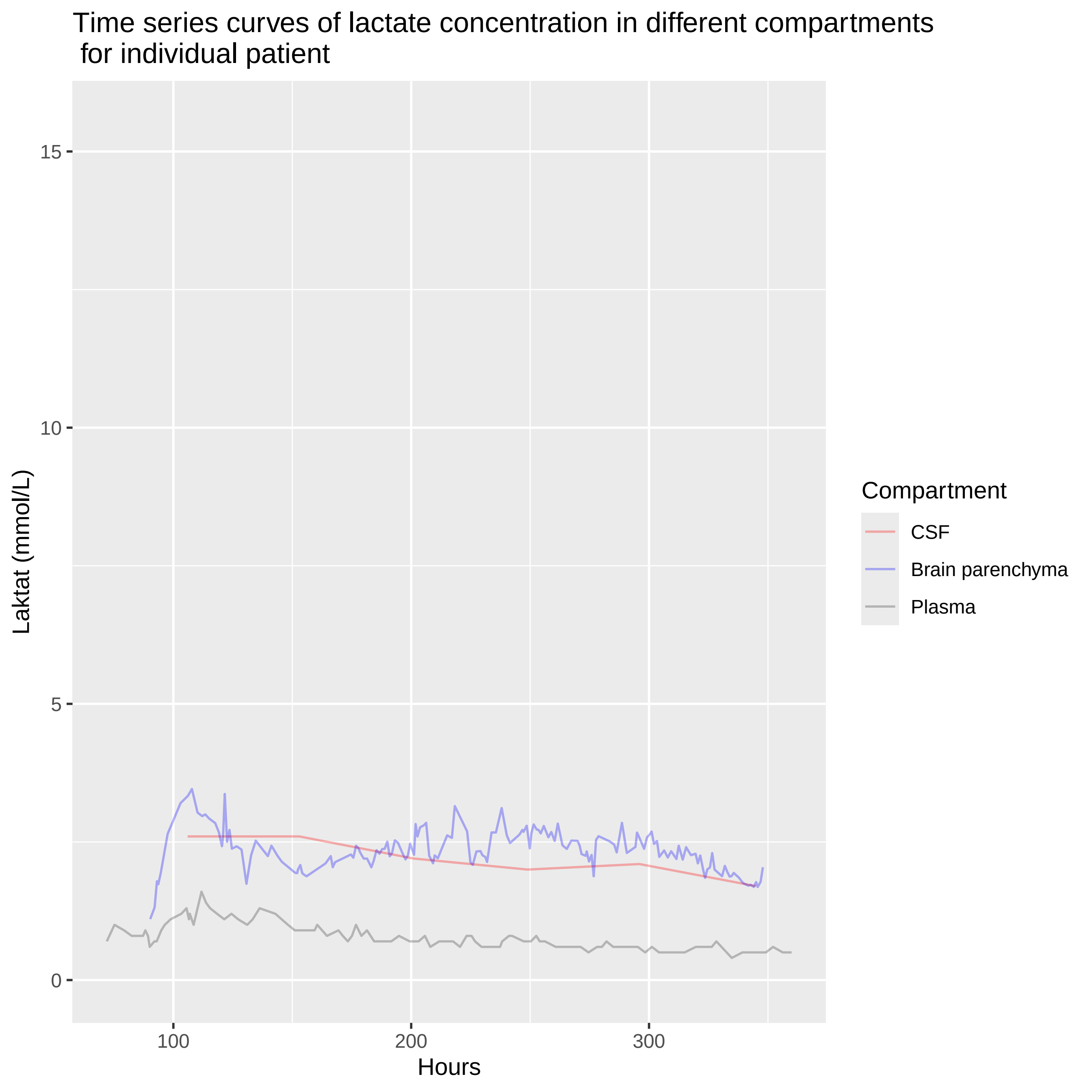

Time series curves of lactate concentration in different compartments
for individual patient
Laktat (mmol/L)
15
10
5
0
100
200
300
Hours
Compartment
CSF
Brain parenchyma
Plasma

Time series curves of lactate concentration in different compartments
 for individual patient

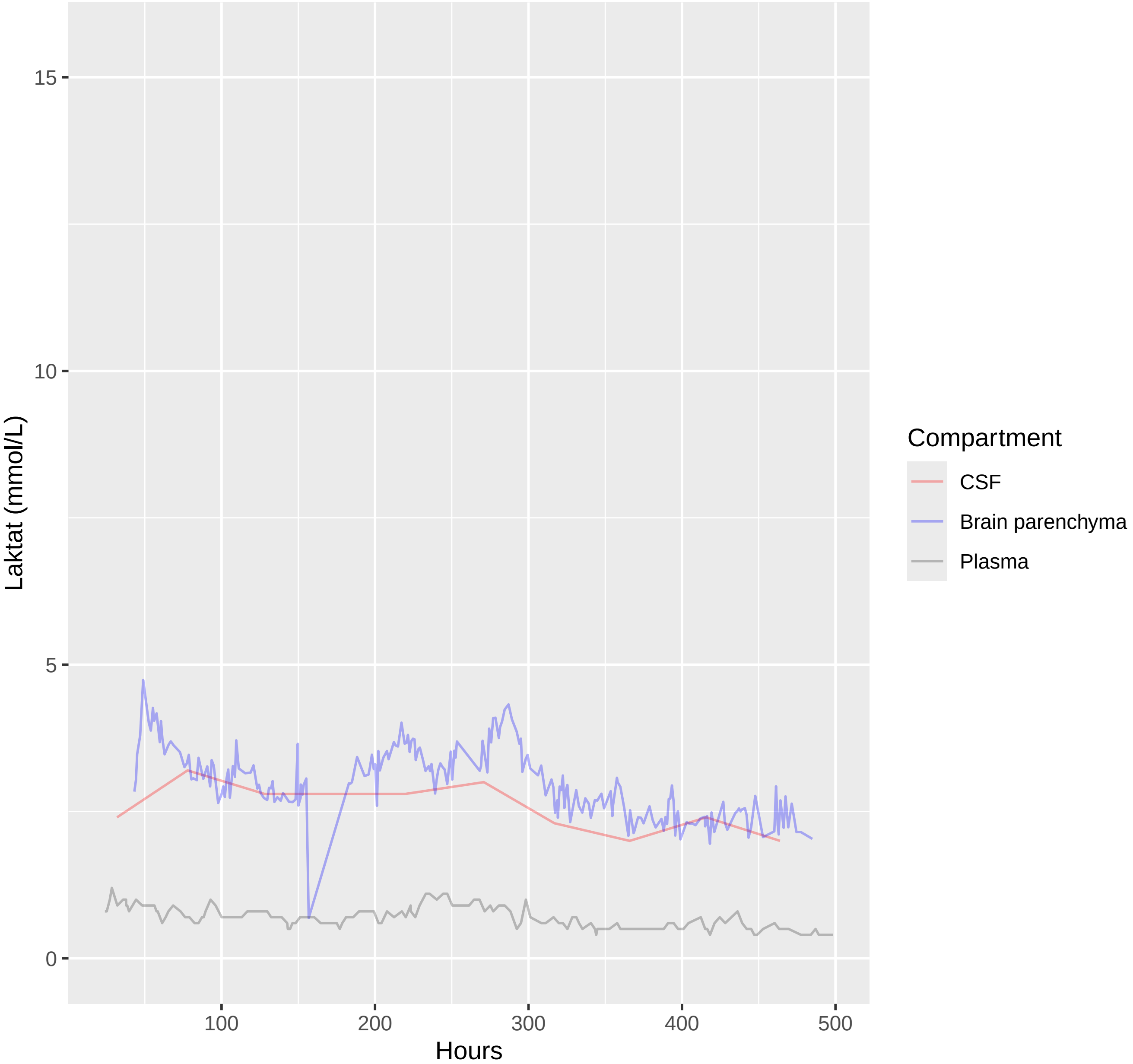

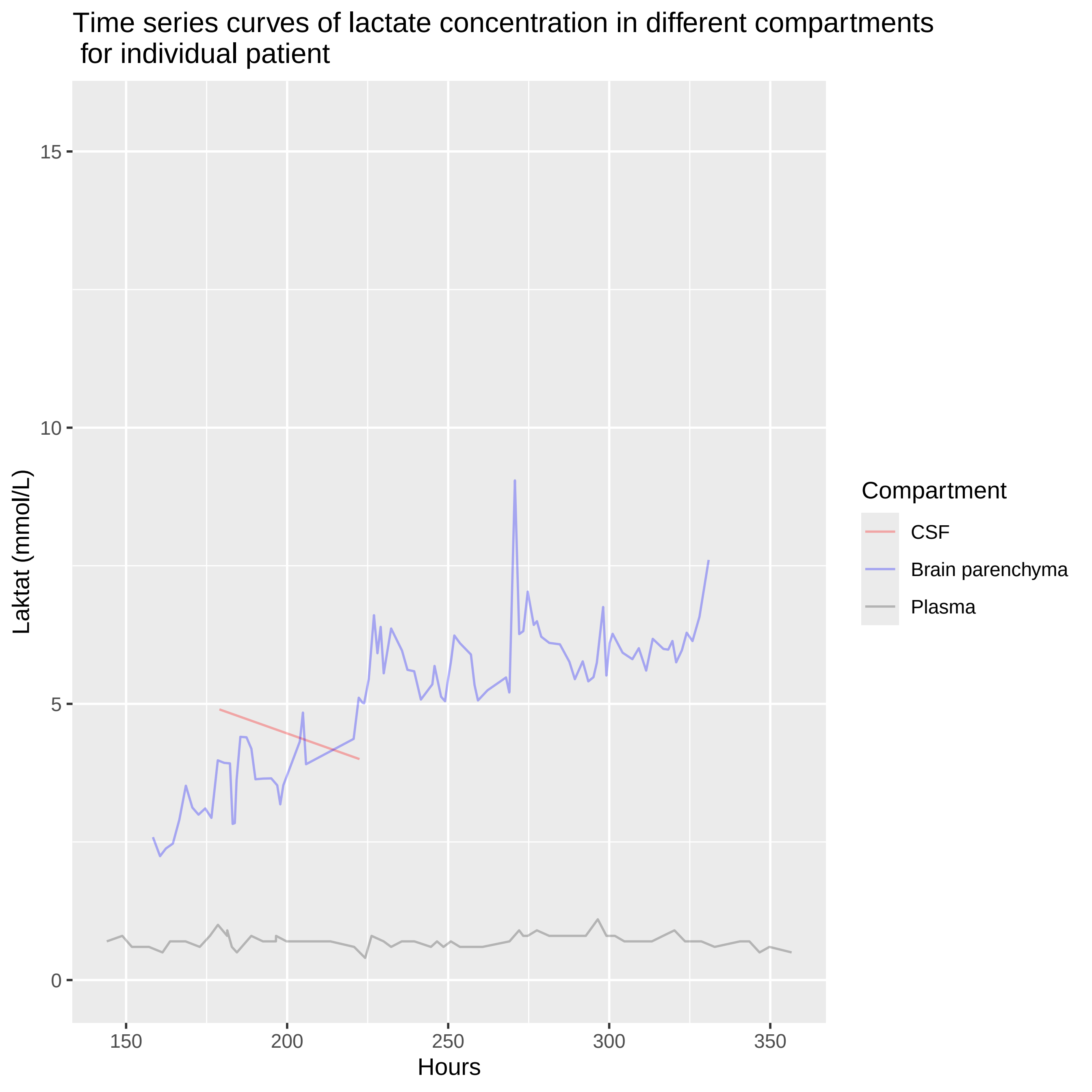

Time series curves of lactate concentration in different compartments
for individual patient
Laktat (mmol/L)
15
10
5
0
150
200
250
300
350
Hours
Compartment
CSF
Brain parenchyma
Plasma

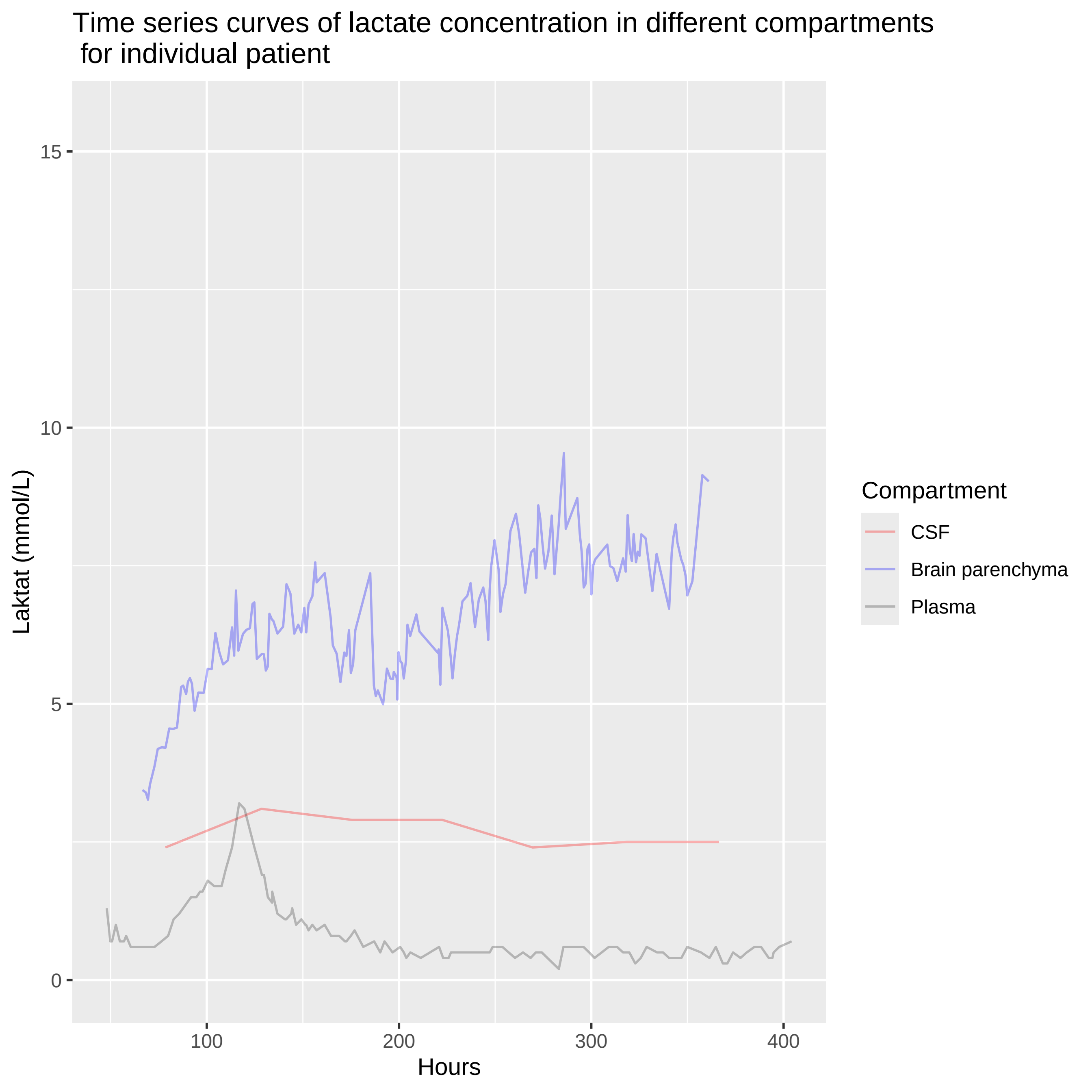

Time series curves of lactate concentration in different compartments
for individual patient
Laktat (mmol/L)
15
10
5
0
100
200
300
400
Hours
Compartment
CSF
Brain parenchyma
Plasma

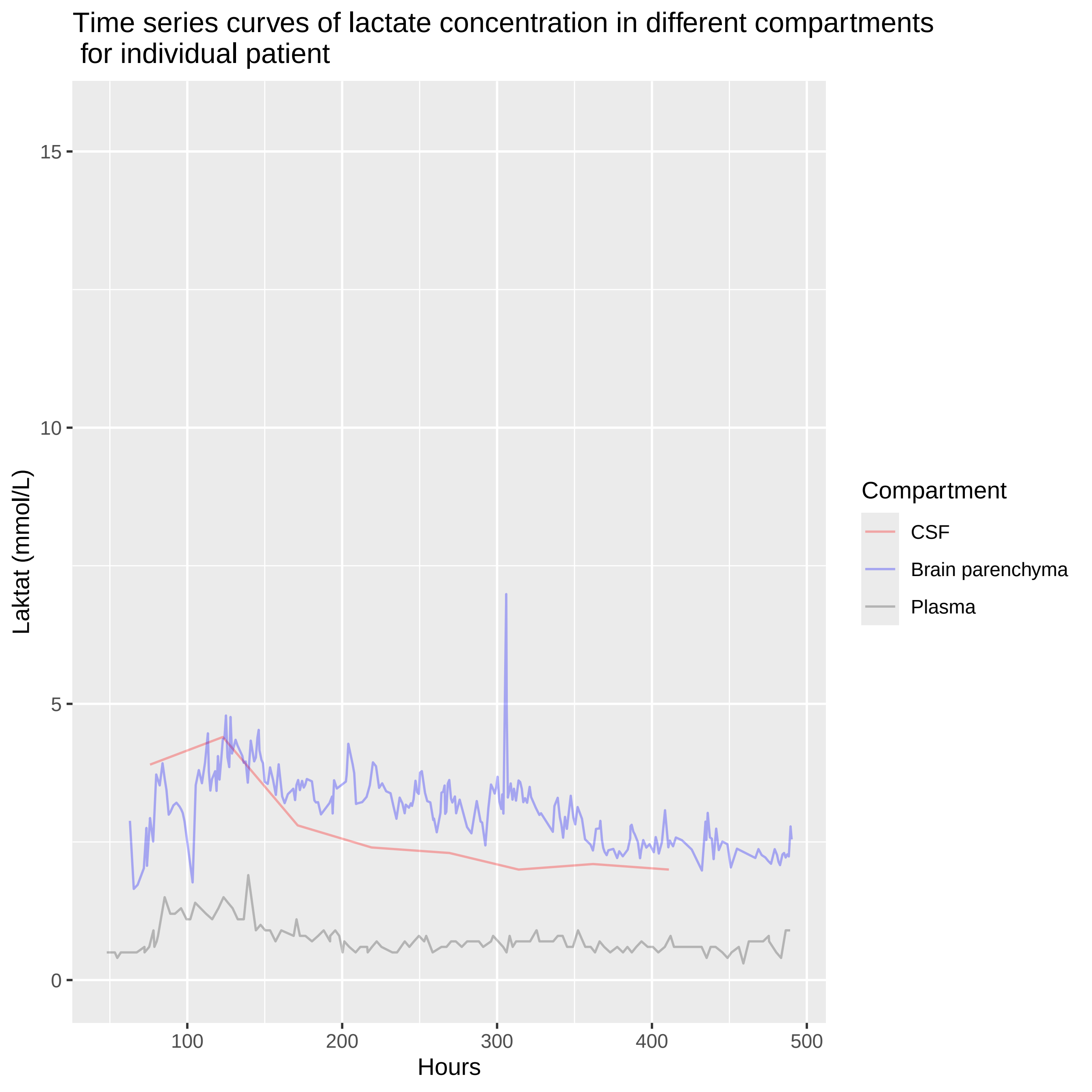

Time series curves of lactate concentration in different compartments
for individual patient
Laktat (mmol/L)
15
10
5
0
100
200
300
400
500
Hours
Compartment
CSF
Brain parenchyma
Plasma

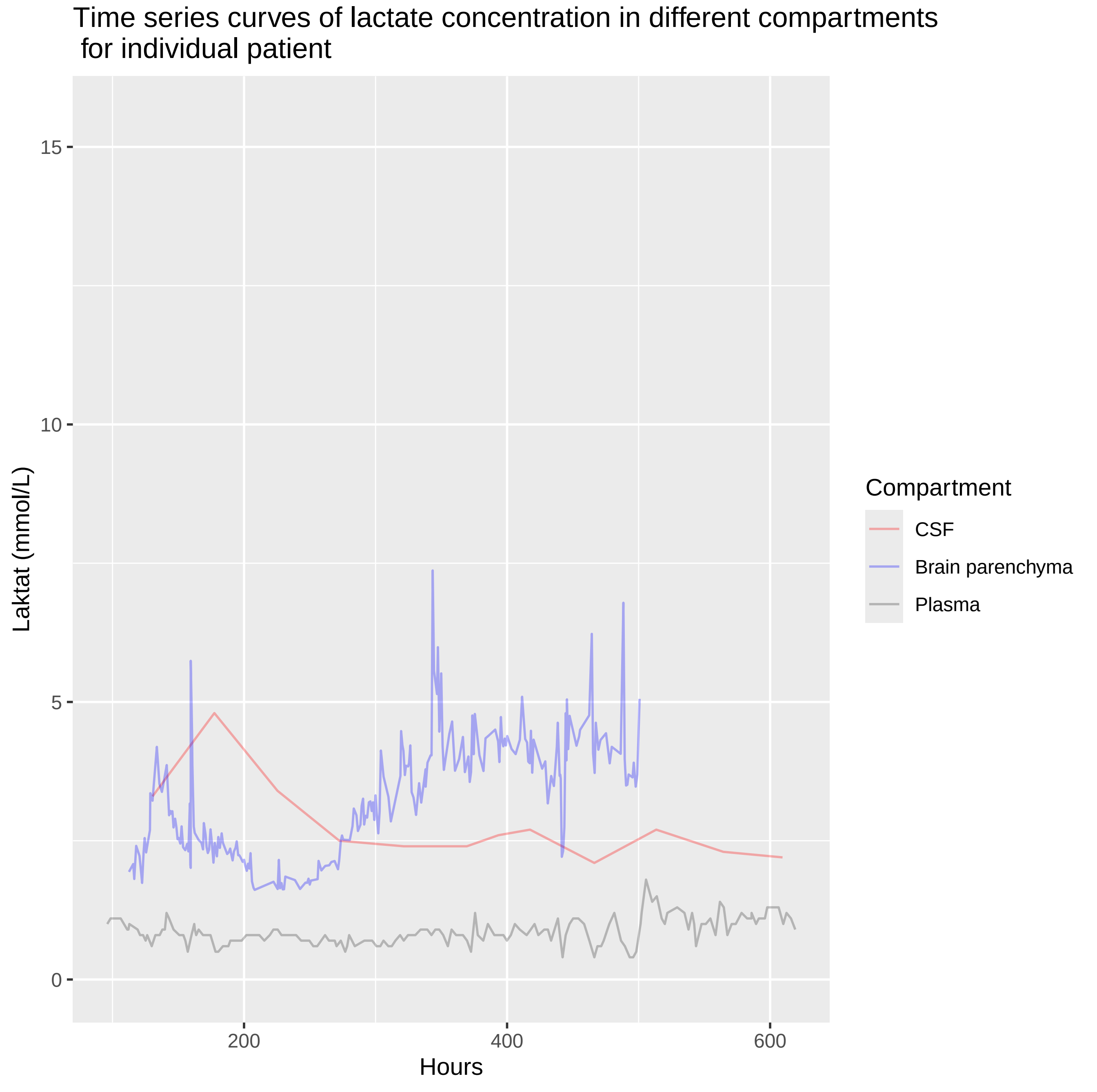

Time series curves of lactate concentration in different compartments
for individual patient
Laktat (mmol/L)
Hours
0
5
10
15
200
400
600
Compartment
CSF
Brain parenchyma
Plasma

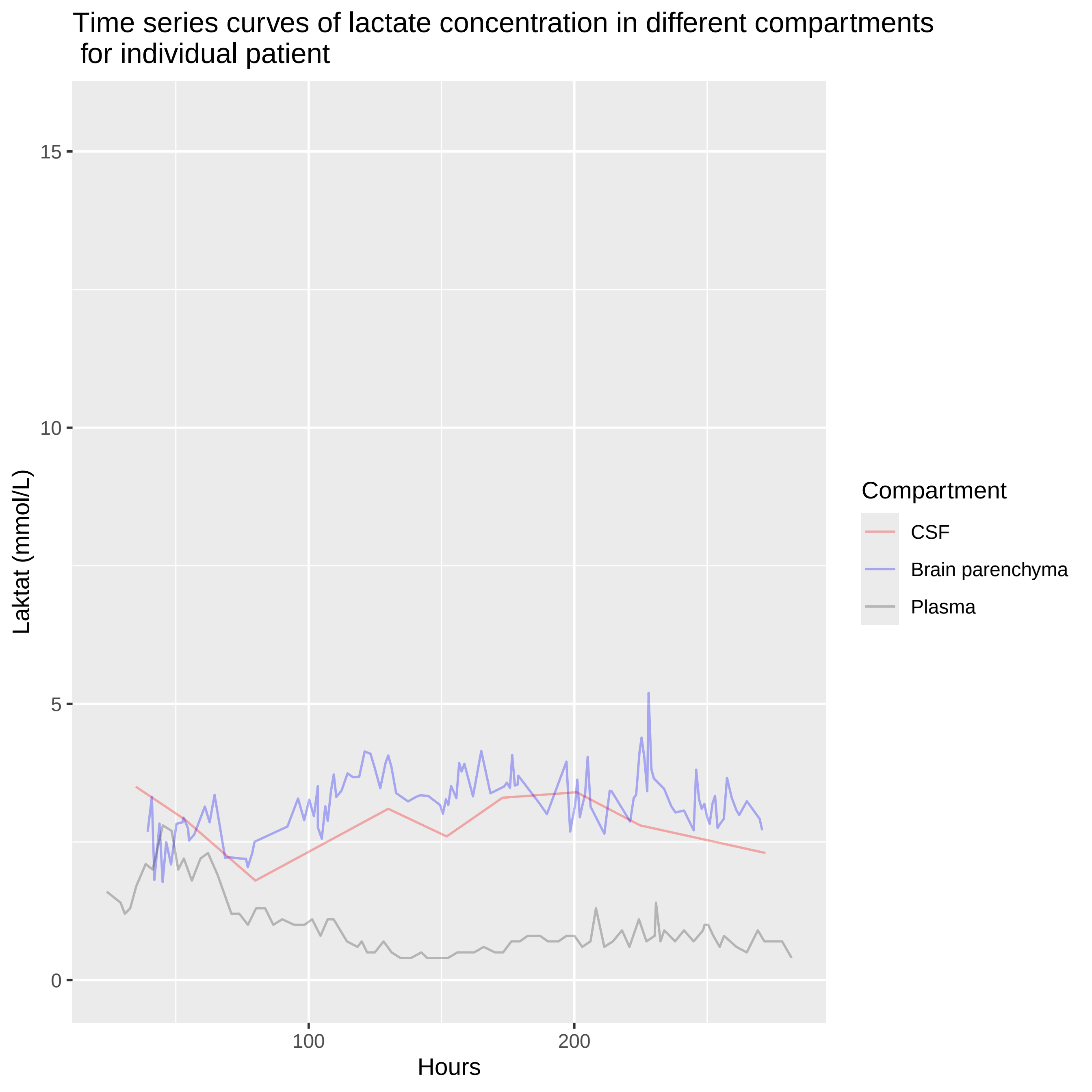

Time series curves of lactate concentration in different compartments
for individual patient
Laktat (mmol/L)
15
10
5
0
100
200
Hours
Compartment
CSF
Brain parenchyma
Plasma

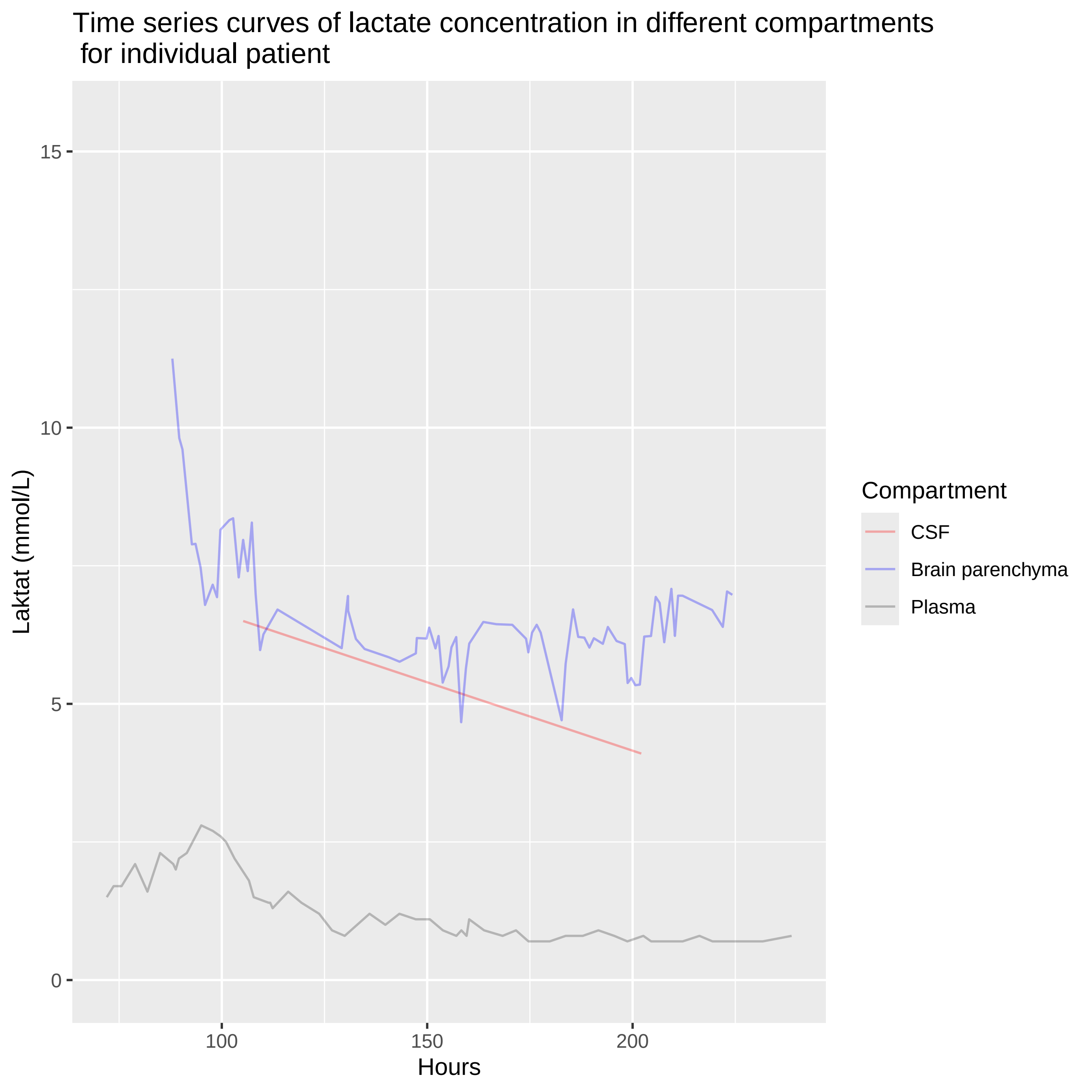
Time series curves of lactate concentration in different compartments
for individual patient
Laktat (mmol/L)
Hours
0
5
10
15
100
150
200
Compartment
CSF
Brain parenchyma
Plasma

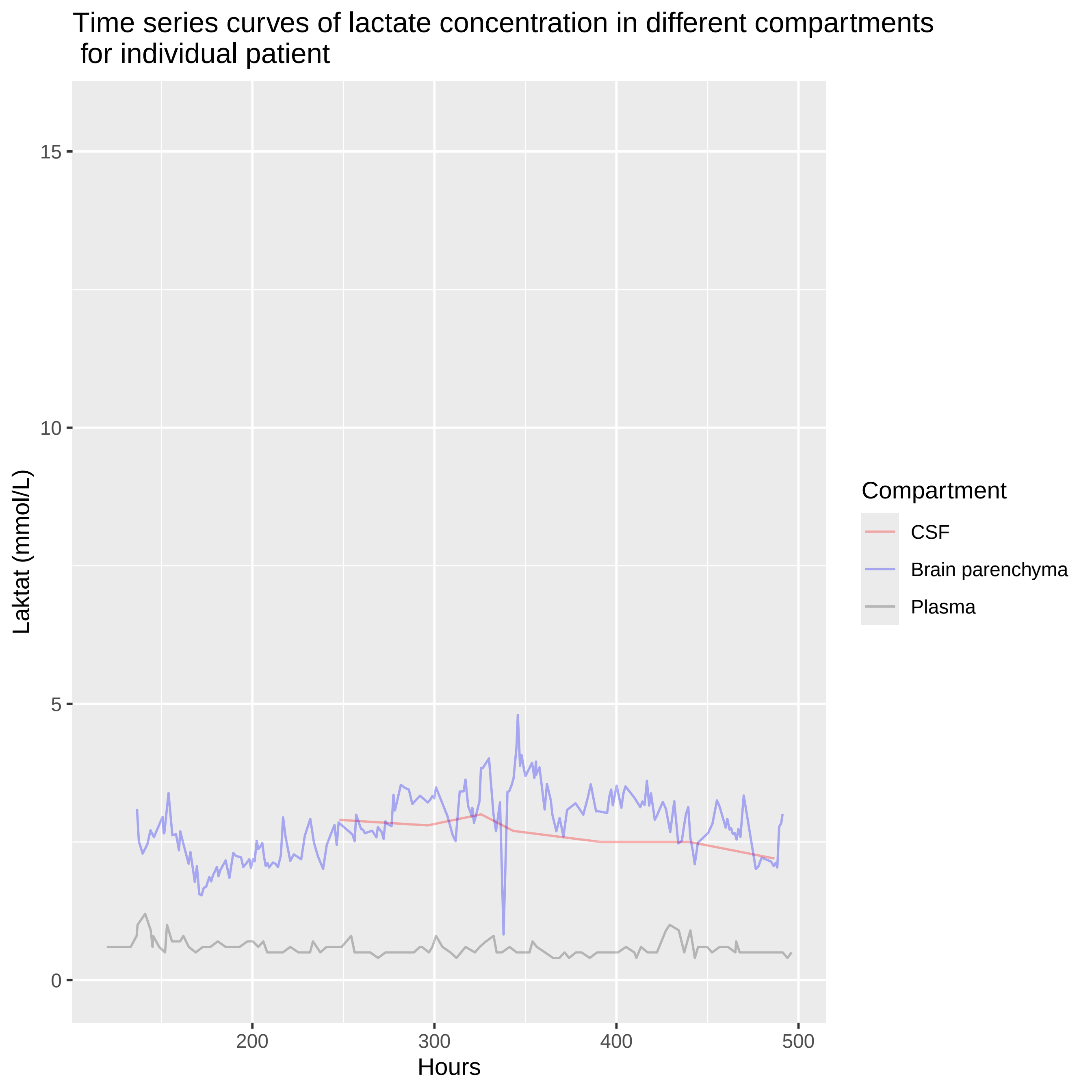
Time series curves of lactate concentration in different compartments
for individual patient
Laktat (mmol/L)
15
10
5
0
200
300
400
500
Hours
Compartment
CSF
Brain parenchyma
Plasma

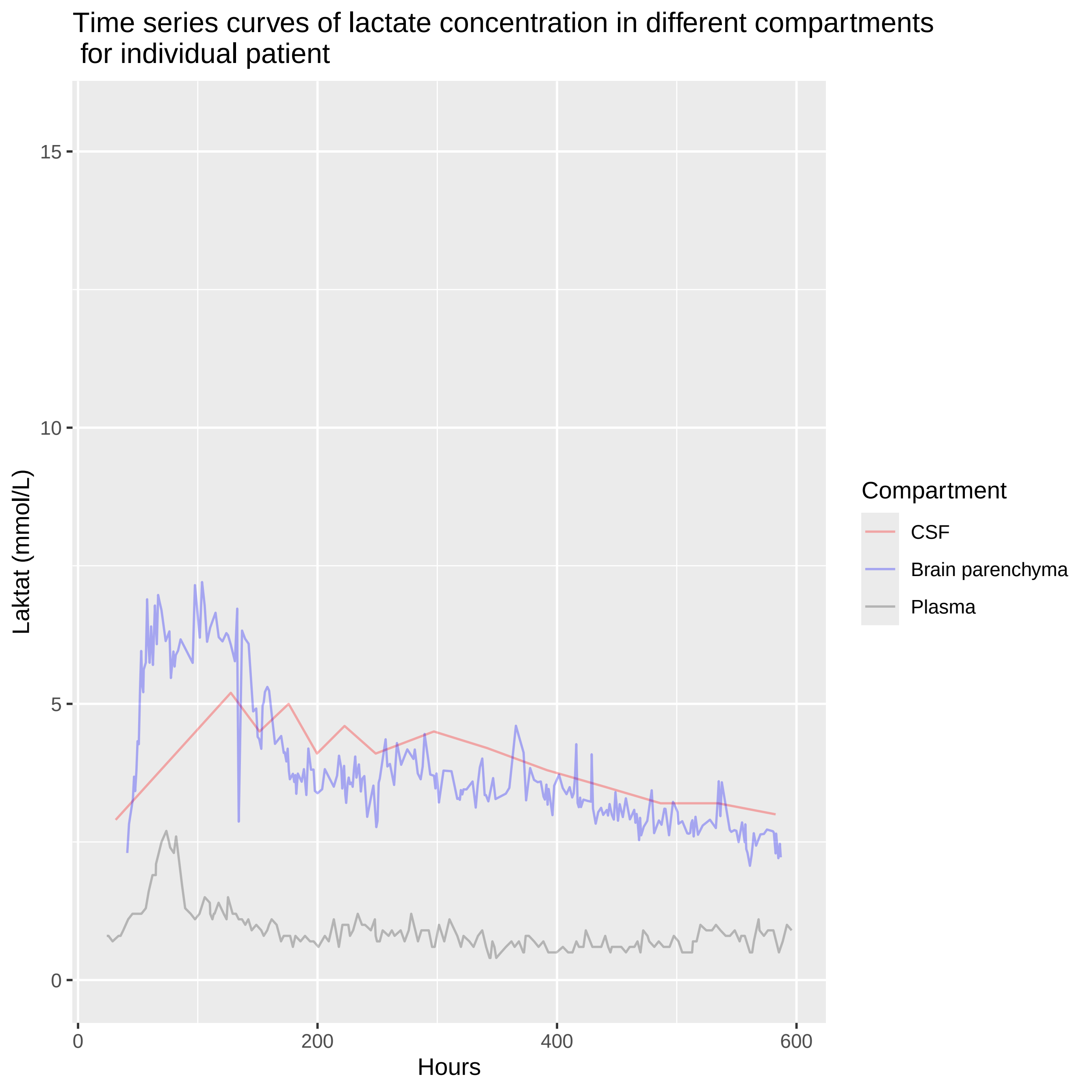
Time series curves of lactate concentration in different compartments
for individual patient
Laktat (mmol/L)
Hours
0
5
10
15
0
200
400
600
Compartment
CSF
Brain parenchyma
Plasma

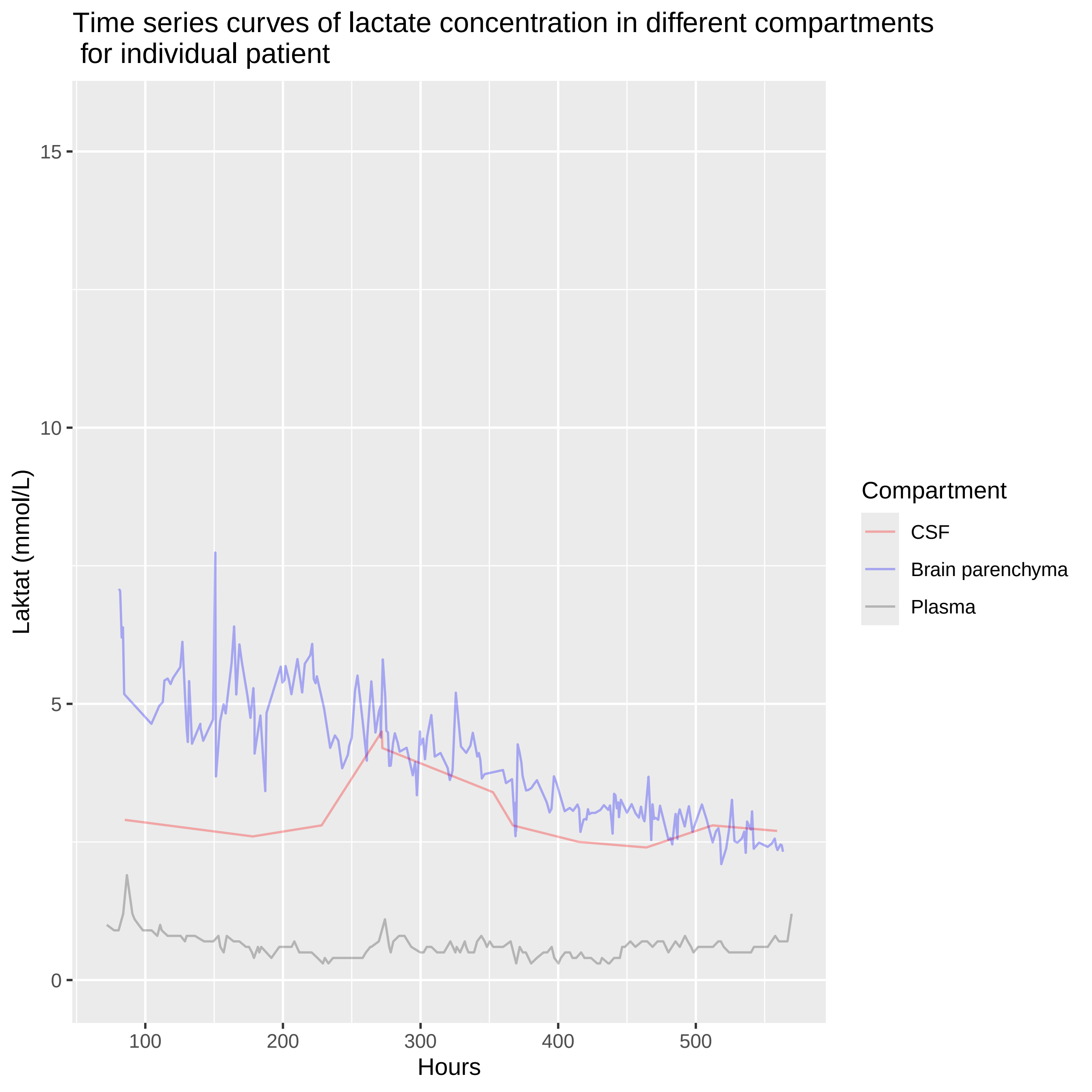

Time series curves of lactate concentration in different compartments
for individual patient
Laktat (mmol/L)
Hours
0
5
10
15
100
200
300
400
500
Compartment
CSF
Brain parenchyma
Plasma

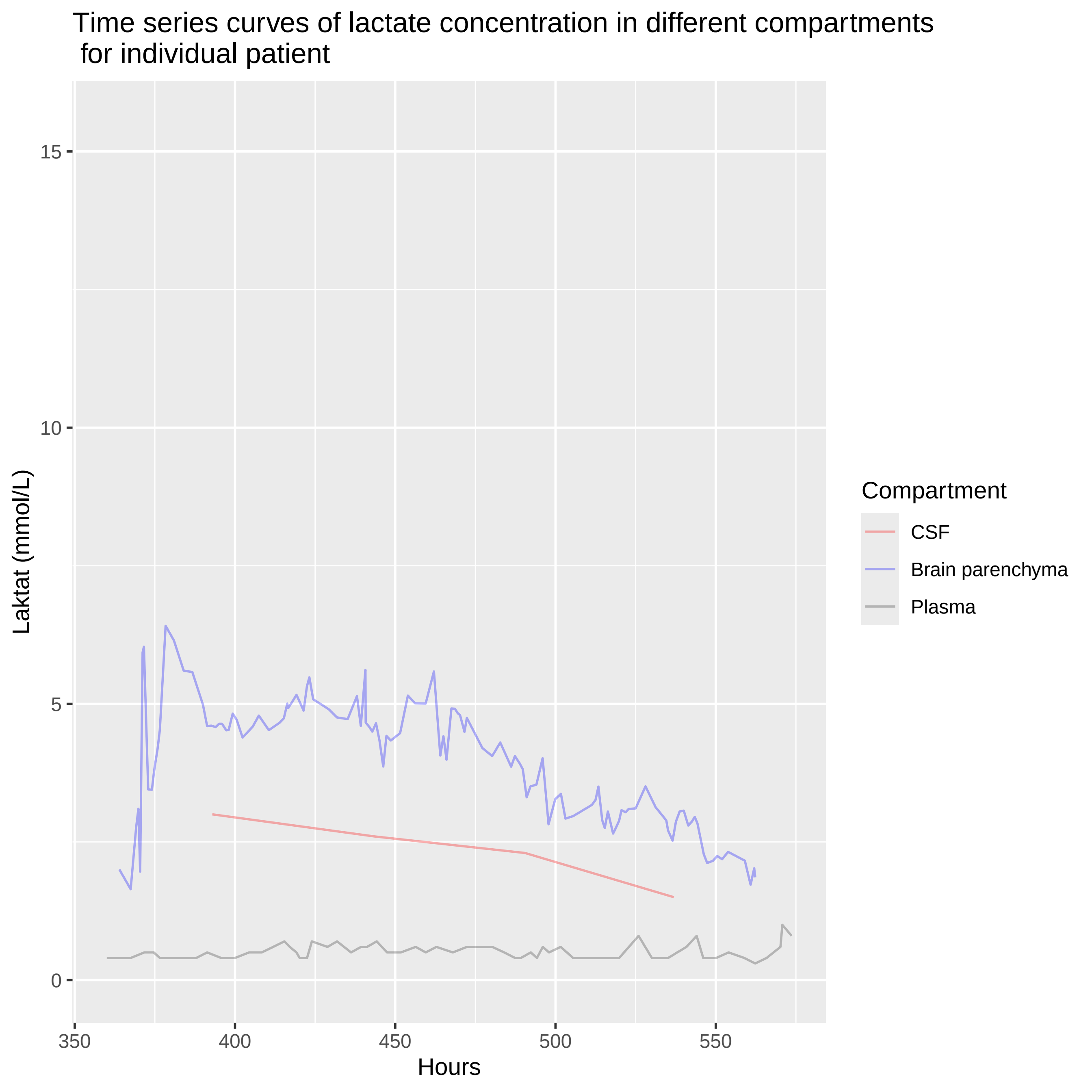
Time series curves of lactate concentration in different compartments
for individual patient
Laktat (mmol/L)
15
10
5
0
350
400
450
500
550
Hours
Compartment
CSF
Brain parenchyma
Plasma

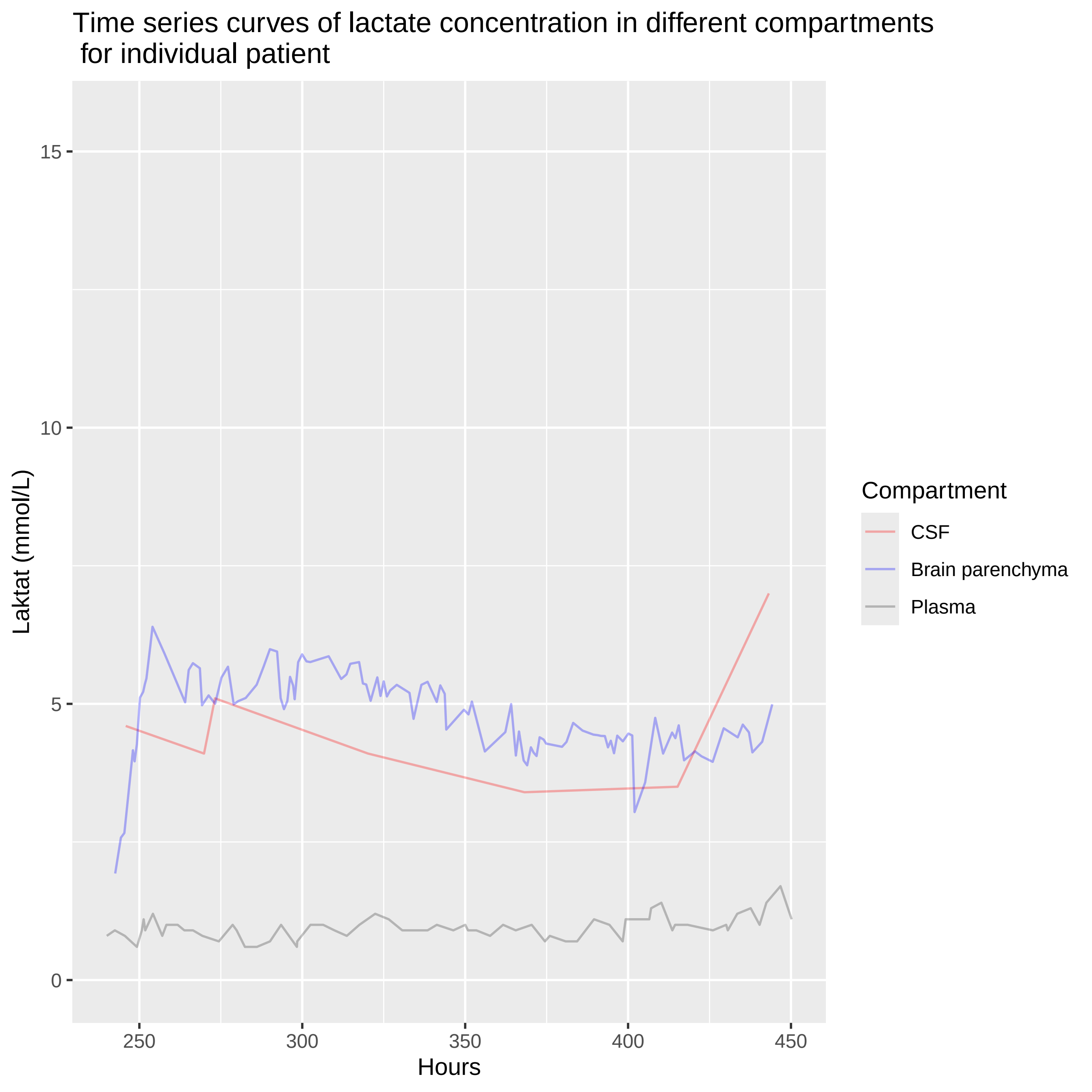

Time series curves of lactate concentration in different compartments
for individual patient
Laktat (mmol/L)
Hours
15
10
5
0
250
300
350
400
450
Compartment
CSF
Brain parenchyma
Plasma

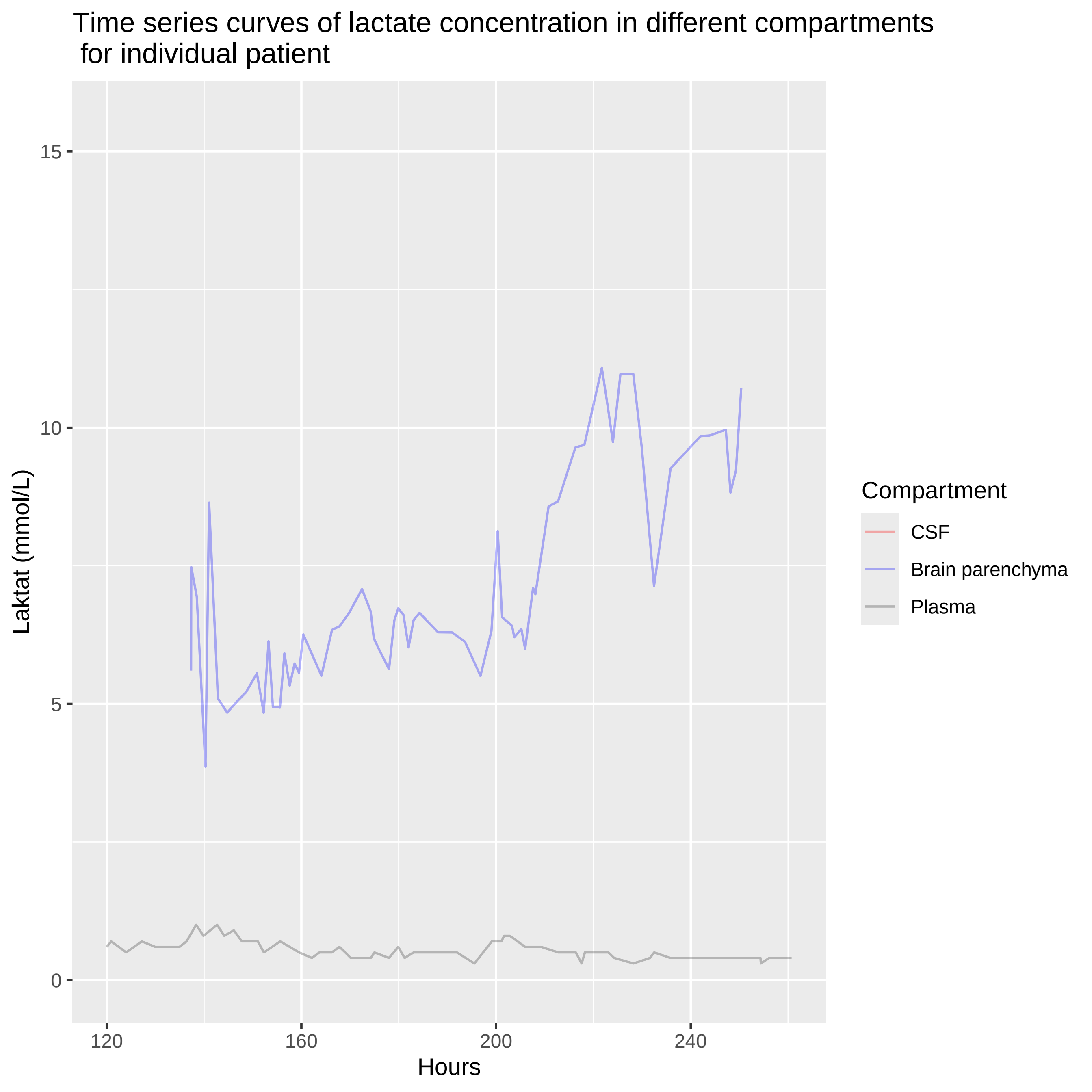

Time series curves of lactate concentration in different compartments
for individual patient
15
10
5
0
Laktat (mmol/L)
120
160
200
240
Hours
Compartment
CSF
Brain parenchyma
Plasma

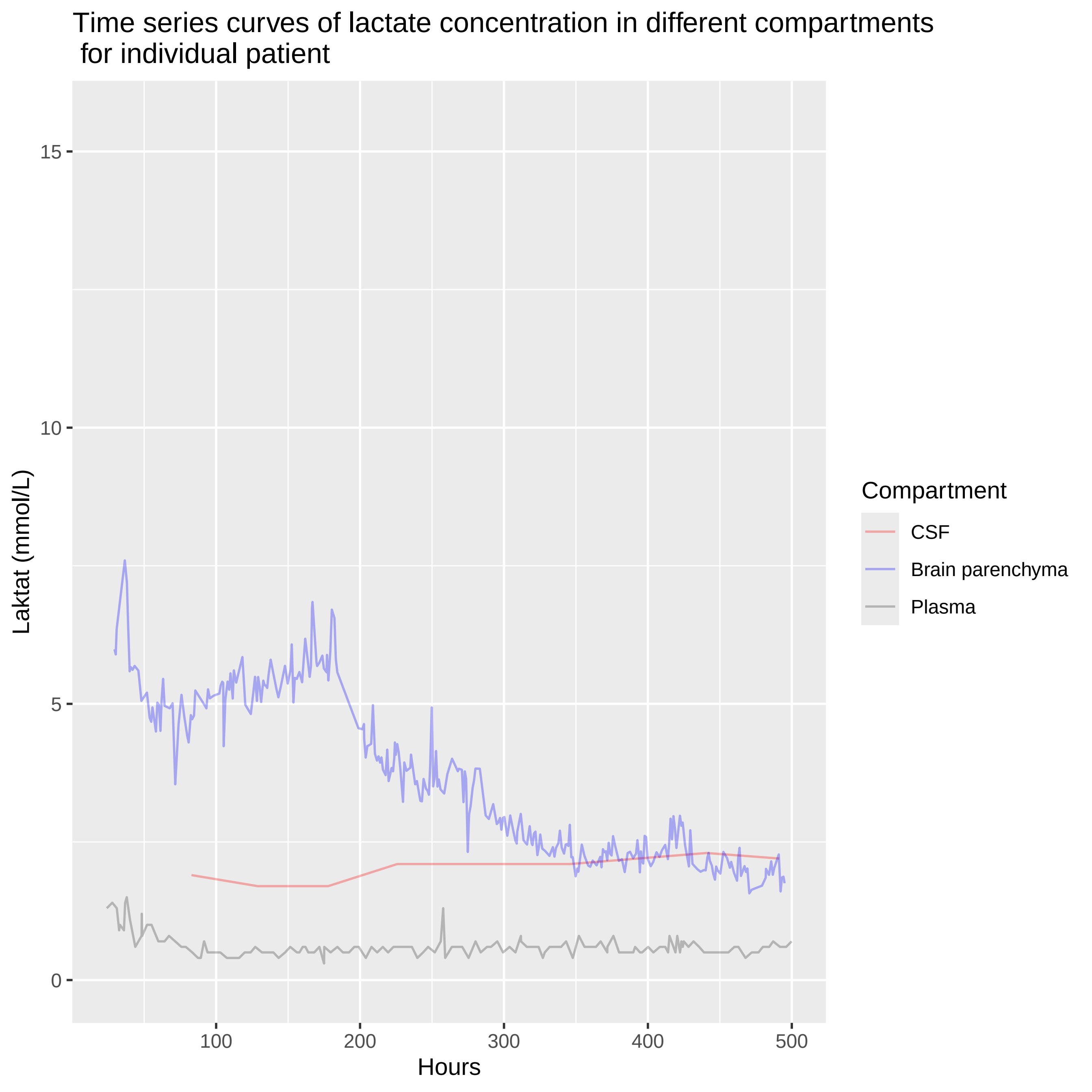

Time series curves of lactate concentration in different compartments
for individual patient
15
10
5
0
100
200
300
400
500
Hours
Laktat (mmol/L)
Compartment
CSF
Brain parenchyma
Plasma

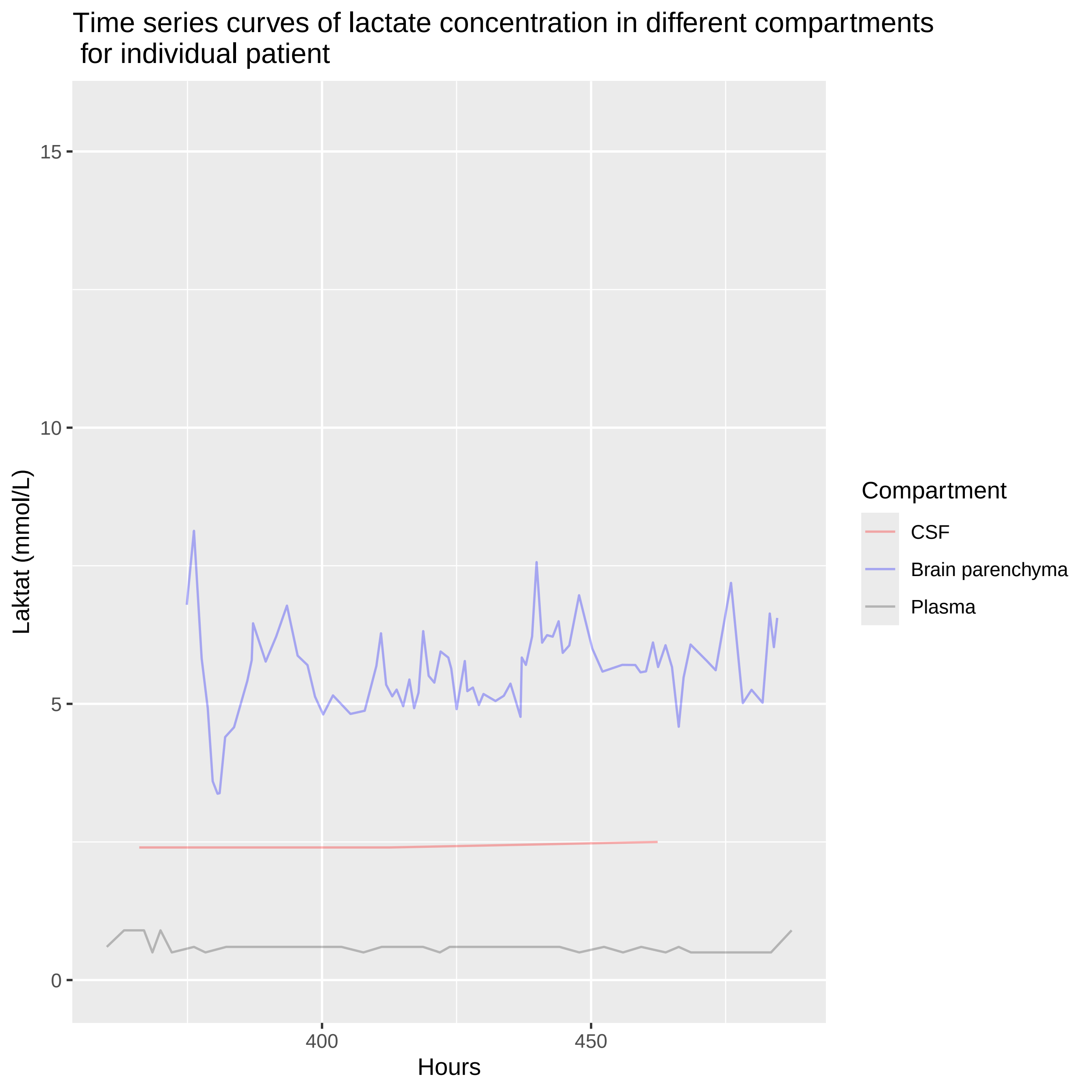

Time series curves of lactate concentration in different compartments
for individual patient
15
10
5
0
400
450
Hours
Laktat (mmol/L)
Compartment
CSF
Brain parenchyma
Plasma

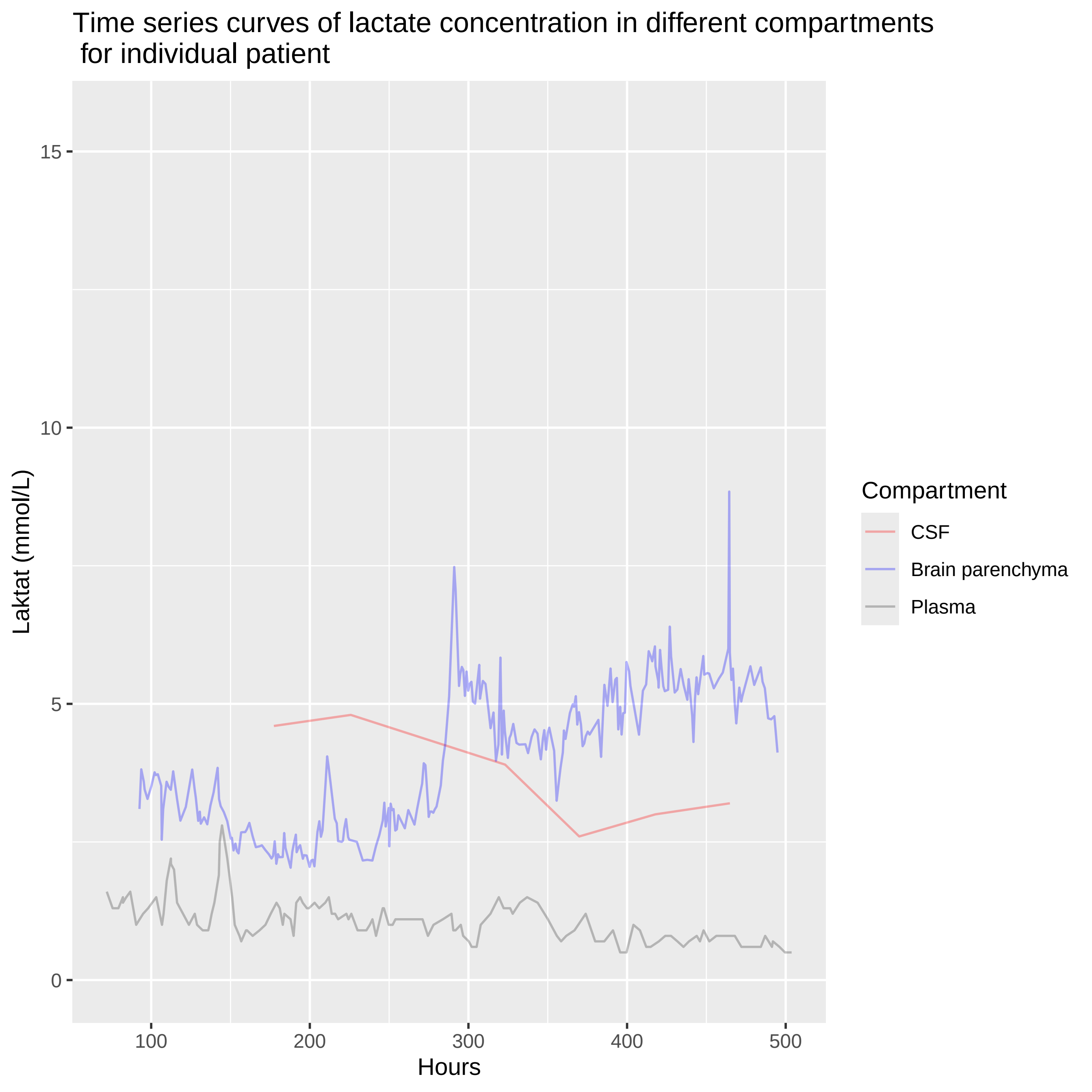
Time series curves of lactate concentration in different compartments
for individual patient
Laktat (mmol/L)
Hours
15
10
5
0
100
200
300
400
500
Compartment
CSF
Brain parenchyma
Plasma

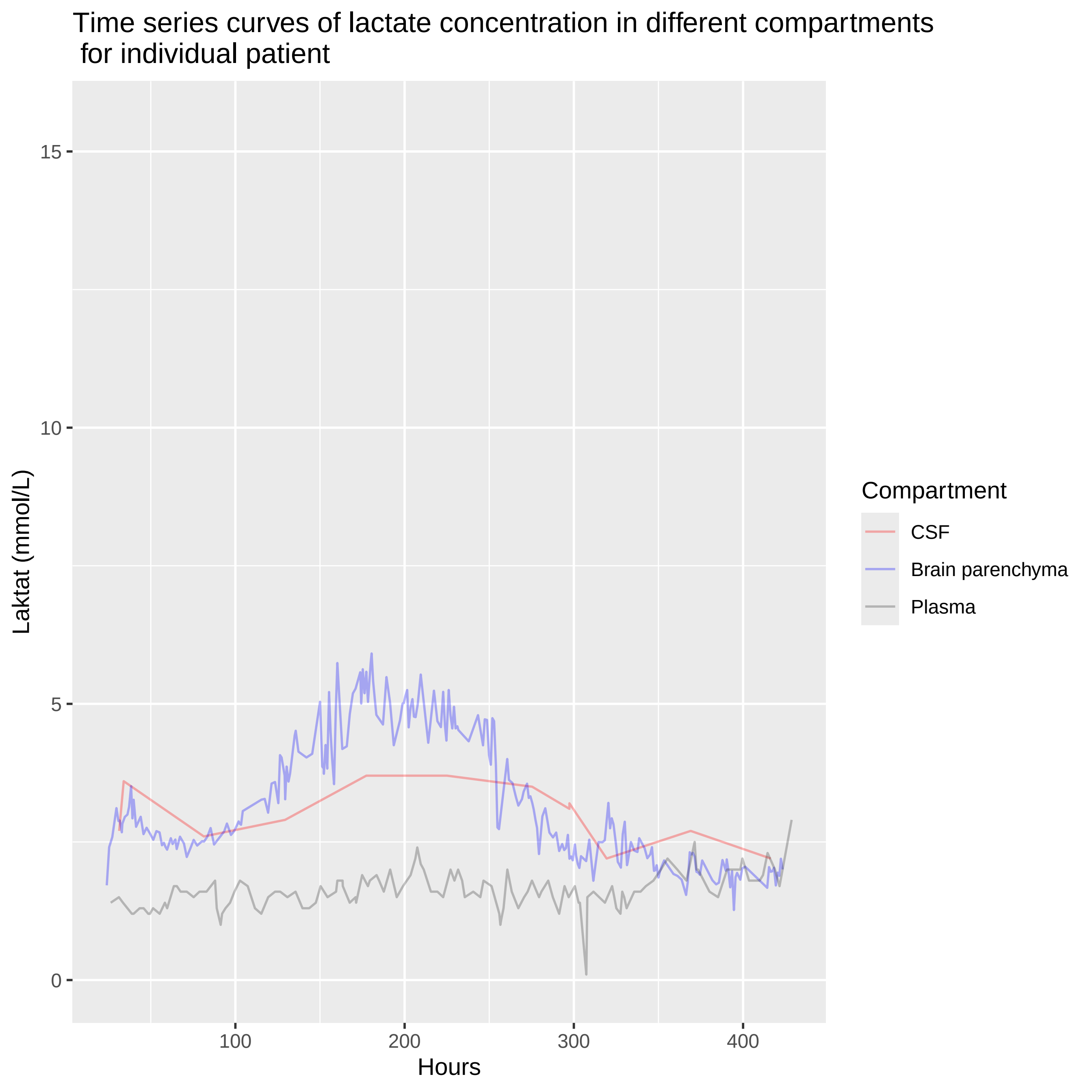

Time series curves of lactate concentration in different compartments
for individual patient
15
10
5
0
Laktat (mmol/L)
100
200
300
400
Hours
Compartment
CSF
Brain parenchyma
Plasma

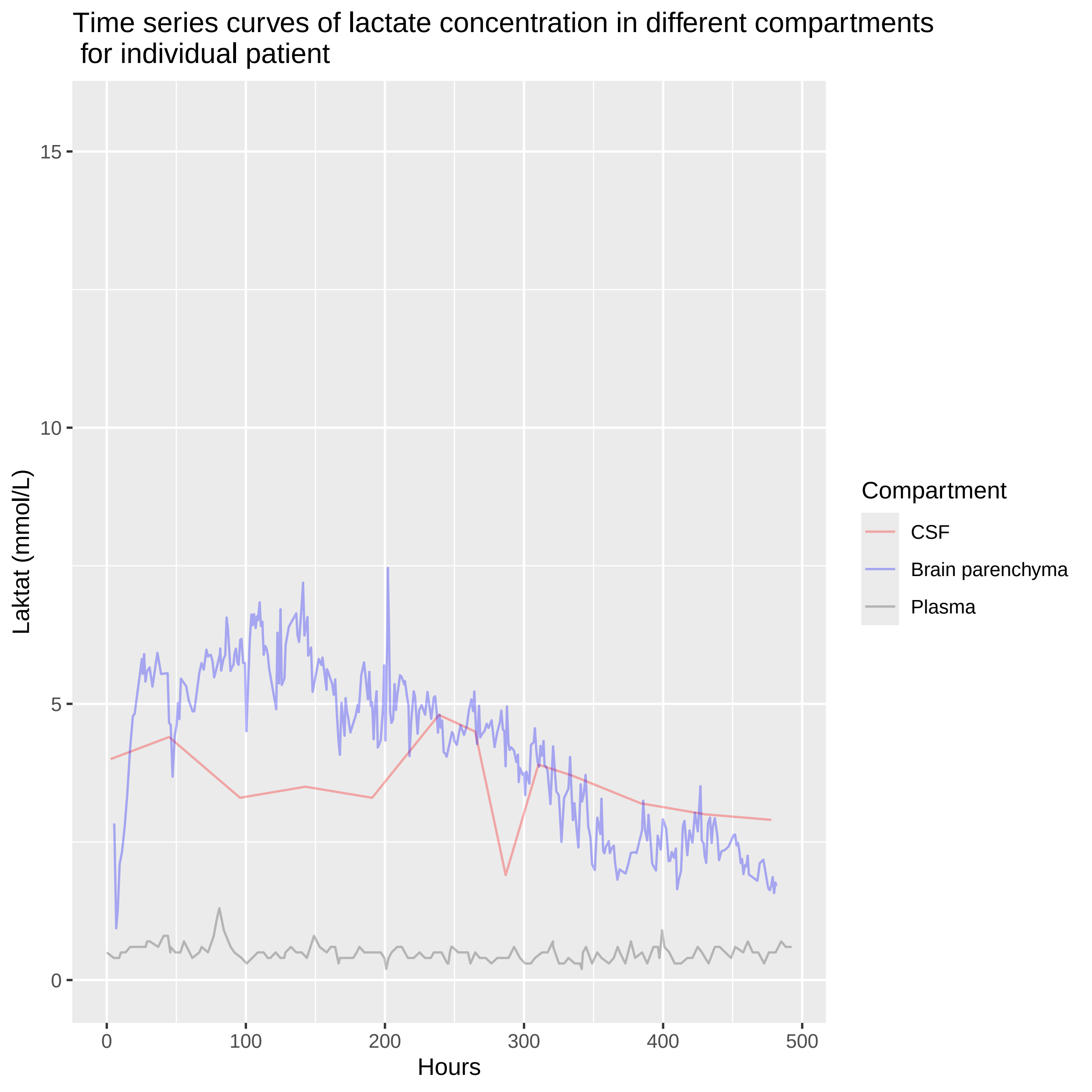
Time series curves of lactate concentration in different compartments
for individual patient
Laktat (mmol/L)
15
10
5
0
0
100
200
300
400
500
Hours
Compartment
CSF
Brain parenchyma
Plasma

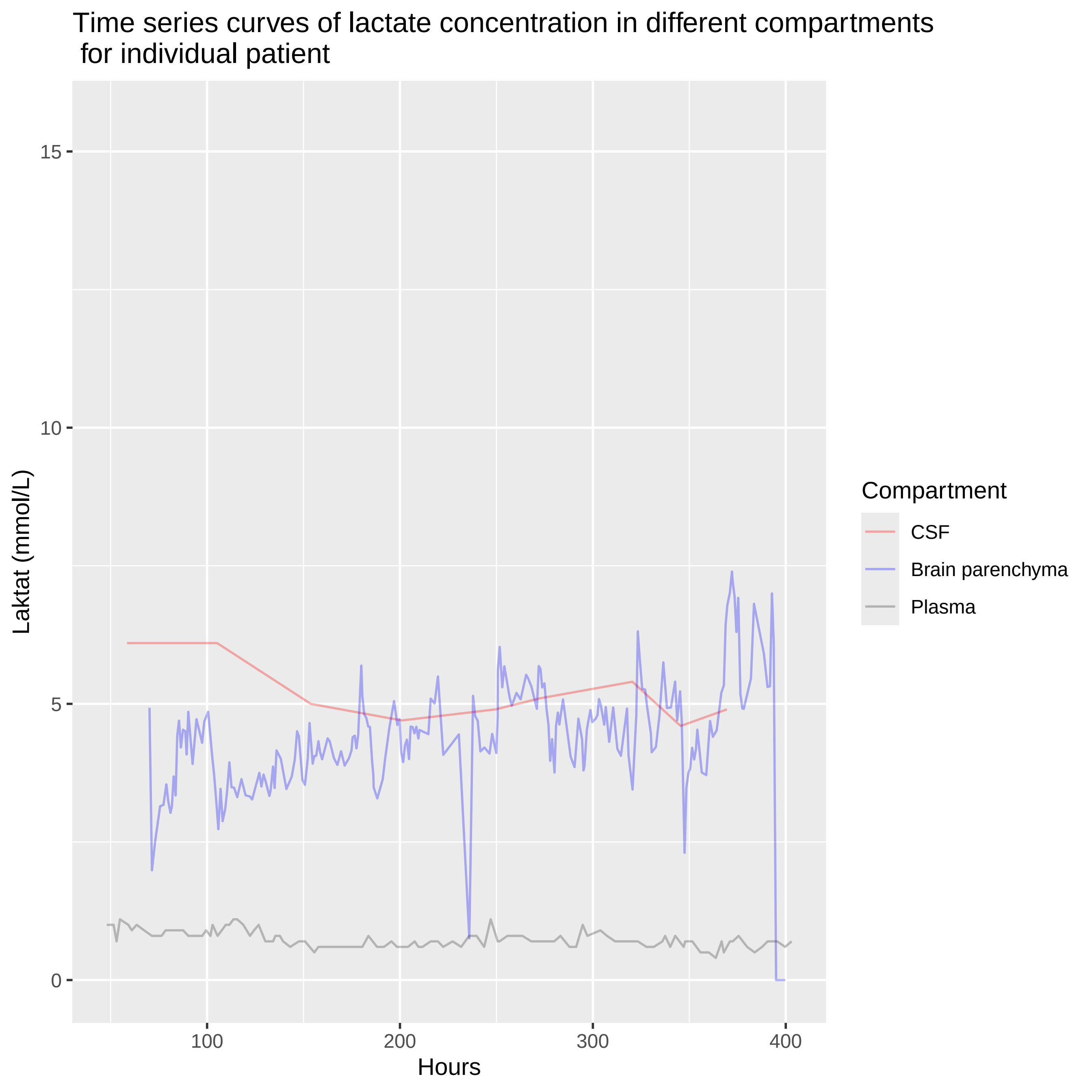

Time series curves of lactate concentration in different compartments
for individual patient
15
10
5
0
Laktat (mmol/L)
100
200
300
400
Hours
Compartment
CSF
Brain parenchyma
Plasma

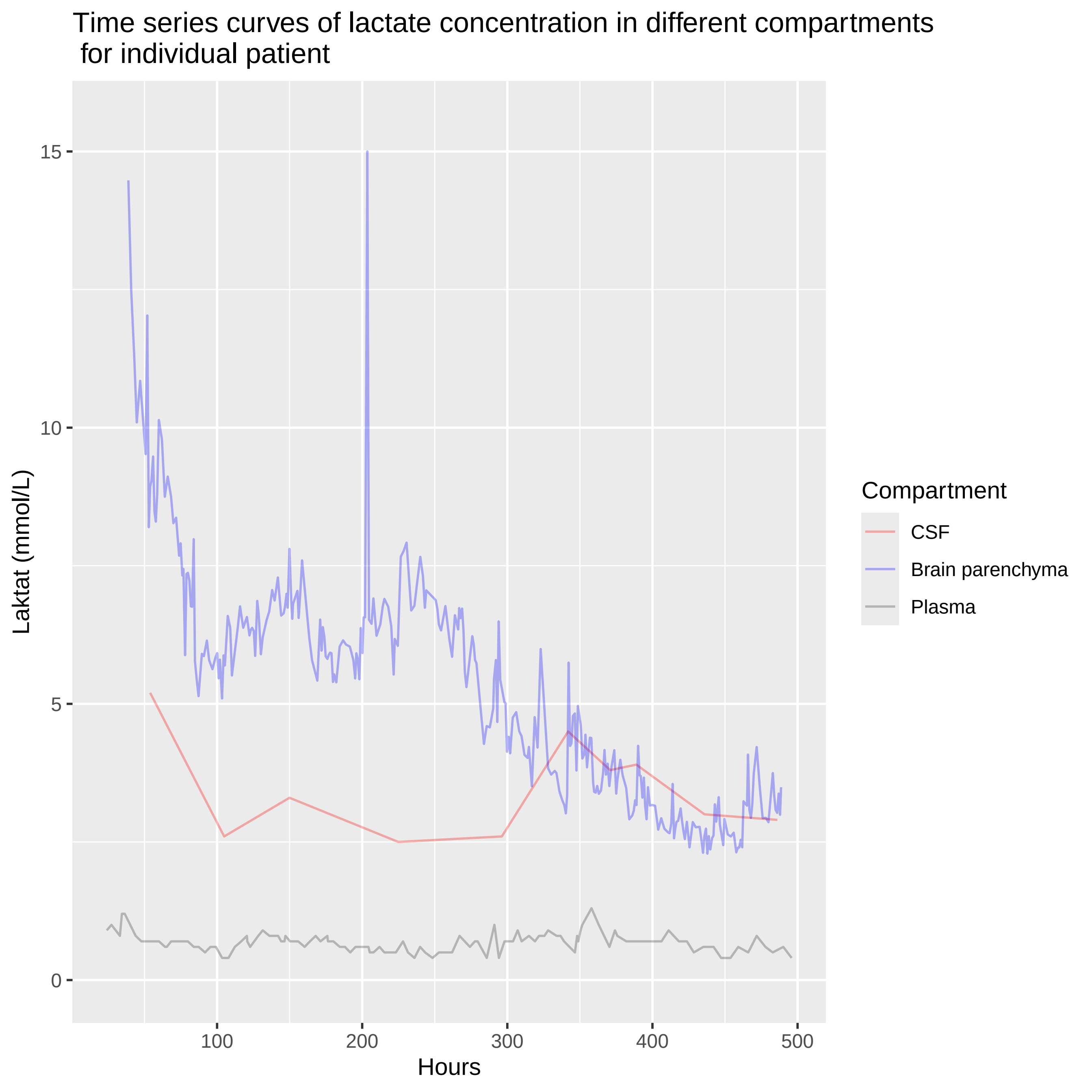

Time series curves of lactate concentration in different compartments
for individual patient
Laktat (mmol/L)
15
10
5
0
100
200
300
400
500
Hours
Compartment
CSF
Brain parenchyma
Plasma

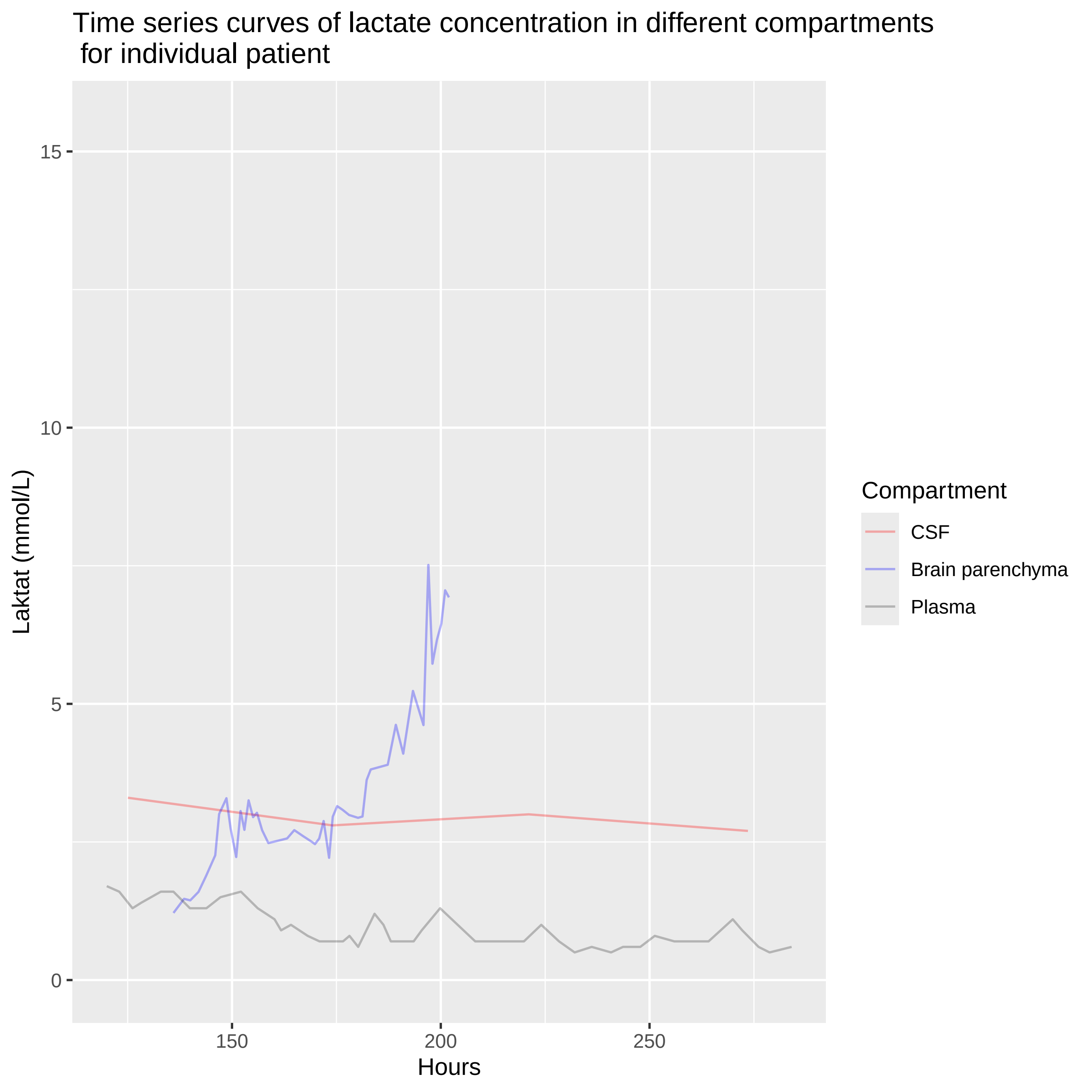

Time series curves of lactate concentration in different compartments
for individual patient
15
10
5
0
Laktat (mmol/L)
150
200
250
Hours
Compartment
CSF
Brain parenchyma
Plasma

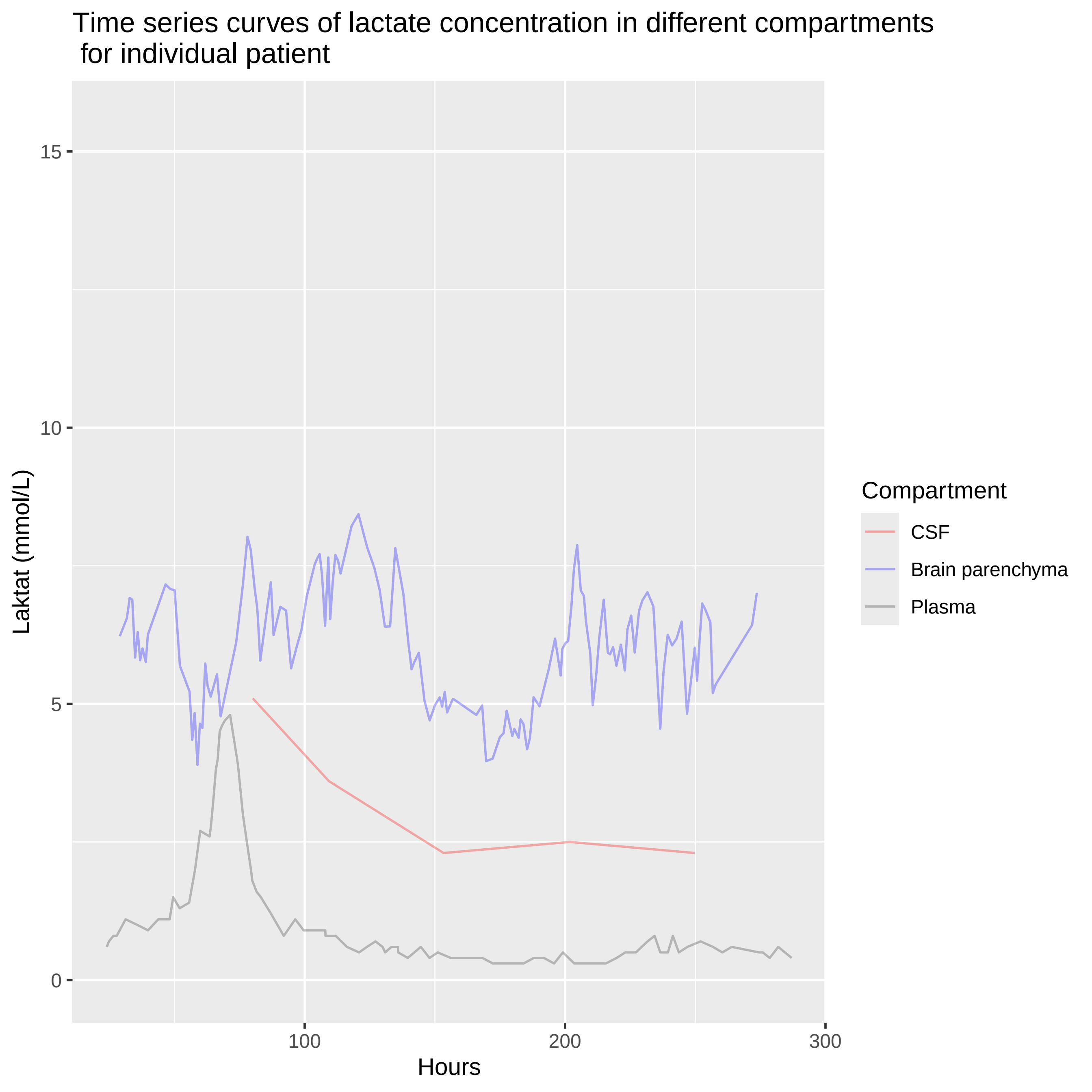

Time series curves of lactate concentration in different compartments
for individual patient
15
10
5
0
Laktat (mmol/L)
100
200
300
Hours
Compartment
CSF
Brain parenchyma
Plasma

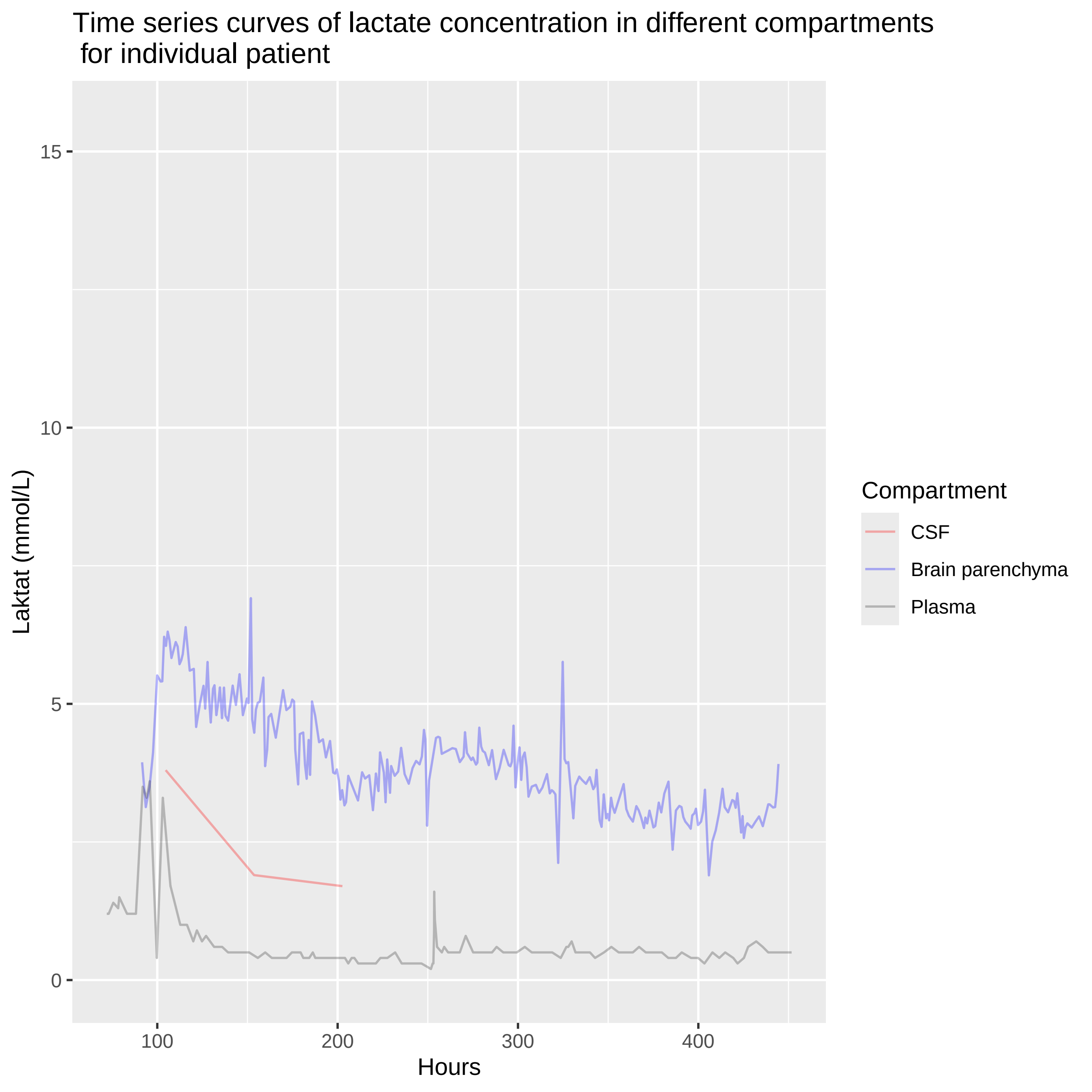

Time series curves of lactate concentration in different compartments
for individual patient
15
10
5
0
100
200
300
400
Hours
Laktat (mmol/L)
Compartment
CSF
Brain parenchyma
Plasma

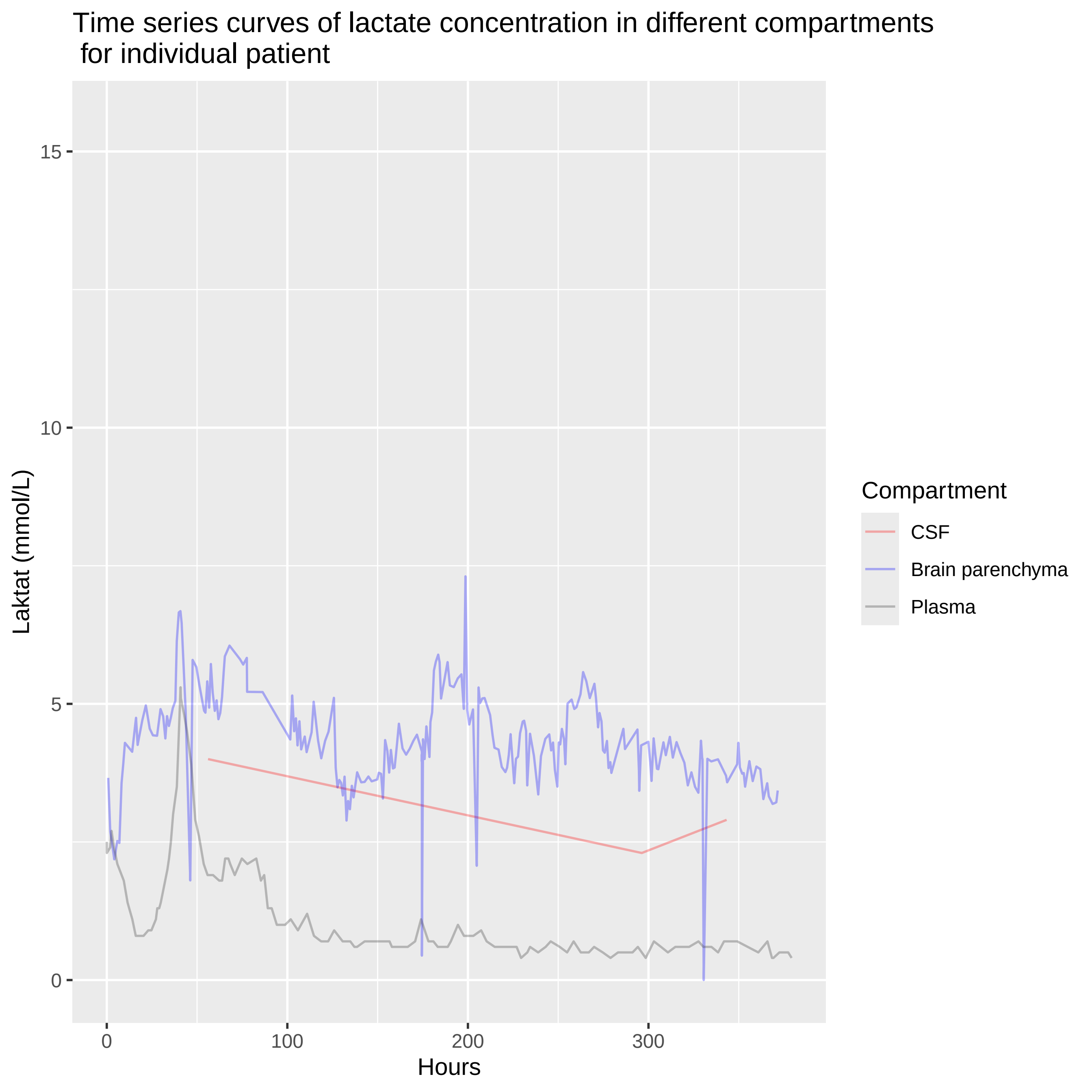

Time series curves of lactate concentration in different compartments
for individual patient
Laktat (mmol/L)
15
10
5
0
0
100
200
300
Hours
Compartment
CSF
Brain parenchyma
Plasma

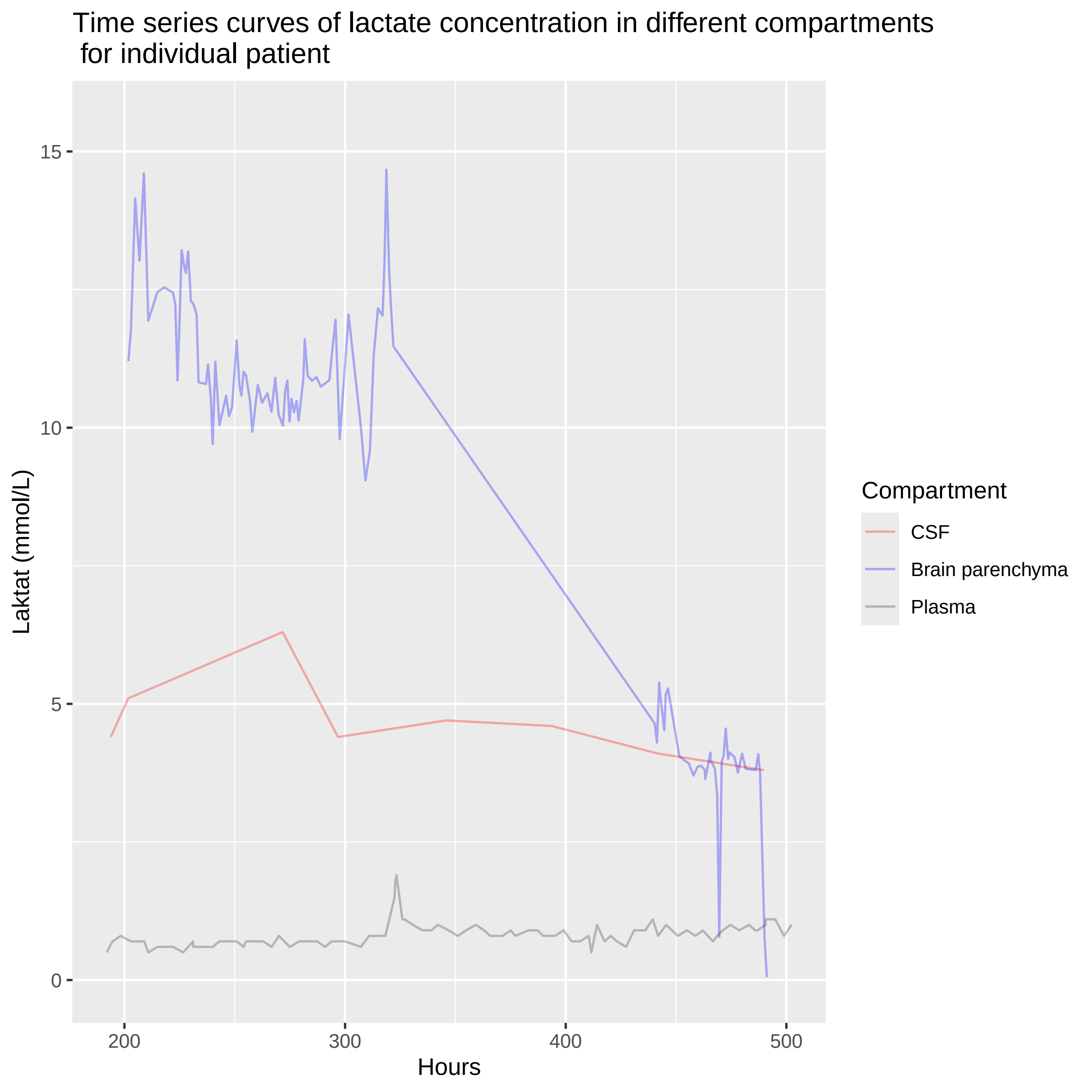

Time series curves of lactate concentration in different compartments
for individual patient
Laktat (mmol/L)
15
10
5
0
200
300
400
500
Hours
Compartment
CSF
Brain parenchyma
Plasma

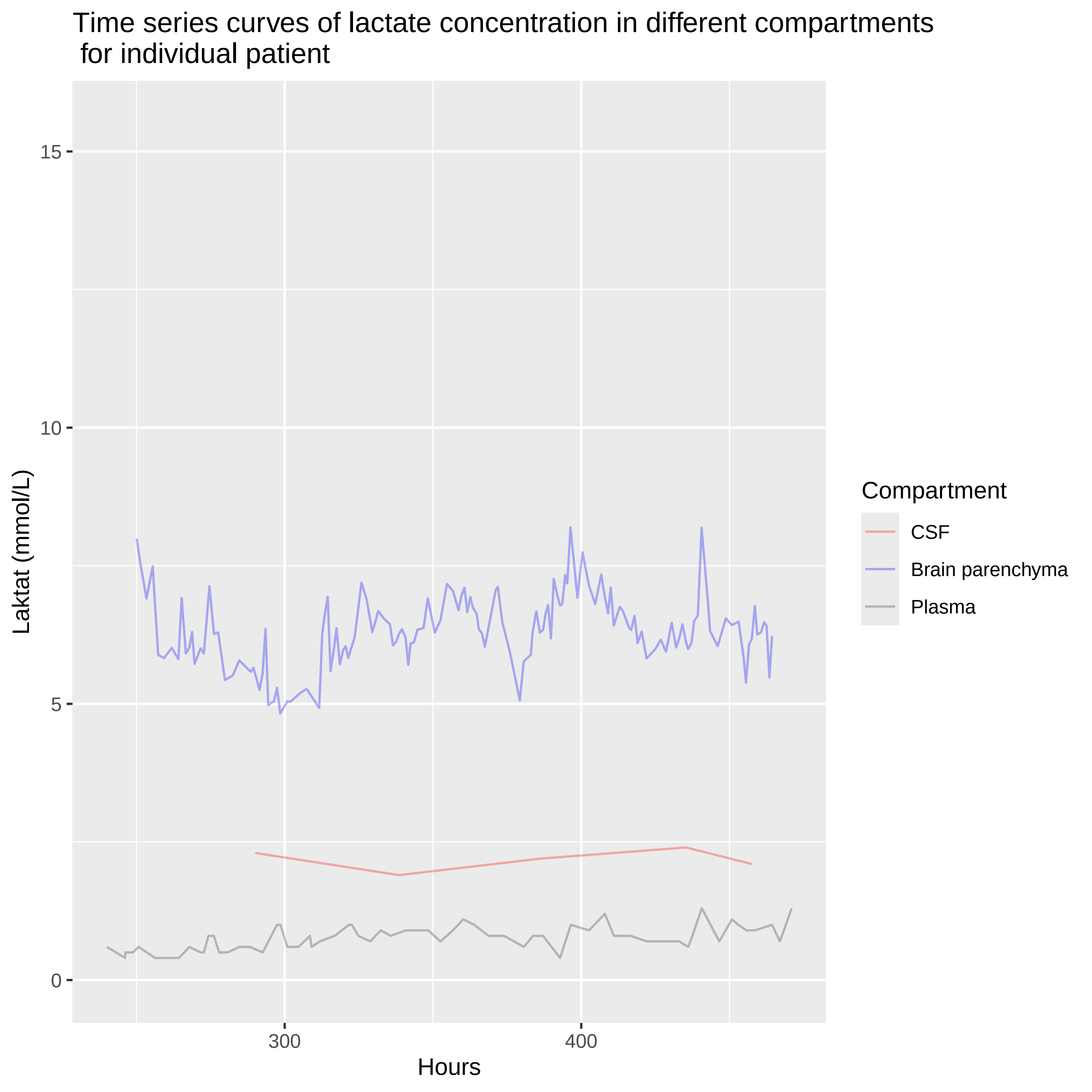

Time series curves of lactate concentration in different compartments
for individual patient
15
10
5
0
Laktat (mmol/L)
300
400
Hours
Compartment
CSF
Brain parenchyma
Plasma

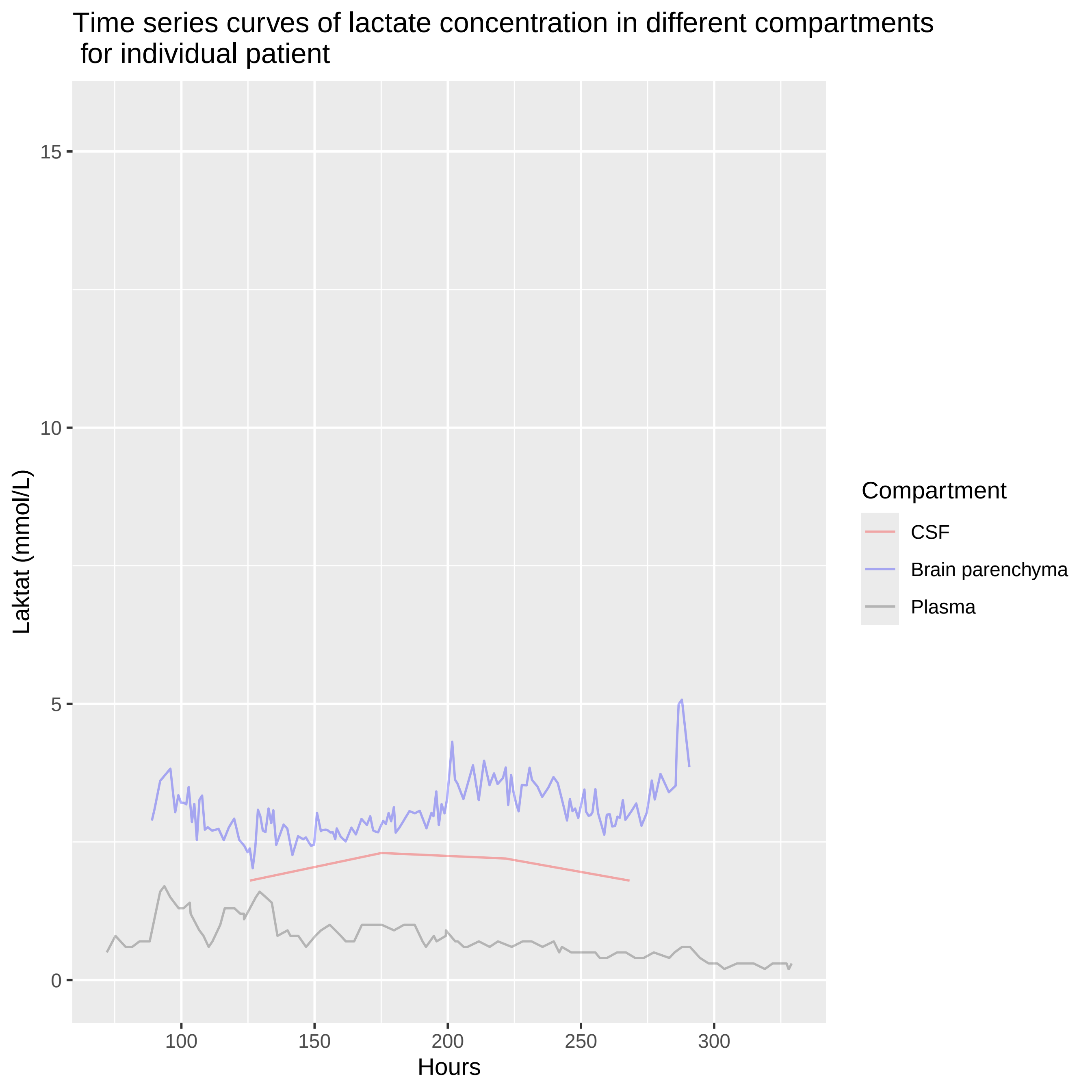
Time series curves of lactate concentration in different compartments
for individual patient
15
10
5
0
Laktat (mmol/L)
100
150
200
250
300
Hours
Compartment
CSF
Brain parenchyma
Plasma

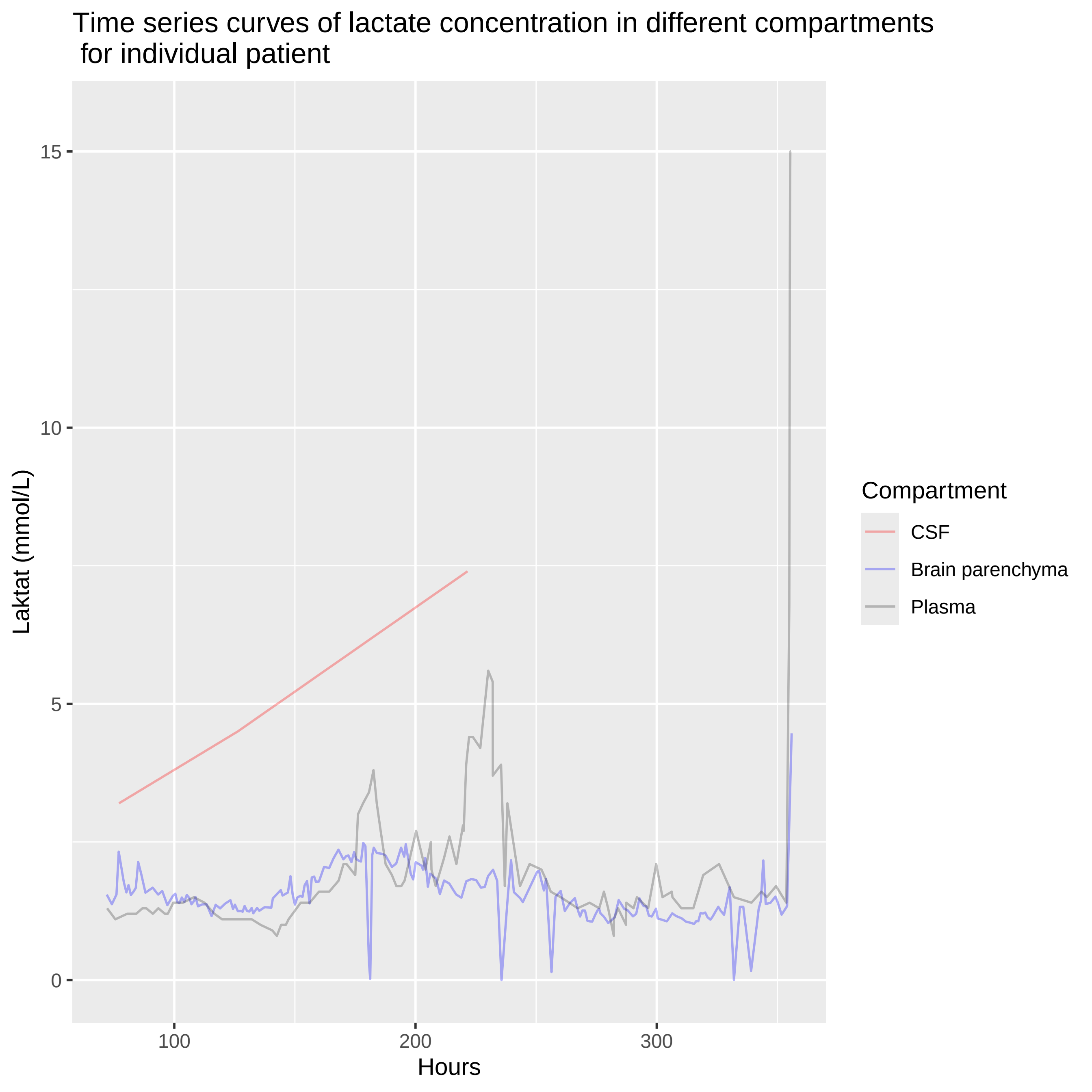
Time series curves of lactate concentration in different compartments
for individual patient
15
10
5
0
Laktat (mmol/L)
100
200
300
Hours
Compartment
CSF
Brain parenchyma
Plasma

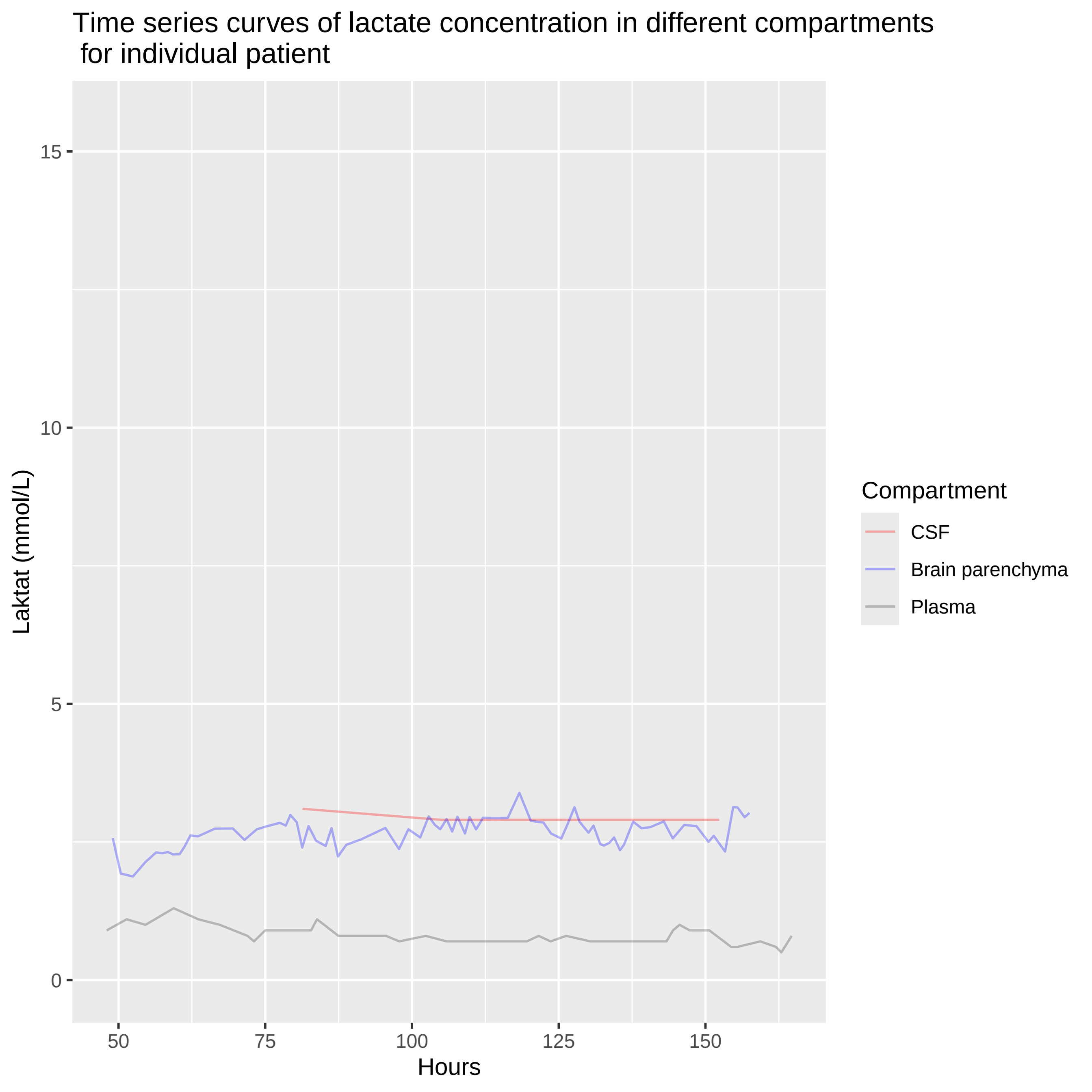

Time series curves of lactate concentration in different compartments
for individual patient
Laktat (mmol/L)
15
10
5
0
50
75
100
125
150
Hours
Compartment
CSF
Brain parenchyma
Plasma

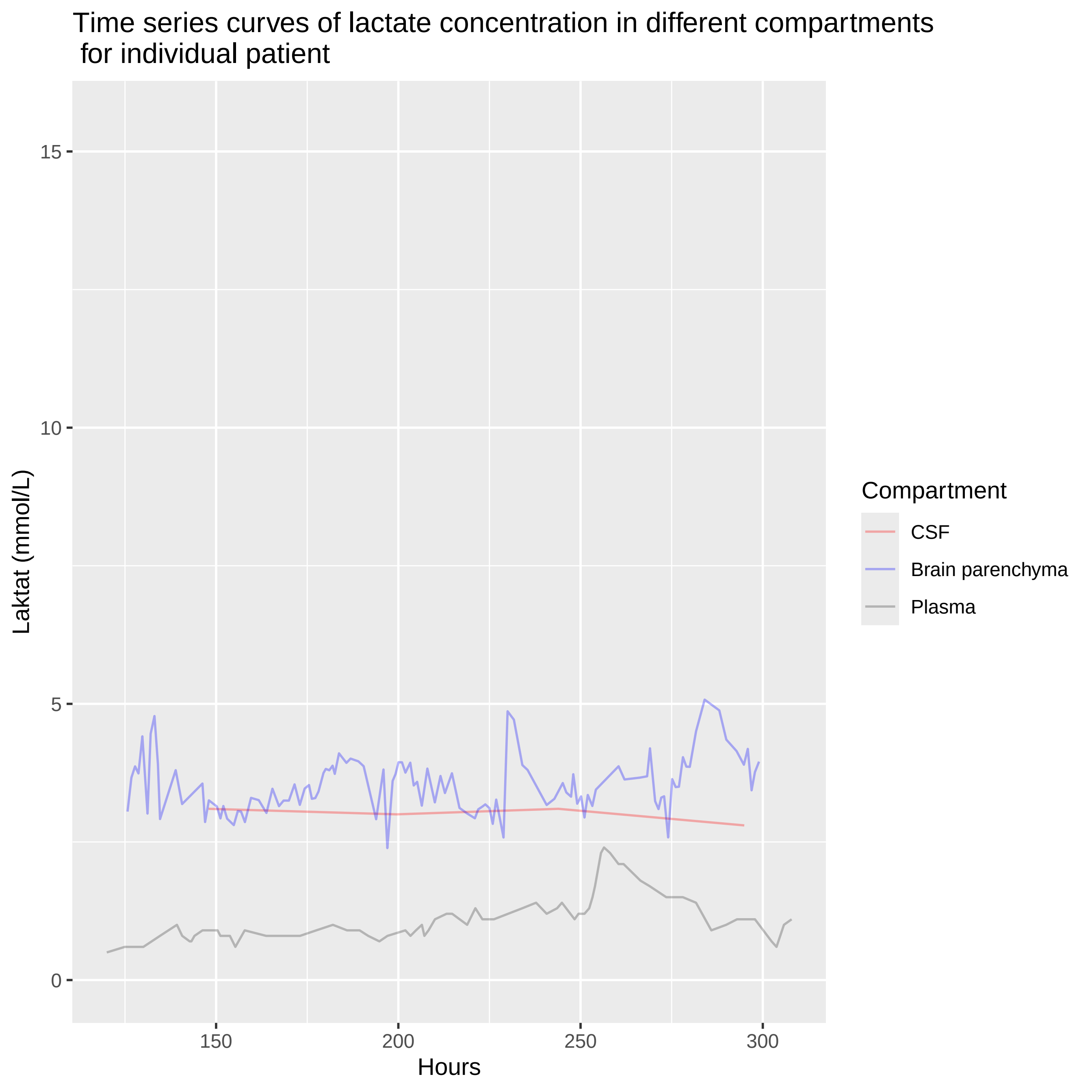

Time series curves of lactate concentration in different compartments
for individual patient
Laktat (mmol/L)
Hours
15
10
5
0
150
200
250
300
Compartment
CSF
Brain parenchyma
Plasma

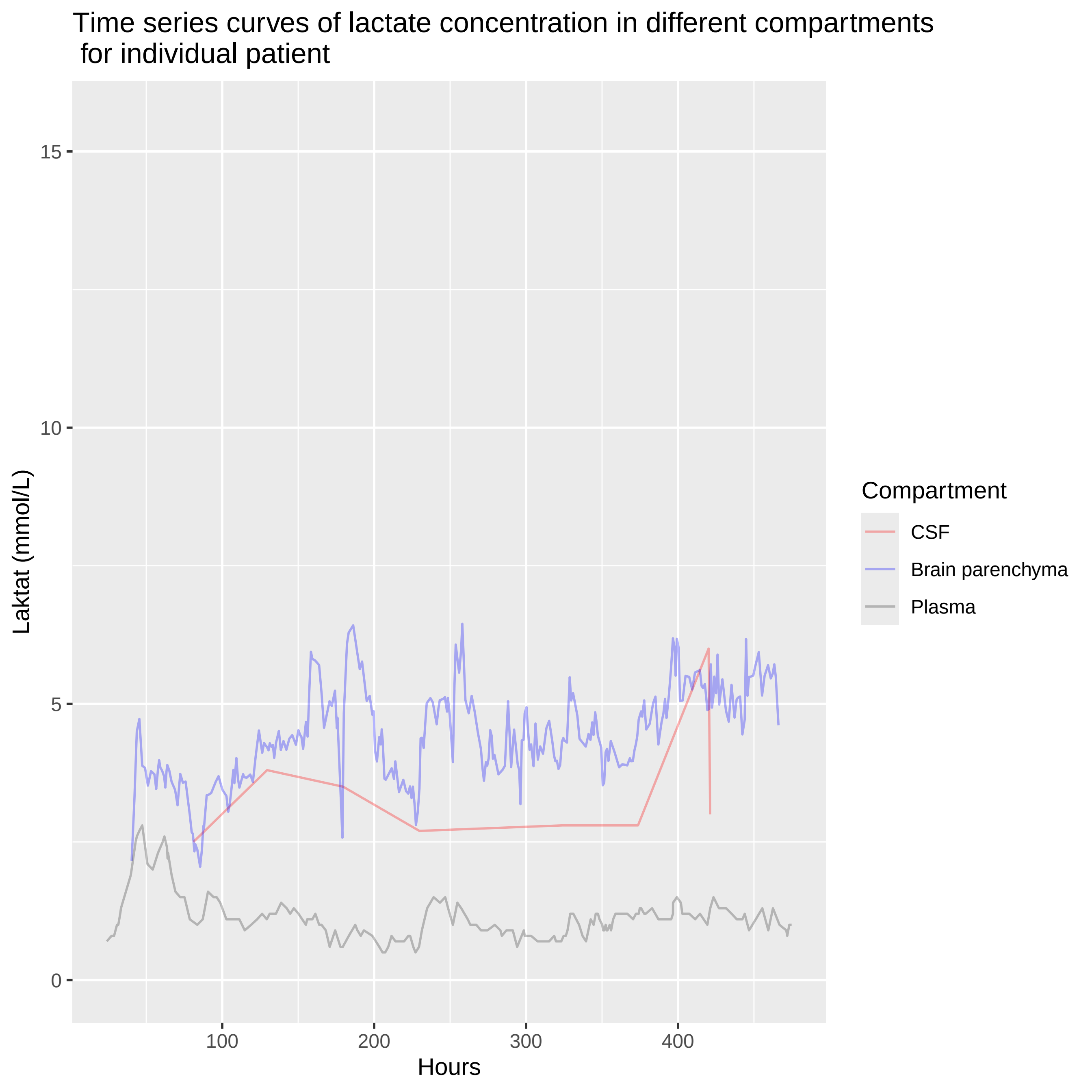

Time series curves of lactate concentration in different compartments
for individual patient
Laktat (mmol/L)
15
10
5
0
100
200
300
400
Hours
Compartment
CSF
Brain parenchyma
Plasma

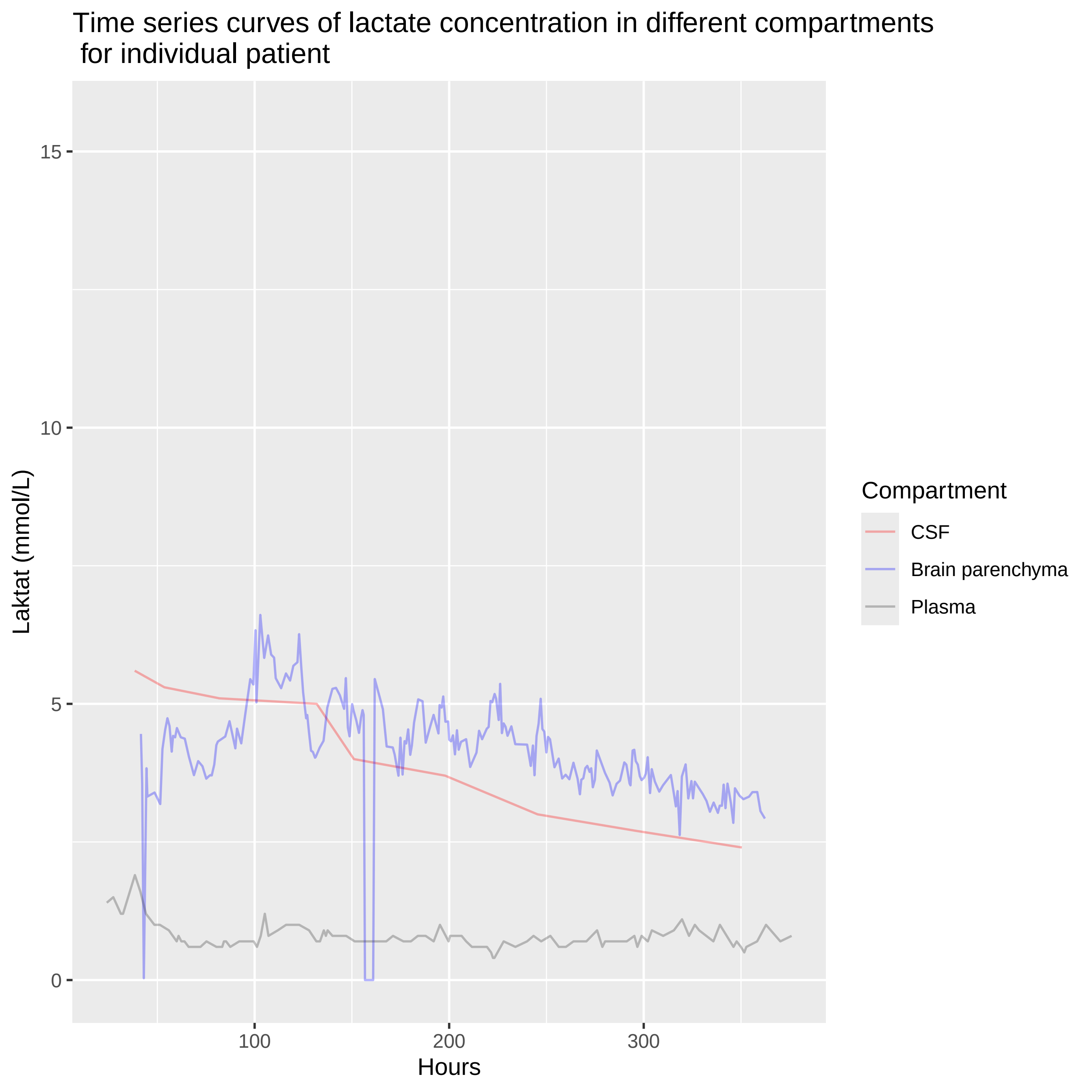

Time series curves of lactate concentration in different compartments
for individual patient
Laktat (mmol/L)
15
10
5
0
100
200
300
Hours
Compartment
CSF
Brain parenchyma
Plasma

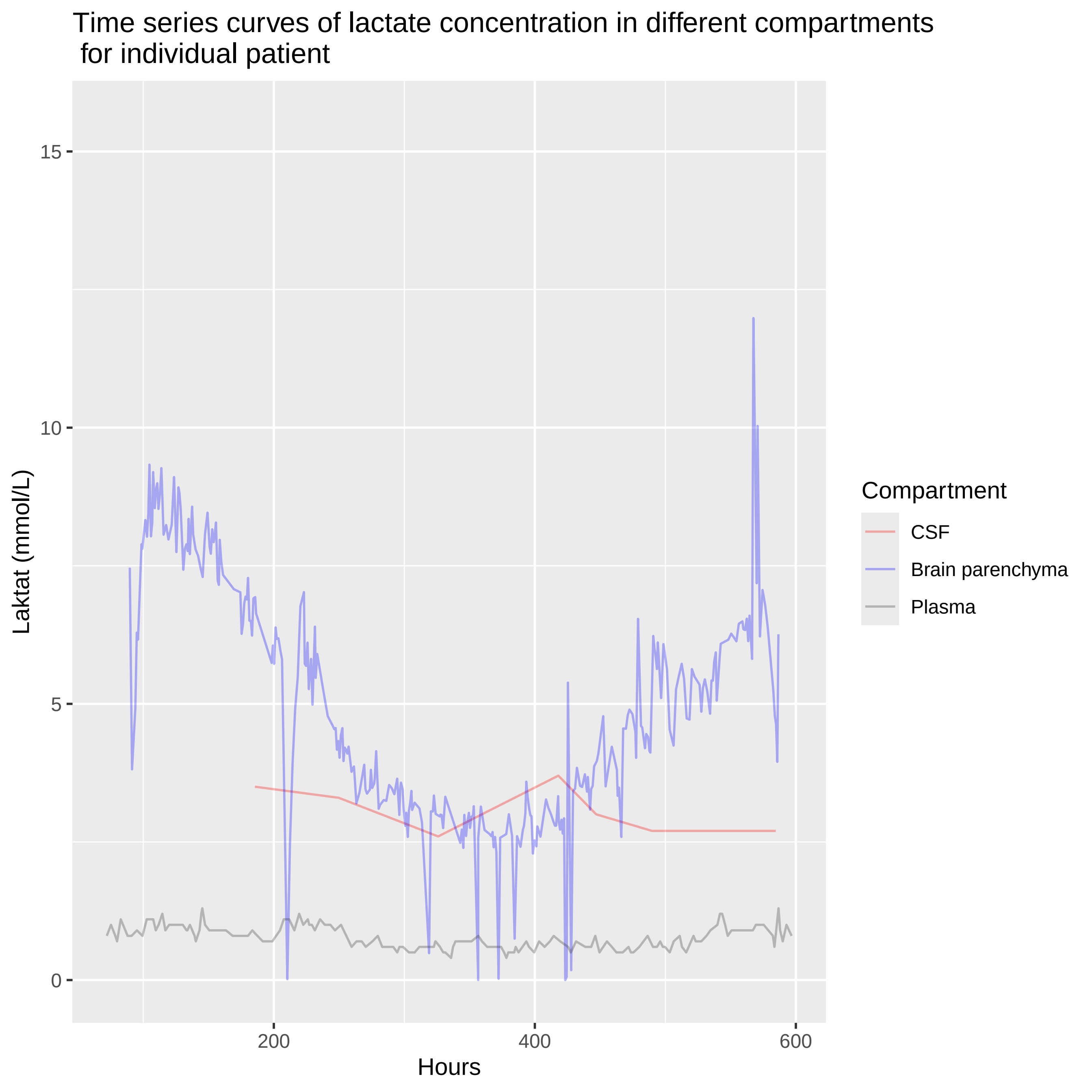
Time series curves of lactate concentration in different compartments
for individual patient
Laktat (mmol/L)
15
10
5
0
200
400
600
Hours
Compartment
CSF
Brain parenchyma
Plasma

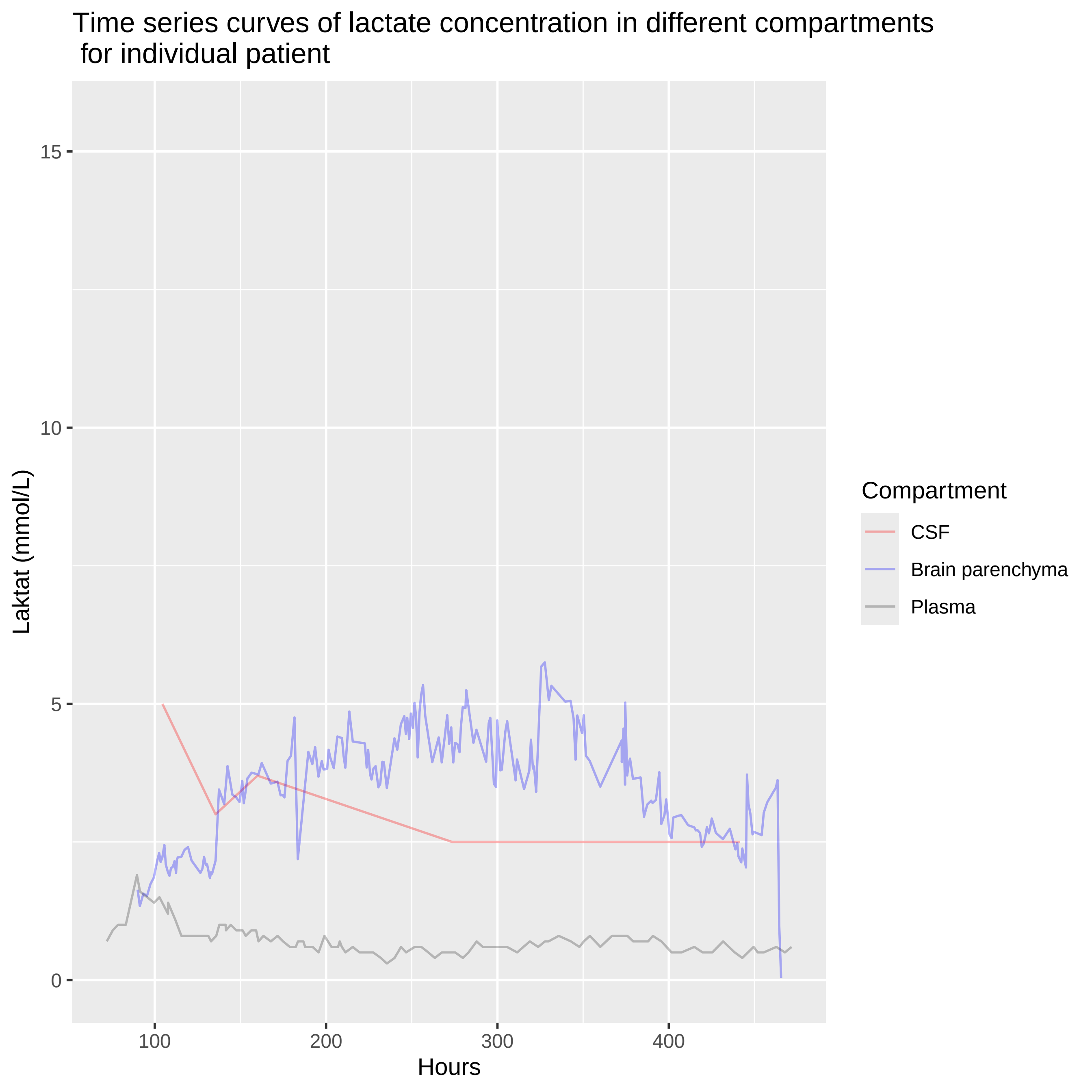
Time series curves of lactate concentration in different compartments
for individual patient
Laktat (mmol/L)
Hours
15
10
5
0
100
200
300
400
Compartment
CSF
Brain parenchyma
Plasma

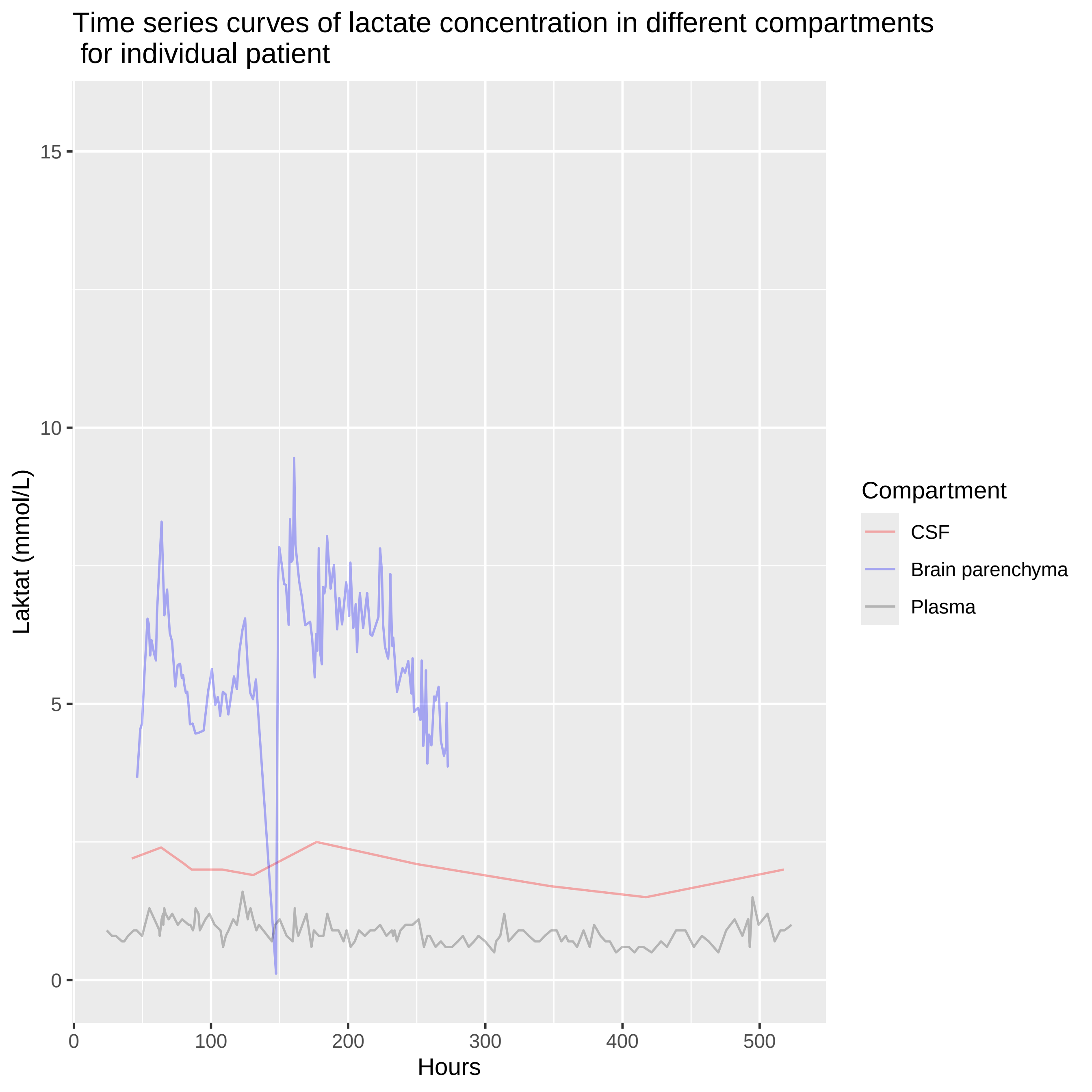
Time series curves of lactate concentration in different compartments
for individual patient
15
10
5
0
Laktat (mmol/L)
0
100
200
300
400
500
Hours
Compartment
CSF
Brain parenchyma
Plasma

# 5. Scatter Plots

Values above 15mmol/L where cut off at 15 mmol/L in the following plots.

## 5.1 CSF Glucose vs. Brain Parenchyma Glucose

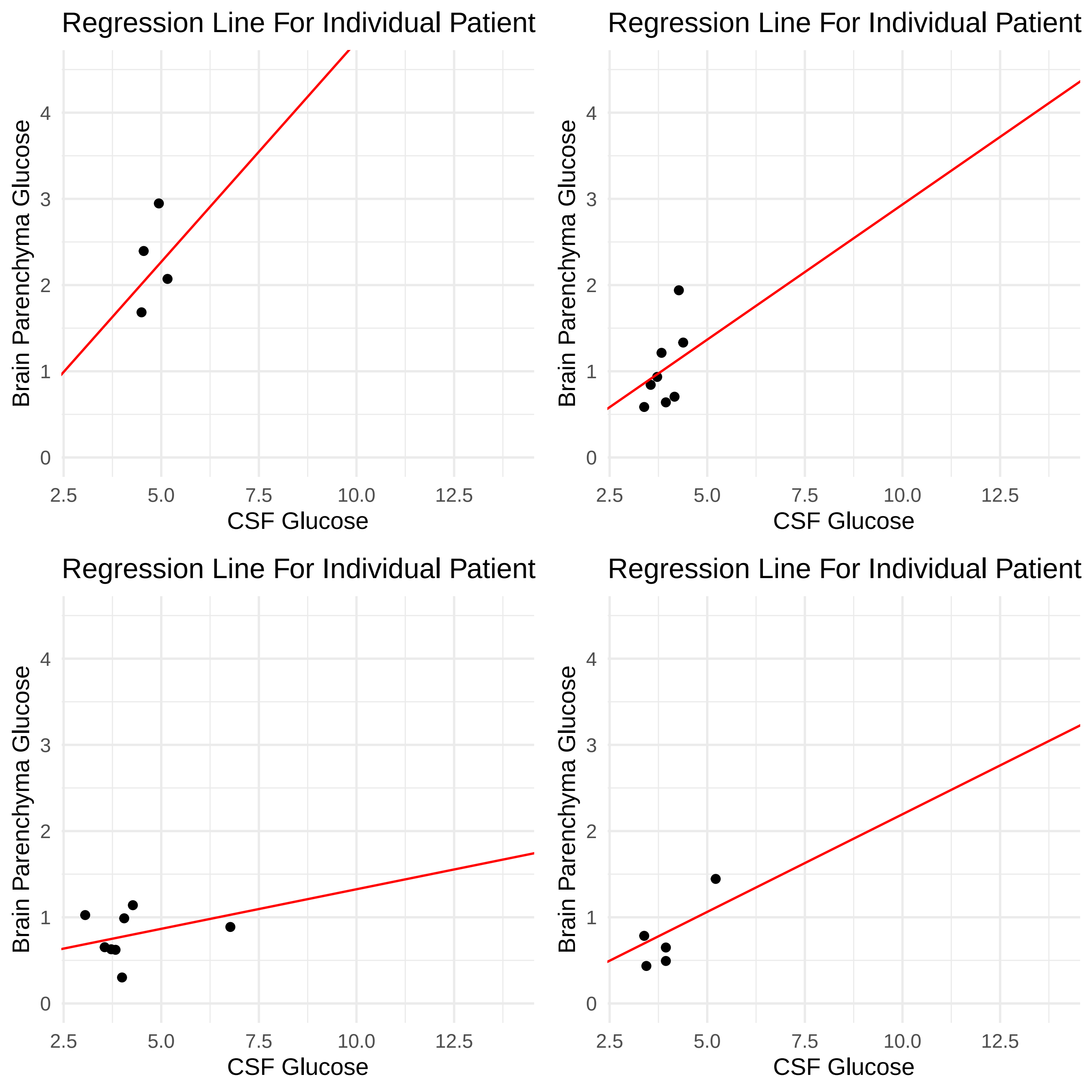
Regression Line For Individual Patient
Brain Parenchyma Glucose
CSF Glucose
0
1
2
3
4
2.5
5.0
7.5
10.0
12.5
Regression Line For Individual Patient
Brain Parenchyma Glucose
CSF Glucose
Regression Line For Individual Patient
Brain Parenchyma Glucose
CSF Glucose
Regression Line For Individual Patient
Brain Parenchyma Glucose
CSF Glucose

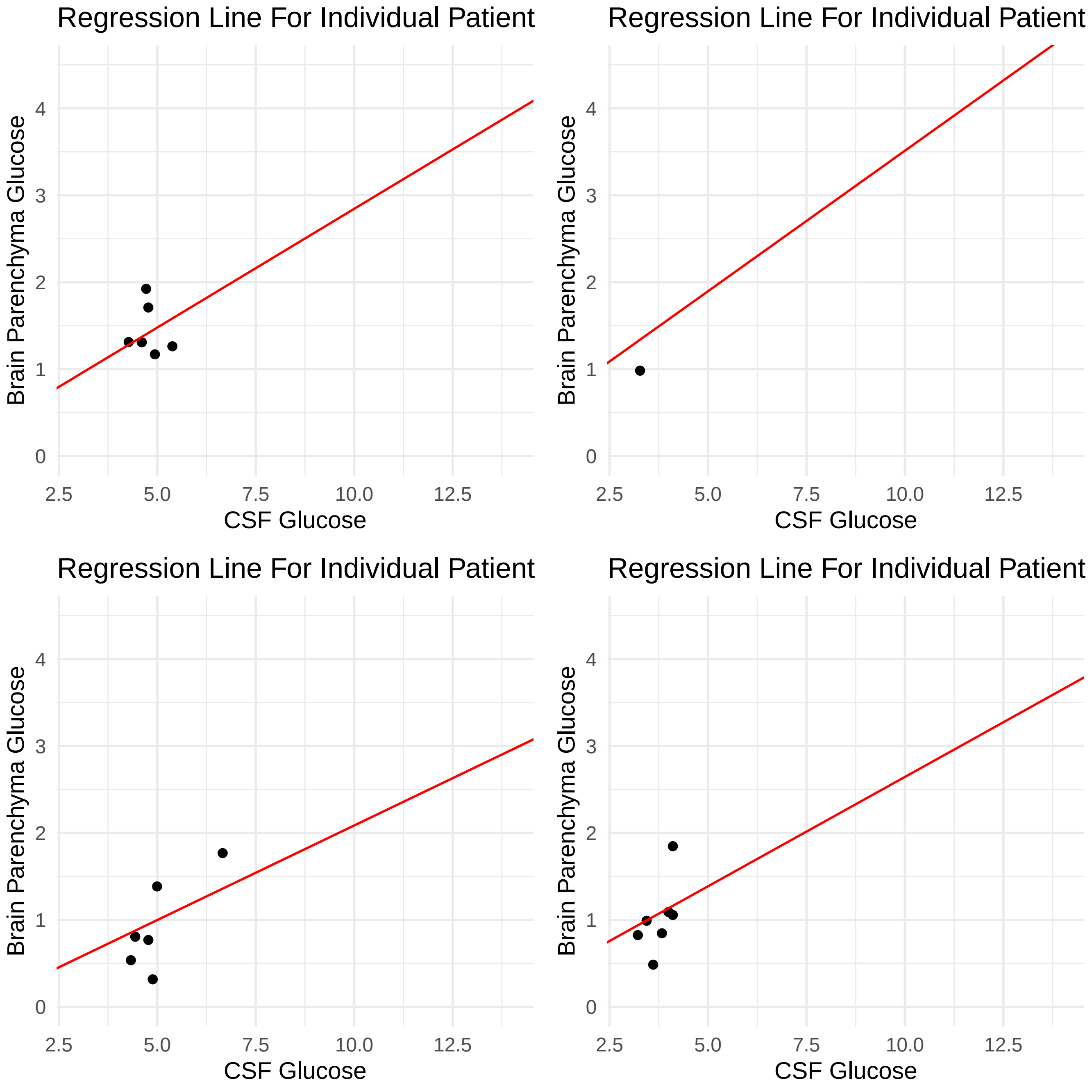
Regression Line For Individual Patient
Brain Parenchyma Glucose
CSF Glucose
0
1
2
3
4
2.5
5.0
7.5
10.0
12.5
Regression Line For Individual Patient
Brain Parenchyma Glucose
CSF Glucose
Regression Line For Individual Patient
Brain Parenchyma Glucose
CSF Glucose
Regression Line For Individual Patient
Brain Parenchyma Glucose
CSF Glucose

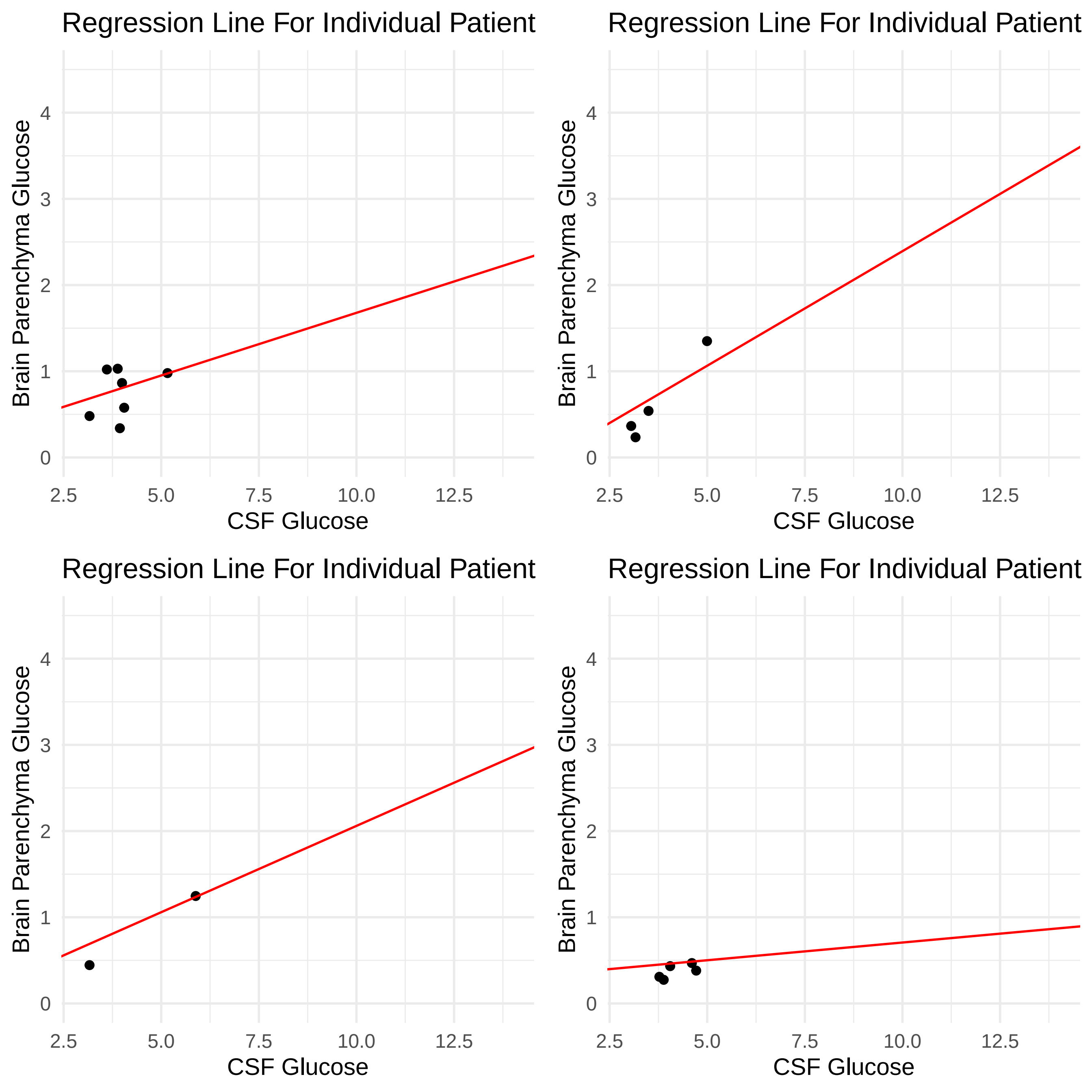
Regression Line For Individual Patient
Brain Parenchyma Glucose
CSF Glucose
0
1
2
3
4
2.5
5.0
7.5
10.0
12.5
Regression Line For Individual Patient
Brain Parenchyma Glucose
CSF Glucose
Regression Line For Individual Patient
Brain Parenchyma Glucose
CSF Glucose
Regression Line For Individual Patient
Brain Parenchyma Glucose
CSF Glucose

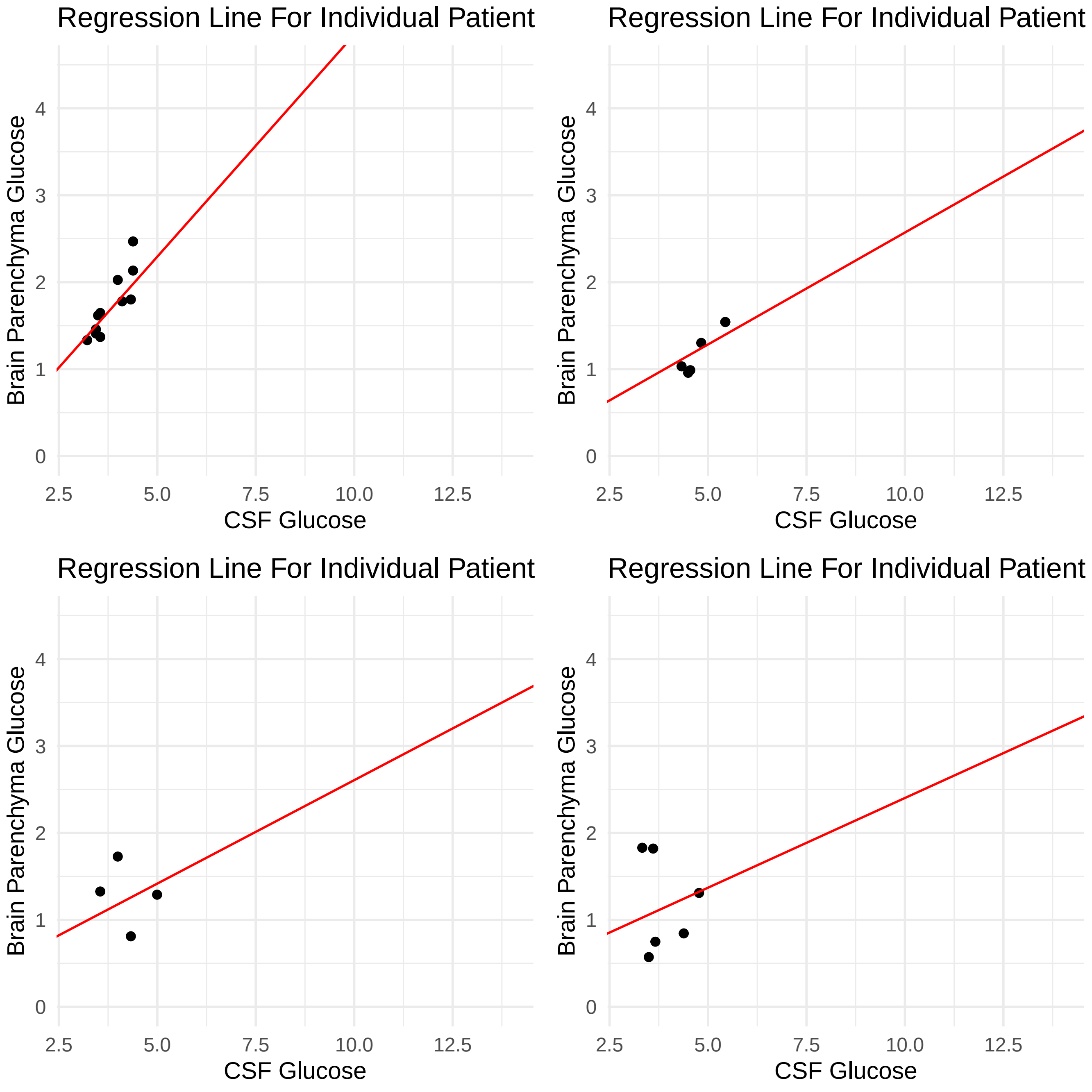

Regression Line For Individual Patient
Regression Line For Individual Patient
Regression Line For Individual Patient
Regression Line For Individual Patient
Brain Parenchyma Glucose
CSF Glucose
0
1
2
3
4
2.5
5.0
7.5
10.0
12.5

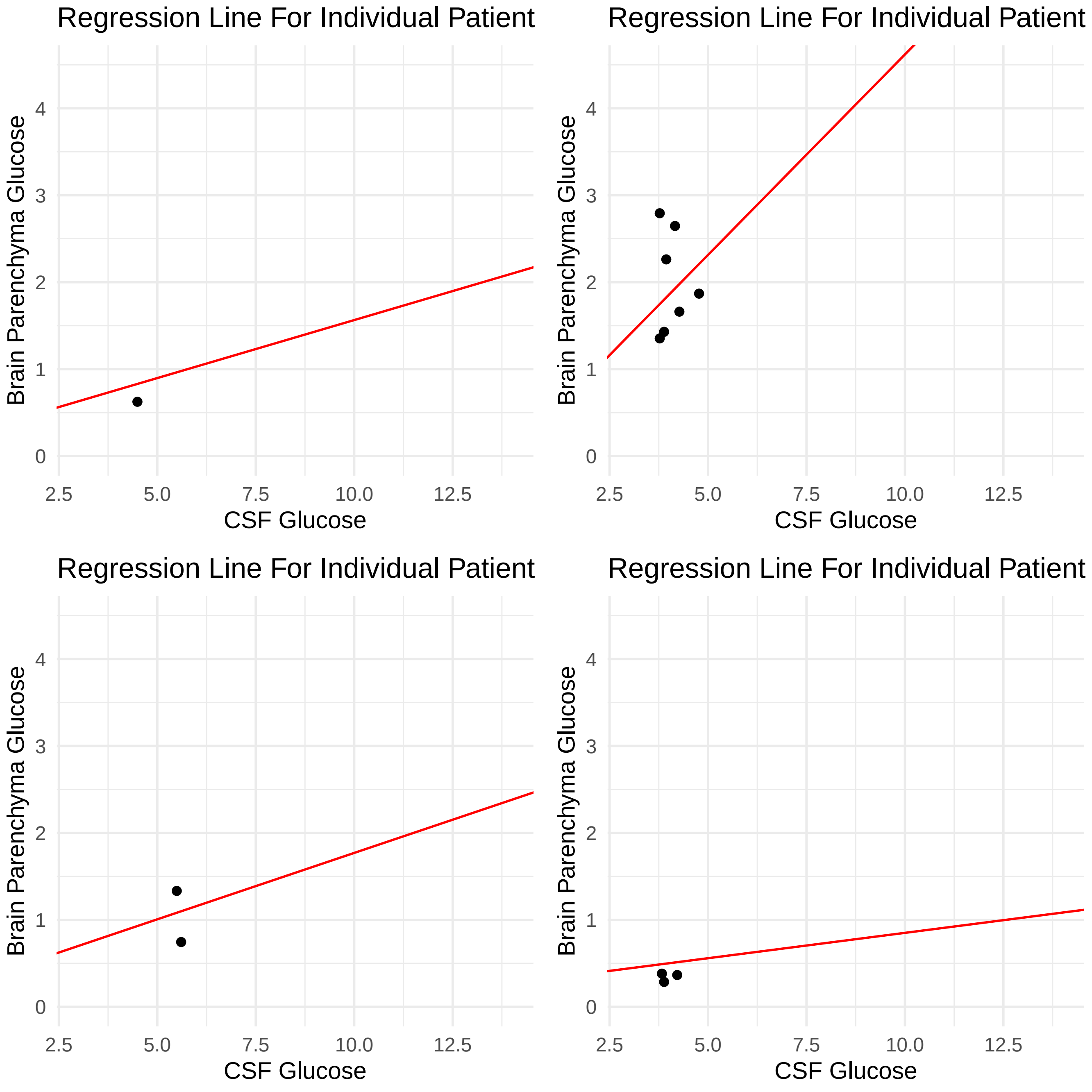
Regression Line For Individual Patient
Regression Line For Individual Patient
Regression Line For Individual Patient
Regression Line For Individual Patient
Brain Parenchyma Glucose
CSF Glucose
0
1
2
3
4
2.5
5.0
7.5
10.0
12.5

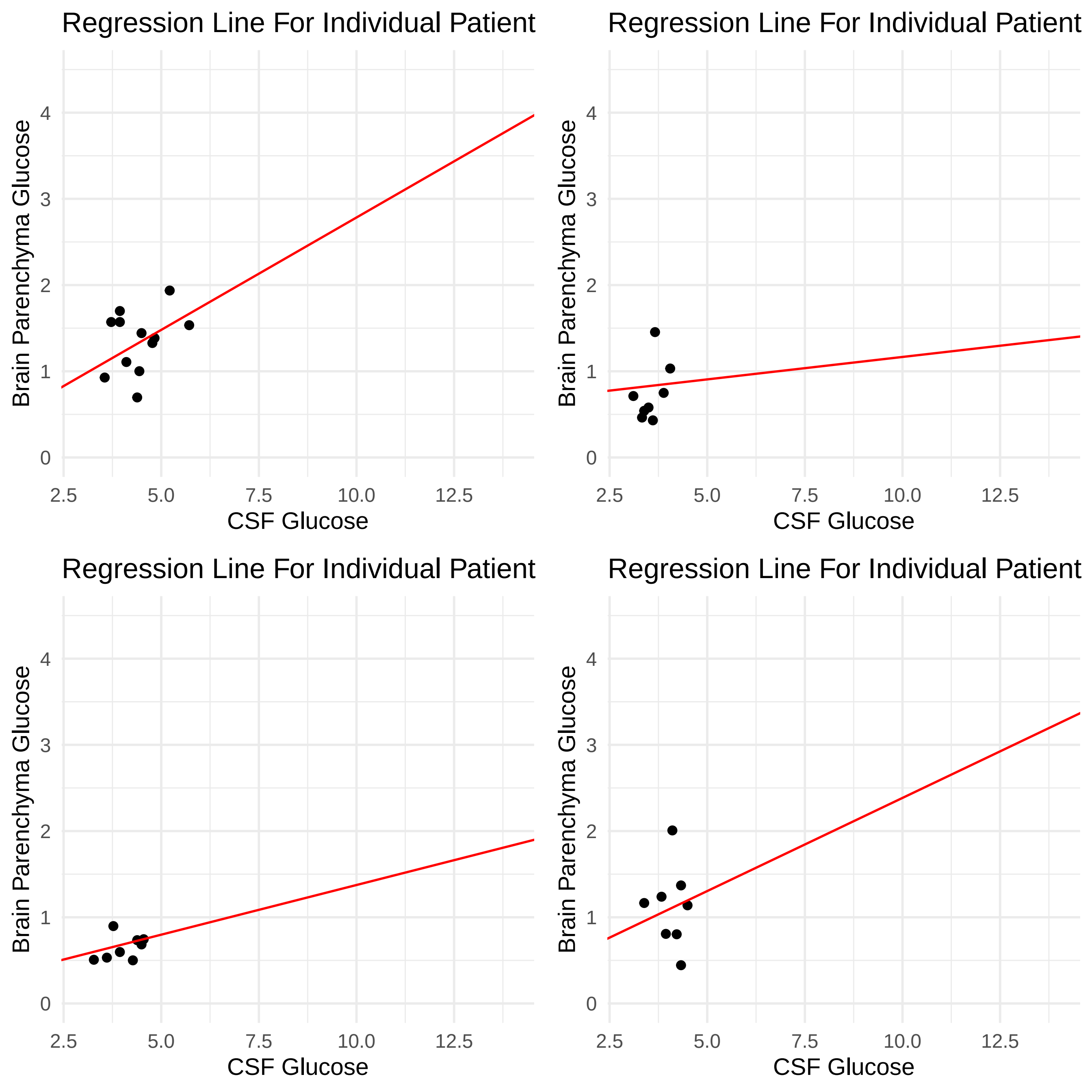
Regression Line For Individual Patient
Brain Parenchyma Glucose
CSF Glucose
0
1
2
3
4
2.5
5.0
7.5
10.0
12.5
Regression Line For Individual Patient
Brain Parenchyma Glucose
CSF Glucose
Regression Line For Individual Patient
Brain Parenchyma Glucose
CSF Glucose
Regression Line For Individual Patient
Brain Parenchyma Glucose
CSF Glucose

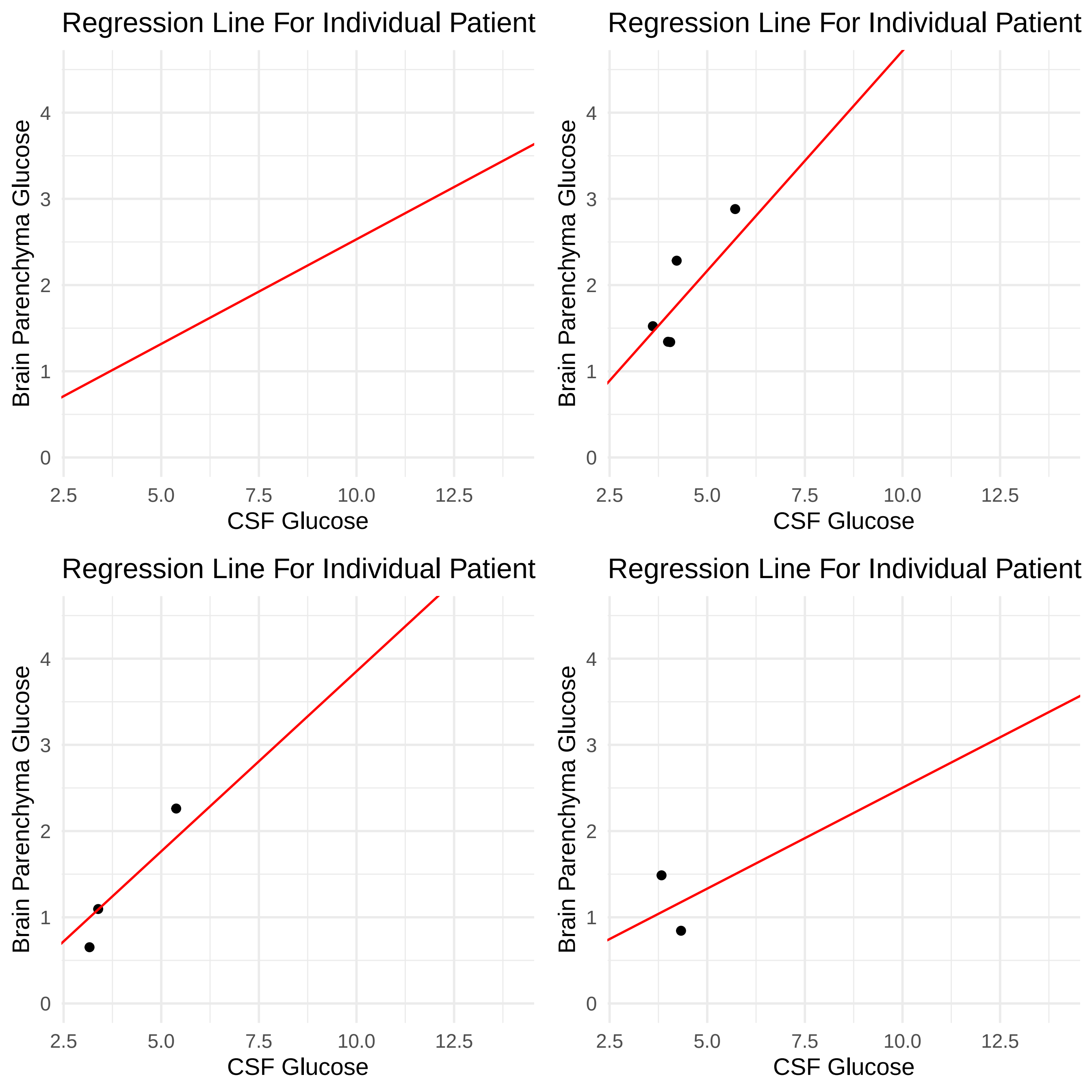

Regression Line For Individual Patient
Brain Parenchyma Glucose
CSF Glucose
0
1
2
3
4
2.5
5.0
7.5
10.0
12.5
Regression Line For Individual Patient
Brain Parenchyma Glucose
CSF Glucose
Regression Line For Individual Patient
Brain Parenchyma Glucose
CSF Glucose
Regression Line For Individual Patient
Brain Parenchyma Glucose
CSF Glucose

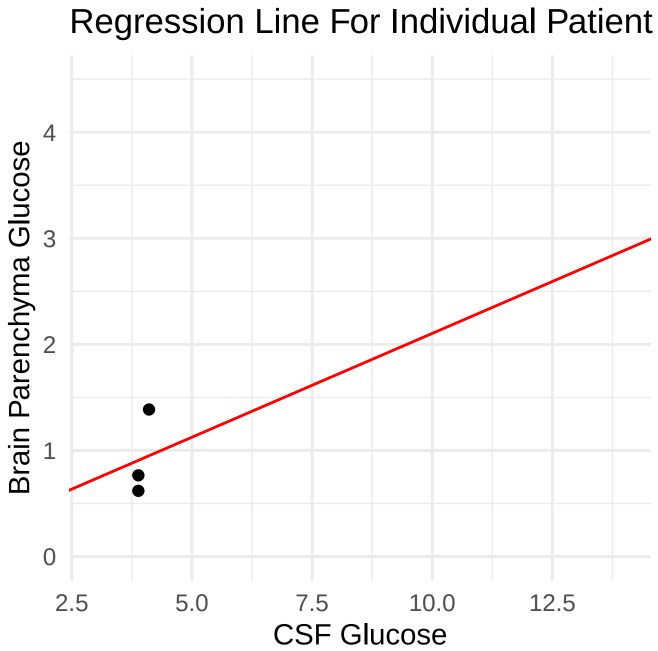

Regression Line For Individual Patient
Brain Parenchyma Glucose
0
1
2
3
4
2.5
5.0
7.5
10.0
12.5
CSF Glucose


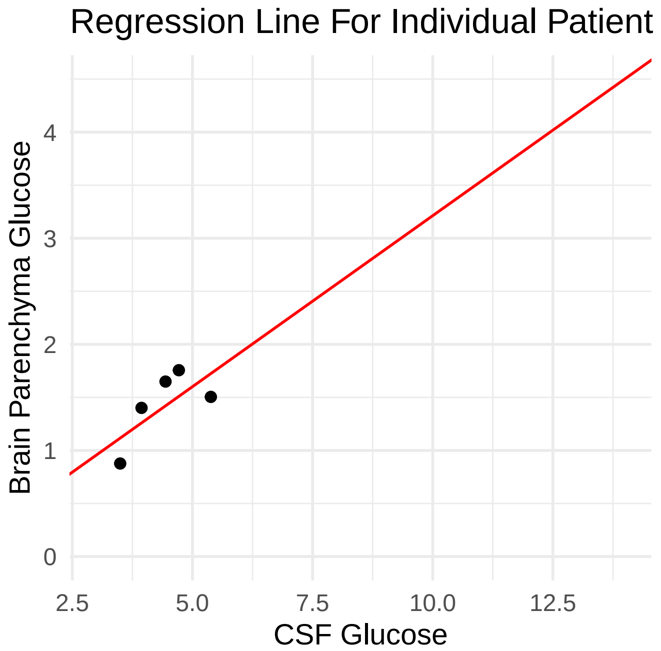

Regression Line For Individual Patient
Brain Parenchyma Glucose
0
1
2
3
4
2.5
5.0
7.5
10.0
12.5
CSF Glucose


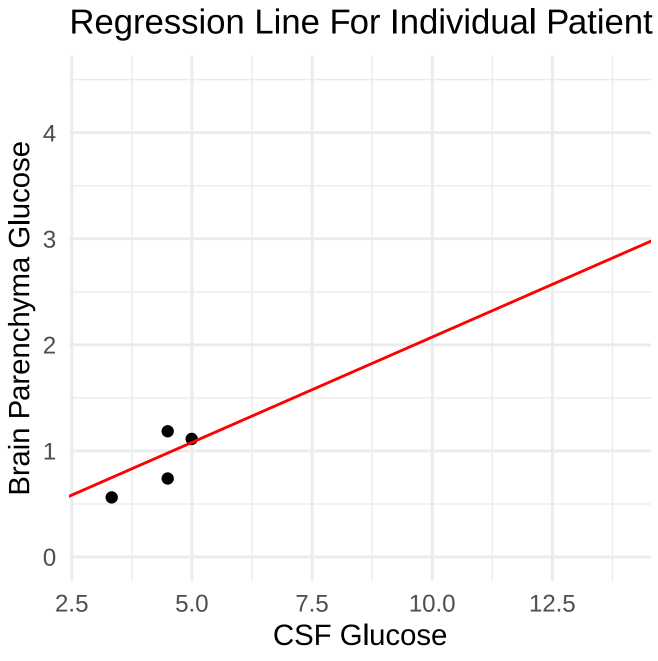

Regression Line For Individual Patient
Brain Parenchyma Glucose
0
1
2
3
4
2.5
5.0
7.5
10.0
12.5
CSF Glucose


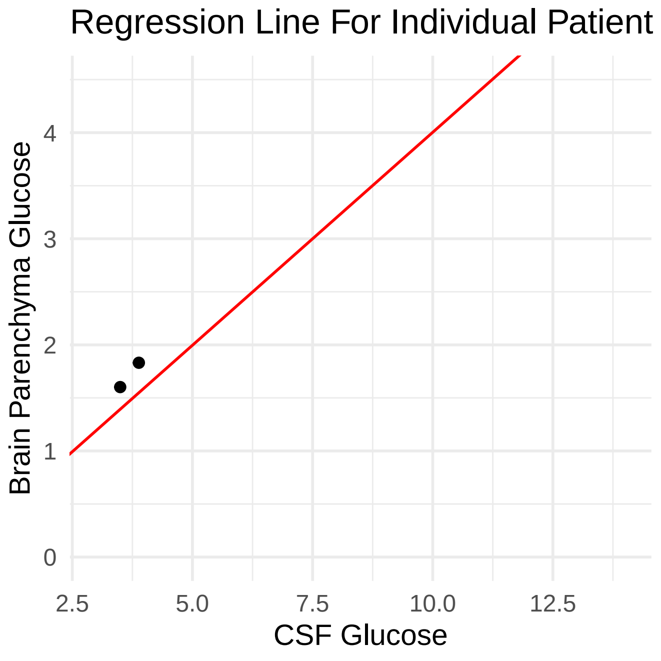

Regression Line For Individual Patient
Brain Parenchyma Glucose
0
1
2
3
4
2.5
5.0
7.5
10.0
12.5
CSF Glucose

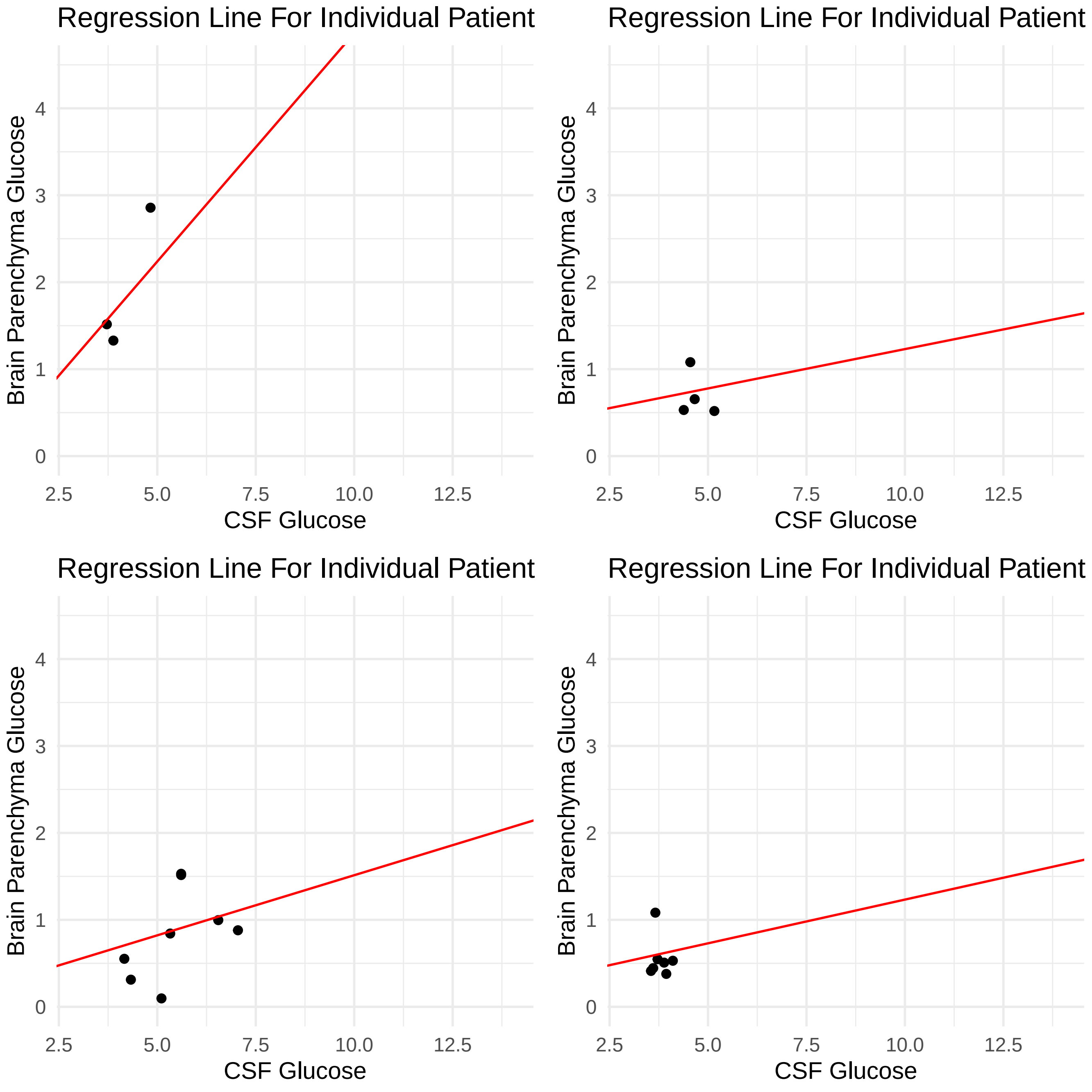
Regression Line For Individual Patient
Brain Parenchyma Glucose
CSF Glucose
0
1
2
3
4
2.5
5.0
7.5
10.0
12.5
Regression Line For Individual Patient
Brain Parenchyma Glucose
CSF Glucose
0
1
2
3
4
2.5
5.0
7.5
10.0
12.5
Regression Line For Individual Patient
Brain Parenchyma Glucose
CSF Glucose
0
1
2
3
4
2.5
5.0
7.5
10.0
12.5
Regression Line For Individual Patient
Brain Parenchyma Glucose
CSF Glucose
0
1
2
3
4
2.5
5.0
7.5
10.0
12.5

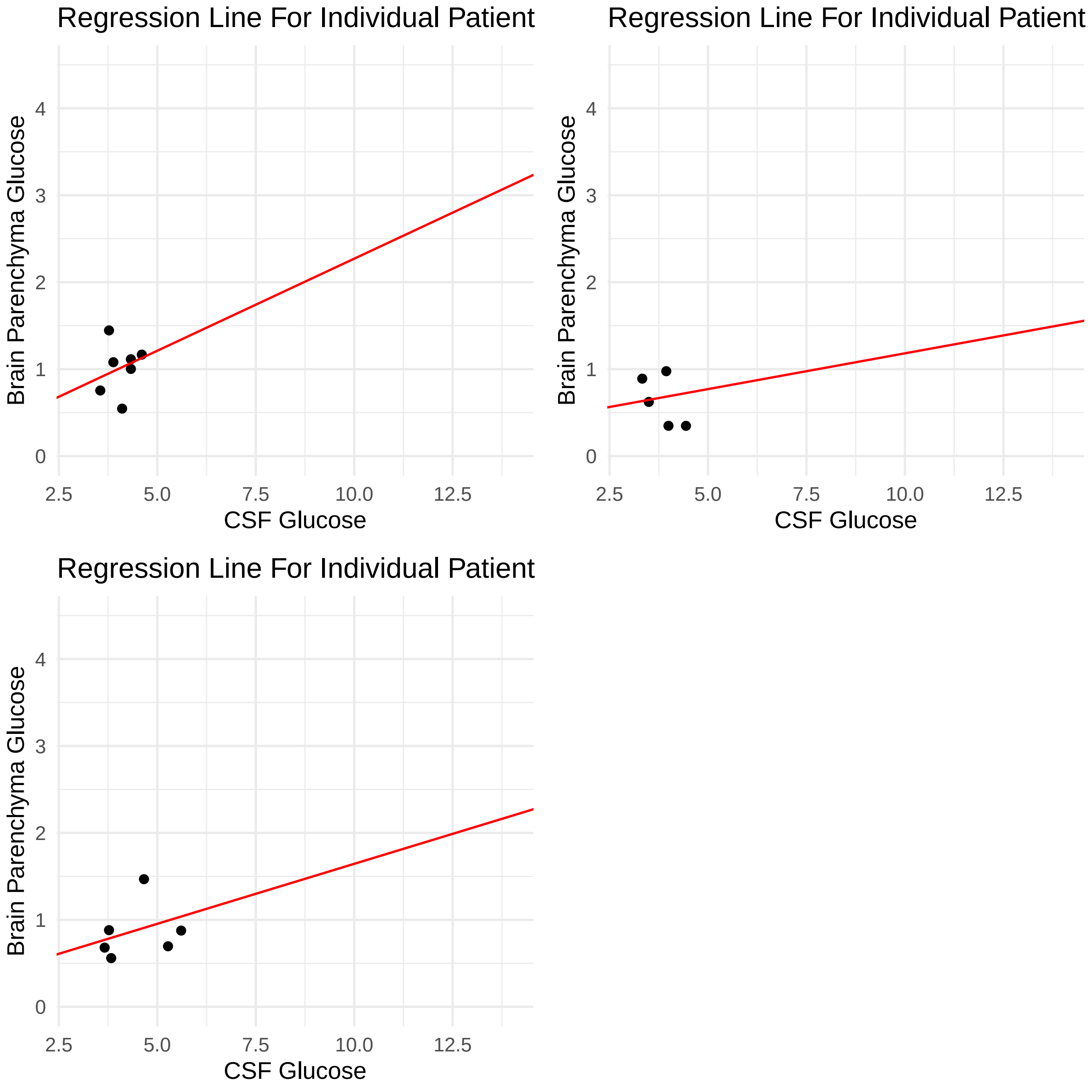
Regression Line For Individual Patient
Brain Parenchyma Glucose
CSF Glucose
0
1
2
3
4
2.5
5.0
7.5
10.0
12.5
Regression Line For Individual Patient
Brain Parenchyma Glucose
CSF Glucose
0
1
2
3
4
2.5
5.0
7.5
10.0
12.5
Regression Line For Individual Patient
Brain Parenchyma Glucose
CSF Glucose
0
1
2
3
4
2.5
5.0
7.5
10.0
12.5

## 5.2 CSF Lactate vs. Brain Parenchyma Lactate

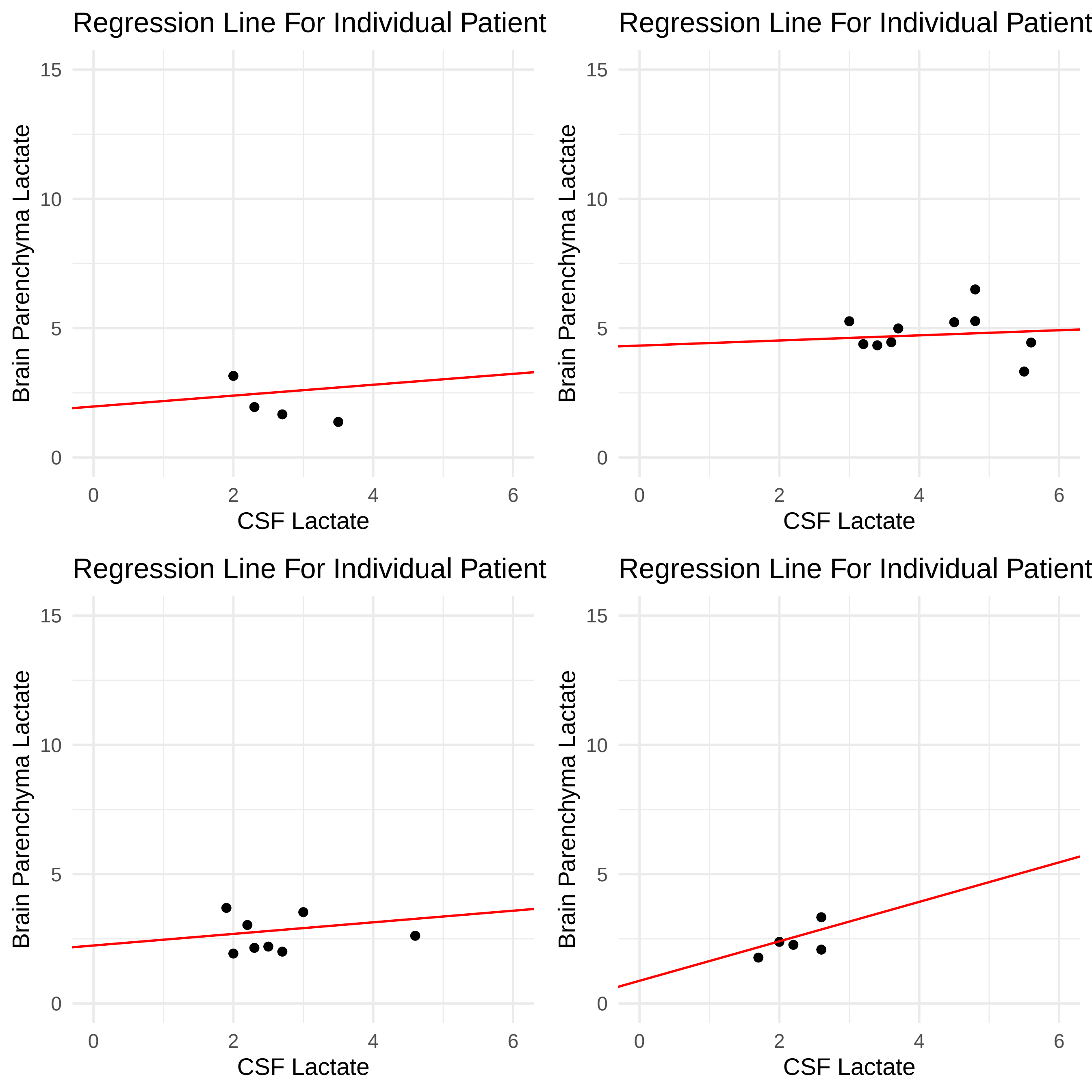
Regression Line For Individual Patient
Brain Parenchyma Lactate
CSF Lactate
0
5
10
15
0
2
4
6
Regression Line For Individual Patient
Brain Parenchyma Lactate
CSF Lactate
Regression Line For Individual Patient
Brain Parenchyma Lactate
CSF Lactate
Regression Line For Individual Patient
Brain Parenchyma Lactate
CSF Lactate

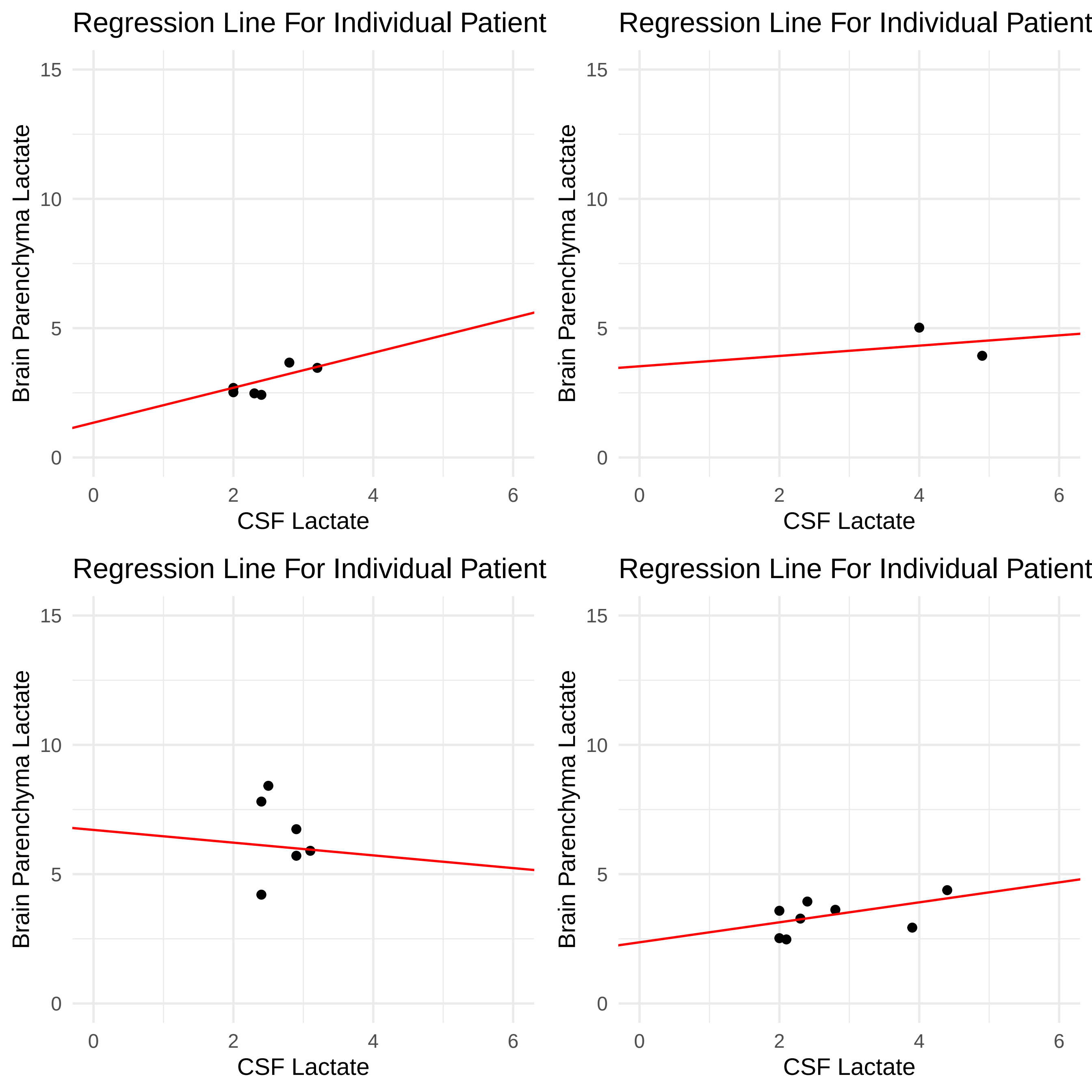
Regression Line For Individual Patient
Regression Line For Individual Patient
Regression Line For Individual Patient
Regression Line For Individual Patient
Brain Parenchyma Lactate
CSF Lactate
0
5
10
15
0
2
4
6

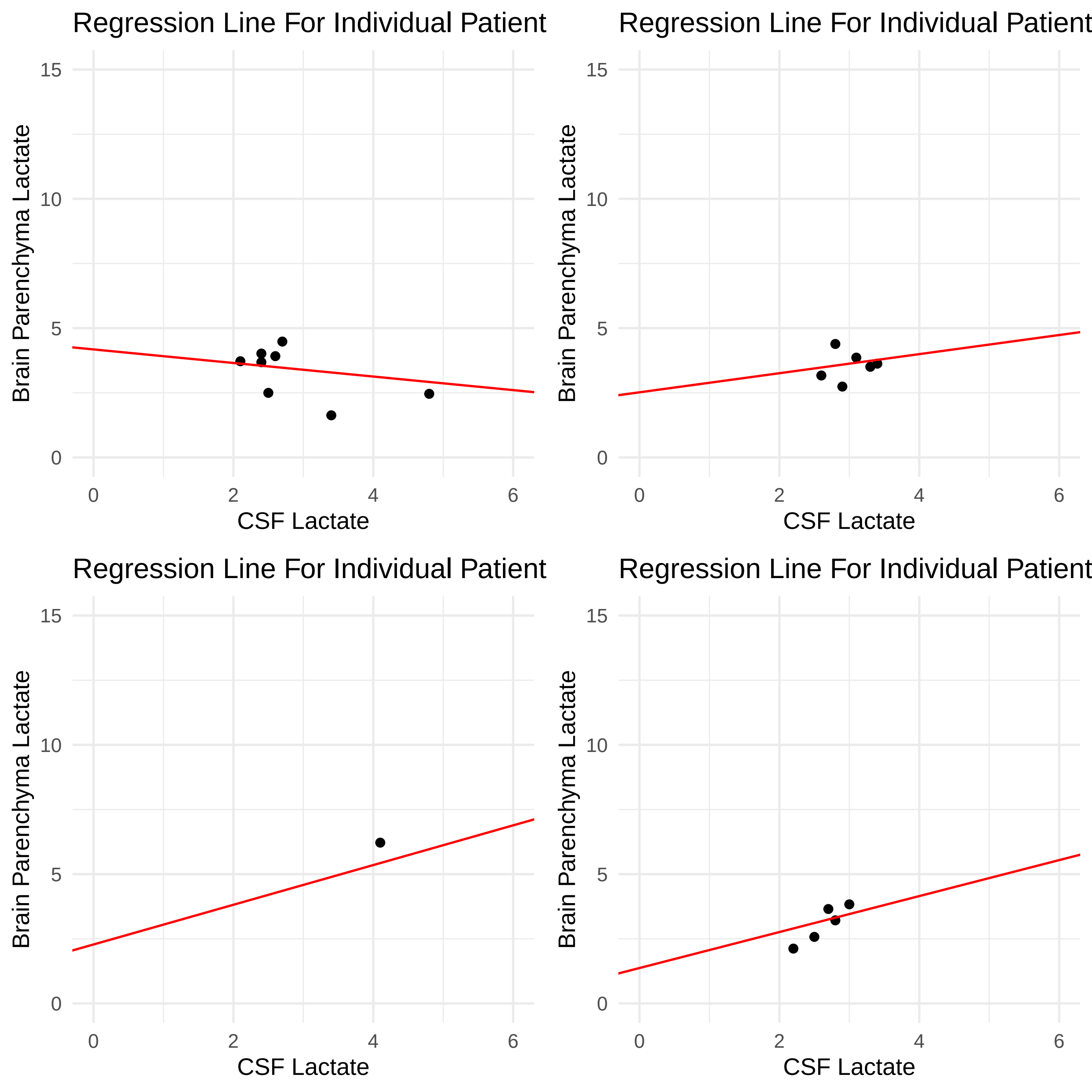
Regression Line For Individual Patient
Brain Parenchyma Lactate
CSF Lactate
0
5
10
15
0
2
4
6
Regression Line For Individual Patient
Brain Parenchyma Lactate
CSF Lactate
Regression Line For Individual Patient
Brain Parenchyma Lactate
CSF Lactate
Regression Line For Individual Patient
Brain Parenchyma Lactate
CSF Lactate

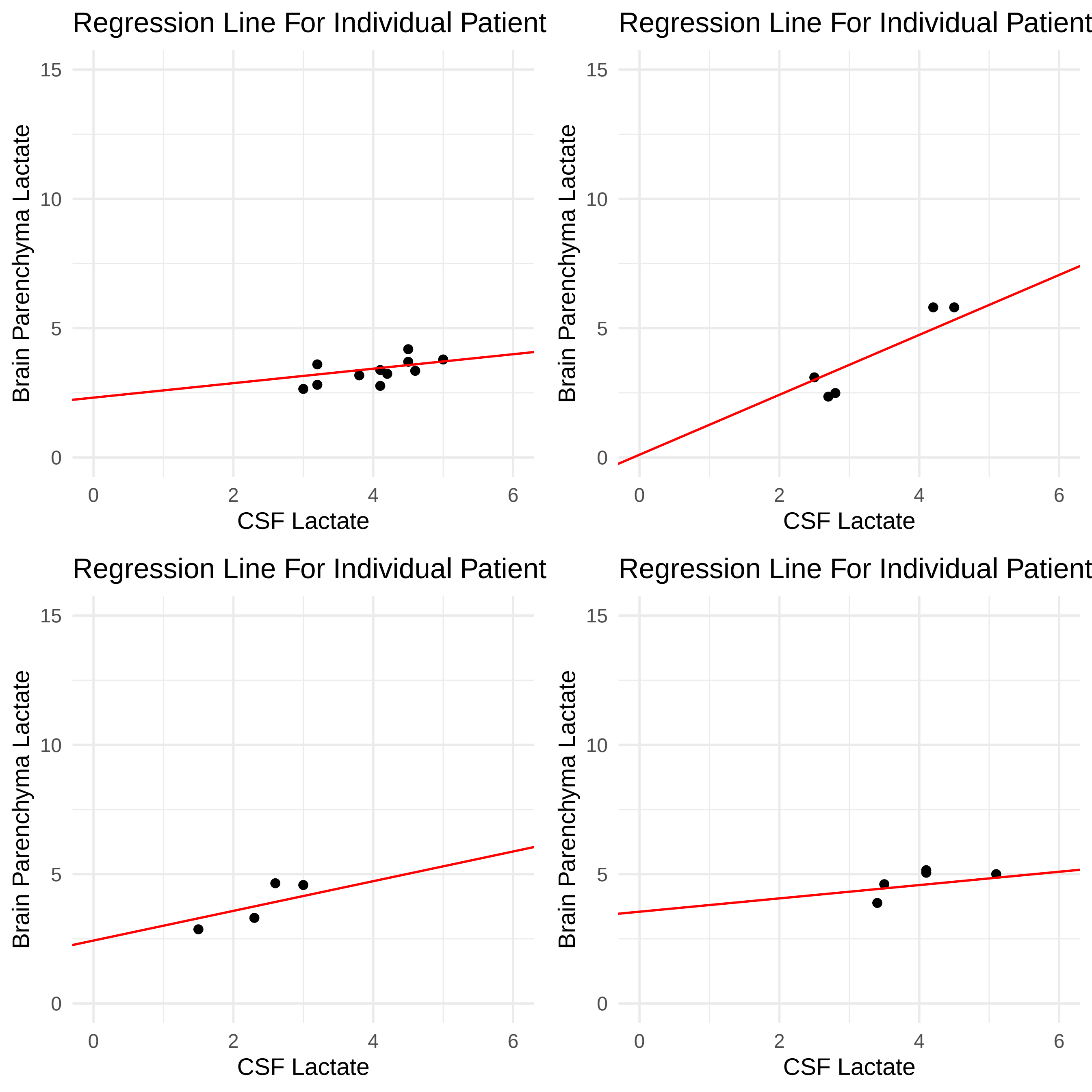

Regression Line For Individual Patient
Brain Parenchyma Lactate
CSF Lactate
15
10
5
0
0
2
4
6
Regression Line For Individual Patient
Brain Parenchyma Lactate
CSF Lactate
15
10
5
0
0
2
4
6
Regression Line For Individual Patient
Brain Parenchyma Lactate
CSF Lactate
15
10
5
0
0
2
4
6
Regression Line For Individual Patient
Brain Parenchyma Lactate
CSF Lactate
15
10
5
0
0
2
4
6

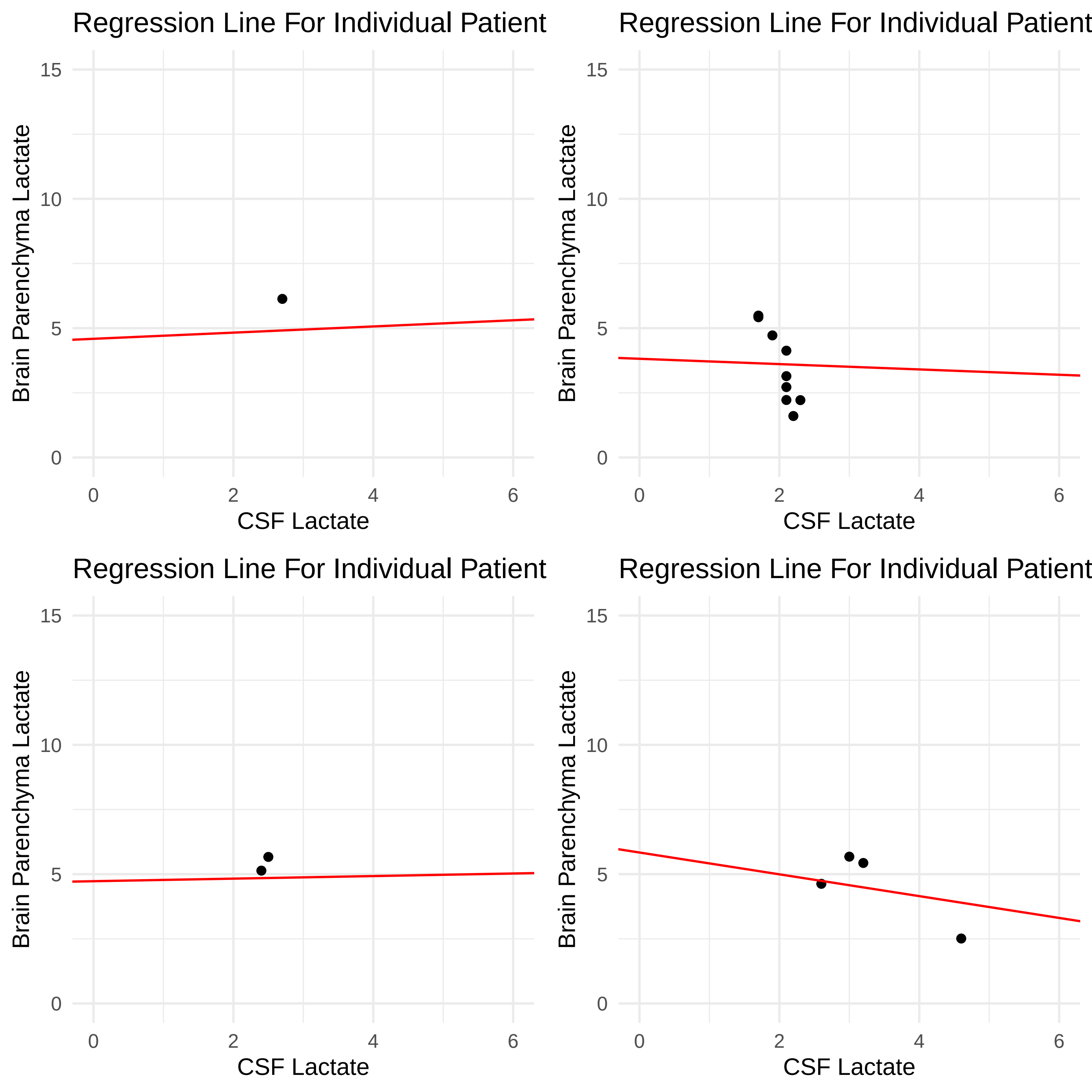

Regression Line For Individual Patient
Brain Parenchyma Lactate
CSF Lactate
0
5
10
15
0
2
4
6

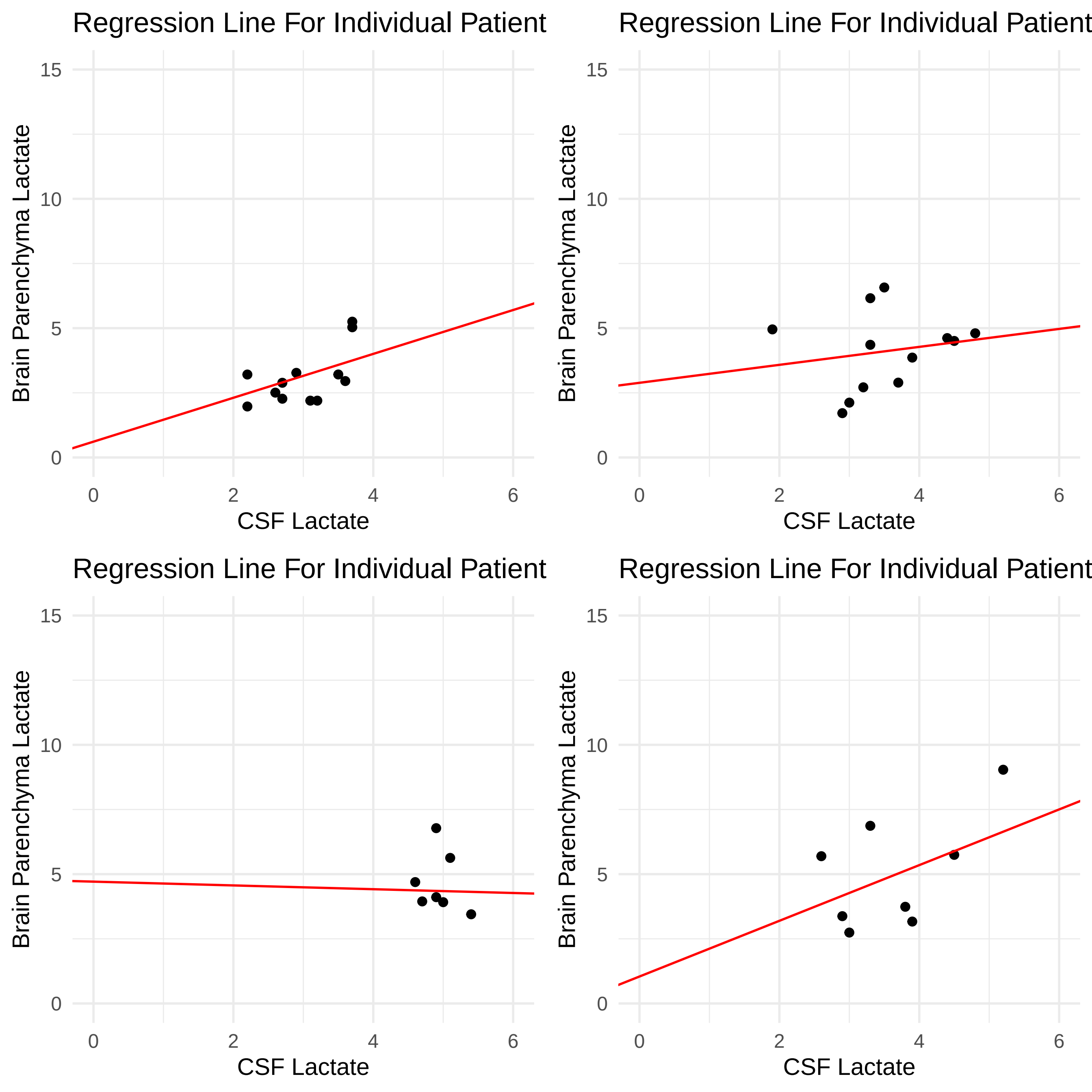
Regression Line For Individual Patient
Brain Parenchyma Lactate
CSF Lactate
Regression Line For Individual Patient
Brain Parenchyma Lactate
CSF Lactate
Regression Line For Individual Patient
Brain Parenchyma Lactate
CSF Lactate
Regression Line For Individual Patient
Brain Parenchyma Lactate
CSF Lactate

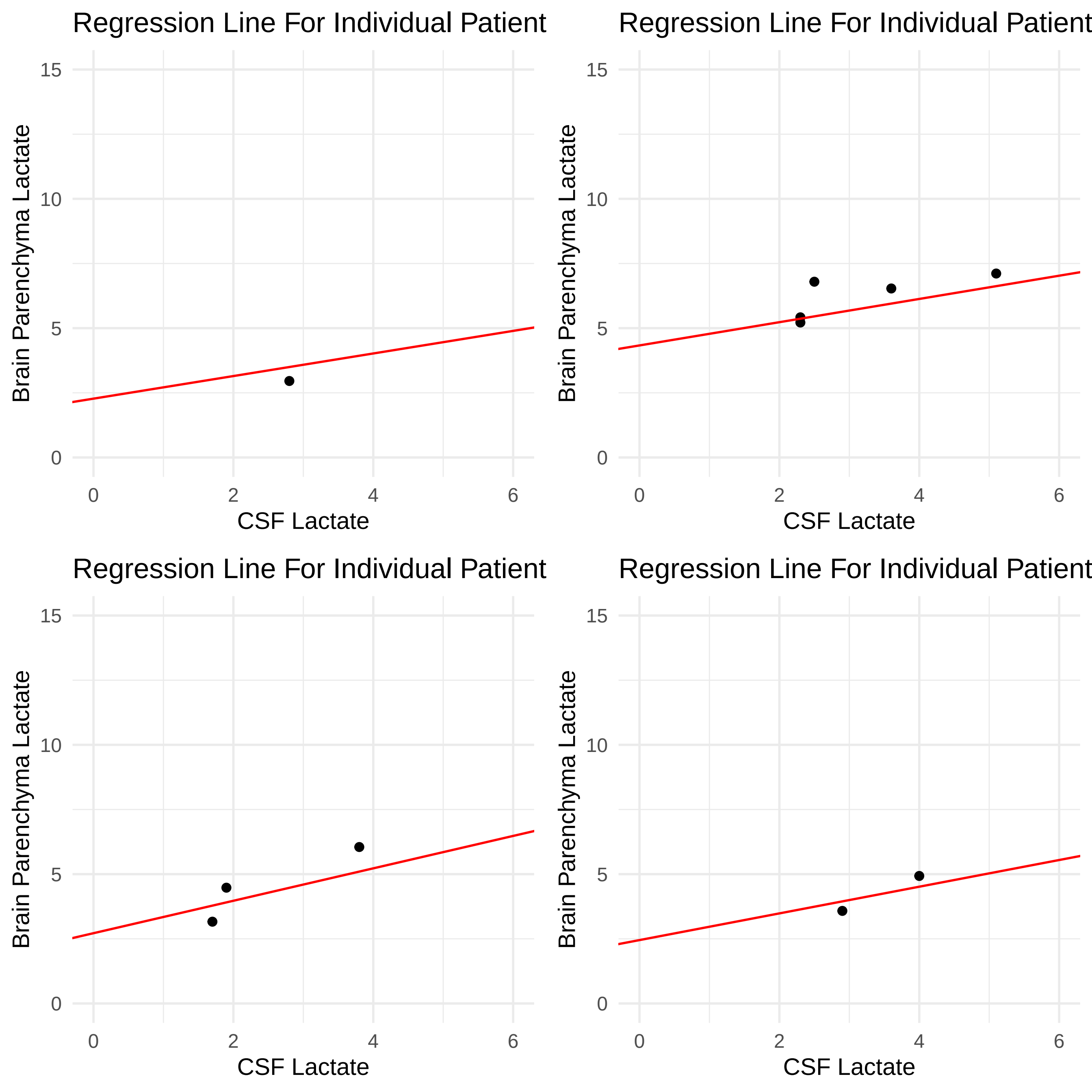

Regression Line For Individual Patient
Brain Parenchyma Lactate
CSF Lactate
0
5
10
15
0
2
4
6

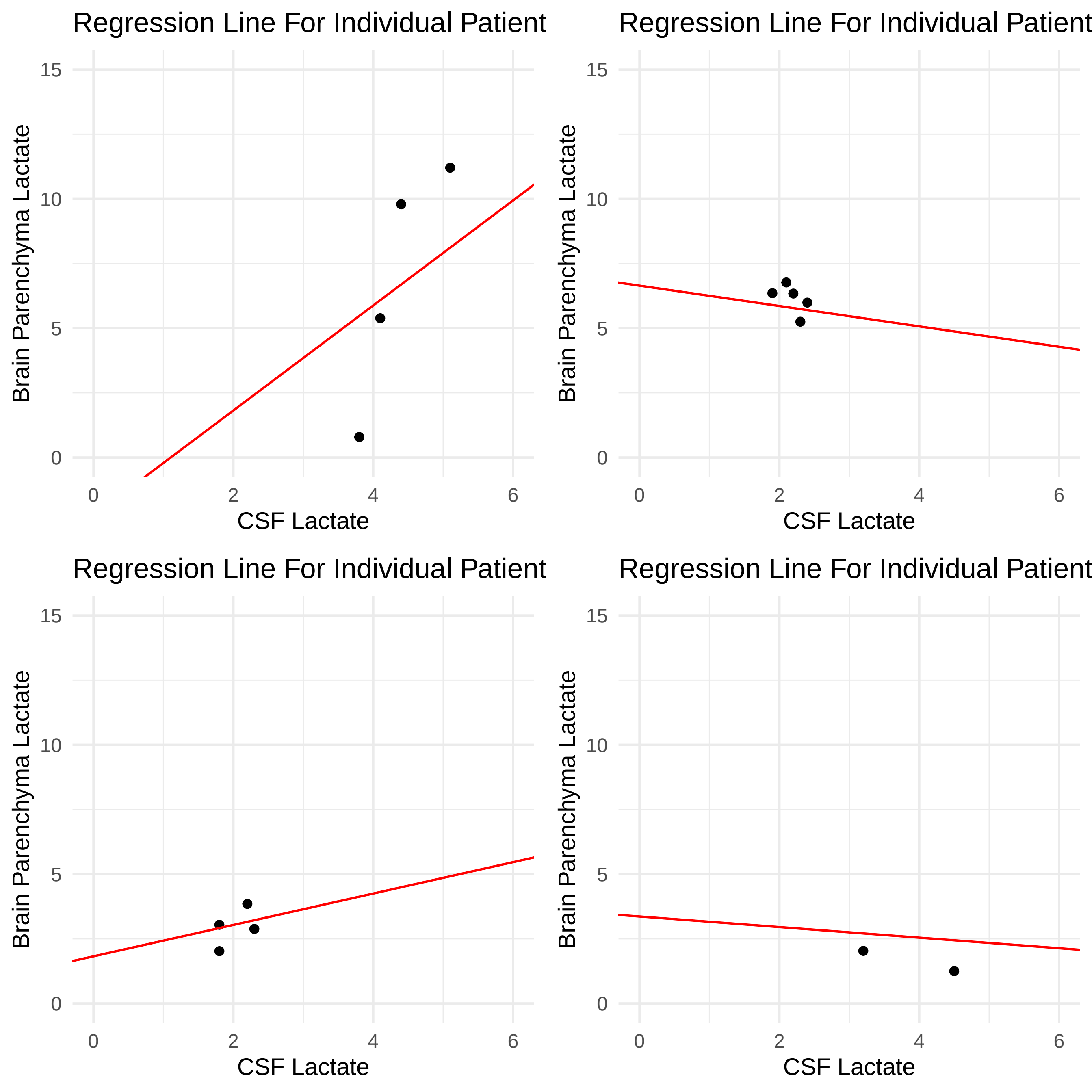
Regression Line For Individual Patient
Brain Parenchyma Lactate
CSF Lactate
0
5
10
15
0
2
4
6
Regression Line For Individual Patient
Brain Parenchyma Lactate
CSF Lactate
Regression Line For Individual Patient
Brain Parenchyma Lactate
CSF Lactate
Regression Line For Individual Patient
Brain Parenchyma Lactate
CSF Lactate

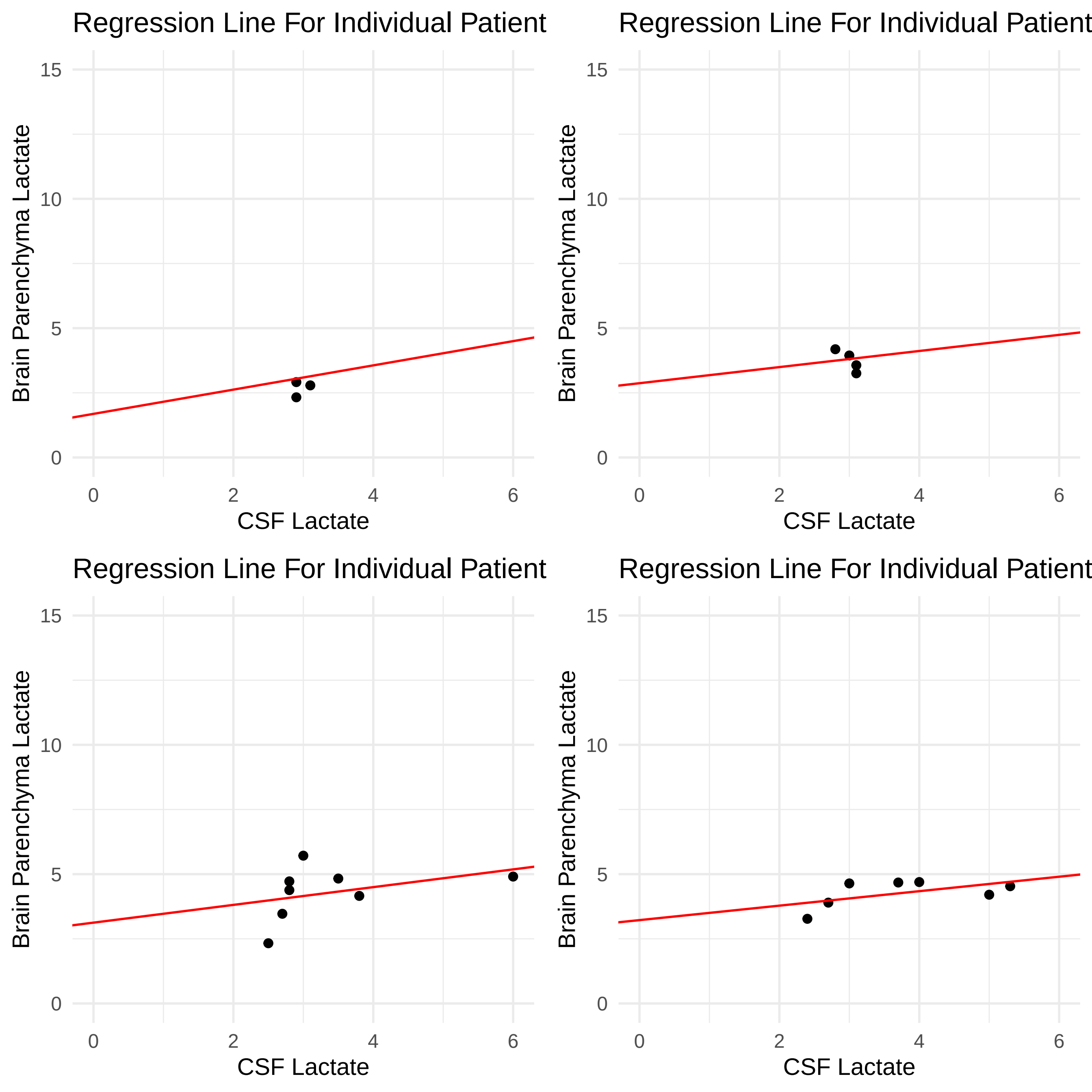
Regression Line For Individual Patient
Brain Parenchyma Lactate
CSF Lactate
0
5
10
15
0
2
4
6
Regression Line For Individual Patient
Brain Parenchyma Lactate
CSF Lactate
Regression Line For Individual Patient
Brain Parenchyma Lactate
CSF Lactate
Regression Line For Individual Patient
Brain Parenchyma Lactate
CSF Lactate

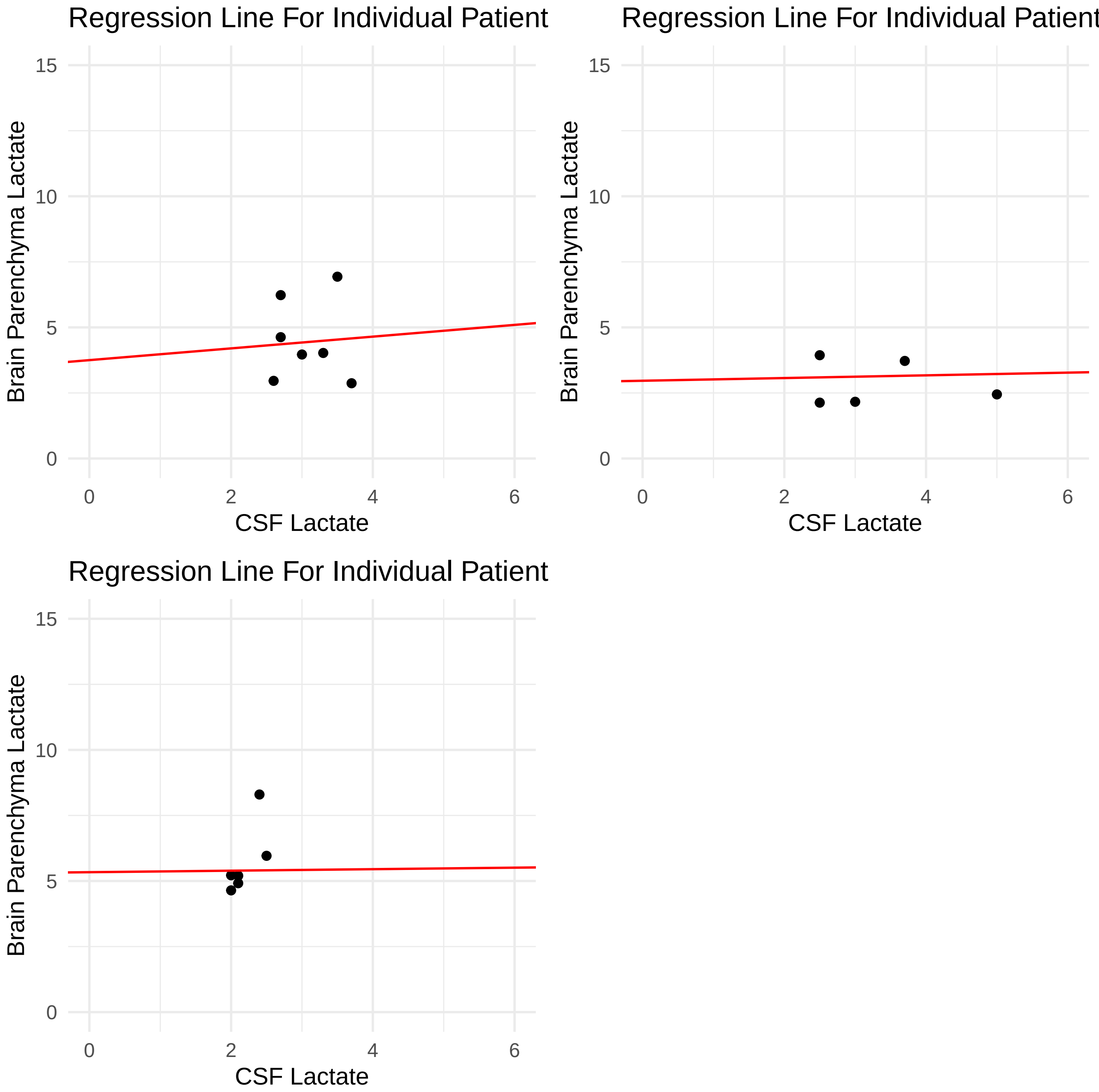
Regression Line For Individual Patient
Brain Parenchyma Lactate
CSF Lactate
0
5
10
15
0
2
4
6
Regression Line For Individual Patient
Brain Parenchyma Lactate
CSF Lactate
0
5
10
15
0
2
4
6
Regression Line For Individual Patient
Brain Parenchyma Lactate
CSF Lactate
0
5
10
15
0
2
4
6

## 5.3 Plasma Glucose vs. Brain Parenchyma Glucose

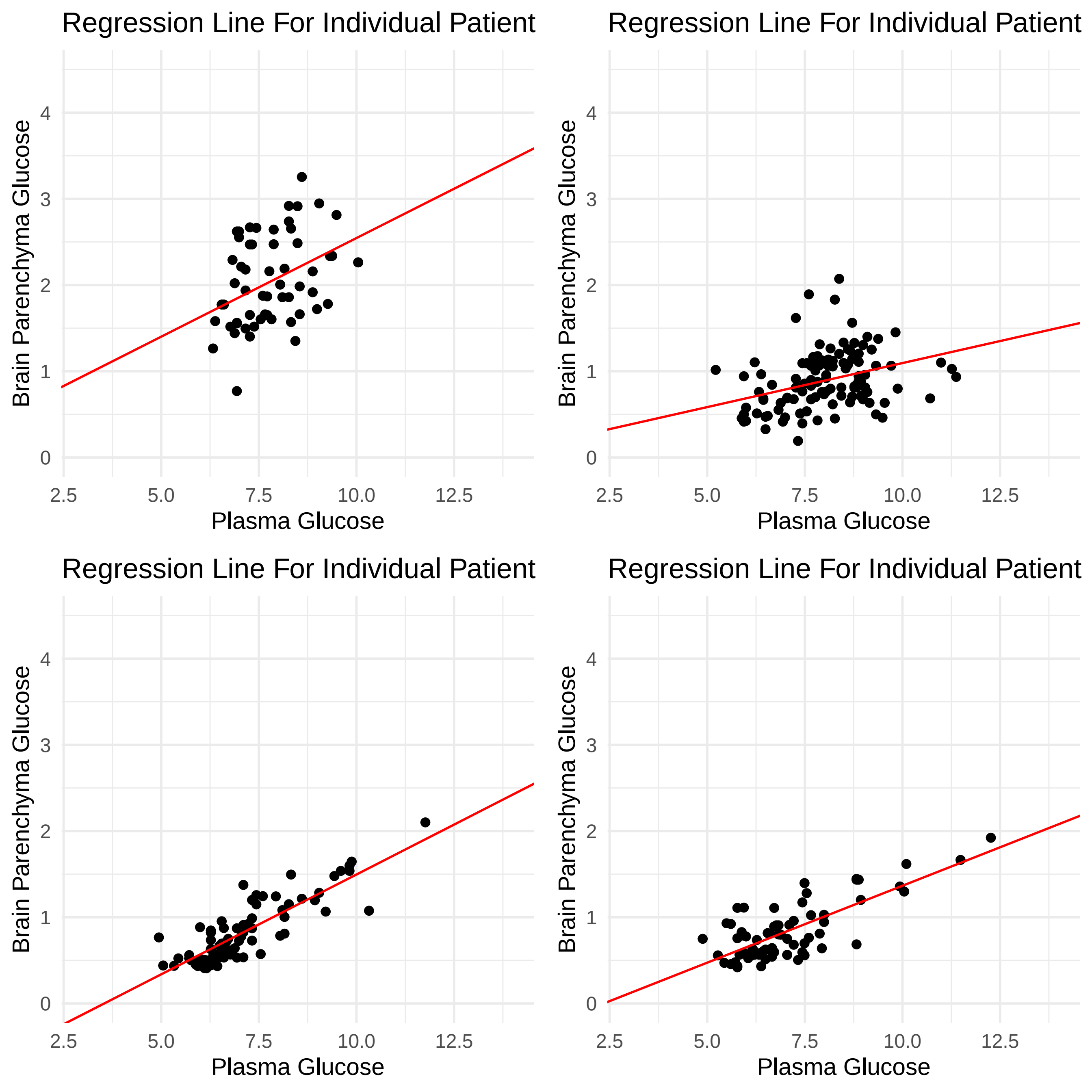
Regression Line For Individual Patient
Brain Parenchyma Glucose
Plasma Glucose
0
1
2
3
4
2.5
5.0
7.5
10.0
12.5
Regression Line For Individual Patient
Brain Parenchyma Glucose
Plasma Glucose
Regression Line For Individual Patient
Brain Parenchyma Glucose
Plasma Glucose
Regression Line For Individual Patient
Brain Parenchyma Glucose
Plasma Glucose

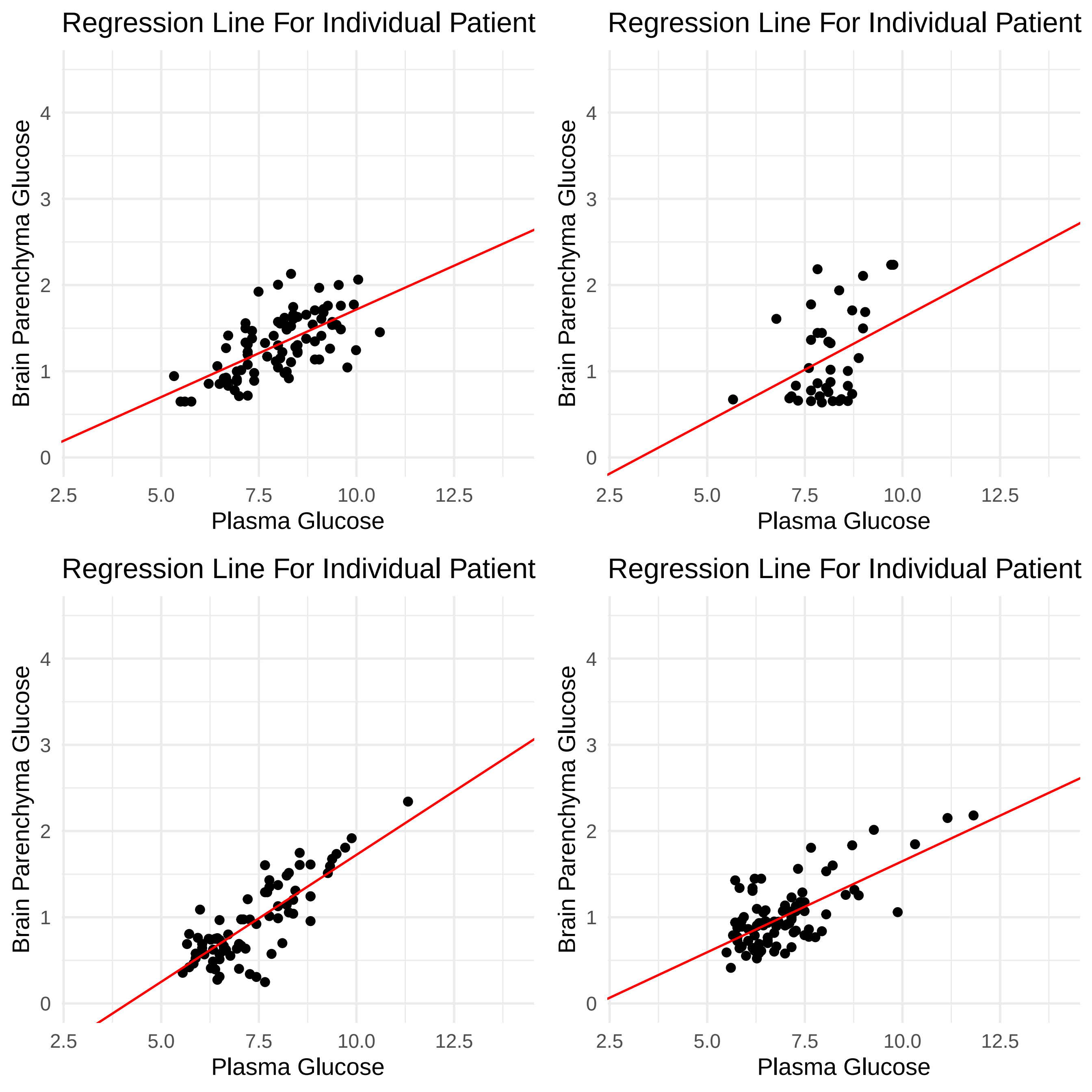
Regression Line For Individual Patient
Brain Parenchyma Glucose
Plasma Glucose
0
1
2
3
4
2.5
5.0
7.5
10.0
12.5
Regression Line For Individual Patient
Brain Parenchyma Glucose
Plasma Glucose
Regression Line For Individual Patient
Brain Parenchyma Glucose
Plasma Glucose
Regression Line For Individual Patient
Brain Parenchyma Glucose
Plasma Glucose

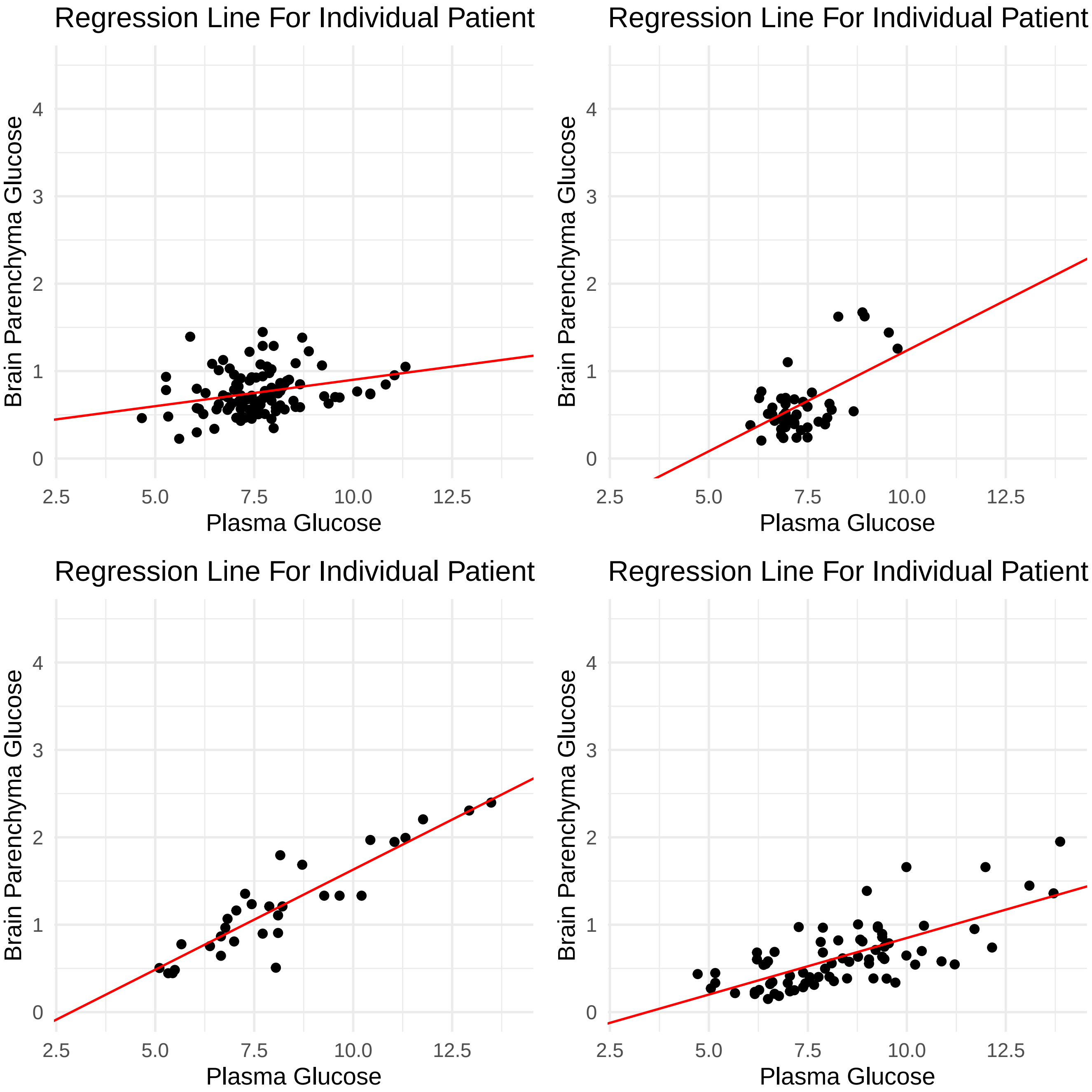

Regression Line For Individual Patient
Brain Parenchyma Glucose
0
1
2
3
4
2.5
5.0
7.5
10.0
12.5
Plasma Glucose
Regression Line For Individual Patient
Brain Parenchyma Glucose
0
1
2
3
4
2.5
5.0
7.5
10.0
12.5
Plasma Glucose
Regression Line For Individual Patient
Brain Parenchyma Glucose
0
1
2
3
4
2.5
5.0
7.5
10.0
12.5
Plasma Glucose
Regression Line For Individual Patient
Brain Parenchyma Glucose
0
1
2
3
4
2.5
5.0
7.5
10.0
12.5
Plasma Glucose

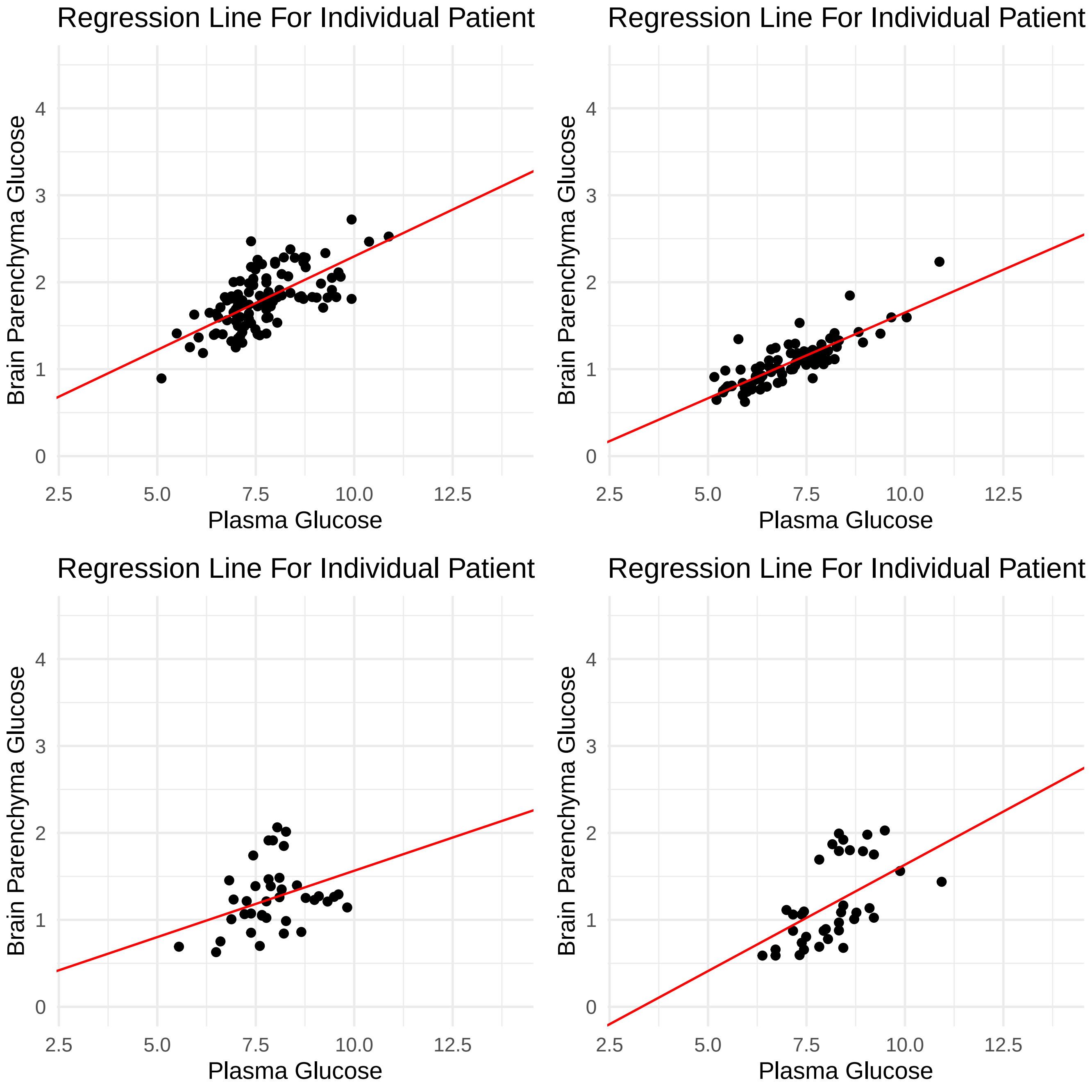
Regression Line For Individual Patient
Regression Line For Individual Patient
Regression Line For Individual Patient
Regression Line For Individual Patient
Brain Parenchyma Glucose
Plasma Glucose
0
1
2
3
4
2.5
5.0
7.5
10.0
12.5

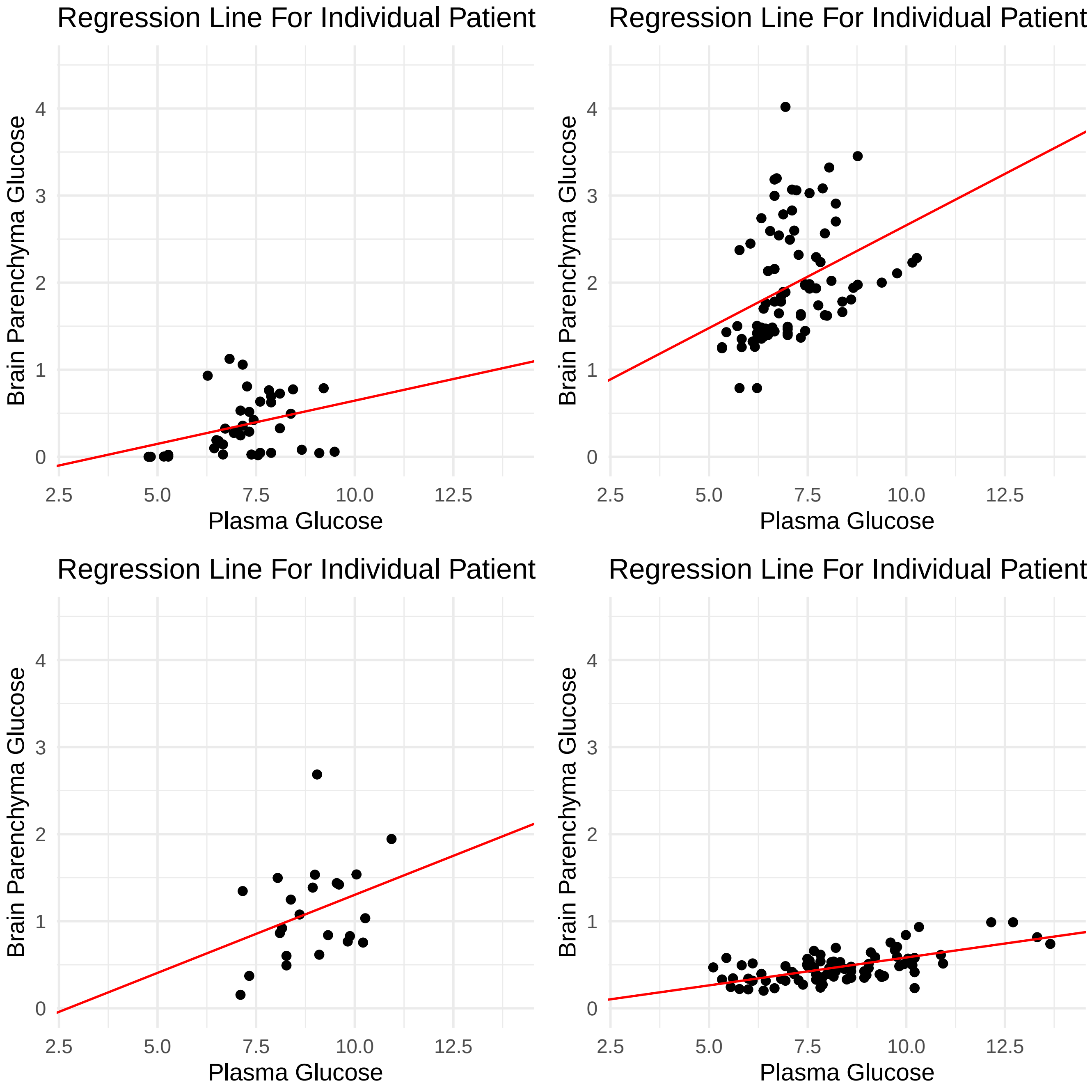
Regression Line For Individual Patient
Brain Parenchyma Glucose
Plasma Glucose
0
1
2
3
4
2.5
5.0
7.5
10.0
12.5
Regression Line For Individual Patient
Brain Parenchyma Glucose
Plasma Glucose
Regression Line For Individual Patient
Brain Parenchyma Glucose
Plasma Glucose
Regression Line For Individual Patient
Brain Parenchyma Glucose
Plasma Glucose

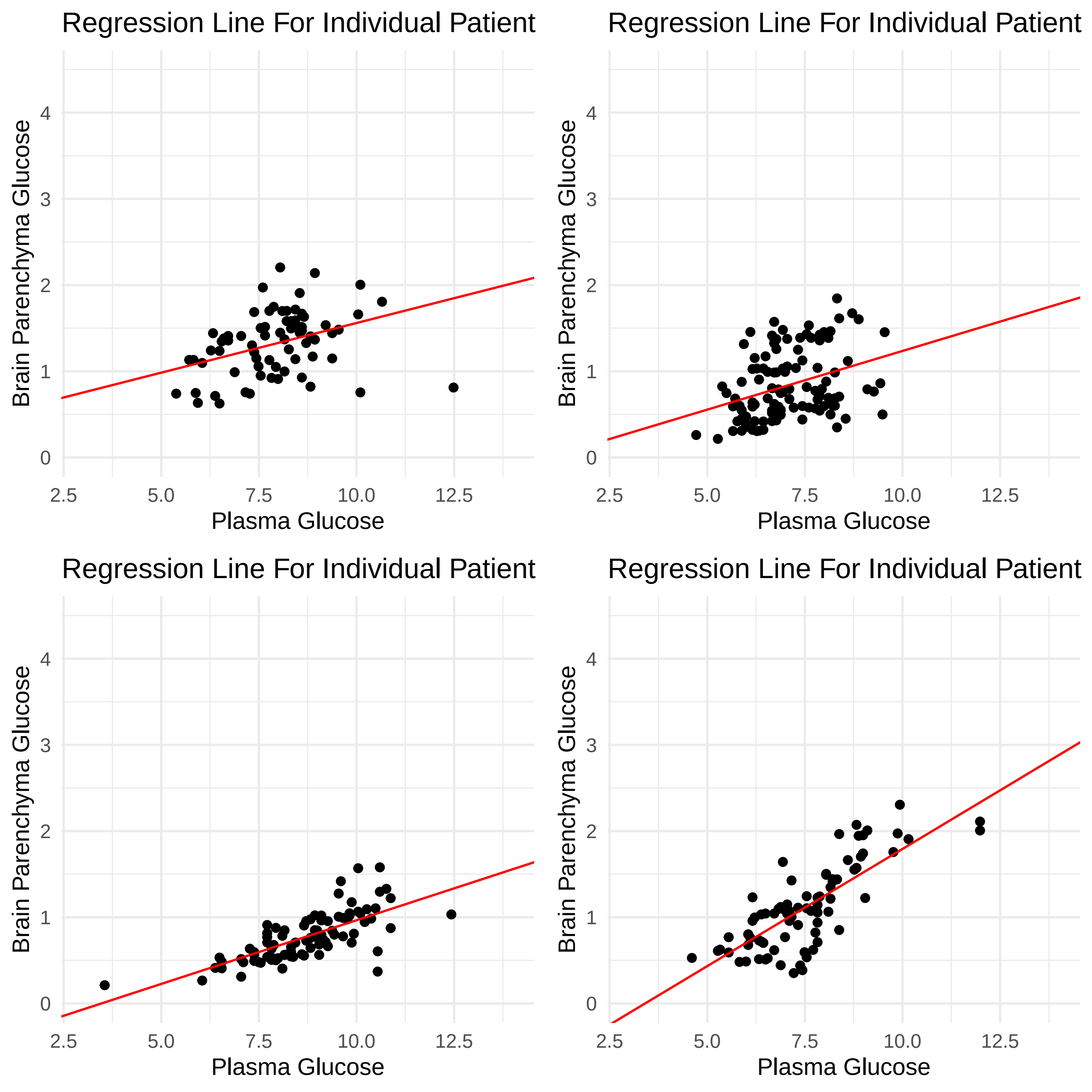
Regression Line For Individual Patient
Regression Line For Individual Patient
Regression Line For Individual Patient
Regression Line For Individual Patient
Brain Parenchyma Glucose
Plasma Glucose
0
1
2
3
4
2.5
5.0
7.5
10.0
12.5

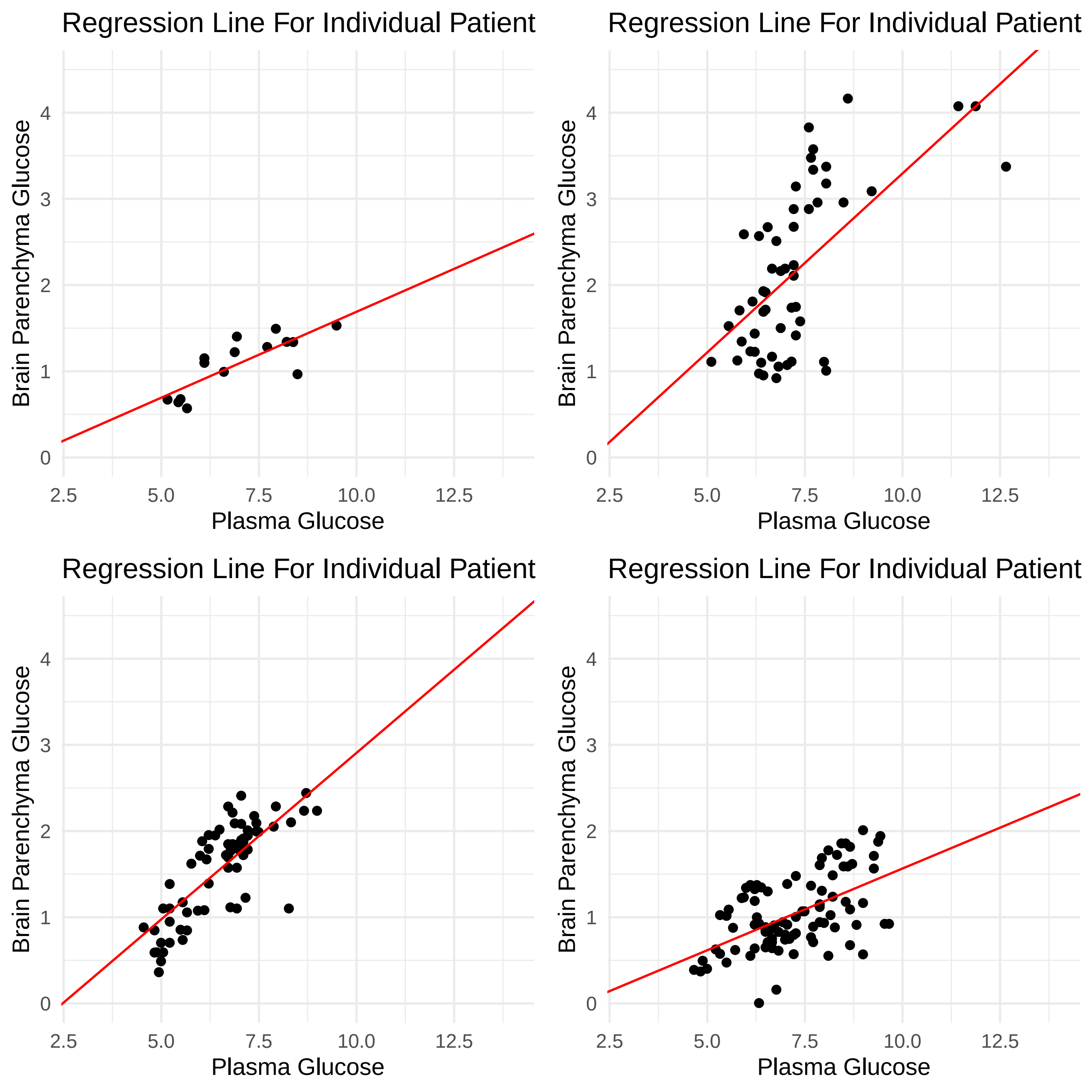
Regression Line For Individual Patient
Brain Parenchyma Glucose
Plasma Glucose
0
1
2
3
4
2.5
5.0
7.5
10.0
12.5
Regression Line For Individual Patient
Brain Parenchyma Glucose
Plasma Glucose
Regression Line For Individual Patient
Brain Parenchyma Glucose
Plasma Glucose
Regression Line For Individual Patient
Brain Parenchyma Glucose
Plasma Glucose

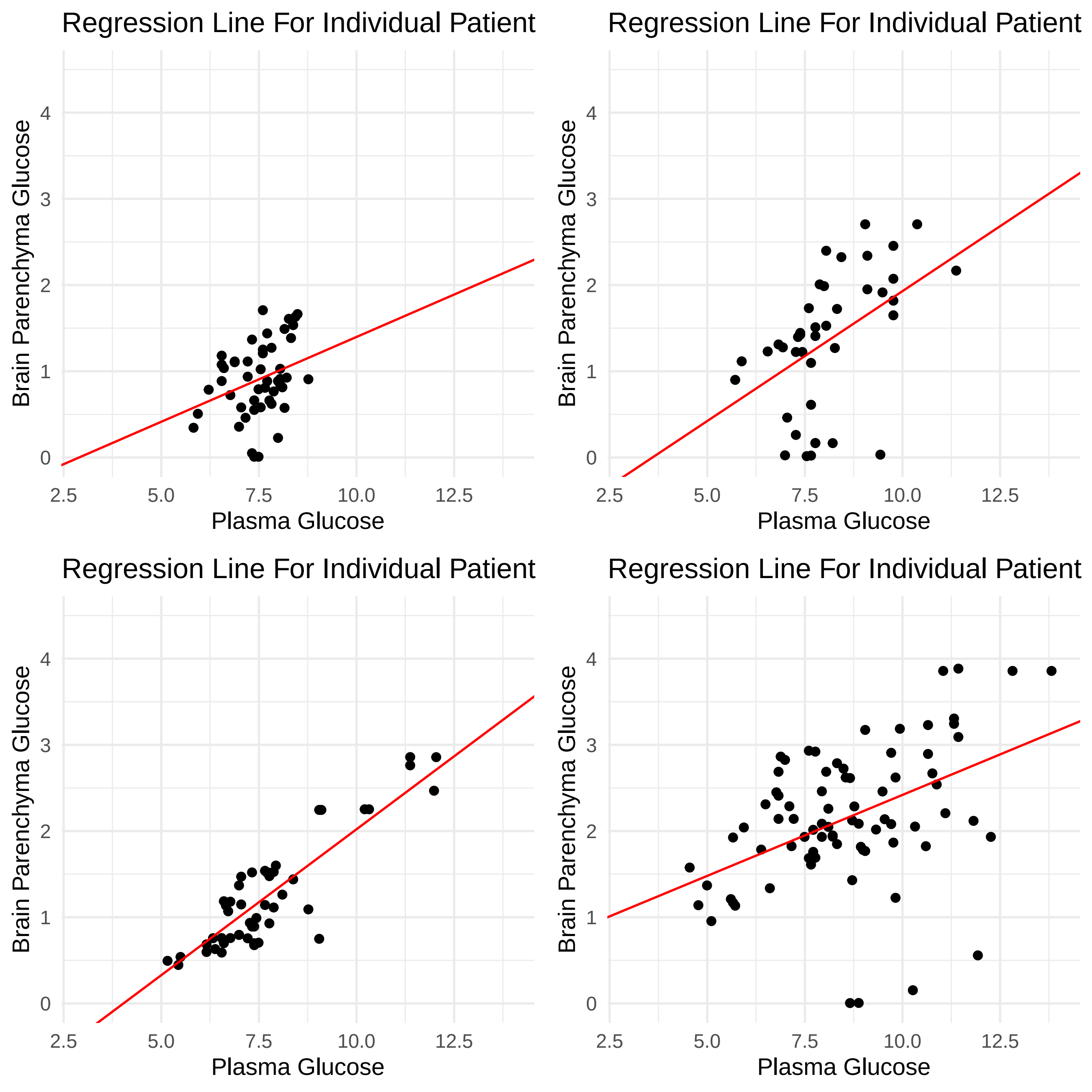

Regression Line For Individual Patient
Brain Parenchyma Glucose
Plasma Glucose
0
1
2
3
4
2.5
5.0
7.5
10.0
12.5

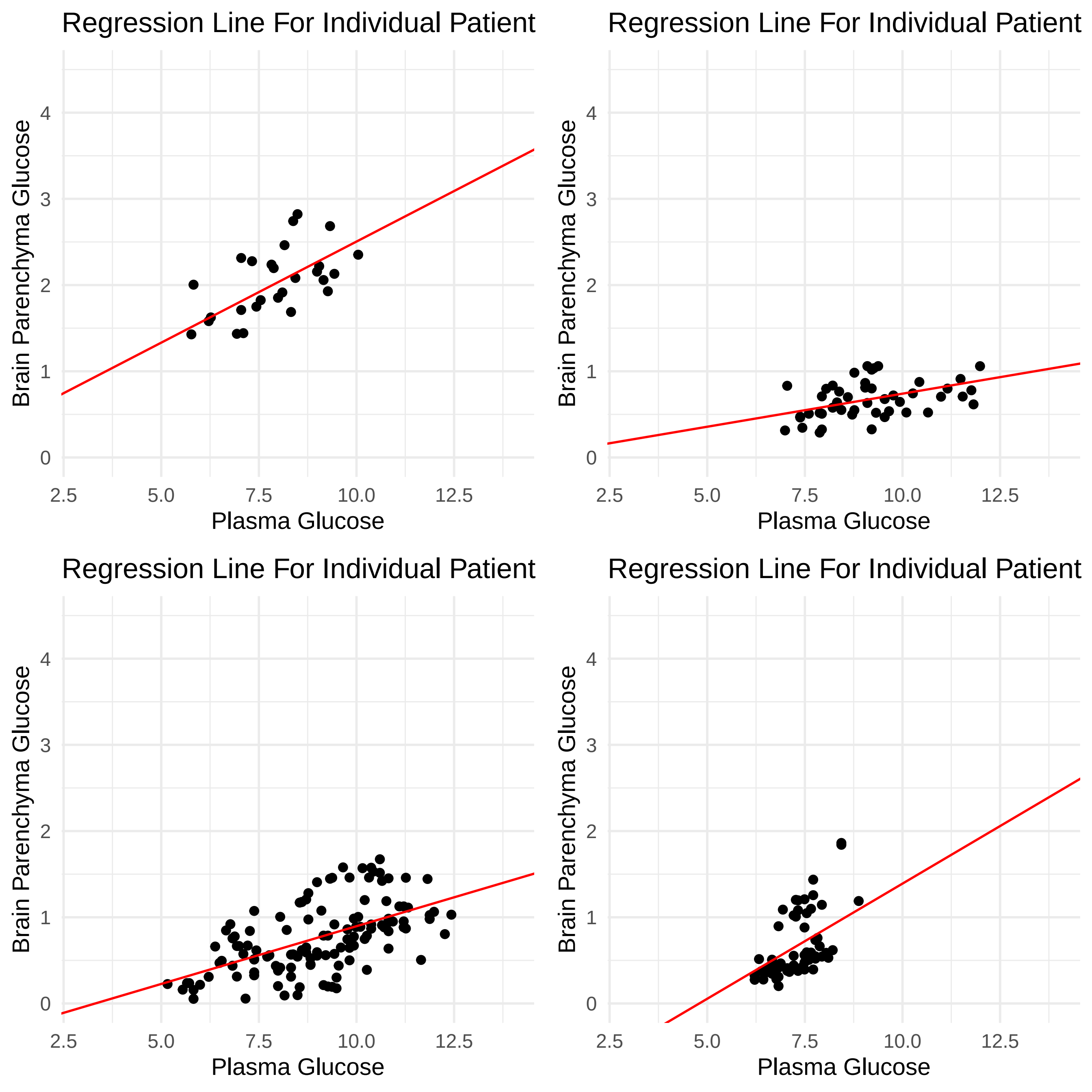
Regression Line For Individual Patient
Brain Parenchyma Glucose
Plasma Glucose
0
1
2
3
4
2.5
5.0
7.5
10.0
12.5
Regression Line For Individual Patient
Brain Parenchyma Glucose
Plasma Glucose
Regression Line For Individual Patient
Brain Parenchyma Glucose
Plasma Glucose
Regression Line For Individual Patient
Brain Parenchyma Glucose
Plasma Glucose

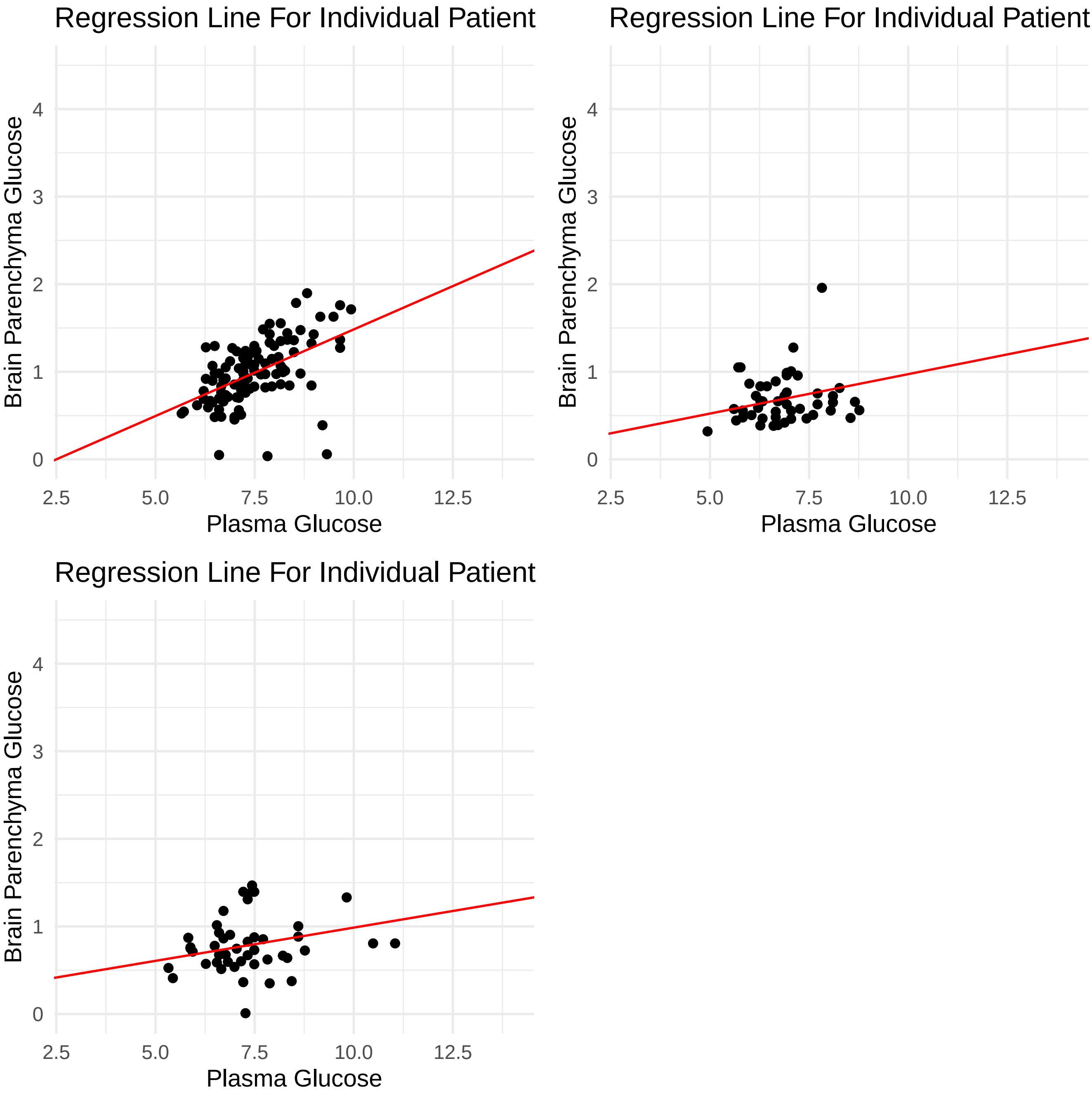
Regression Line For Individual Patient
Brain Parenchyma Glucose
Plasma Glucose
0
1
2
3
4
2.5
5.0
7.5
10.0
12.5
Regression Line For Individual Patient
Brain Parenchyma Glucose
Plasma Glucose
Regression Line For Individual Patient
Brain Parenchyma Glucose
Plasma Glucose

## 5.4 Plasma Lactate vs. Brain Parenchyma Lactate

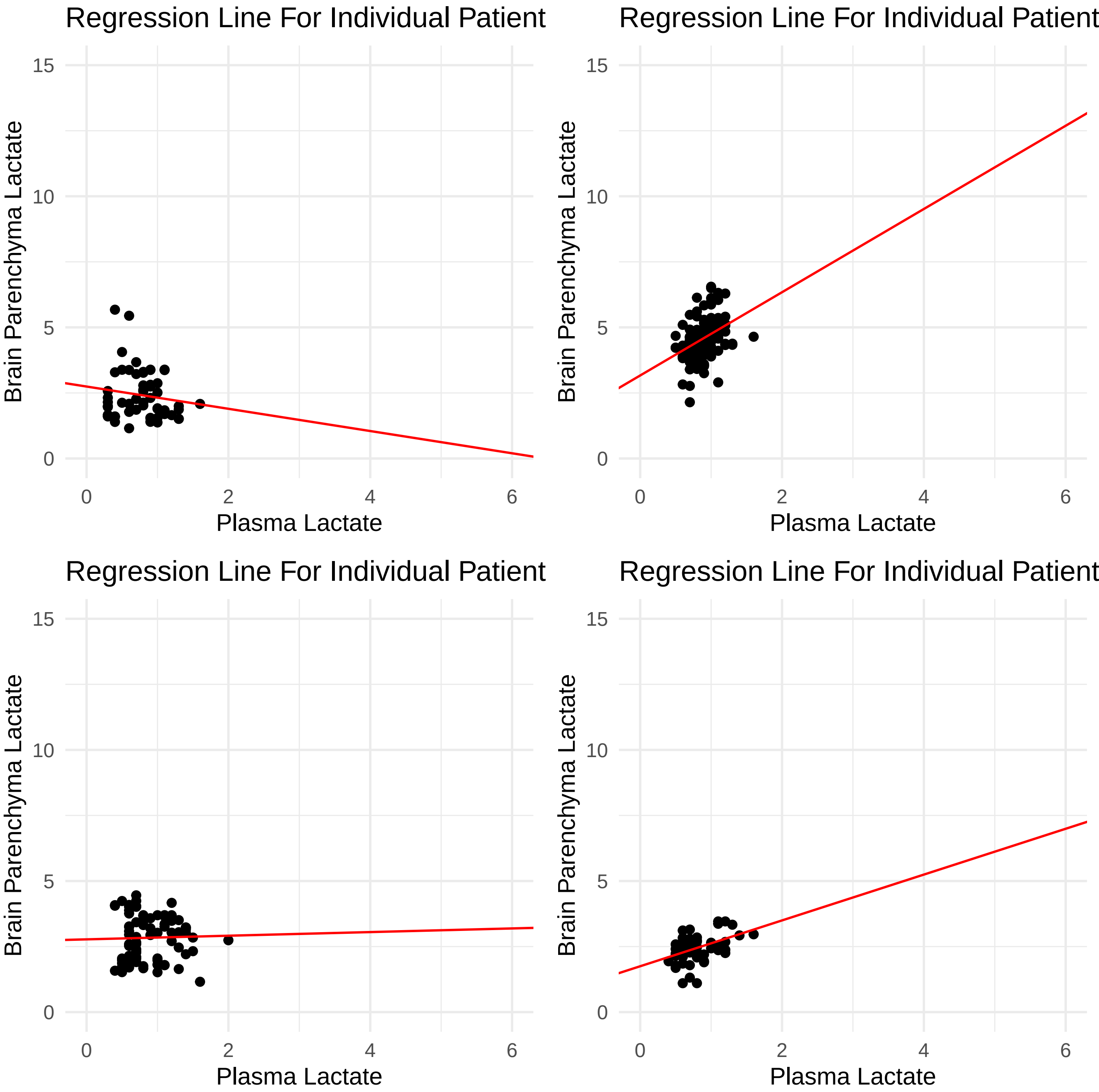

Regression Line For Individual Patient
Brain Parenchyma Lactate
15
10
5
0
0
2
4
6
Plasma Lactate
Regression Line For Individual Patient
Brain Parenchyma Lactate
15
10
5
0
0
2
4
6
Plasma Lactate
Regression Line For Individual Patient
Brain Parenchyma Lactate
15
10
5
0
0
2
4
6
Plasma Lactate
Regression Line For Individual Patient
Brain Parenchyma Lactate
15
10
5
0
0
2
4
6
Plasma Lactate

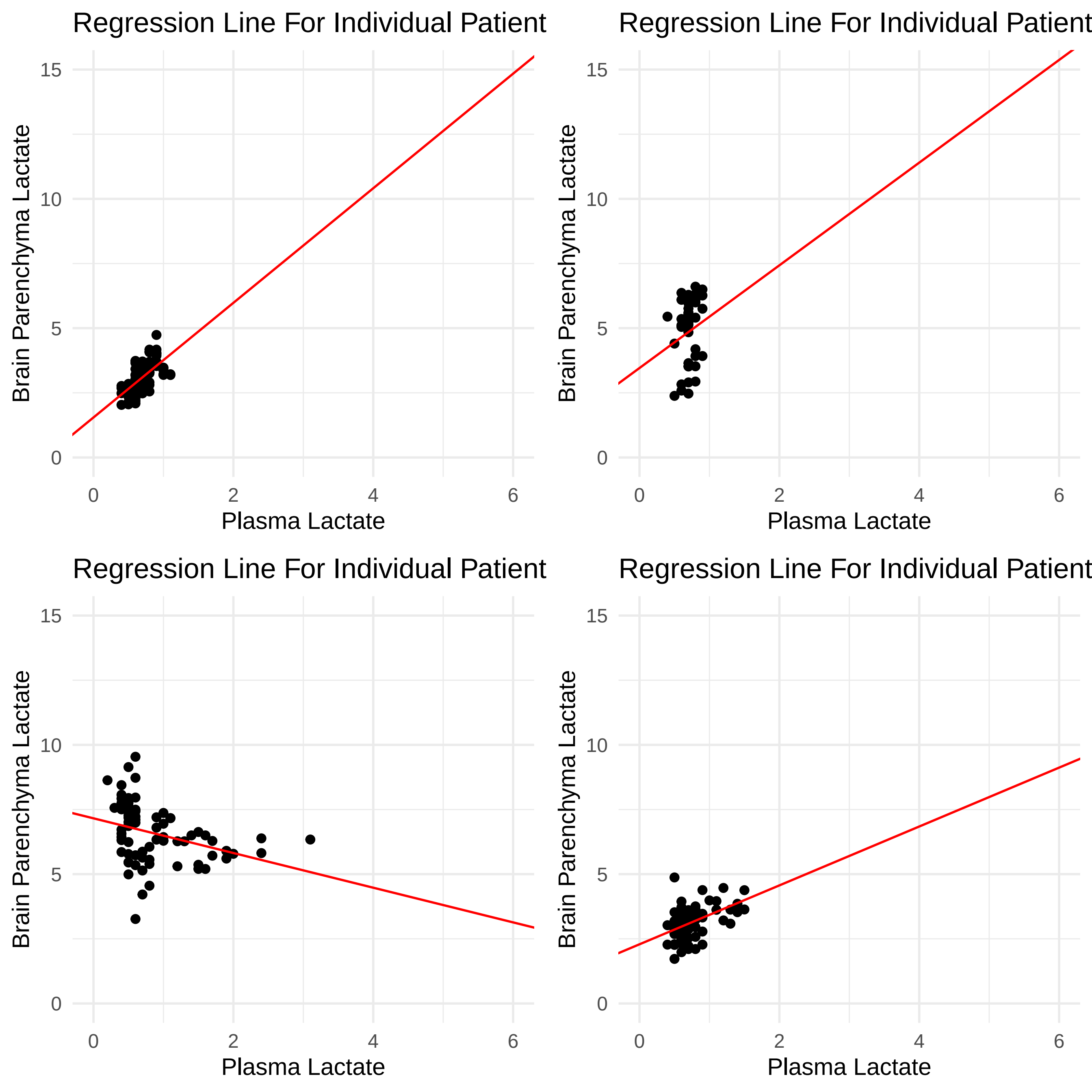

Regression Line For Individual Patient
Brain Parenchyma Lactate
Plasma Lactate
0
5
10
15
0
2
4
6
Regression Line For Individual Patient
Brain Parenchyma Lactate
Plasma Lactate
0
5
10
15
0
2
4
6
Regression Line For Individual Patient
Brain Parenchyma Lactate
Plasma Lactate
0
5
10
15
0
2
4
6
Regression Line For Individual Patient
Brain Parenchyma Lactate
Plasma Lactate
0
5
10
15
0
2
4
6

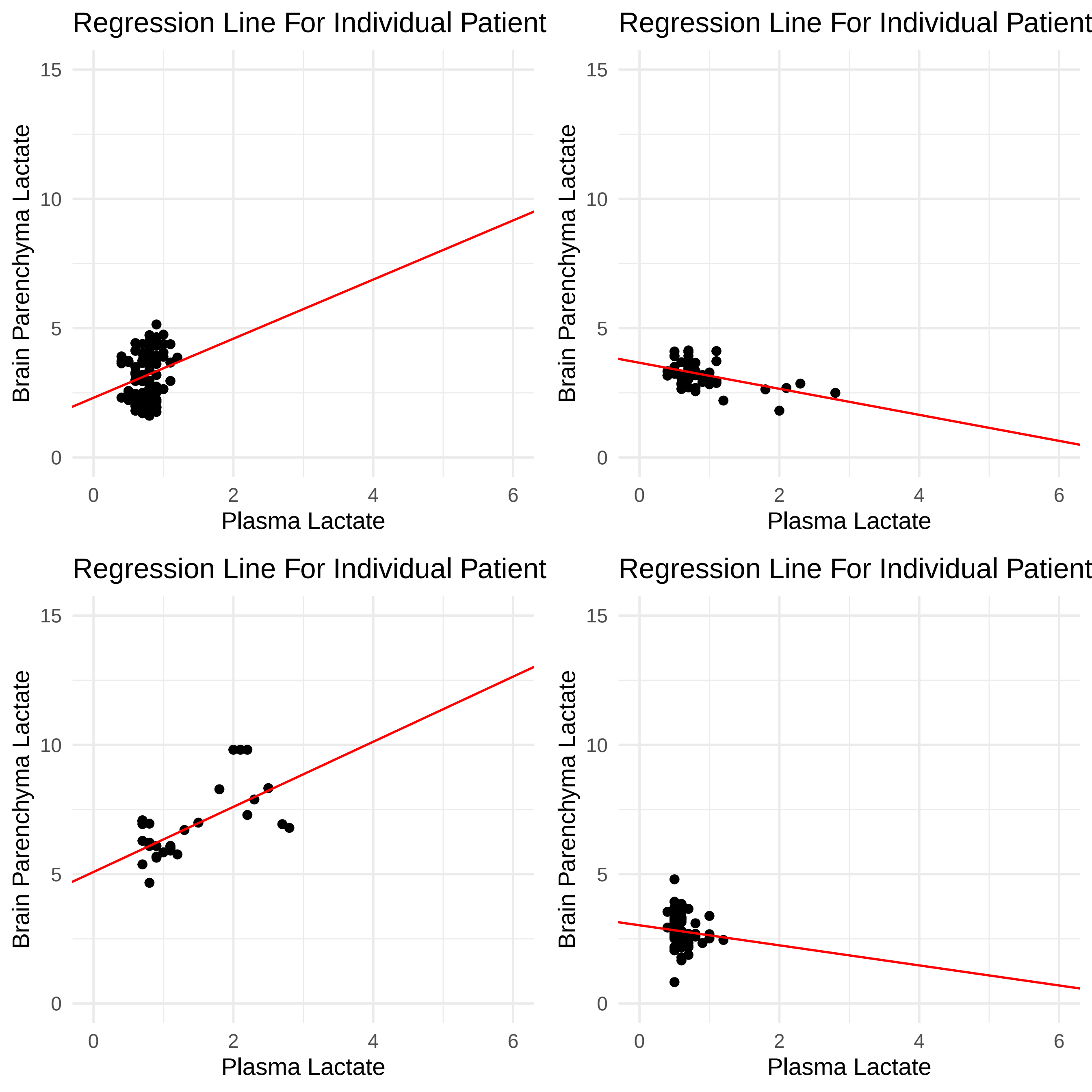

Regression Line For Individual Patient
Brain Parenchyma Lactate
Plasma Lactate
0
2
4
6
0
5
10
15
Regression Line For Individual Patient
Brain Parenchyma Lactate
Plasma Lactate
Regression Line For Individual Patient
Brain Parenchyma Lactate
Plasma Lactate
Regression Line For Individual Patient
Brain Parenchyma Lactate
Plasma Lactate

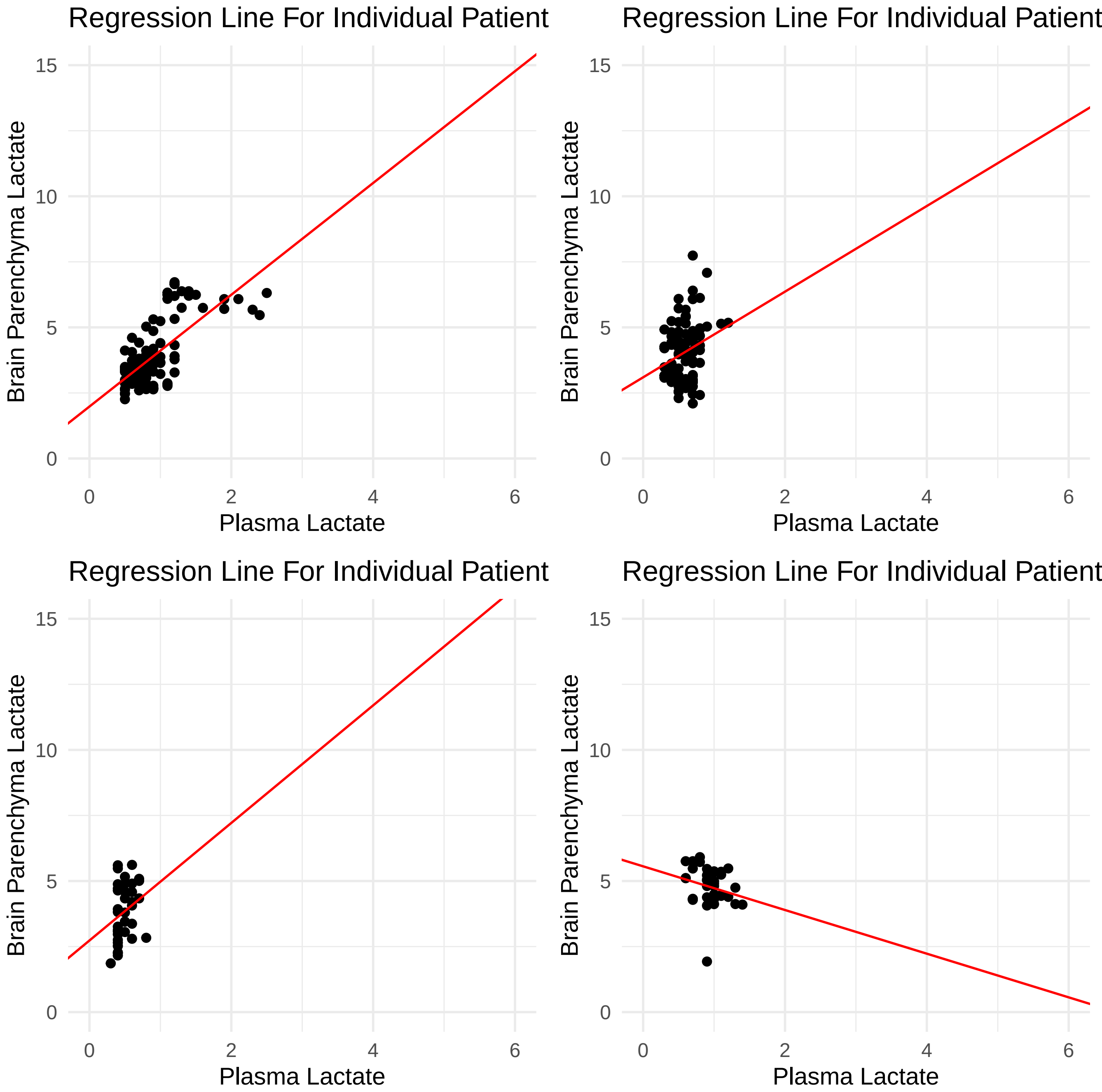
Regression Line For Individual Patient
Brain Parenchyma Lactate
Plasma Lactate
0
5
10
15
0
2
4
6
Regression Line For Individual Patient
Brain Parenchyma Lactate
Plasma Lactate
0
5
10
15
0
2
4
6
Regression Line For Individual Patient
Brain Parenchyma Lactate
Plasma Lactate
0
5
10
15
0
2
4
6
Regression Line For Individual Patient
Brain Parenchyma Lactate
Plasma Lactate
0
5
10
15
0
2
4
6

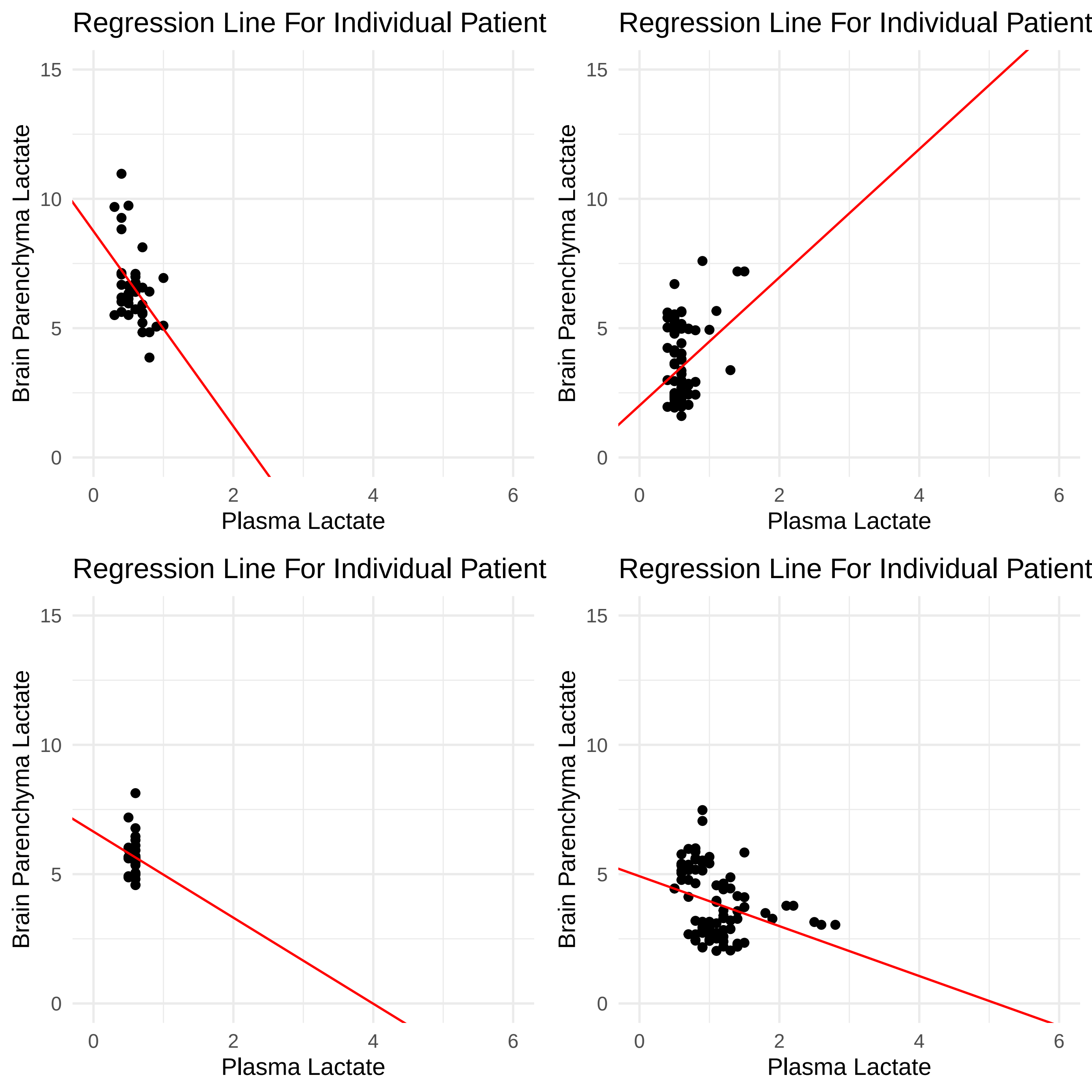
Regression Line For Individual Patient
Brain Parenchyma Lactate
Plasma Lactate
0
5
10
15
0
2
4
6
Regression Line For Individual Patient
Brain Parenchyma Lactate
Plasma Lactate
Regression Line For Individual Patient
Brain Parenchyma Lactate
Plasma Lactate
Regression Line For Individual Patient
Brain Parenchyma Lactate
Plasma Lactate

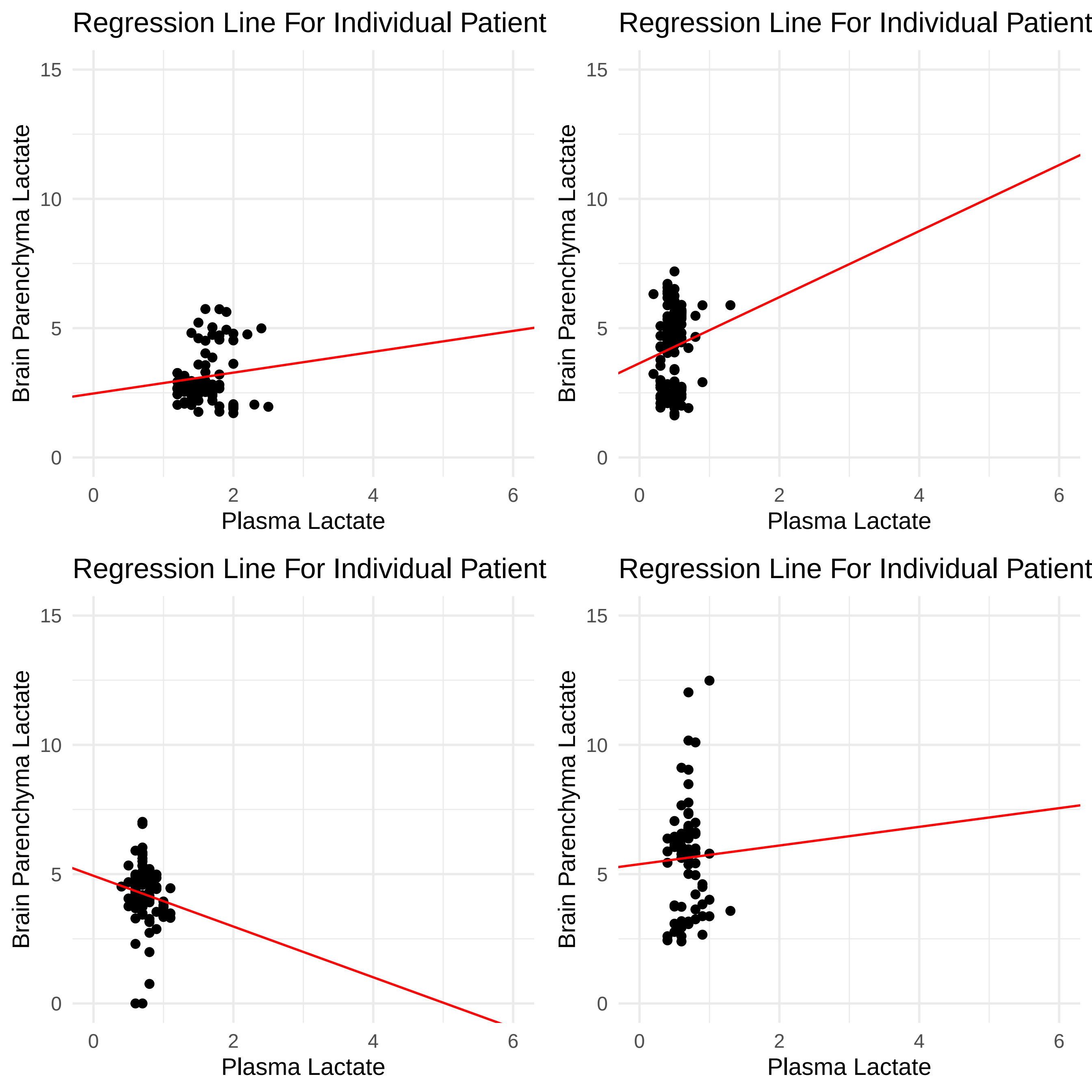
Regression Line For Individual Patient
Brain Parenchyma Lactate
Plasma Lactate
0
5
10
15
0
2
4
6
Regression Line For Individual Patient
Brain Parenchyma Lactate
Plasma Lactate
Regression Line For Individual Patient
Brain Parenchyma Lactate
Plasma Lactate
Regression Line For Individual Patient
Brain Parenchyma Lactate
Plasma Lactate

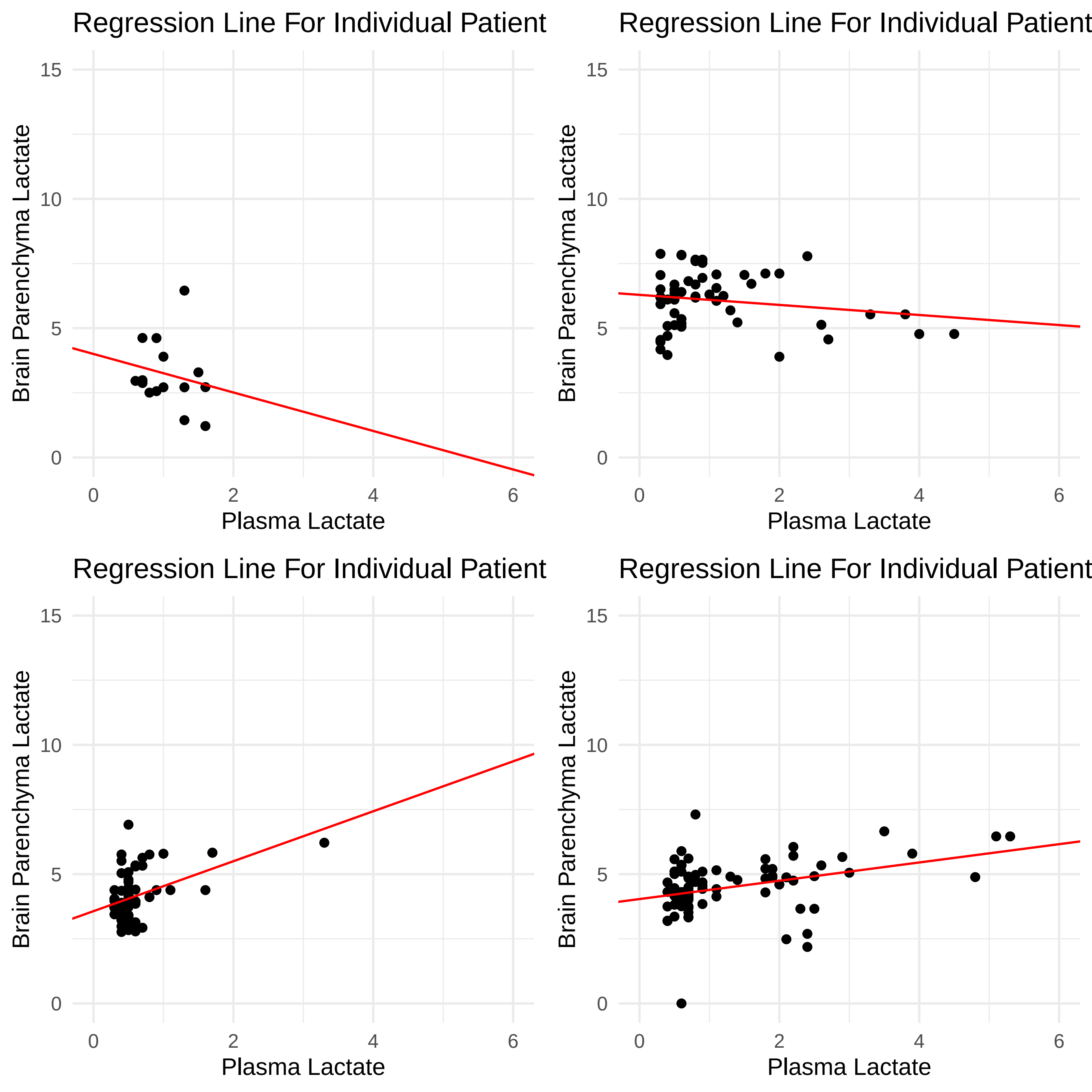

Regression Line For Individual Patient
Brain Parenchyma Lactate
Plasma Lactate
0
5
10
15
0
2
4
6
Regression Line For Individual Patient
Brain Parenchyma Lactate
Plasma Lactate
Regression Line For Individual Patient
Brain Parenchyma Lactate
Plasma Lactate
Regression Line For Individual Patient
Brain Parenchyma Lactate
Plasma Lactate

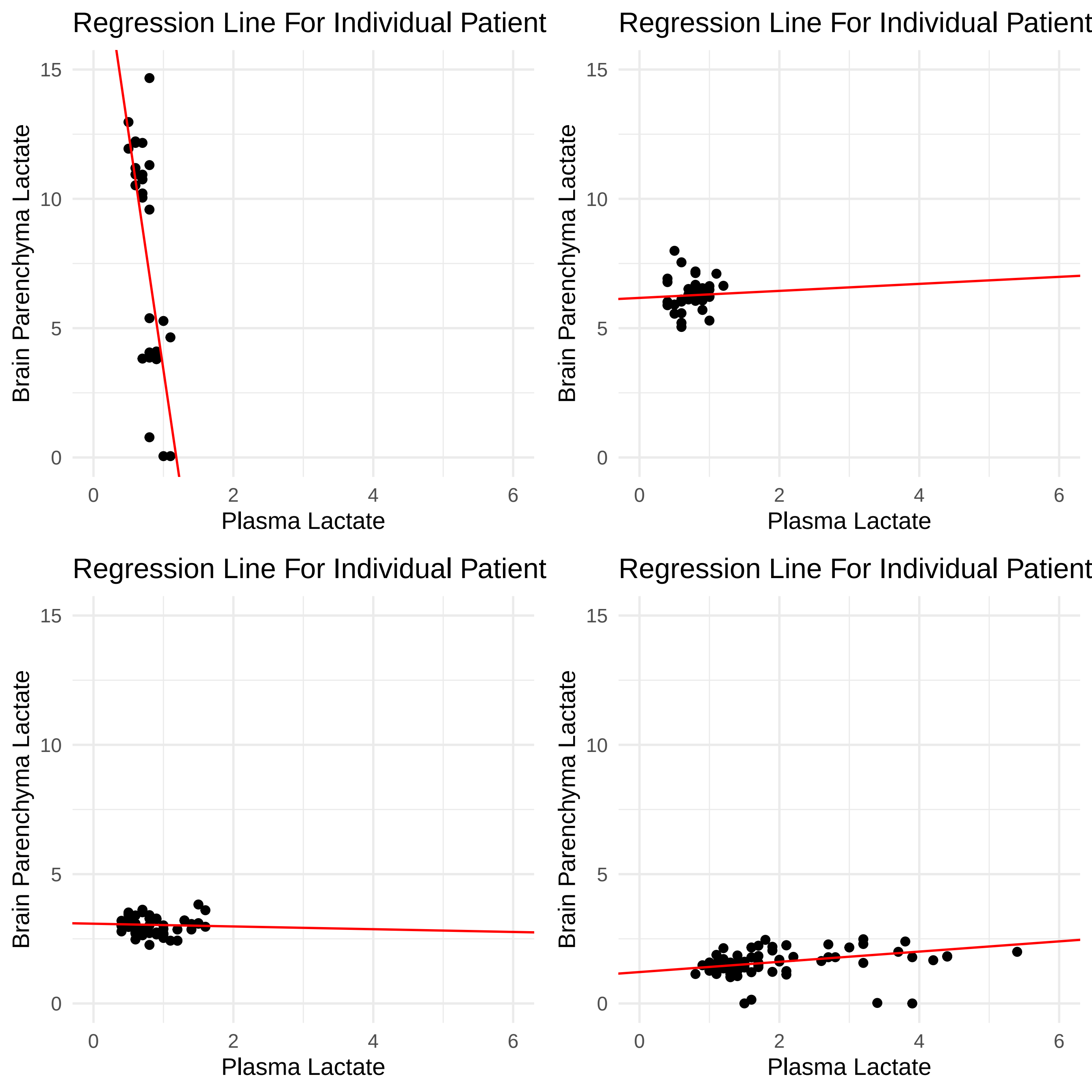

Regression Line For Individual Patient
Brain Parenchyma Lactate
Plasma Lactate
0
5
10
15
0
2
4
6
Regression Line For Individual Patient
Brain Parenchyma Lactate
Plasma Lactate
0
5
10
15
0
2
4
6
Regression Line For Individual Patient
Brain Parenchyma Lactate
Plasma Lactate
0
5
10
15
0
2
4
6
Regression Line For Individual Patient
Brain Parenchyma Lactate
Plasma Lactate
0
5
10
15
0
2
4
6

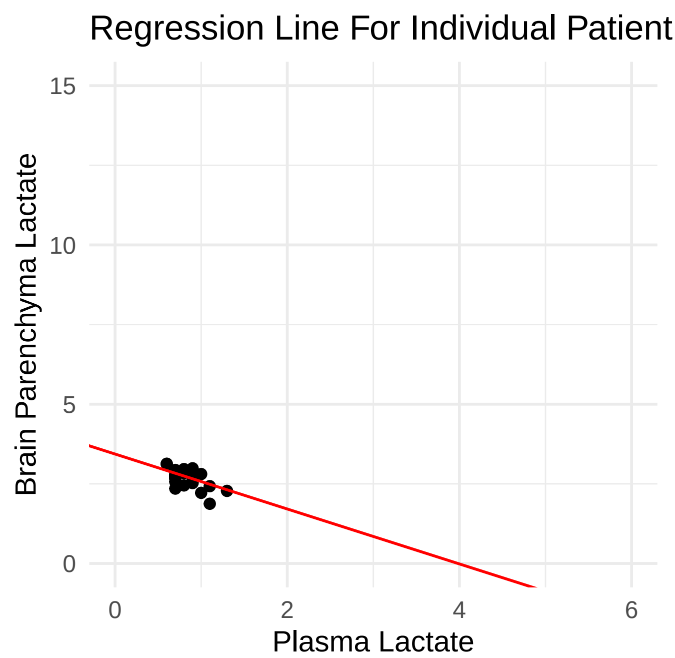
Regression Line For Individual Patient
Brain Parenchyma Lactate
Plasma Lactate
0
5
10
15
0
2
4
6

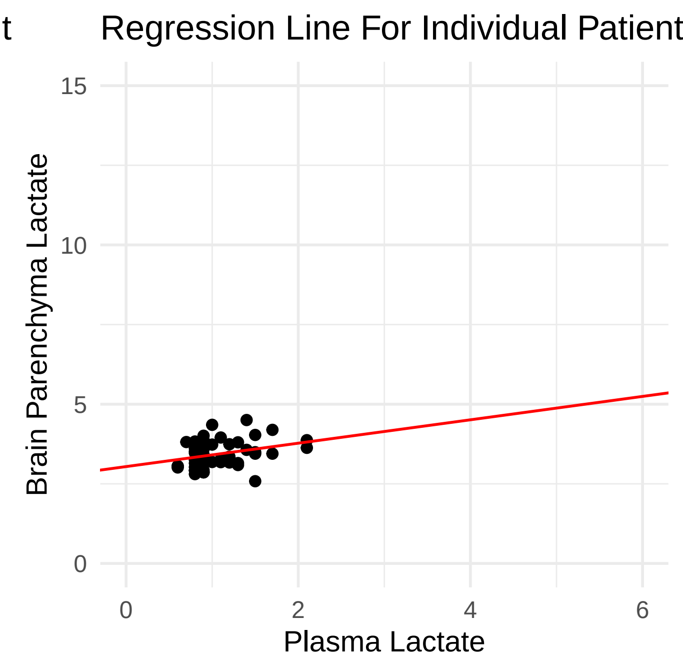
Regression Line For Individual Patient
Brain Parenchyma Lactate
Plasma Lactate
0
5
10
15
0
2
4
6

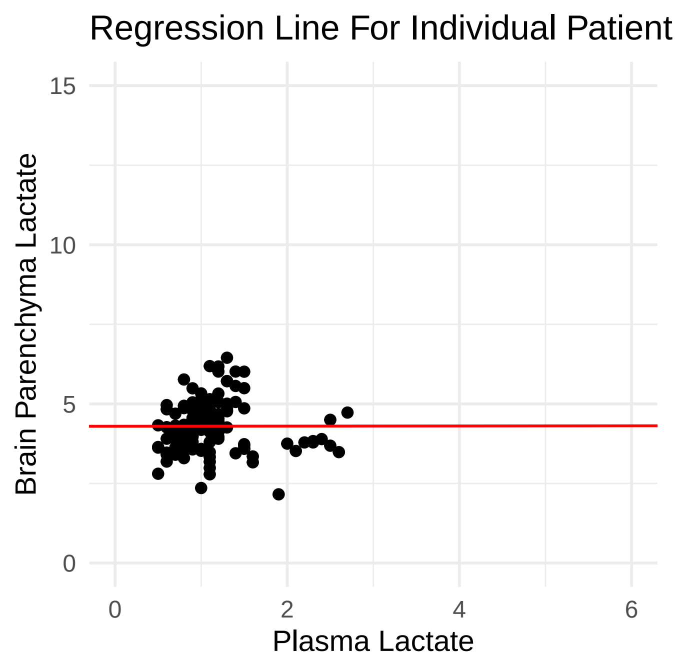
Regression Line For Individual Patient
Brain Parenchyma Lactate
Plasma Lactate
0
5
10
15
0
2
4
6

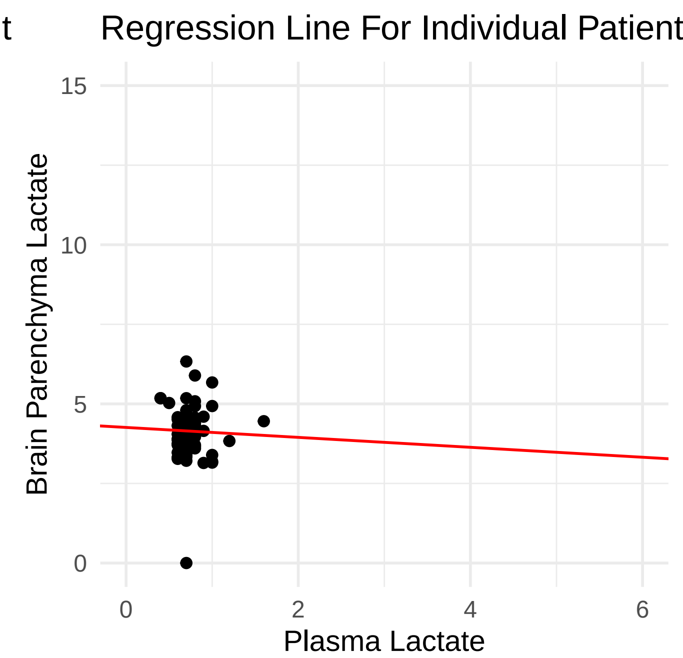
Regression Line For Individual Patient
Brain Parenchyma Lactate
Plasma Lactate
0
5
10
15
0
2
4
6

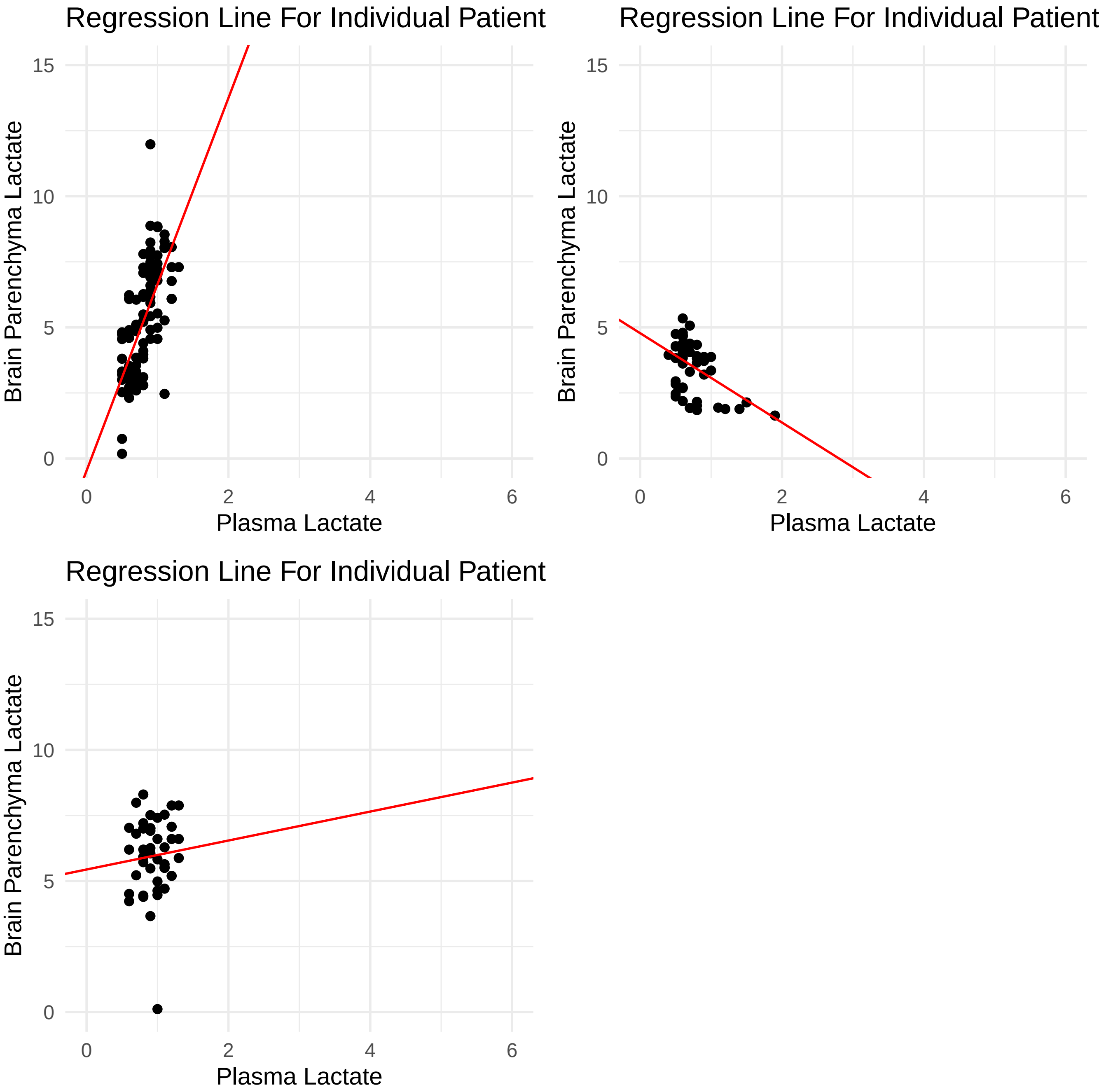
Regression Line For Individual Patient
Regression Line For Individual Patient
Regression Line For Individual Patient
Brain Parenchyma Lactate
Plasma Lactate
0
5
10
15
0
2
4
6

## 5.5 Plasma Glucose vs. CSF Glucose

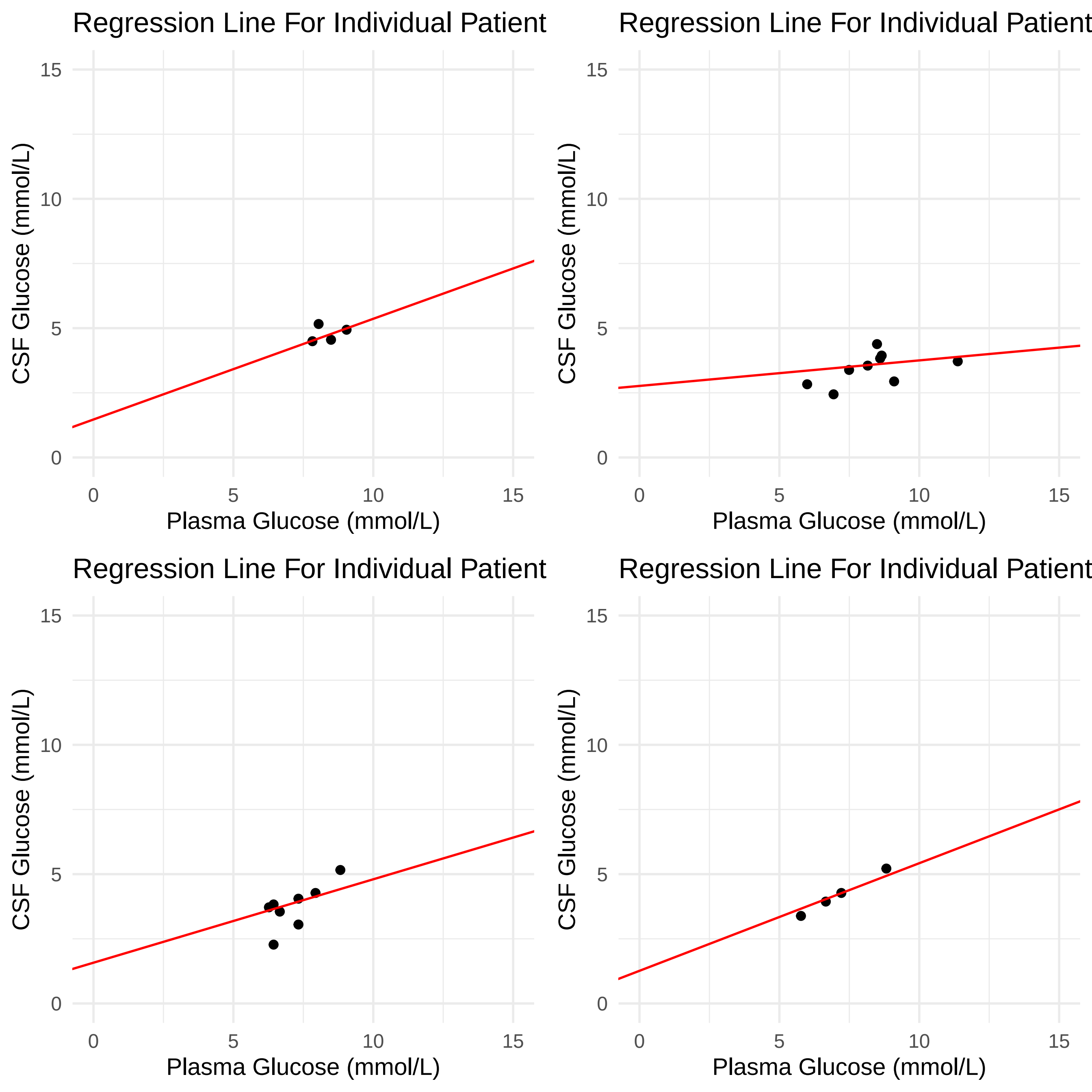

Regression Line For Individual Patient
CSF Glucose (mmol/L)
Plasma Glucose (mmol/L)
0
5
10
15
Regression Line For Individual Patient
CSF Glucose (mmol/L)
Plasma Glucose (mmol/L)
Regression Line For Individual Patient
CSF Glucose (mmol/L)
Plasma Glucose (mmol/L)
Regression Line For Individual Patient
CSF Glucose (mmol/L)
Plasma Glucose (mmol/L)

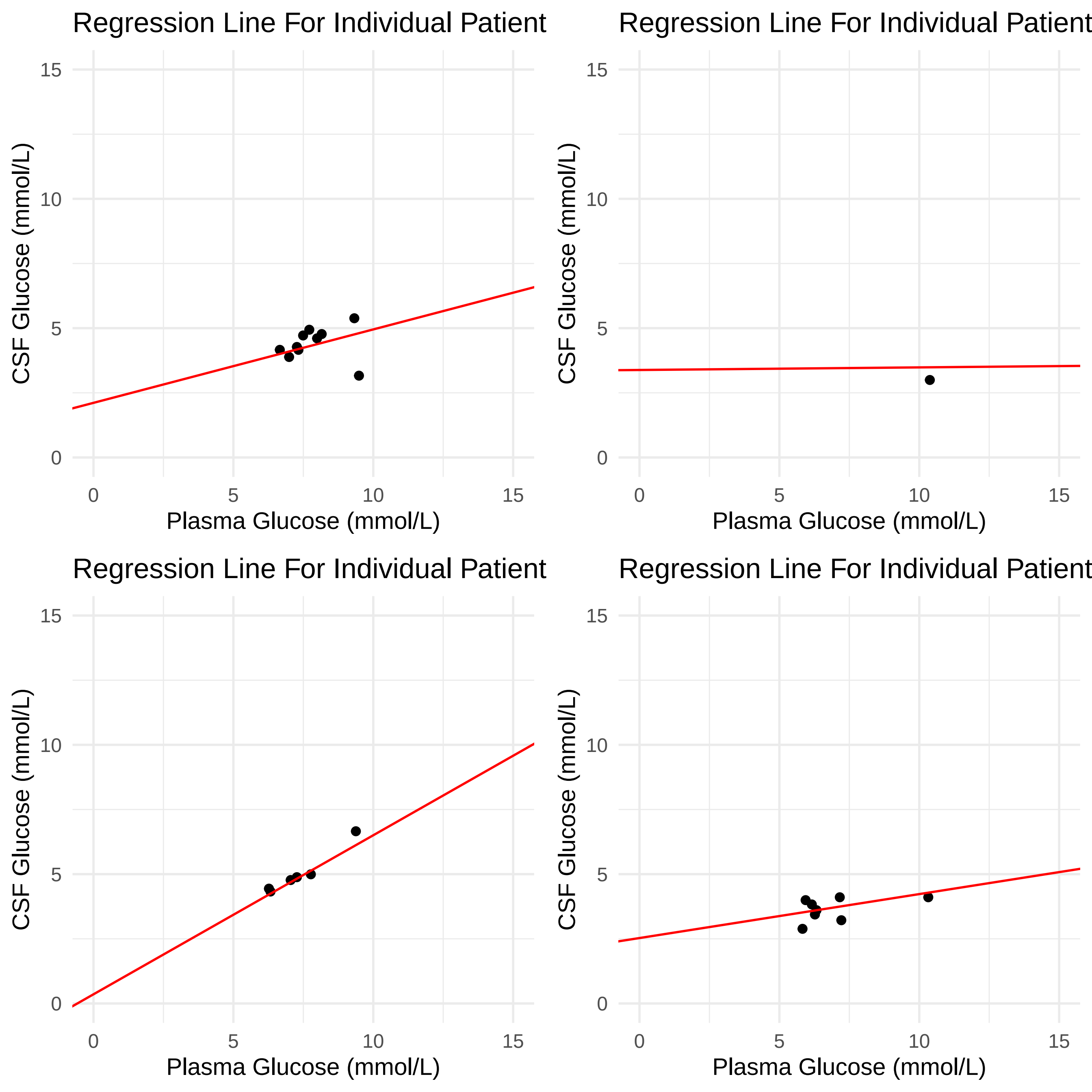
Regression Line For Individual Patient
CSF Glucose (mmol/L)
Plasma Glucose (mmol/L)
0
5
10
15
Regression Line For Individual Patient
CSF Glucose (mmol/L)
Plasma Glucose (mmol/L)
Regression Line For Individual Patient
CSF Glucose (mmol/L)
Plasma Glucose (mmol/L)
Regression Line For Individual Patient
CSF Glucose (mmol/L)
Plasma Glucose (mmol/L)

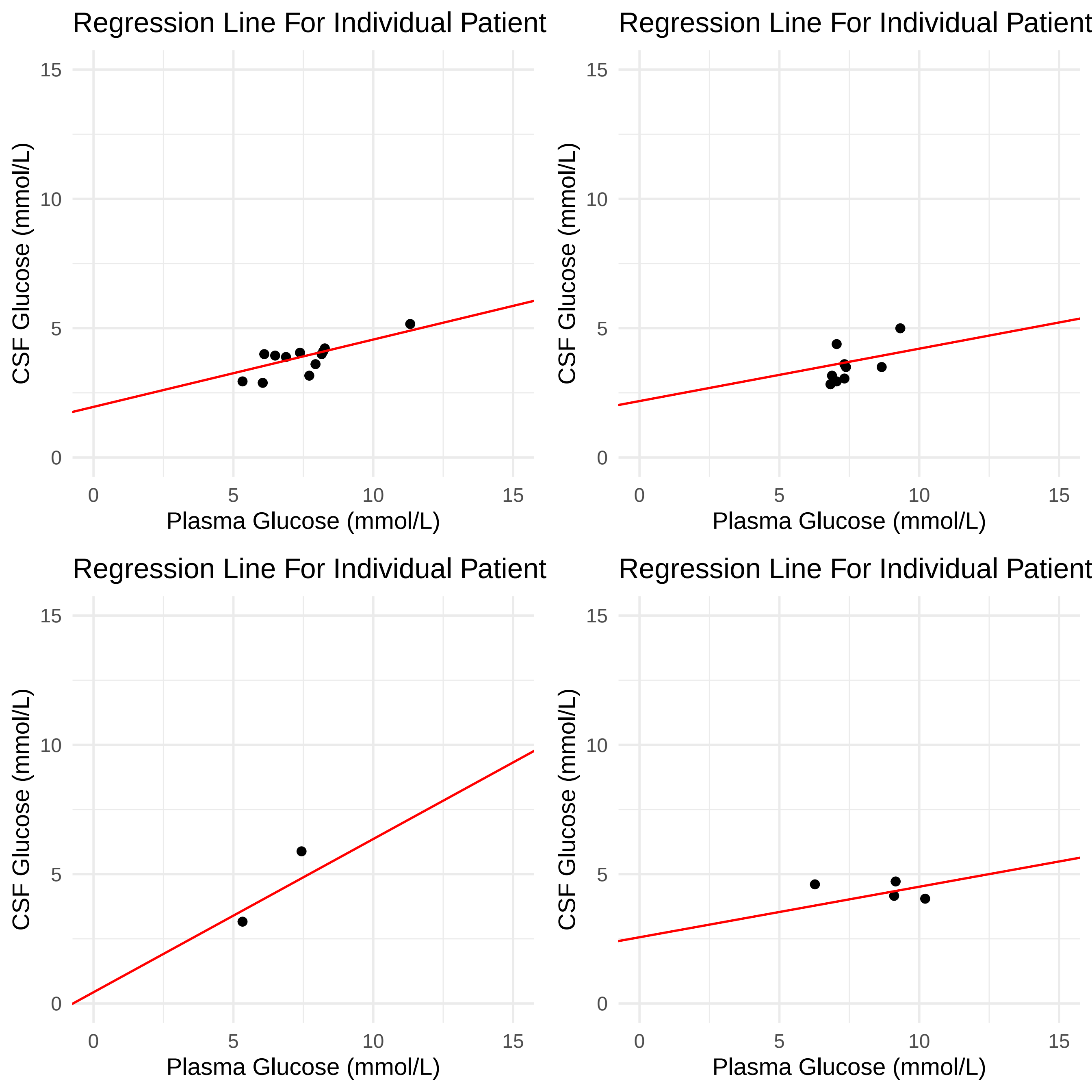

Regression Line For Individual Patient
CSF Glucose (mmol/L)
Plasma Glucose (mmol/L)
0
5
10
15
0
5
10
15
Regression Line For Individual Patient
CSF Glucose (mmol/L)
Plasma Glucose (mmol/L)
Regression Line For Individual Patient
CSF Glucose (mmol/L)
Plasma Glucose (mmol/L)
Regression Line For Individual Patient
CSF Glucose (mmol/L)
Plasma Glucose (mmol/L)

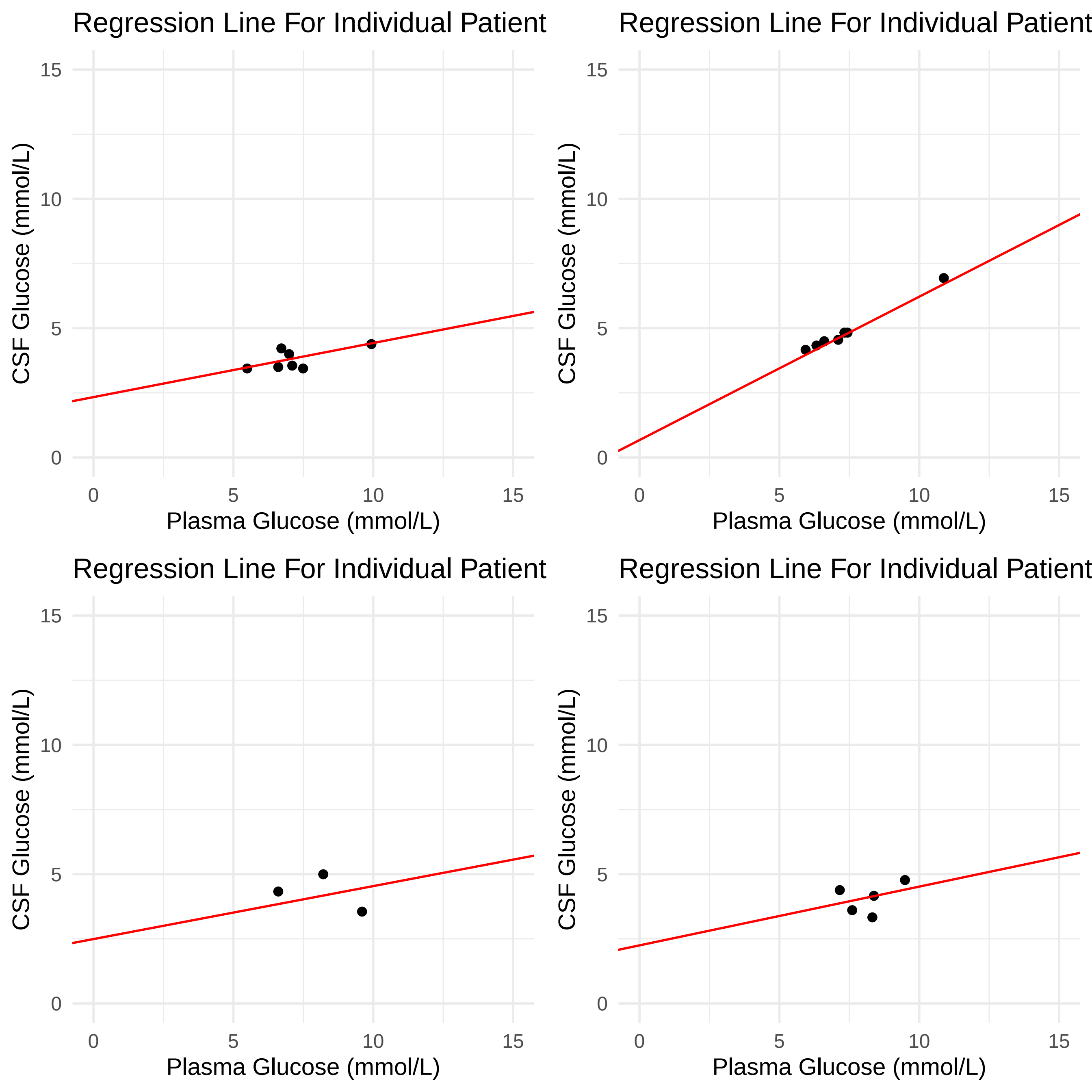

Regression Line For Individual Patient
CSF Glucose (mmol/L)
Plasma Glucose (mmol/L)
0
5
10
15
0
5
10
15
Regression Line For Individual Patient
CSF Glucose (mmol/L)
Plasma Glucose (mmol/L)
Regression Line For Individual Patient
CSF Glucose (mmol/L)
Plasma Glucose (mmol/L)
Regression Line For Individual Patient
CSF Glucose (mmol/L)
Plasma Glucose (mmol/L)

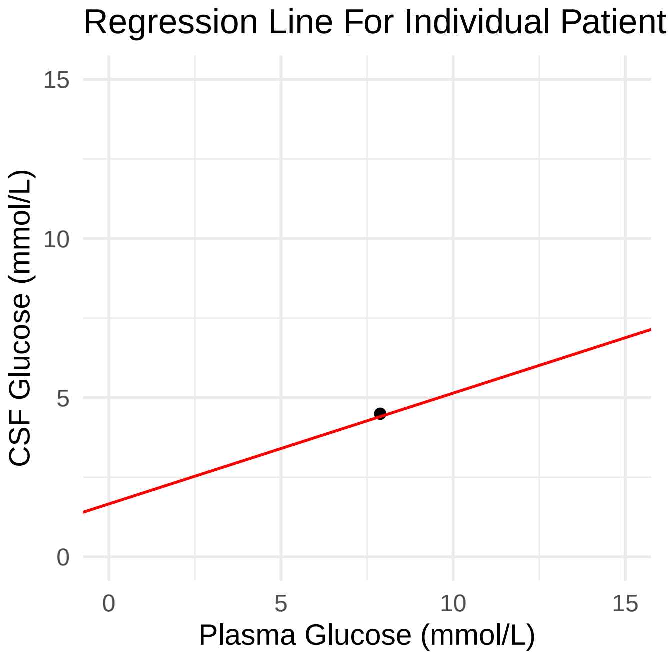
Regression Line For Individual Patient
15
10
5
0
CSF Glucose (mmol/L)
0
5
10
15
Plasma Glucose (mmol/L)
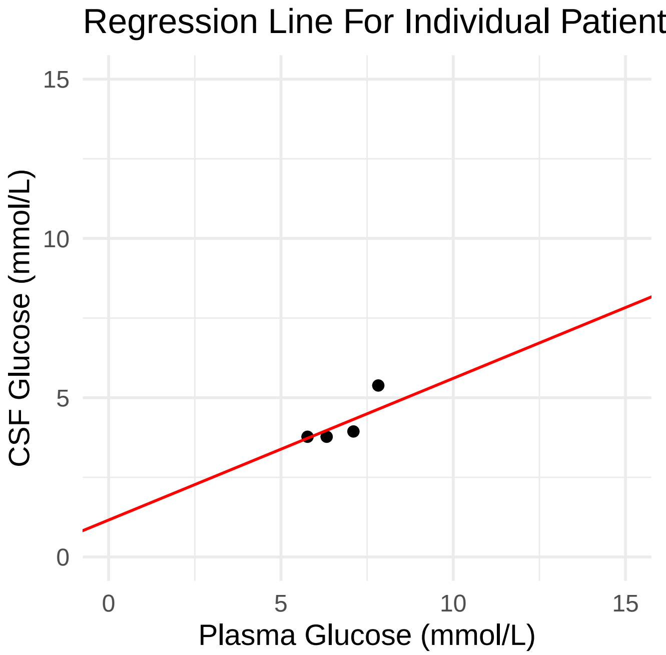
Regression Line For Individual Patient
15
10
5
0
CSF Glucose (mmol/L)
0
5
10
15
Plasma Glucose (mmol/L)
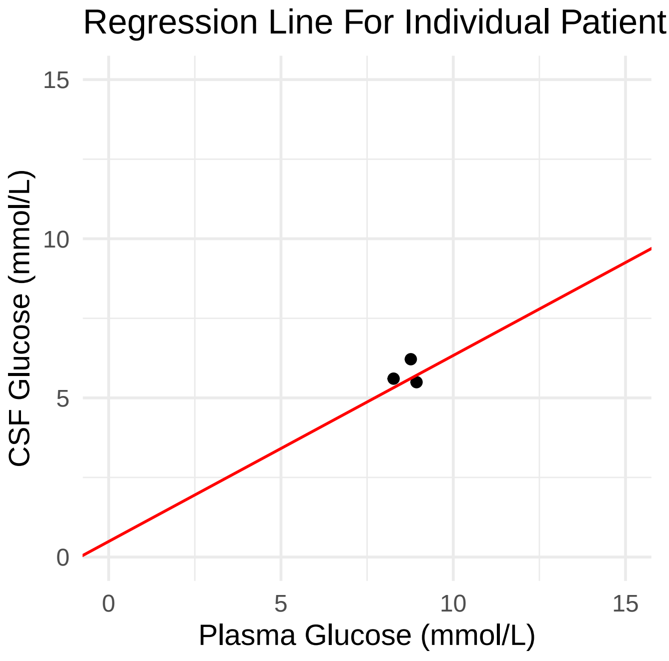
Regression Line For Individual Patient
15
10
5
0
CSF Glucose (mmol/L)
0
5
10
15
Plasma Glucose (mmol/L)
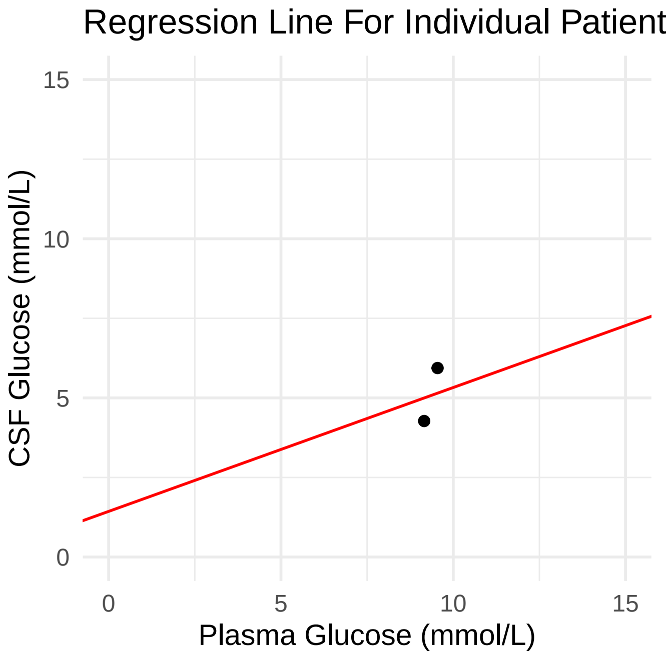
Regression Line For Individual Patient
15
10
5
0
CSF Glucose (mmol/L)
0
5
10
15
Plasma Glucose (mmol/L)

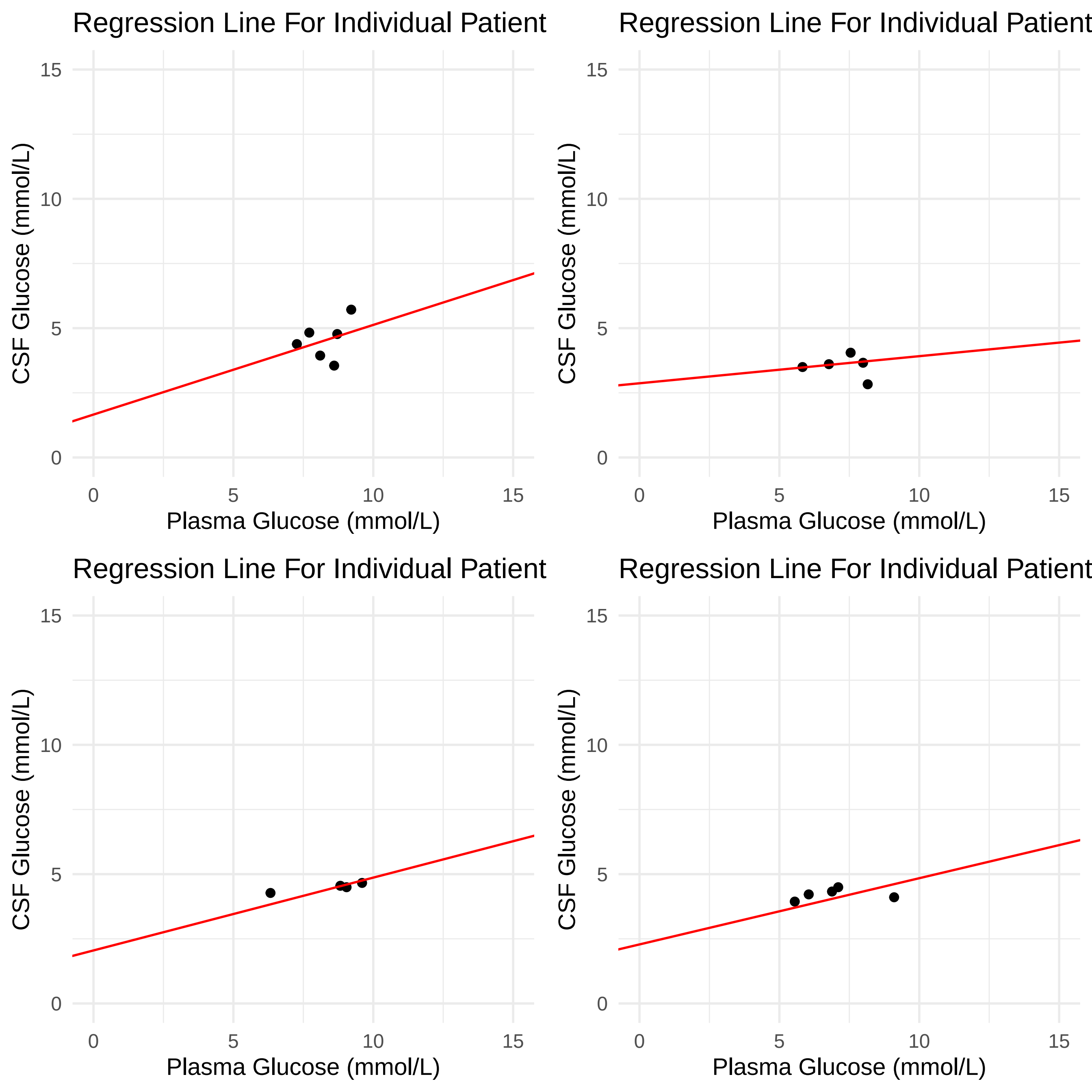

Regression Line For Individual Patient
CSF Glucose (mmol/L)
Plasma Glucose (mmol/L)
0
5
10
15
Regression Line For Individual Patient
CSF Glucose (mmol/L)
Plasma Glucose (mmol/L)
0
5
10
15
Regression Line For Individual Patient
CSF Glucose (mmol/L)
Plasma Glucose (mmol/L)
0
5
10
15
Regression Line For Individual Patient
CSF Glucose (mmol/L)
Plasma Glucose (mmol/L)
0
5
10
15

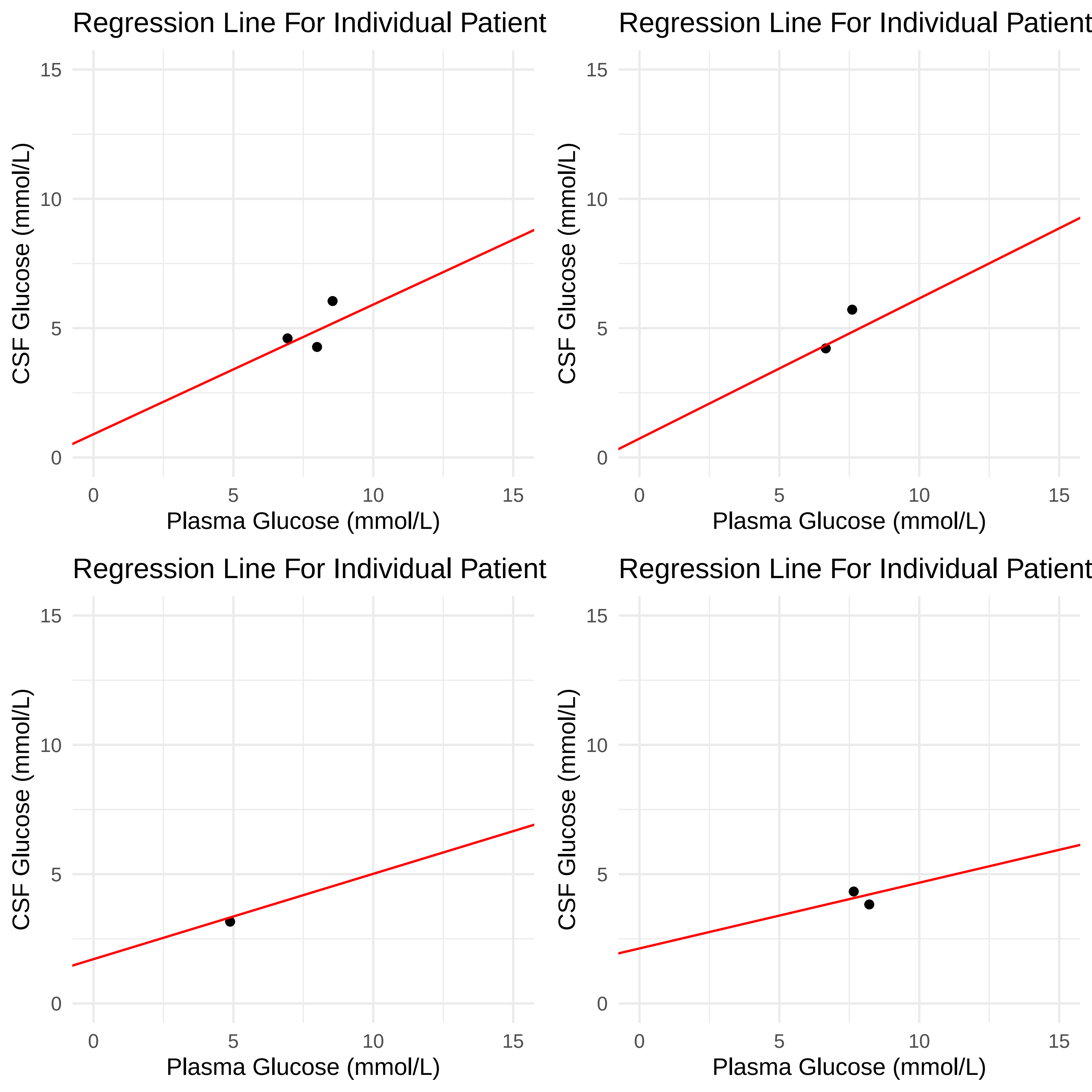
Regression Line For Individual Patient
Regression Line For Individual Patient
Regression Line For Individual Patient
Regression Line For Individual Patient
CSF Glucose (mmol/L)
Plasma Glucose (mmol/L)
0
5
10
15

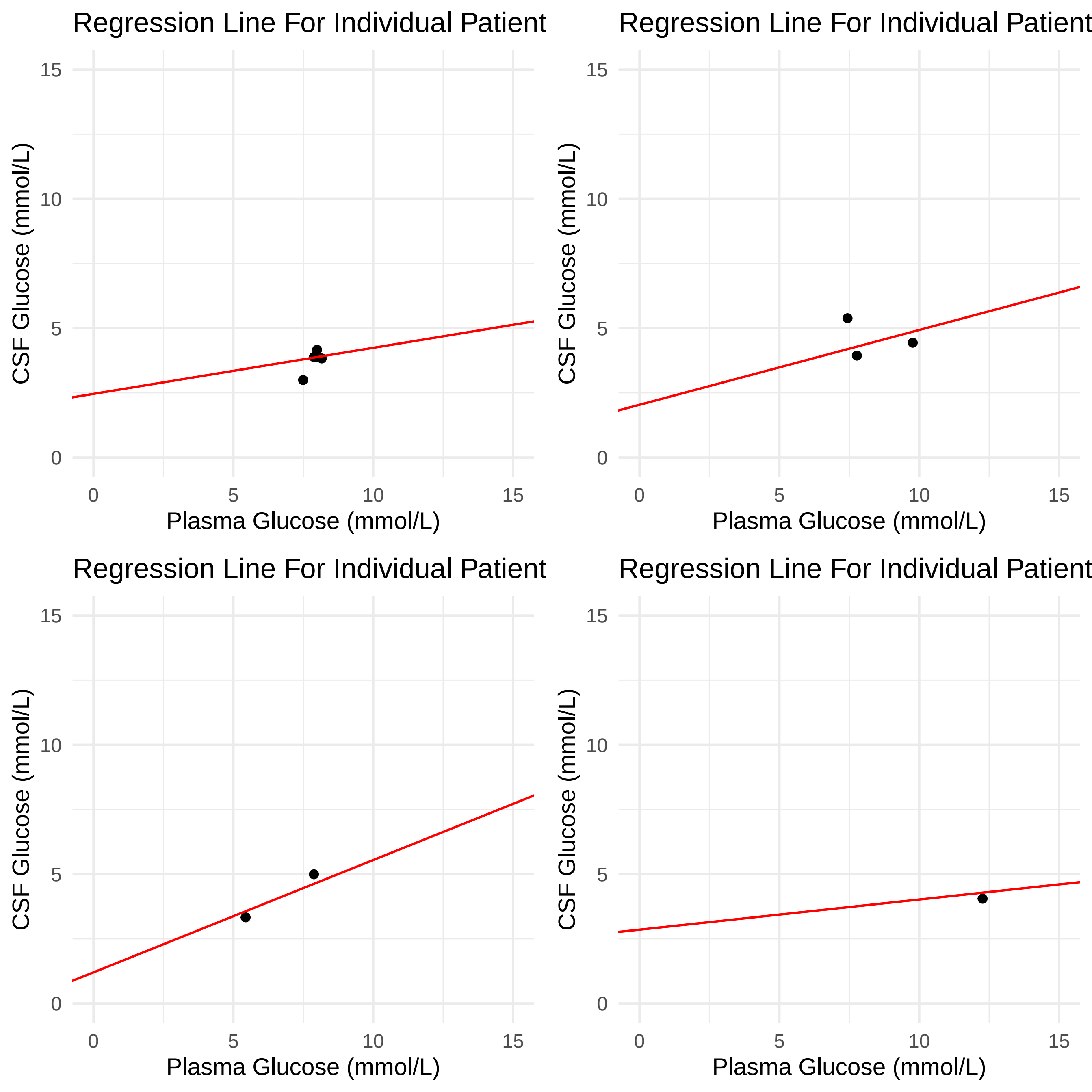
Regression Line For Individual Patient
CSF Glucose (mmol/L)
Plasma Glucose (mmol/L)
0
5
10
15
Regression Line For Individual Patient
CSF Glucose (mmol/L)
Plasma Glucose (mmol/L)
Regression Line For Individual Patient
CSF Glucose (mmol/L)
Plasma Glucose (mmol/L)
Regression Line For Individual Patient
CSF Glucose (mmol/L)
Plasma Glucose (mmol/L)

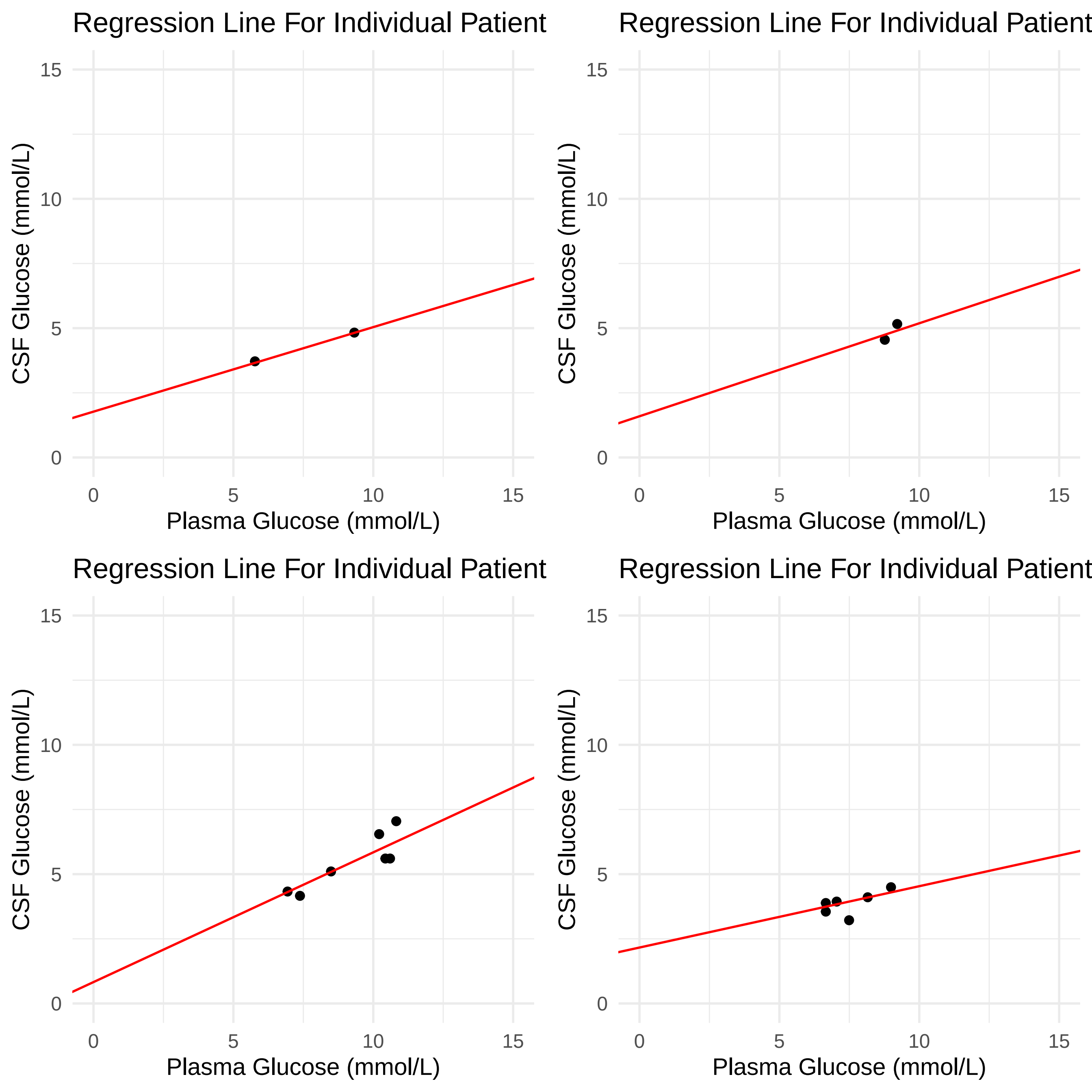
Regression Line For Individual Patient
CSF Glucose (mmol/L)
Plasma Glucose (mmol/L)
0
5
10
15
Regression Line For Individual Patient
CSF Glucose (mmol/L)
Plasma Glucose (mmol/L)
Regression Line For Individual Patient
CSF Glucose (mmol/L)
Plasma Glucose (mmol/L)
Regression Line For Individual Patient
CSF Glucose (mmol/L)
Plasma Glucose (mmol/L)

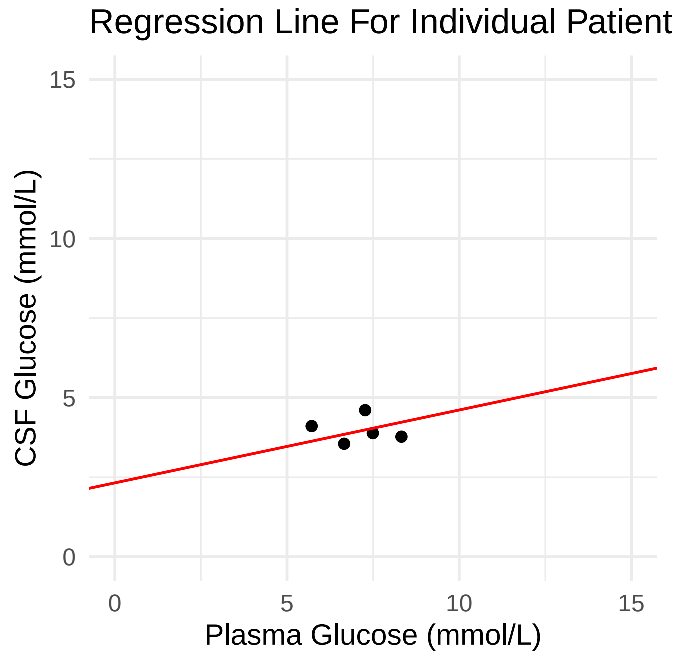
Regression Line For Individual Patient
CSF Glucose (mmol/L)
Plasma Glucose (mmol/L)
0
5
10
15
0
5
10
15

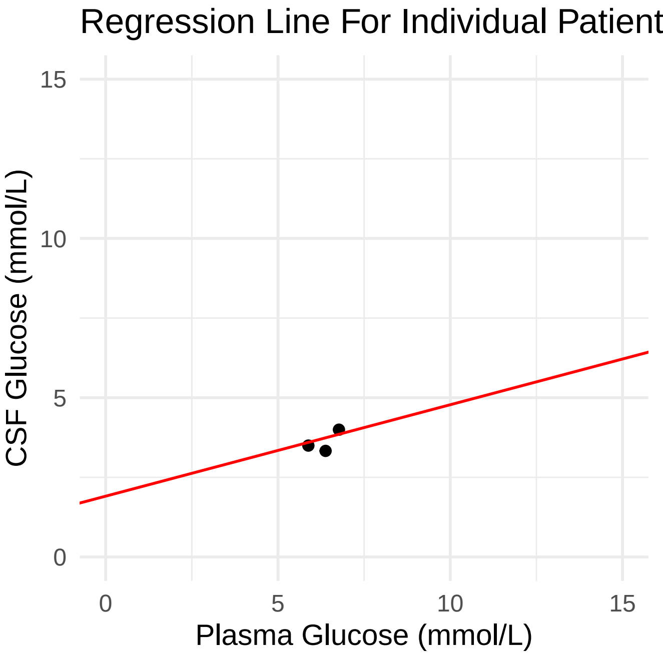
Regression Line For Individual Patient
CSF Glucose (mmol/L)
Plasma Glucose (mmol/L)
0
5
10
15
0
5
10
15

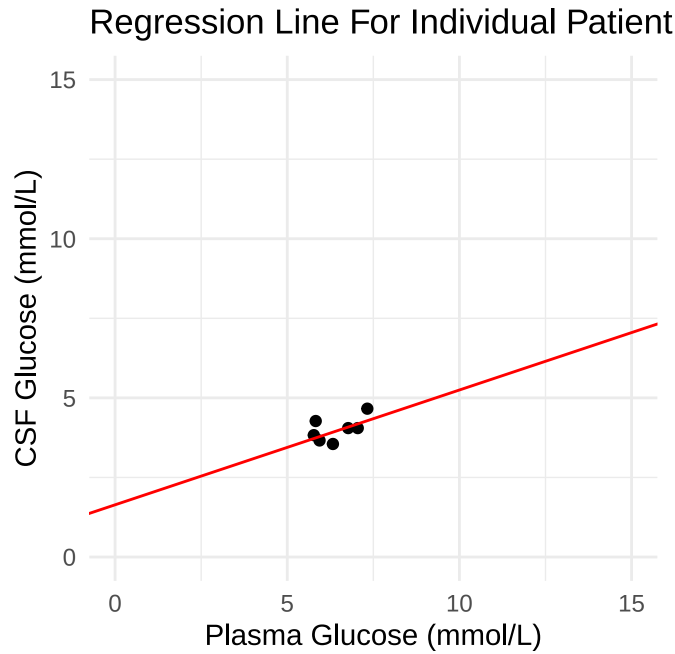
Regression Line For Individual Patient
CSF Glucose (mmol/L)
Plasma Glucose (mmol/L)
0
5
10
15
0
5
10
15

## 5.6 Plasma Lactate vs. CSF Lactate

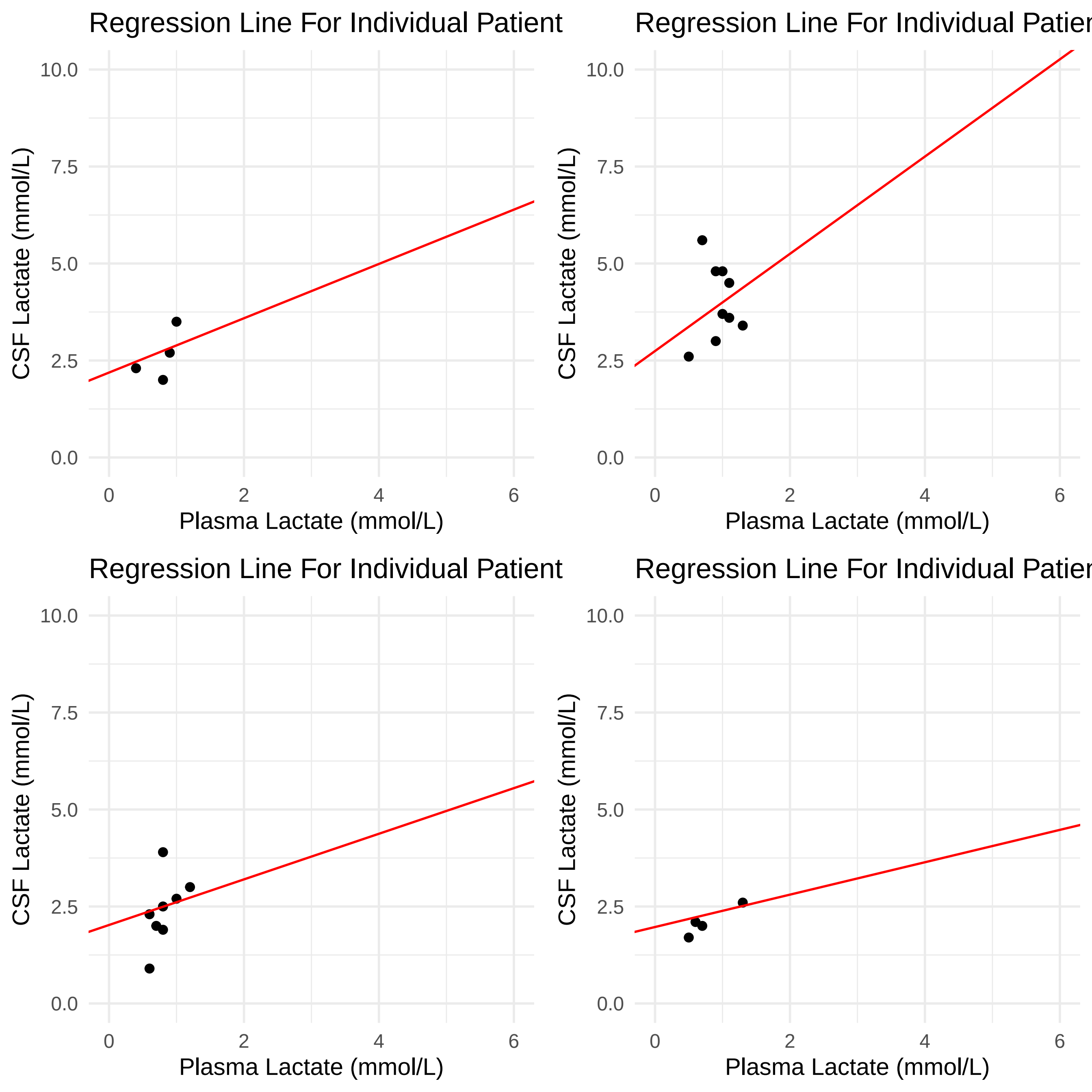
Regression Line For Individual Patient
CSF Lactate (mmol/L)
Plasma Lactate (mmol/L)
0.0
2.5
5.0
7.5
10.0
0
2
4
6
Regression Line For Individual Patien
CSF Lactate (mmol/L)
Plasma Lactate (mmol/L)
Regression Line For Individual Patient
CSF Lactate (mmol/L)
Plasma Lactate (mmol/L)
Regression Line For Individual Patien
CSF Lactate (mmol/L)
Plasma Lactate (mmol/L)

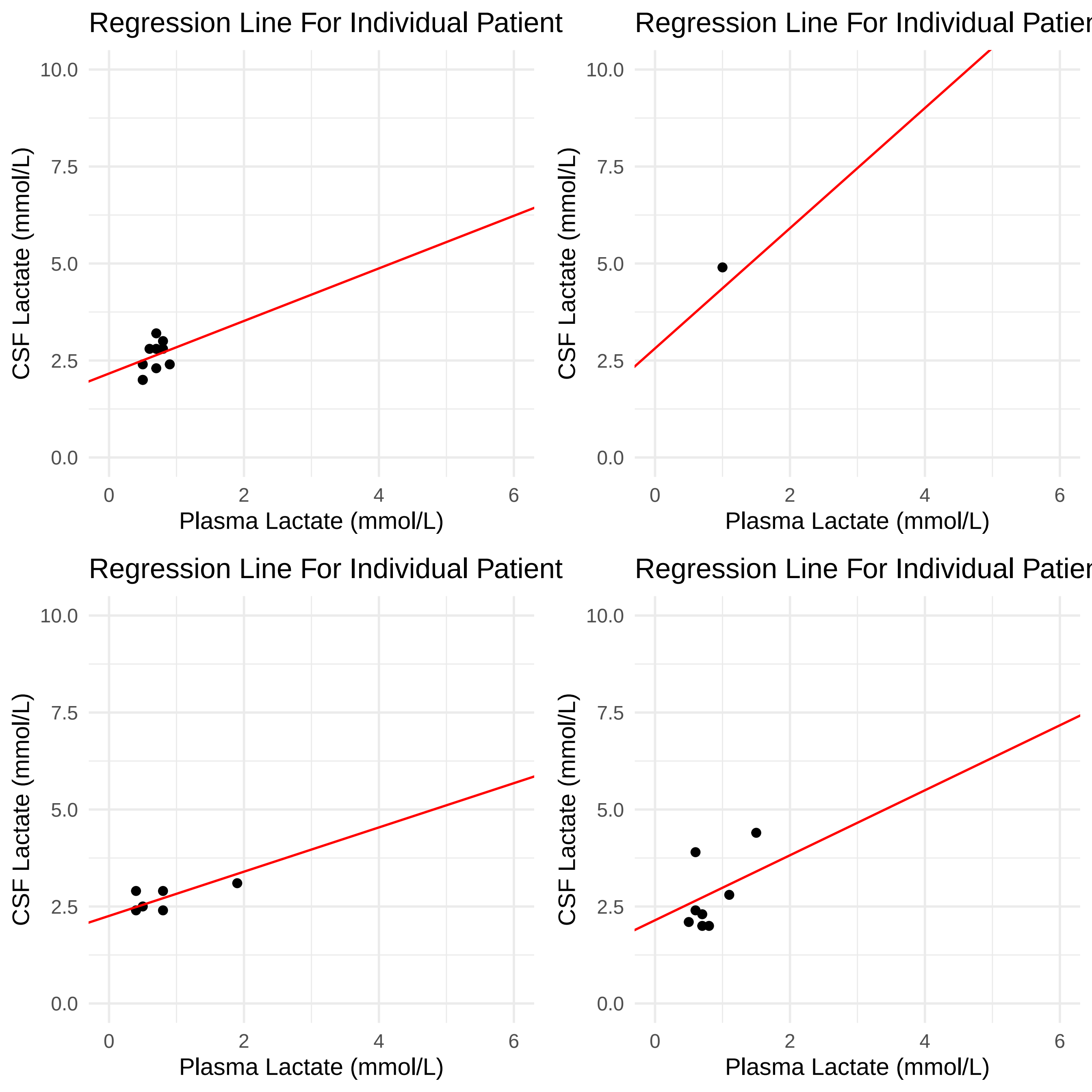
Regression Line For Individual Patient
Regression Line For Individual Patien
Regression Line For Individual Patient
Regression Line For Individual Patien
CSF Lactate (mmol/L)
Plasma Lactate (mmol/L)
0.0
2.5
5.0
7.5
10.0
0
2
4
6

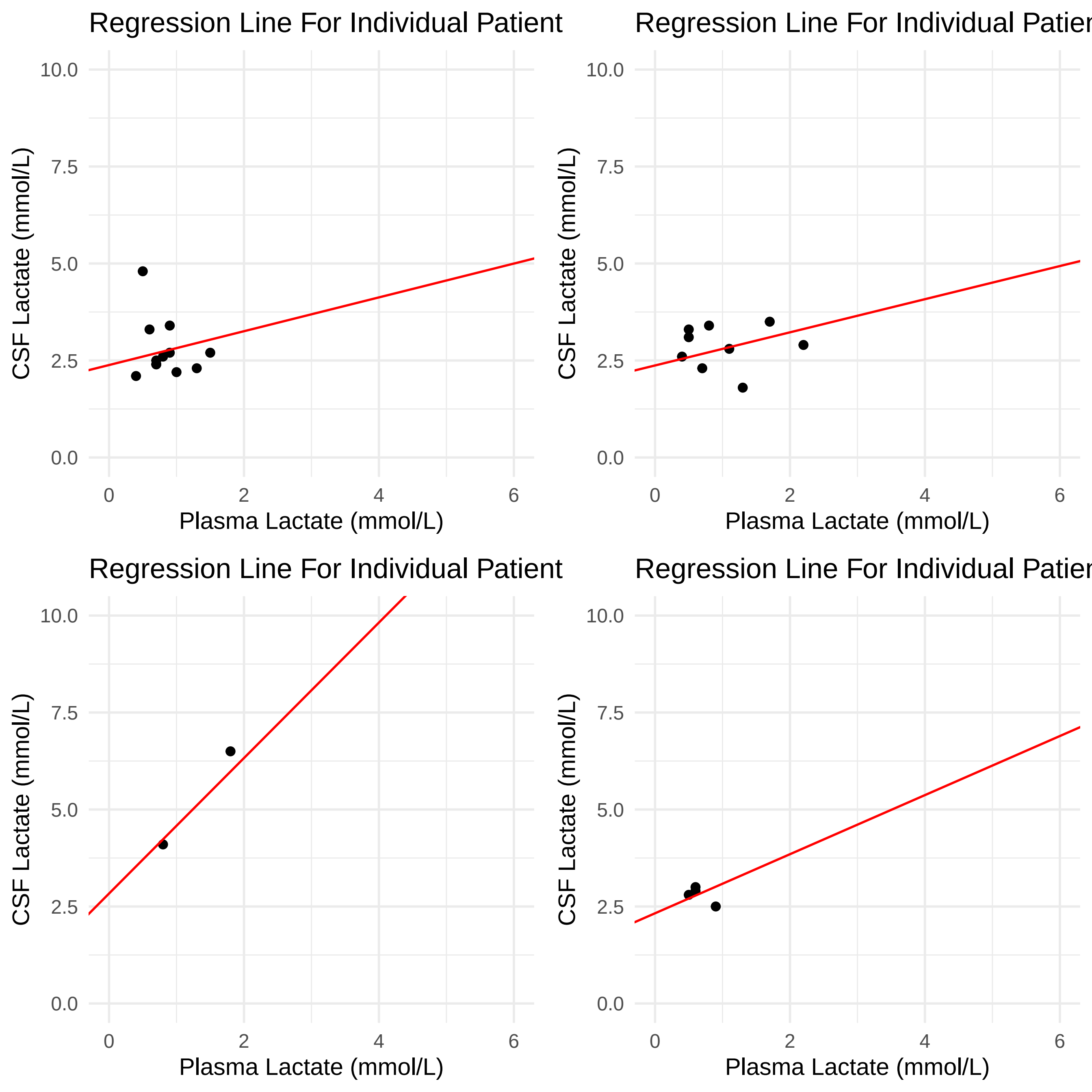

Regression Line For Individual Patient
10.0
7.5
5.0
2.5
0.0
CSF Lactate (mmol/L)
0
2
4
6
Plasma Lactate (mmol/L)
Regression Line For Individual Patien
10.0
7.5
5.0
2.5
0.0
CSF Lactate (mmol/L)
0
2
4
6
Plasma Lactate (mmol/L)
Regression Line For Individual Patient
10.0
7.5
5.0
2.5
0.0
CSF Lactate (mmol/L)
0
2
4
6
Plasma Lactate (mmol/L)
Regression Line For Individual Patien
10.0
7.5
5.0
2.5
0.0
CSF Lactate (mmol/L)
0
2
4
6
Plasma Lactate (mmol/L)

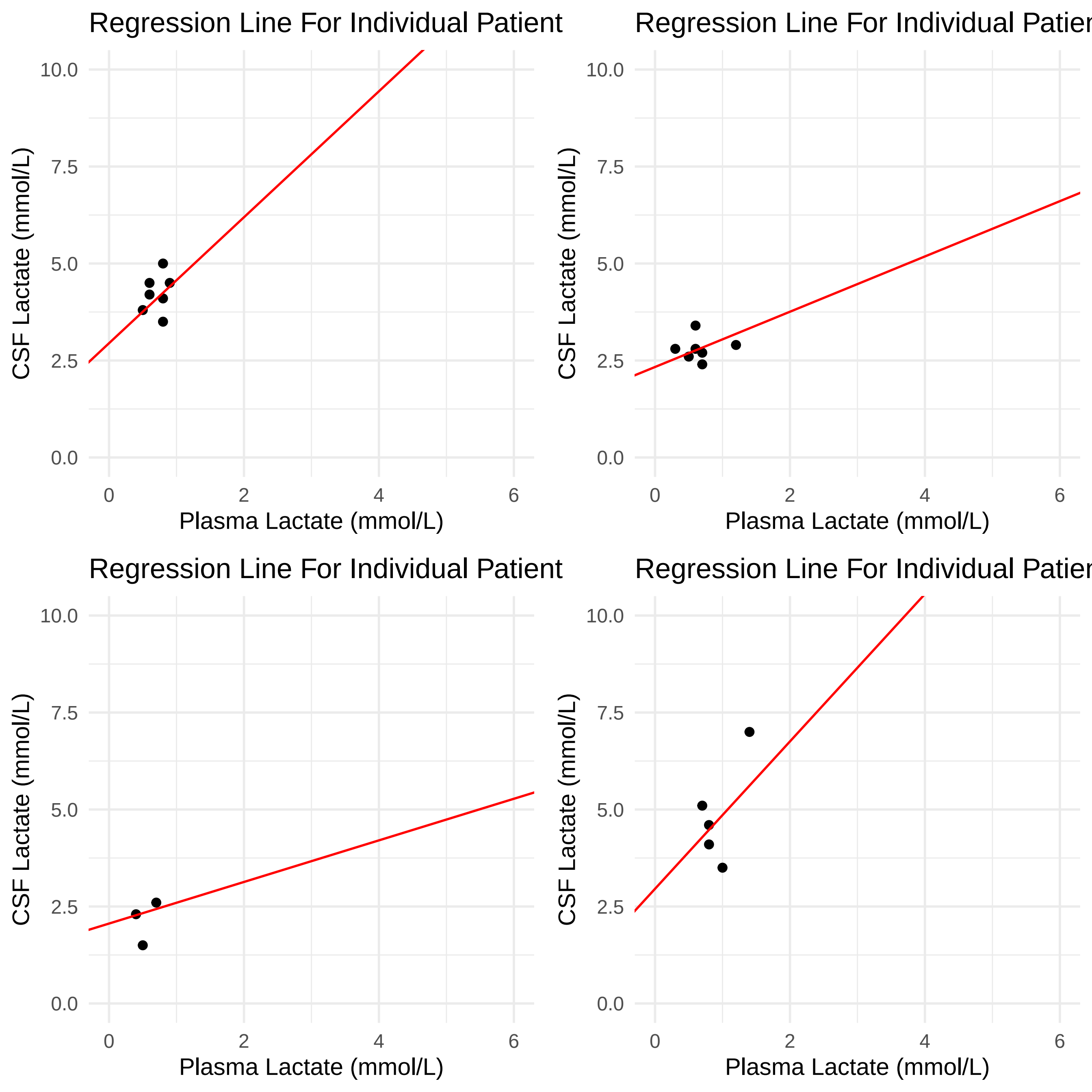

Regression Line For Individual Patient
CSF Lactate (mmol/L)
Plasma Lactate (mmol/L)
0.0
2.5
5.0
7.5
10.0
0
2
4
6
Regression Line For Individual Patien
CSF Lactate (mmol/L)
Plasma Lactate (mmol/L)
Regression Line For Individual Patient
CSF Lactate (mmol/L)
Plasma Lactate (mmol/L)
Regression Line For Individual Patien
CSF Lactate (mmol/L)
Plasma Lactate (mmol/L)

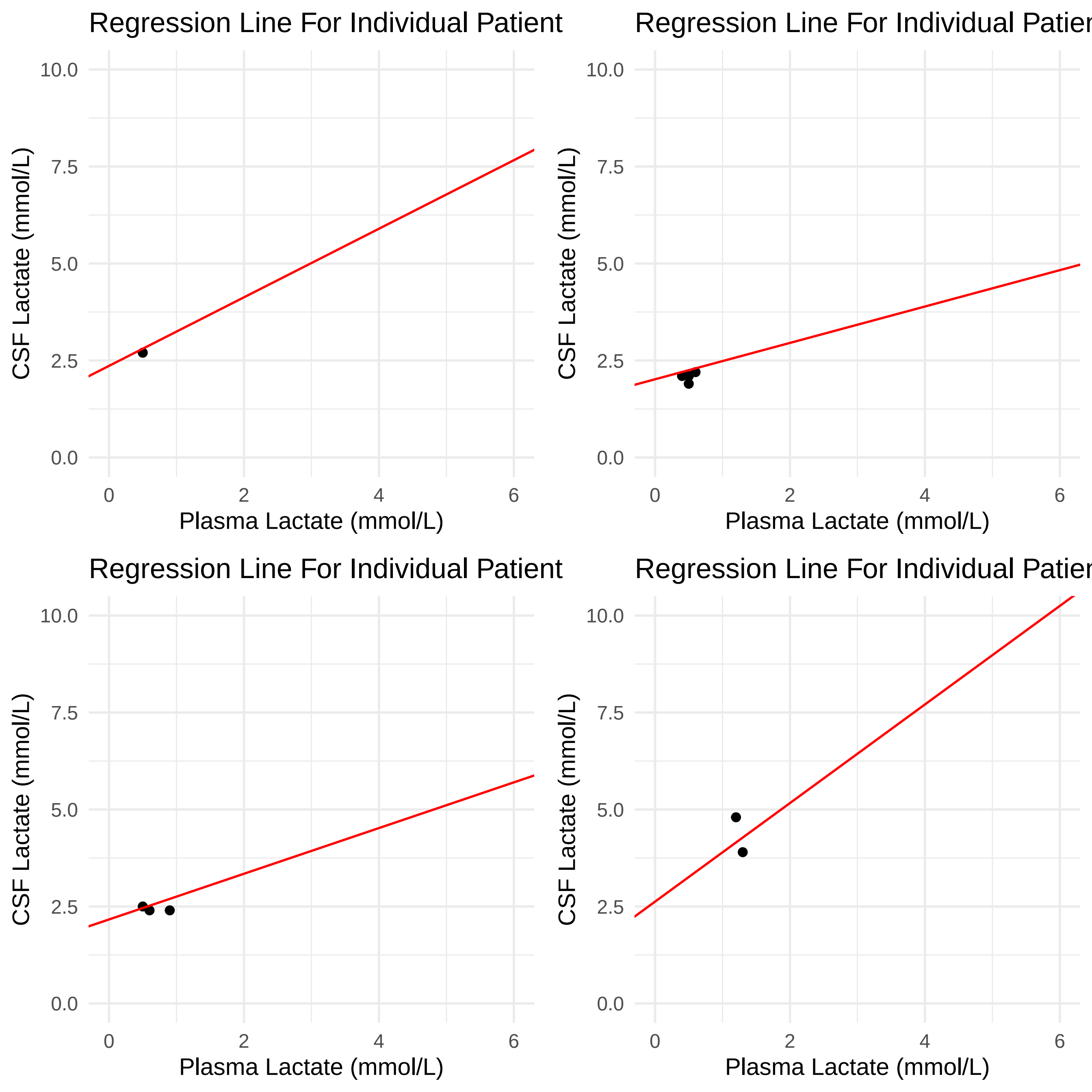
Regression Line For Individual Patient
CSF Lactate (mmol/L)
10.0
7.5
5.0
2.5
0.0
0
2
4
6
Plasma Lactate (mmol/L)
Regression Line For Individual Patien
CSF Lactate (mmol/L)
10.0
7.5
5.0
2.5
0.0
0
2
4
6
Plasma Lactate (mmol/L)
Regression Line For Individual Patient
CSF Lactate (mmol/L)
10.0
7.5
5.0
2.5
0.0
0
2
4
6
Plasma Lactate (mmol/L)
Regression Line For Individual Patien
CSF Lactate (mmol/L)
10.0
7.5
5.0
2.5
0.0
0
2
4
6
Plasma Lactate (mmol/L)

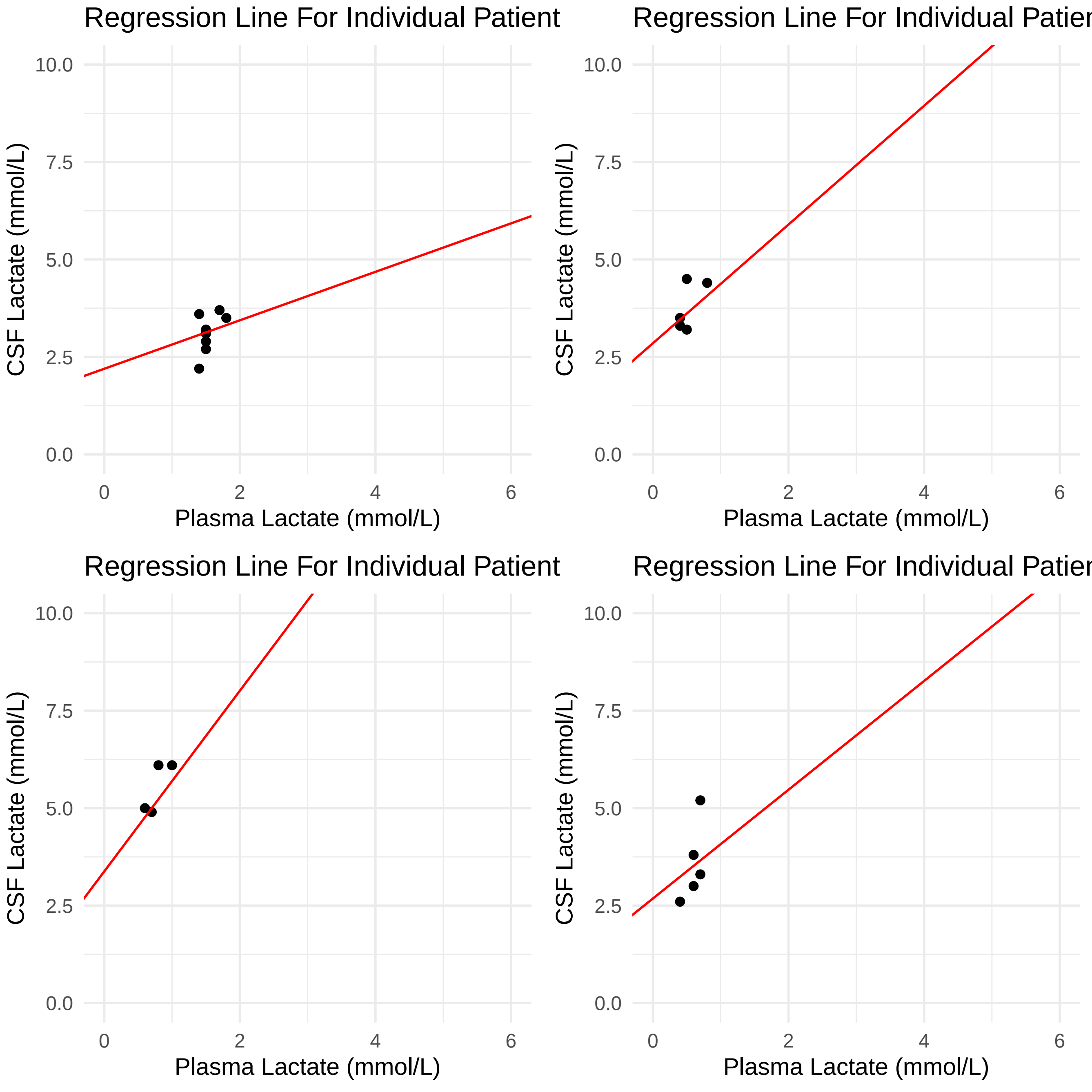
Regression Line For Individual Patient
Regression Line For Individual Patien
Regression Line For Individual Patient
Regression Line For Individual Patien
CSF Lactate (mmol/L)
Plasma Lactate (mmol/L)
0.0
2.5
5.0
7.5
10.0
0
2
4
6

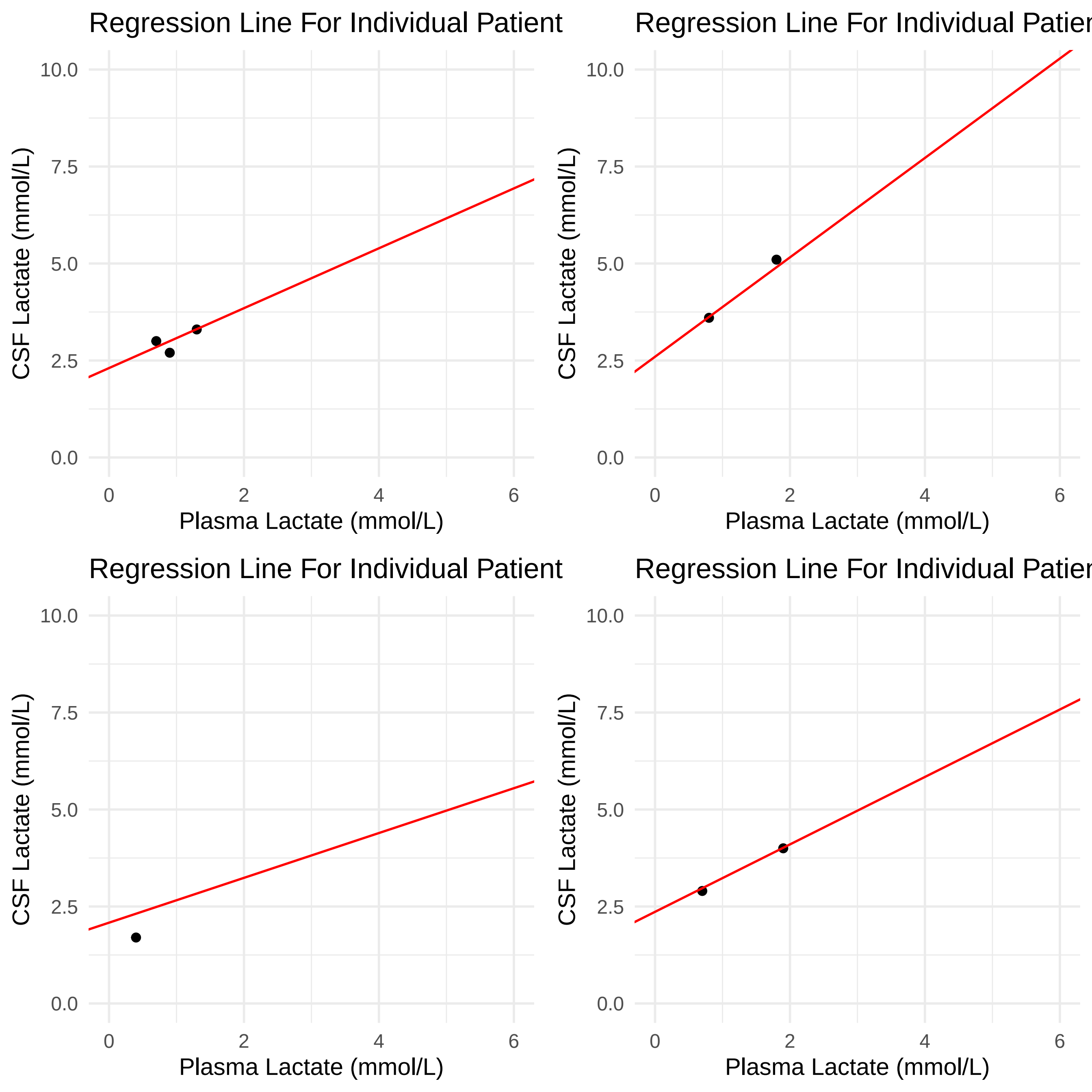

Regression Line For Individual Patient
CSF Lactate (mmol/L)
10.0
7.5
5.0
2.5
0.0
0
2
4
6
Plasma Lactate (mmol/L)
Regression Line For Individual Patien
CSF Lactate (mmol/L)
10.0
7.5
5.0
2.5
0.0
0
2
4
6
Plasma Lactate (mmol/L)
Regression Line For Individual Patient
CSF Lactate (mmol/L)
10.0
7.5
5.0
2.5
0.0
0
2
4
6
Plasma Lactate (mmol/L)
Regression Line For Individual Patien
CSF Lactate (mmol/L)
10.0
7.5
5.0
2.5
0.0
0
2
4
6
Plasma Lactate (mmol/L)

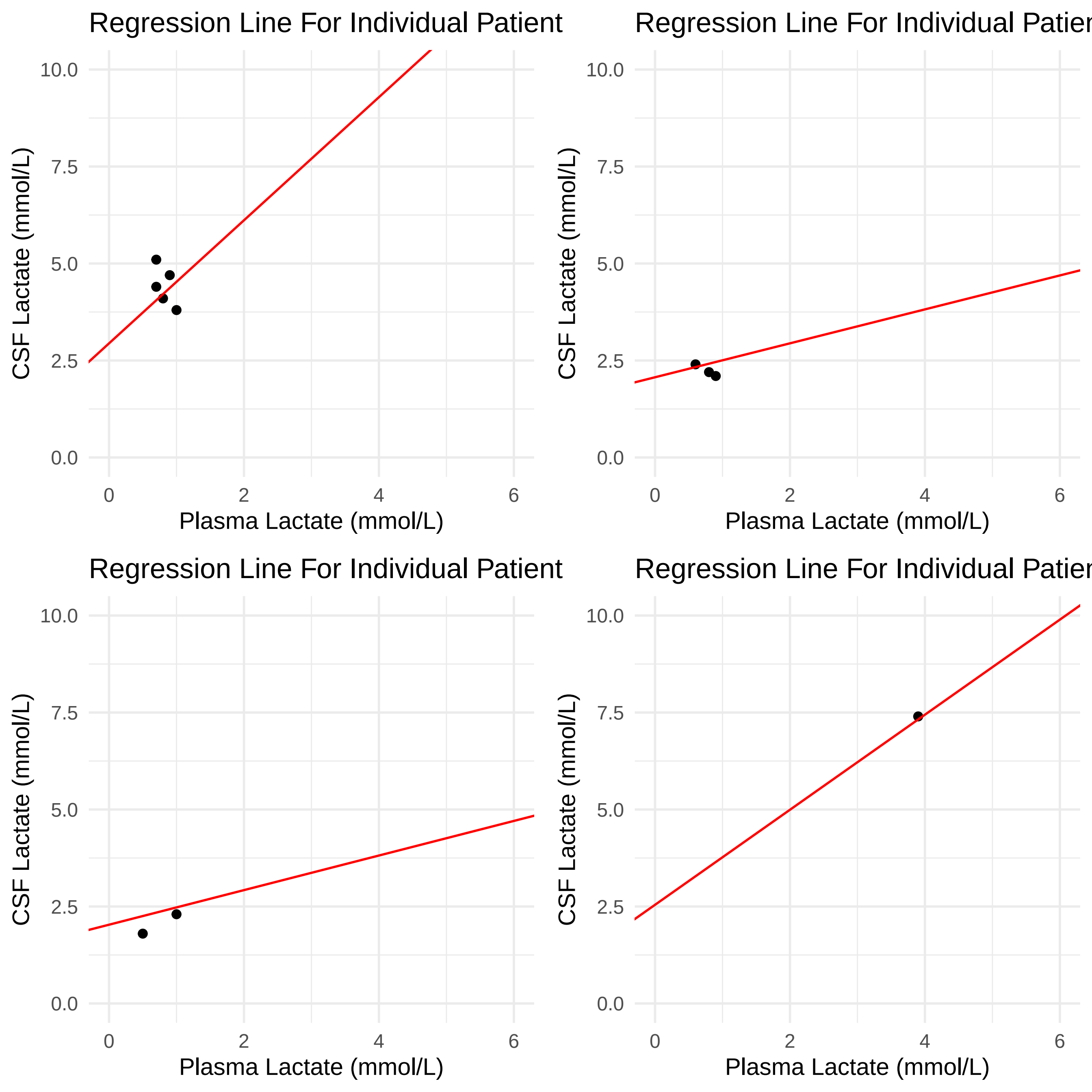

Regression Line For Individual Patient
CSF Lactate (mmol/L)
10.0
7.5
5.0
2.5
0.0
0
2
4
6
Plasma Lactate (mmol/L)
Regression Line For Individual Patien
CSF Lactate (mmol/L)
Plasma Lactate (mmol/L)
Regression Line For Individual Patient
CSF Lactate (mmol/L)
Plasma Lactate (mmol/L)
Regression Line For Individual Patien
CSF Lactate (mmol/L)
Plasma Lactate (mmol/L)

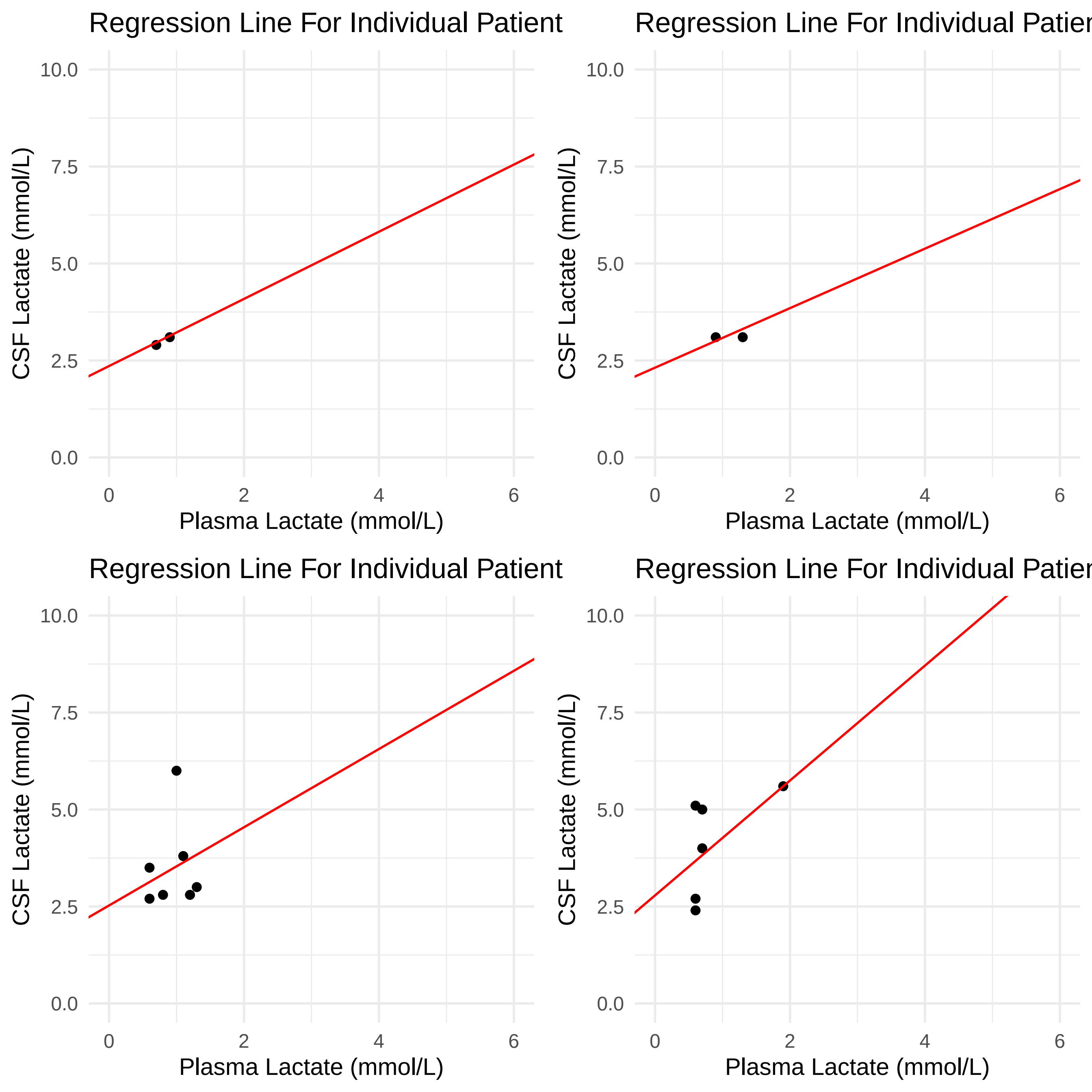

Regression Line For Individual Patient
Regression Line For Individual Patien
Regression Line For Individual Patient
Regression Line For Individual Patien
CSF Lactate (mmol/L)
Plasma Lactate (mmol/L)
0.0
2.5
5.0
7.5
10.0
0
2
4
6

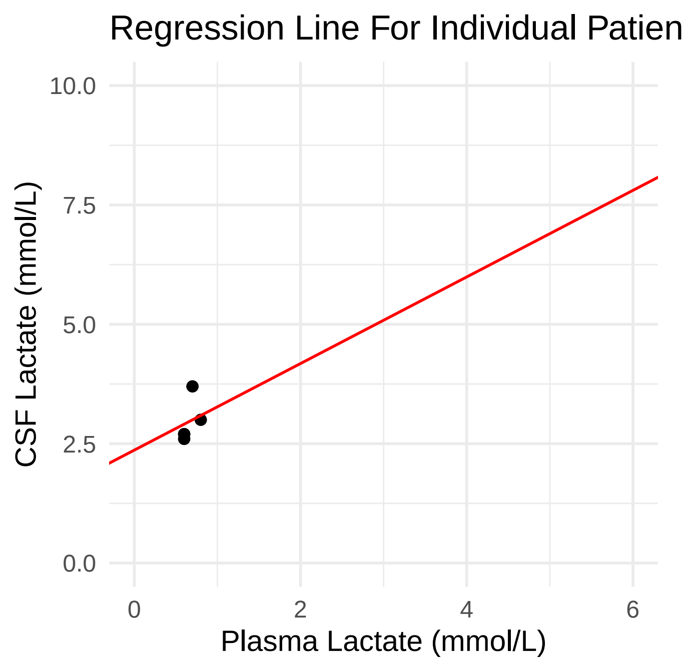
Regression Line For Individual Patient
CSF Lactate (mmol/L)
10.0
7.5
5.0
2.5
0.0
0
2
4
6
Plasma Lactate (mmol/L)

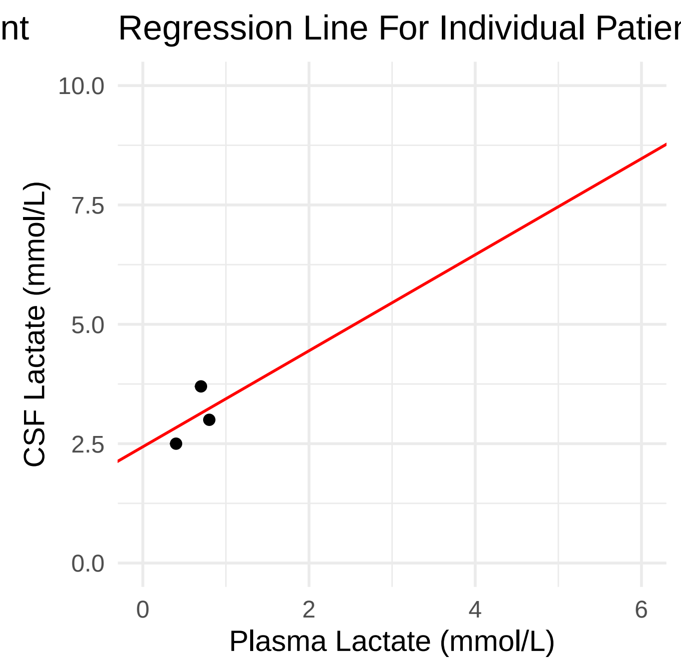
Regression Line For Individual Patie
CSF Lactate (mmol/L)
10.0
7.5
5.0
2.5
0.0
0
2
4
6
Plasma Lactate (mmol/L)

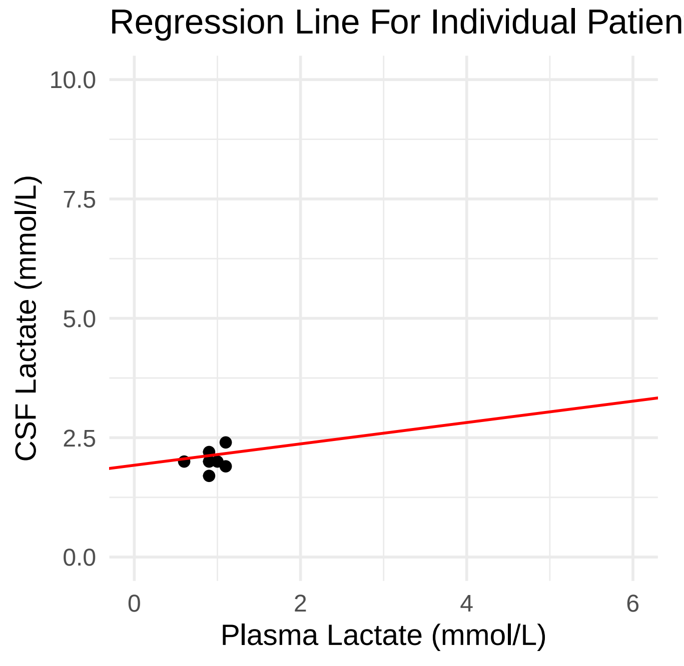
Regression Line For Individual Patient
CSF Lactate (mmol/L)
10.0
7.5
5.0
2.5
0.0
0
2
4
6
Plasma Lactate (mmol/L)
